# Methylation-Regulated Long Non-Coding RNA Expression in Ulcerative Colitis

**DOI:** 10.3390/ijms241310500

**Published:** 2023-06-22

**Authors:** Christopher G. Fenton, Mithlesh Kumar Ray, Wei Meng, Ruth H. Paulssen

**Affiliations:** 1 Clinical Bioinformatics Research Group, Department of Clinical Medicine, UiT-The Arctic University of Norway, N-9037 Tromsø, Norway; christopher.fenton@uit.no (C.G.F.); wei.meng@uit.no (W.M.); 2 Genomic Support Centre Tromsø (GSCT), Department of Clinical Medicine, UiT-The Arctic University of Norway, N-9037 Tromsø, Norway

**Keywords:** long non-coding RNAs, DNA methylation, ulcerative colitis, epigenetics

## Abstract

Long non-coding RNAs (lncRNAs) have been shown to play a role in the pathogenesis of ulcerative colitis (UC). Although epigenetic processes such as DNA methylation and lncRNA expression are well studied in UC, the importance of the interplay between the two processes has not yet been fully explored. It is, therefore, believed that interactions between environmental factors and epigenetics contribute to disease development. Mucosal biopsies from 11 treatment-naïve UC patients and 13 normal controls were used in this study. From each individual sample, both whole-genome bisulfite sequencing data (WGBS) and lncRNA expression data were analyzed. Correlation analysis between lncRNA expression and upstream differentially methylated regions (DMRs) was used to identify lncRNAs that might be regulated by DMRs. Furthermore, proximal protein-coding genes associated with DMR-regulated lncRNAs were identified by correlating their expression. The study identified UC-associated lncRNAs such as MIR4435-2HG, ZFAS1, IL6-AS1, and Pvt1, which may be regulated by DMRs. Several genes that are involved in inflammatory immune responses were found downstream of DMR-regulated lncRNAs, including SERPINB1, CCL18, and SLC15A4. The interplay between lncRNA expression regulated by DNA methylation in UC might improve our understanding of UC pathogenesis.

## 1. Introduction

Ulcerative colitis (UC) is a relapsing chronic inflammatory disease of the colon and one of the most common conditions of inflammatory bowel disease (IBD) [1]. The development of UC is influenced by a complex interplay between the host immune system, genetic variation, intestinal microbiota, and environmental factors [2,3]. The link between environmental factors and the genome is thought to be via epigenetic mechanisms, including DNA methylation [4], histone modifications [5], and interactions with non-coding RNAs [6]. Methylation can alter the expression of genes associated with UC pathogenesis [7,8,9].

Long non-coding RNAs (lncRNAs) are transcripts that are longer than 200 nt and have no protein-coding capacity. LncRNAs have multiple mechanisms to regulate gene expression including the modulation of transcription, mRNA stability, translation, and protein subcellular location by interacting with DNA, RNA, or protein to form large complexes [10]. LncRNAs have been shown to play a significant role in various biological processes including the regulation of gene expression, epigenetic regulation, and disease development [10]. Several studies have identified lncRNAs playing a role in the disease development and pathogenesis of UC [11,12,13,14,15,16,17]. DNA methylation is a key regulator of gene expression and contributes to lncRNA expression [18].

The interplay between DNA methylation and lncRNA expression has been implicated in various biological processes, including embryonic development, cancer, and neurological disorders [19,20,21]. The interplay between lncRNAs and methylation is not limited to promoter methylation but represents part of a complex regulatory network [21]. Like protein-coding genes, the transcription of lncRNAs can be affected by promoter methylation [22]. LncRNAs may in turn regulate the epigenome by interacting with different epigenetic factors including DNMTs or other genes involved in chromatin organization [23]. The crosstalk between DNA methylation and lncRNAs has been confirmed by findings regarding lncRNA promoter methylation and dysregulation in response to methylation inhibitor treatments [21]. Changes in the promoter methylation state cause the significant dysregulation of many lncRNAs, including Pvt1, NEAT1, and LINC00261, and play a role in disease pathogenesis [21,24]. This study focuses on lncRNAs that may be regulated by differentially methylated regions (DMRs).

This study aims to provide valuable knowledge for future functional studies of lncRNAs associated with UC pathogenesis.

## 2. Results

**Figure 1:**
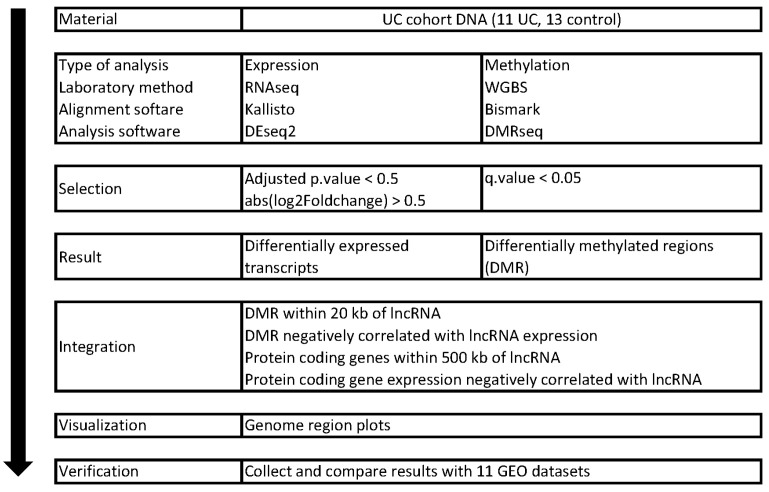
Schematic overview of material, methods, and software used in the study.

A schematic overview of the methods and software used to generate the results used in this study is presented in Figure 1. The study workflow comprised several steps: WGBS (whole-genome bisulfite sequencing) data were aligned to the human reference genome using Bismark, and RNAseq fastq files were aligned to the human reference transcriptome using Kallisto. Differentially methylated regions (DMR) and differentially expressed (DE) transcripts were identified using DMRSeq and DESeq2, respectively. Using correlation analysis between lncRNA expression and adjacent DMR methylation levels, potentially methylation-regulated lncRNAs were selected. Methylation-regulated lncRNA expression was correlated with adjacent protein-coding transcript expression to predict target protein-coding genes for selected lncRNAs. The obtained results were visualized using Gviz and verified with ten other GEO UC datasets.

### 2.1. Identification of Differentially Expressed Transcripts with DESeq2

DEseq2 was run on the transcript count matrix generated by the Kallisto aligner on raw Illumina fastq reads, generated from 11 treatment-naïve mucosal biopsy UC samples and 13 control samples. A total of 1292 lncRNAs had an adjusted *p*-value less than 0.05 and an absolute fold change value greater than 0.5.

### 2.2. Identification of Differentially Methylated Regions (DMRs) with DMRseq

A total of 5796 DMRs were obtained with a q-value < 0.05 in the UC samples (n = 11) compared with the normal control group (n = 13). The DMRs included 1380 hypermethylated and 4416 hypomethylated regions (Table S1). The average size of the DMRs was 288 bp, and the average number of CpGs in the DMRs was 15.

### 2.3. LncRNAs That May Be Regulated by DMRs

LncRNAs that were within 20 kb upstream or downstream of a DMR and whose expression negatively correlated with DMR methylation levels were considered lncRNAs that are potentially regulated by a proximal DMR. A total of 254 lncRNAs met the above criteria. A total of 188 lncRNA were upregulated in UC, and 66 were downregulated in UC (Table S2).

### 2.4. Proteins That May Be Influenced by DMR-Regulated LncRNAs

**Figure 2:**
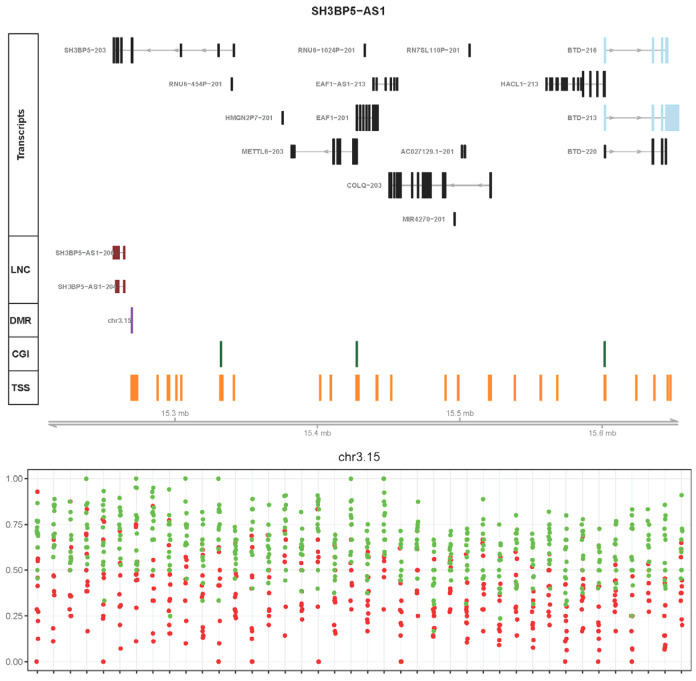
Example of a genomic region containing a differentially methylated region (DMR) chr3.15 and differentially expressed (DE) lncRNA SH3BP5-AS1 transcripts. The top transcript track represents the regions found between the DMR, lncRNA transcripts, and DE protein-coding transcripts of interest. Transcripts indicated in light blue denote DE protein-coding transcripts that may be influenced by DMR-regulated lncRNA transcripts, which are shown in brown. Transcripts indicated in black are the largest transcripts for each gene found within the region. The LNC track denotes the position of the DE lncRNA transcripts; the DMR track denotes the position of the DMR, which is shown in purple. The CGI track denotes the position of known CpG islands, which are shown in green. The TSS (transcription starting site) track denotes the position of known TSSs, which are shown in orange. The bottom track of the top panel shows the approximate distance in Mb. The bottom panel shows the relative methylation levels for the chr3.15 DMR. Red dots indicate the relative methylation values of the UC samples. The relative methylation values from the control samples are indicated in green.

**Figure 3:**
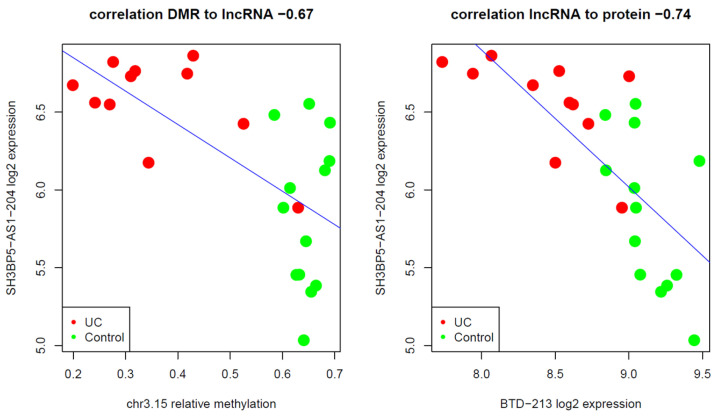
An example of correlations between sample DMR methylation levels, lncRNA, and adjacent protein-coding transcript expressions. On the left, the correlation between differentially expressed (DE) lncRNA transcript SH3BP5-AS1-204 and the mean-sample relative methylation levels of DMR chr3.15. On the right is the correlation between DE lncRNA transcript SH3BP5-AS1-204 expression and proximal protein-coding DE BDT transcripts.

Differentially expressed protein-coding genes that were within 500 kb upstream or downstream of a DMR-regulated lncRNA were considered for correlational expression analysis. A total of 244 protein-coding genes were found whose expressions were significantly and negatively correlated with lncRNA expression. This discussion focuses on those genes that may play a role in UC pathogenesis. Of the above proteins, 110 were upregulated in UC, and 134 were downregulated in UC versus the control. The results are summarized in Tables S3 and S4. Figure 2 shows an example of a genomic region containing a DMR, DE lncRNA transcripts, and DE protein-coding transcripts. An example of the correlation between the DMRs, DE lncRNA transcripts, and adjacent DE protein-coding transcripts is shown in Figure 3. All genomic regions of interest can be seen in Figure S1.

### 2.5. Cell Deconvolution

**Figure 4:**
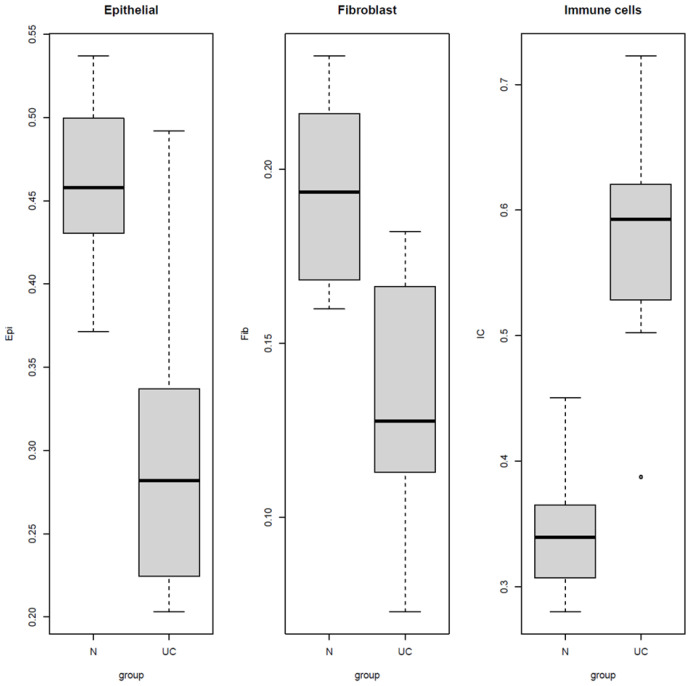
Box plots of fractions of cell types present in normal and UC tissue samples. Each plot indicates a significant difference in cell distribution between UC and normal samples. The Y-axis depicts cell fractions of tissue samples ranging from 0 to 1. The X-axis indicate the range of cell fractions in control (N) and UC samples.

To estimate types of cell fractions in UC and the normal controls’ mucosal tissues, the EpiDISH cell deconvolution algorithm was adapted for use with methylation data. The deconvolution estimated relative fractions of epithelial, fibroblast, and immune cells present in the tissue samples. A cell-type fraction estimate revealed increased fractions of immune cells in tissues from UC patients, whereas fractions of epithelial cells and fibroblasts were increased in the control samples (Figure 4).

### 2.6. Verification of DMR-Regulated lncRNAs and Proximal Proteins

To help verify the correlation between lncRNAs and adjacent protein expression, normalized matrices from 11 datasets were collected: GSE109142, GSE128682, GSE206285, GSE36807, GSE38713, GSE47908, GSE13367, GSE16879, GSE48958, GSE59071, and GSE73661. A total of 35 lncRNAs showed a significant correlation with adjacent protein expression in at least one dataset (Table S5). An overview of the number of samples in each GEO dataset, as well as sample locations, is shown in Table S6.

## 3. Discussion

Environmental factors have been implicated in both the incidence of UC and the likelihood of relapse in UC patients [25] and are thought to have a direct effect on the epigenome, including the expression of lncRNAs and methylation status [26]. Both lncRNA and DNA methylation have been shown to regulate the transcription of protein-coding genes [18]. However, the interplay between DNA methylation, the expression of lncRNAs, and the expression of protein-coding genes has not been explored in detail in UC.

The focus of this study was to identify lncRNAs that were negatively correlated with adjacent DMR methylation levels. The implication is that elevated levels of DMR methylation (hypermethylation) in UC samples should result in lower adjacent lncRNA expression and vice versa (hypomethylation). To explore the possible cis effects of these lncRNAs, neighboring DE protein-coding genes whose expression negatively correlated with lncRNA expression were identified. This ensures that lncRNAs and adjacent protein-coding genes are unlikely to be regulated by the same DMR. Defining the lncRNA cis-regulation of gene expression is difficult, as lncRNAs have been shown to regulate the expression of both proximal and distal genes [27]. Recent reports suggest that the 3D conformation of the genome guides lncRNAs to distal binding sites [28]. Therefore, several studies have considered the possible effects of lncRNA expression on genes within 500 kb of lncRNAs [29,30].

Recent publications have shown that methylation events outside 1–2 kb of the promoter can have effects on gene expression. It has been shown that increasing the range queried from 5 kb to 20 kb can add an additional ~0.5% of DEGs that associate with the identified DMRs [31]. Therefore, the influence of methylation on lncRNA expression in DMRs within 20 kb was considered.

The results identified protein-coding genes and lncRNAs that were previously associated with UC. Protein-coding genes adjacent to possible DMR-regulated lncRNAs include chemokine C-C motif ligand 18 (CCL18), potassium voltage-gated channel subfamily B member 1 (KCNB1), and serpin family B member 1 (SERPINB1). The increased expression of CCL18, which has been linked to inflammation and the migration of T cells, is correlated with the expression of lncRNA AC244100.3 [32]. KCNB1 is correlated with DE lncRNA ZFAS1 and is downregulated in active UC. KCNB1 regulates the cellular K^+^-efflux necessary for enterocyte apoptosis and has been proposed as a therapeutic target for IBD [33]. In addition, KCNB1 has been identified in several cancers, including gastric and colorectal cancers (CRC). KCNB1 is downregulated in both CRC and gastric cancers [34,35]. The expression of lncRNA GMDS-DT is correlated with the expression of neutrophil elastase (NE) inhibitor protein-coding gene SERPINB1. In UC, activated neutrophils secrete NE, which plays a key role in colonic epithelial cell destruction. The increased expression levels of SERPINB1 might protect colonic epithelial cells by reducing NE activity [36].

Potentially DMR-regulated lncRNAs have been implicated in immunity, inflammation, and IBD, including AC007750.1 (lnc-SLC4A10-7), SH3BP5 antisense RNA 1 (SH3BP5-AS1), FOXD2-adjacent opposite strand RNA 1 (FOXD2-AS1), mir4435-2 host gene (MIR4435-2HG), and cytoskeleton regulator RNA (CYTOR). The expression of AC007750.1 is correlated with DPP-4 (dipeptidyl peptidase-4) expression, which is a potential biomarker for IBD. DPP-4 stimulates the production and release of cytokines, chemokines, and neuropeptides, thereby playing a role in the inflammatory response [37,38]. LncRNA SH3BP5-AS1 is correlated with biotinidase (BTD). The association between DMR, SH3BP5-AS1, and BTD is shown in Figure 2 and Figure 3. Biotin deficiency plays a role in the induction of Th1- and TH17-mediated proinflammatory responses [39]. The observed downregulation of BTD in UC may result in the dysfunction of cellular immune responses [40].

A reduction in FOXD2-AS1 expression correlates with an upregulation of PDZK1-interacting protein 1 (PDZK1IP1) in UC, which may contribute to the inflammatory responses associated with UC [41].

The dysregulation of MIR4435-2HG in UC might play a key role in the inflammatory process and has been shown to be associated with CRC [37,42,43]. MIR4435-2HG is correlated with the expression of B cell lymphoma 2 (Bcl-2)-interacting protein (BCL2L11), which is associated with an increase in apoptosis resistance, resulting in impaired epithelial cell turnover [44]. In addition, BCL2L11 also plays a major role in immune tolerance in UC [45]. CYTOR plays a role in promoting inflammation and epithelial–mesenchymal transition, ultimately promoting cellular invasion and CRC progression [46]. The expression of lncRNA CYTOR is correlated with the expression of FABP1, which is involved in the intestinal absorption of dietary long-chain fatty acids [47]. The dysregulation of CYTOR may disrupt FABP1-mediated fatty acid metabolism, which has been implied to contribute to the pathophysiology of UC [48,49].

Tissue samples are heterogeneous, and DNA methylation is a highly cell-type-specific event [50]. Therefore, EpiDISH cell deconvolution was adapted for use with methylation data and used to estimate cell-type fractions in both UC and control samples (Figure 2). EpiDISH was chosen simply because over 70% of the DMR sites overlapped known Illumina EPIC array sites. EPIC arrays are widely used to study methylation. The distribution of cell fractions was consistent with previous deconvolution results obtained from transcriptomic analysis of active UC [42]. The reduced epithelial fraction may be indicative of cell degradation, which is a major characteristic of UC [51].

Our results show several potentially DMR-regulated lncRNAs associated with epithelial cell proliferation and migration, including HOXA-AS2 and HOXA-AS3 [52,53]. Interestingly, these lncRNAs are under DMR regulation and are downregulated in UC. The downregulation of HOXA-AS2 and HOXA-AS3 may reduce epithelial cell differentiation and migration during UC. The increased proportion of immune cells in the colon of patients with UC is due to the recruitment and activation of these cells in response to ongoing inflammation in the gut [54]. The epigenetically upregulated lncRNAs ADORA2A-AS1 [55] and IL6-AS1 [56] may be associated with immune cell infiltration, which is a characteristic of inflammation. These potentially DMR-regulated lncRNAs may help explain the higher abundance of immune cells in UC patients. Several of the DMR-regulated lncRNA expressions in this study were found to be differentially expressed in UC in our previous study (114 of 254) [17].

Verifying results in GEO (Gene Expression Omnibus) is difficult. No independent datasets with both methylation levels and gene expression levels for UC could be found. Therefore, an attempt was made to see if significant negative correlations between the lncRNAs and adjacent expression of protein-coding genes could be found in 11 published UC GEO datasets. Comparing annotations between GEO datasets is difficult, as recently annotated lncRNAs such as AL359962 simply do not appear in previously deposited microarray datasets, leaving approximately 58 lncRNAs that could be found in at least 1 of the 11 UC–control GEO datasets. Another challenge is that several of the 11 GEO datasets selected to verify the correlation between lncRNAs and adjacent protein-coding genes were samples collected from locations other than mucosal biopsies, including the ileum, the rectum, etc. (Table S6). For 35 lncRNAs, at least 1 GEO set confirmed a significant correlation between the lncRNA and adjacent protein expression (Table S5). For the 35 lncRNAs, a significant correlation was found, on average, in 25% of the datasets. Given the diversity of the GEO datasets, this represents a positive result. The normalized count matrix for this experiment can be found in Supplementary Table S7.

As a limitation of this work, it is hereby noted that the results presented are derived from in silico analysis and need experimental validation in the future.

## 4. Materials and Methods

### 4.1. Study Cohort

The study cohort comprised mucosal biopsies from patients with newly diagnosed, treatment-naïve UC with mild-to-moderate disease (n = 11) and control subjects (n = 13). Tissue samples from subjects which underwent cancer screening and showed normal colonoscopy and normal colonic histological examinations, served as controls. UC was diagnosed based on established clinical endoscopic and histological criteria, as defined by ECCO guidelines [57]. The grade of inflammation was assessed during colonoscopy using the UC disease activity index (UCDAI) endoscopic sub-score, with 3 to 10 indicating mild-to-moderate disease [58]. The biopsies from UC samples showed clinical scores of 8.2 ± SD 1.3 and endoscopic scores of 1.9 ± SD 0.5. The biopsies from the control subjects showed normal colonoscopies, colon histology, and immunochemistry, with clinical and endoscopic scores of 0. All biopsies were taken from the sigmoid part of the colon. The age distribution within the groups was 39 ± SD 12 years in the UC group and 53 ± SD 18 in the control subjects. The gender distribution was 7 males and 4 females in the UC group and 11 males and 2 females in the control group. The samples were taken from an established Biobank approved by the Norwegian Board of Health. The participants signed an informed and written consent form. The study was approved by the Regional Ethics Committee of North Norway and Norwegian Social Science Data Services (REK Nord 2012/1349). The raw fastq files of the transcriptomes were generated previously (GSE 128682), and raw WGBS fastq files from a previously published work were used [7]. However, to obtain optimal results, only the highest-coverage WGBS samples were included in the cohort of this study. Both transcriptomic data and data obtained by WGBS were reanalyzed for this manuscript, with a newer human genome build (GENCODE V38).

### 4.2. DNA and RNA Isolation

Both DNA and RNA were isolated using the Allprep DNA/RNA Mini Kit from Qiagen (Cat no: 80204) and the QIAcube instrument (Qiagen, Venlo, The Netherlands) according to the manufacturer’s protocol. RNA and DNA quantity and purity were assessed as previously described [7,42]. All RNA samples used for analyses had a RIN value between 8.0 and 10.0. DNA and RNA samples were kept at −70 °C until further use.

### 4.3. Library Preparation and Next-Generation Sequencing

Library preparations and sequencing were conducted as described previously [7,42].

### 4.4. Preprocessing of Data

The human reference genome hg38 was downloaded from GENCODE and indexed using Bismark version 0.22.3. The data from each sample were then aligned to the indexed reference genome using the Bowtie2 aligner within Bismark. The methylation level in each cytosine was then determined using Bismark with the following parameters: −gzip –bedGraph—cytosine_report –no_overlap—buffer_size 10 G –paired –ignore 3 –ignore_r2 3 ––ignore_3prime_r2 2. Methylation data output contained read coverage and the percentage of methylated cytosine at each cytosine position of the genome.

### 4.5. Identification of DMRs

The R DMRseq package (version 1.4.9) was used to find differentially methylated regions (DMRs) between UC samples and normal samples from the Bismark output files. CpG sites with less than 6× coverage were set to 0 prior to DMRseq analysis, and only CpG sites with a minimum of 6× coverage in 50% of both groups were kept, as recommended by the software. DMRs with DMRseq q-values of less than 0.05 were considered significantly differentially regulated regions (Table S1).

### 4.6. Cell Deconvolution

To compare methylation with transcriptional cell deconvolution, the EpiDISH package in R (https://bioconductor.org/packages/release/bioc/html/EpiDISH.html, accessed on 21 January 2023) was adapted to estimate the relative proportions of different cell types present in a tissue sample. EpiDISH requires Illumina EPIC array identifiers and a matrix of beta values. DMRs were given EPIC array identifiers by overlapping DMR genomic positions with EPIC array positions. Approximately 70% of DMR locations overlapped within EPIC-array-annotated genomic positions. A matrix of the average relative methylation value per sample per DMR was used as the beta matrix. The Robust Partial Correlation (RPC) mode in EpiDISH was utilized to estimate the relative numbers of epithelial, fibroblast, and immune cells in each sample (UC and control).

### 4.7. RNAseq

Illumina-generated fastq sequences were aligned with a reference human transcriptome using the Kallisto RNA-seq aligner. The transcript read count table from the Kallisto output was imported into the DESseq2 R package for identifying differentially expressed transcripts. The lncRNA catalog was retrieved from GENCODE V38 using the transcript type “lncRNA”. Only transcripts with a DESeq2-adjusted *p*-value of < 0.05 and an absolute foldchange greater than 0.5. were considered differentially expressed DE transcripts. The vst function of the DESeq2 package was used to create a normalized count matrix in the correlational analyses.

### 4.8. Identifying lncRNAs That May Be under DMR Regulation

DMRs located within 20 kb of a DE lncRNA were considered for correlation analysis. The R cor.test package was used to calculate the correlation and correlational *p*-value between the mean-sample relative methylation and DE lncRNA-normalized transcript counts. Only DE lncRNAs whose transcript expressions were negatively correlated with DMR methylation levels (correlation *p*-value of < 0.05) were considered possible DMR-regulated lncRNAs (Table S2).

### 4.9. Identifying Proteins That May Be under DMR-Regulated lncRNA Regulation

Only differentially expressed protein-coding transcripts within 500 kb of the DMR-regulated lncRNAs were considered. The lncRNA expression was then correlated with the neighboring proteins using the R cor.test package. Only protein-coding transcripts that significantly negatively correlated (correlation *p*-value of < 0.05) with DMR-regulated lncRNA transcripts were considered (Table S3). The R Gviz package was used to help visualize the relationship between the DMR methylation level, lncRNA transcript expression, lncRNA-DMR correlation, CpG islands, and TSS (Figure S1). TSS annotation was downloaded from the refTSS database (http://reftss.clst.riken.jp/reftss/Main_Page, accessed on 17 December 20222). The CpG island positions of the human genome (hg38) were downloaded from the UCSC table browser (https://genome.ucsc.edu/cgi-bin/hgTables, accessed on 17 December 2022).

### 4.10. Verification of DMR-Regulated lncRNAs and Proximal Partners in Other GEO Datasets

To help verify the DMR-regulated lncRNA and proximal protein results, the normalized matrices of the UC and control samples from 11 UC datasets (GSE109142, GSE128682, GSE206285, GSE36807, GSE38713, GSE47908, GSE13367, GSE16879, GSE48958, GSE59071, and GSE73661) were used. Table S5 compares the expression of lncRNAs, and adjacent proteins found in this study with the above datasets. Specifically, other datasets where a significant negative correlation between lncRNAs and adjacent protein-coding regions could be found. Additional information about the mean difference in expression (UC vs. control) for lncRNAs and adjacent proteins is provided in Table S5. Background information about the GEO datasets can be found in Table S6, including the number of UC and control samples, and their origin.

## 5. Conclusions

This study suggests a fine-tuned and complex regulatory mechanism between methylation, lncRNAs, and protein expression in UC. The results might open new avenues for diagnostic or therapeutic strategies.

## Data Availability

The DESeq2 VST-normalized RNA-seq Kallisto transcript count matrix for the samples (Table S4) and all other data generated or analyzed during this study are included in the published article and Supplementary Materials. Regarding the availability of the DNA data, it is hereby noted that, according to Norwegian Health Research Act § 34, the processing of health information can only take place in accordance with the consent given. In this case, the availability of unprocessed DNA information would not be in accordance with the participants’ consent.

## Supplementary Materials

The following supporting information can be downloaded at: https://www.mdpi.com/article/10.3390/ijms241310500/s1.
