# Supplementary material for: Methylation-Regulated Long Non-Coding RNA Expression in Ulcerative Colitis
Source: Int J Mol Sci. 2023 Jun 22;24(13):10500. doi: 10.3390/ijms241310500 (PMC10341861; doi:10.3390/ijms241310500)
Supplement: Supplementary file 1 [file ijms-24-10500-s001.zip › Figure_S1.pdf]

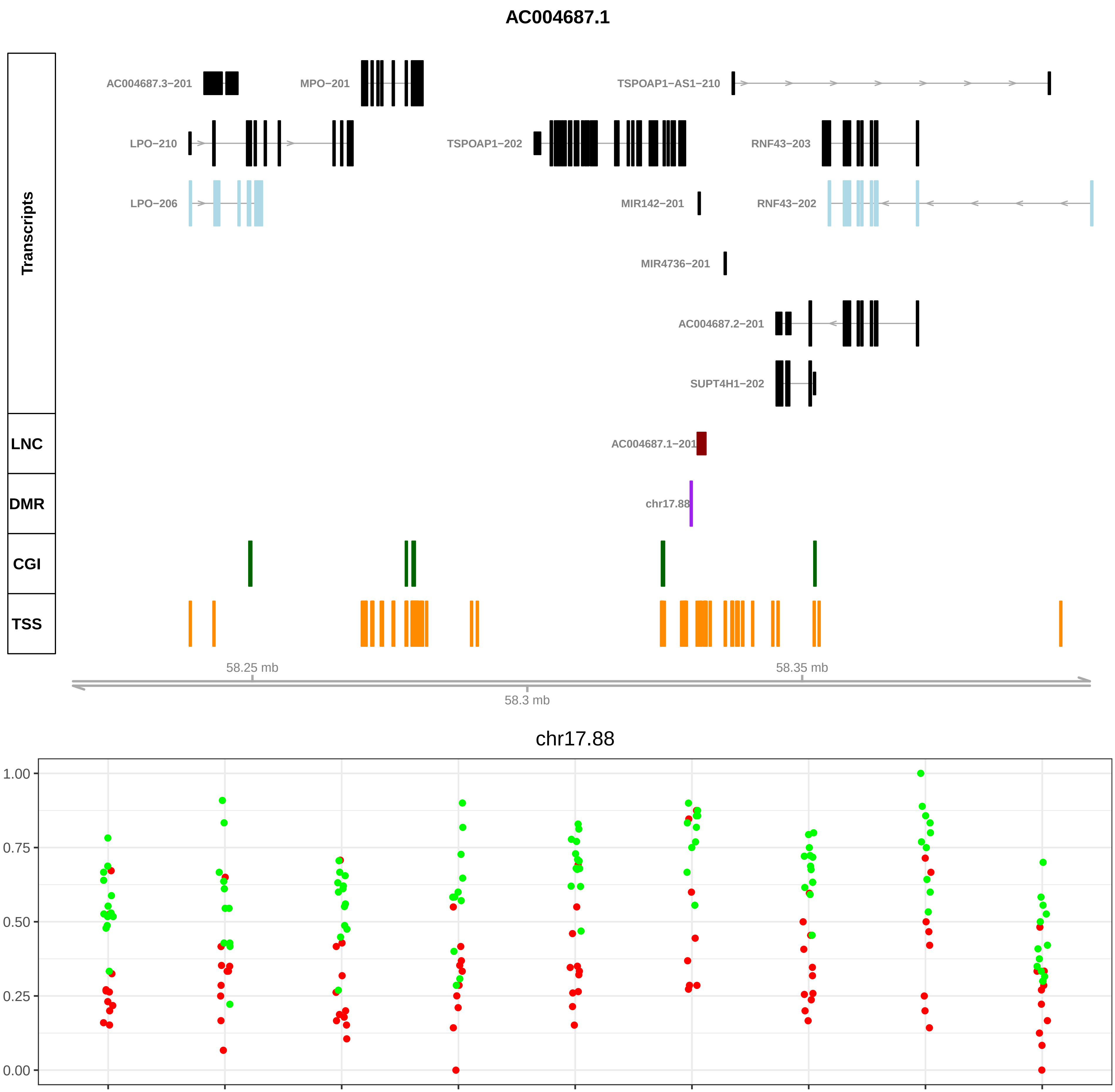

AC004687.1
Transcripts
LNC
DMR
CGI
TSS
AC004687.3−201
MPO−201
TSPOAP1−AS1−210
LPO−210
TSPOAP1−202
RNF43−203
LPO−206
MIR142−201
RNF43−202
MIR4736−201
AC004687.2−201
SUPT4H1−202
AC004687.1−201
chr17.88
58.25 mb
58.35 mb
58.3 mb
chr17.88
1.00
0.75
0.50
0.25
0.00

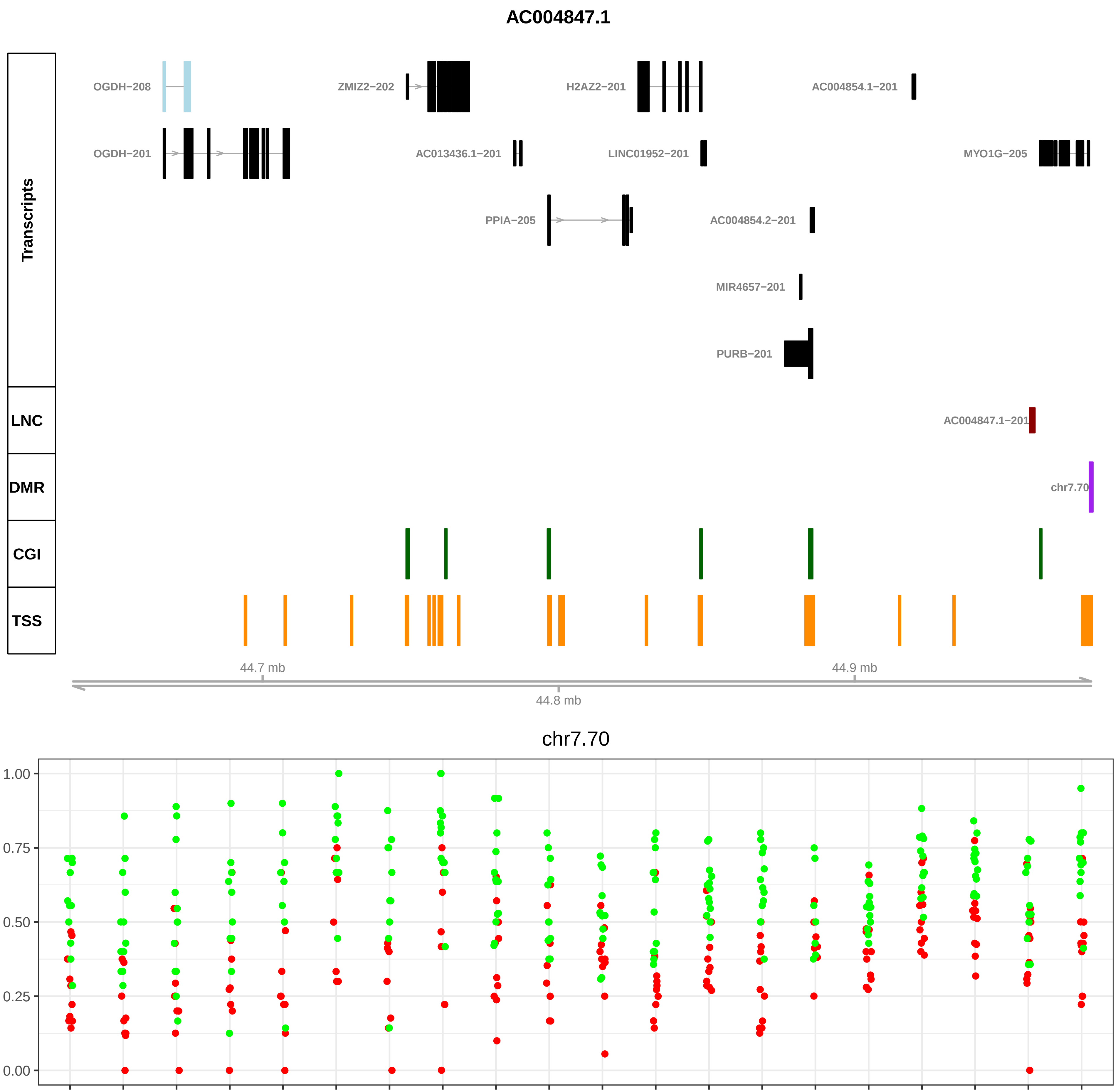

AC004847.1
Transcripts
OGDH−208
ZMIZ2−202
H2AZ2−201
AC004854.1−201
OGDH−201
AC013436.1−201
LINC01952−201
MYO1G−205
PPIA−205
AC004854.2−201
MIR4657−201
PURB−201
LNC
AC004847.1−201
DMR
chr7.70
CGI
TSS
44.7 mb
44.9 mb
44.8 mb
chr7.70
1.00
0.75
0.50
0.25
0.00

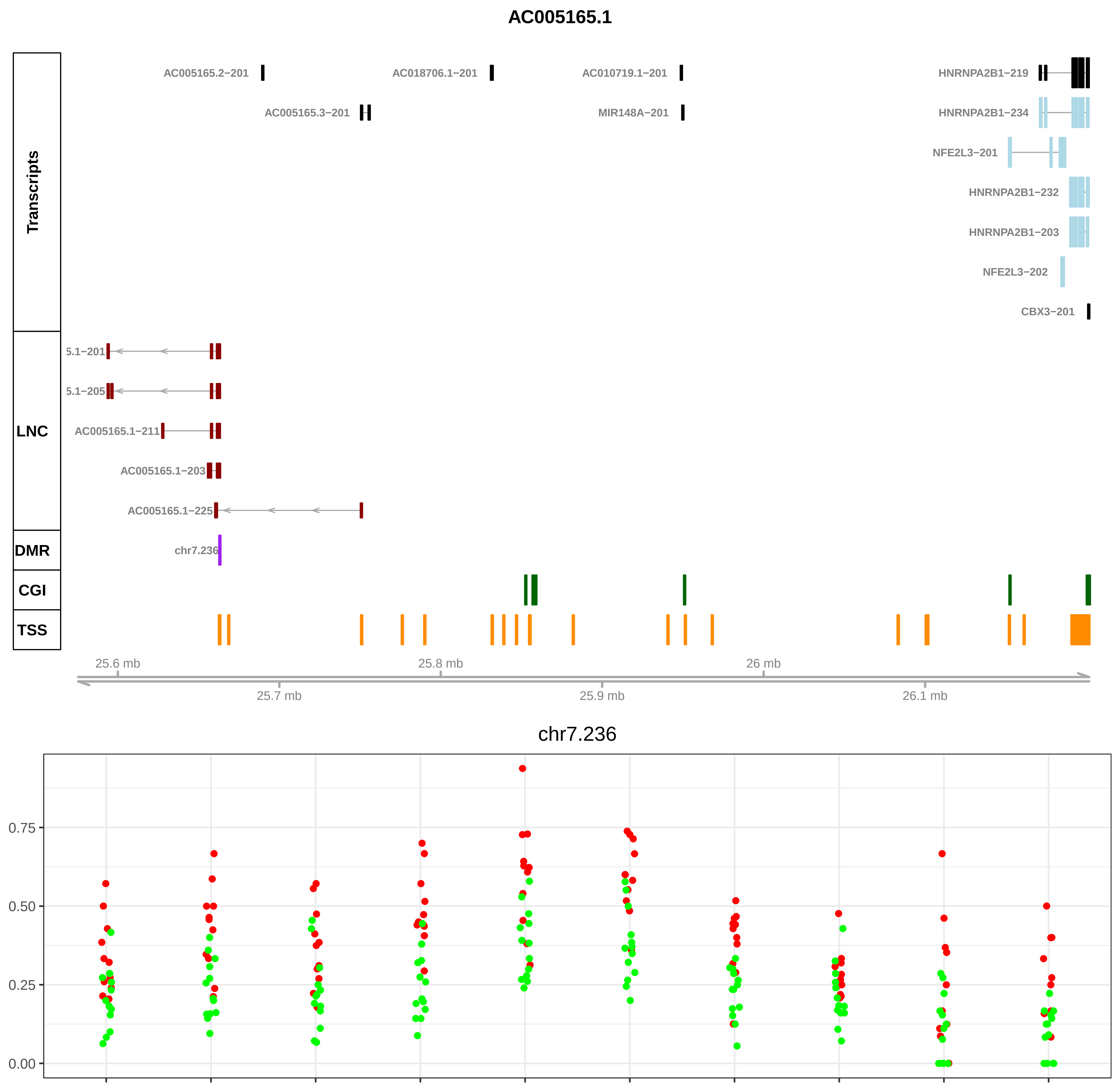
AC005165.1
Transcripts
AC005165.2−201
AC018706.1−201
AC010719.1−201
HNRNPA2B1−219
AC005165.3−201
MIR148A−201
HNRNPA2B1−234
NFE2L3−201
HNRNPA2B1−232
HNRNPA2B1−203
NFE2L3−202
CBX3−201
LNC
5.1−201
5.1−205
AC005165.1−211
AC005165.1−203
AC005165.1−225
DMR
chr7.236
CGI
TSS
25.6 mb
25.8 mb
26 mb
25.7 mb
25.9 mb
26.1 mb
chr7.236
0.75
0.50
0.25
0.00

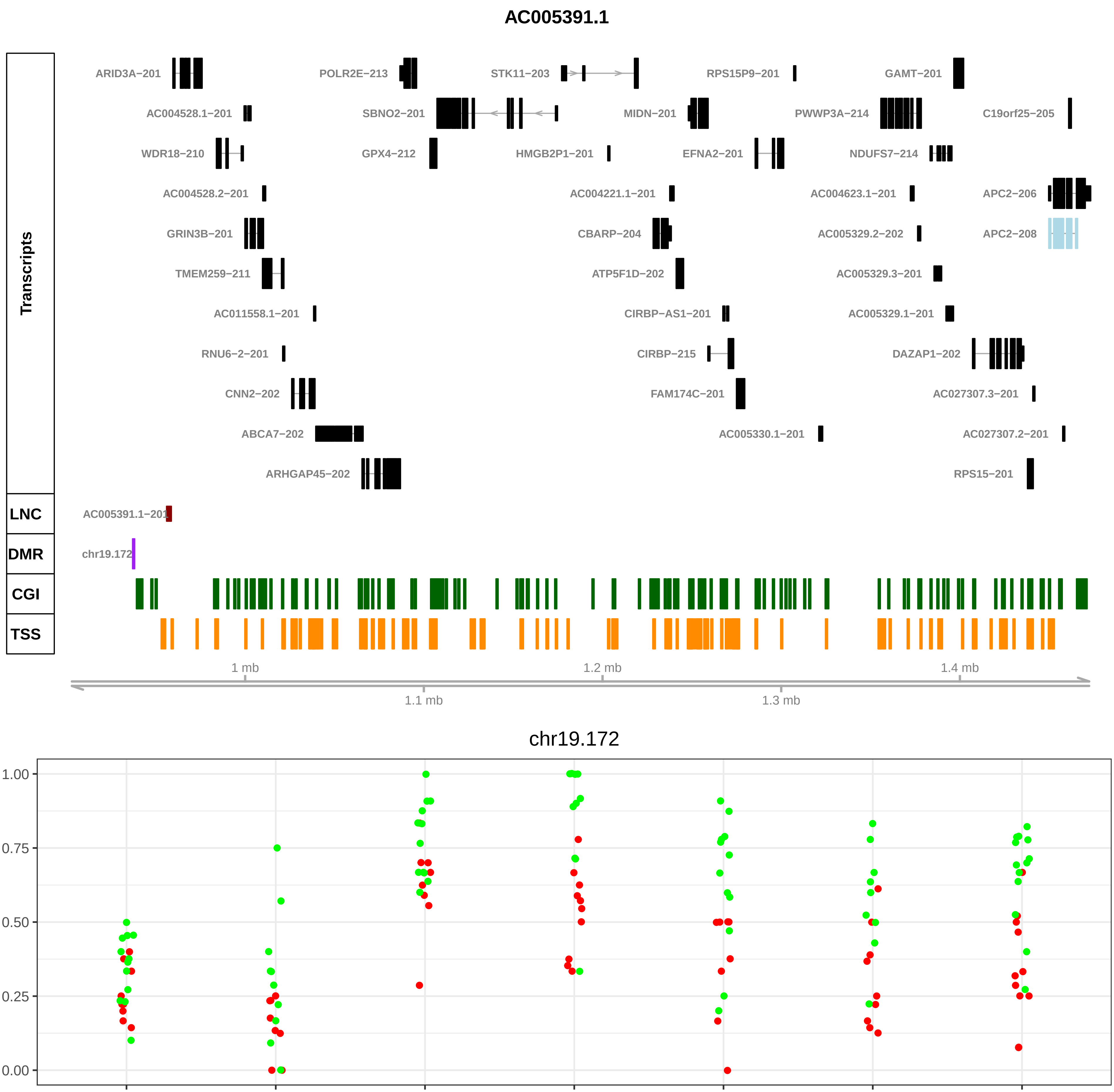
AC005391.1
Transcripts
LNC
DMR
CGI
TSS
ARID3A−201
POLR2E−213
STK11−203
RPS15P9−201
GAMT−201
AC004528.1−201
SBNO2−201
MIDN−201
PWWP3A−214
C19orf25−205
WDR18−210
GPX4−212
HMGB2P1−201
EFNA2−201
NDUFS7−214
AC004528.2−201
AC004221.1−201
AC004623.1−201
APC2−206
GRIN3B−201
CBARP−204
AC005329.2−202
APC2−208
TMEM259−211
ATP5F1D−202
AC005329.3−201
AC011558.1−201
CIRBP−AS1−201
AC005329.1−201
RNU6−2−201
CIRBP−215
DAZAP1−202
CNN2−202
FAM174C−201
AC027307.3−201
ABCA7−202
AC005330.1−201
AC027307.2−201
ARHGAP45−202
RPS15−201
AC005391.1−201
chr19.172
1 mb
1.1 mb
1.2 mb
1.3 mb
1.4 mb
chr19.172
1.00
0.75
0.50
0.25
0.00

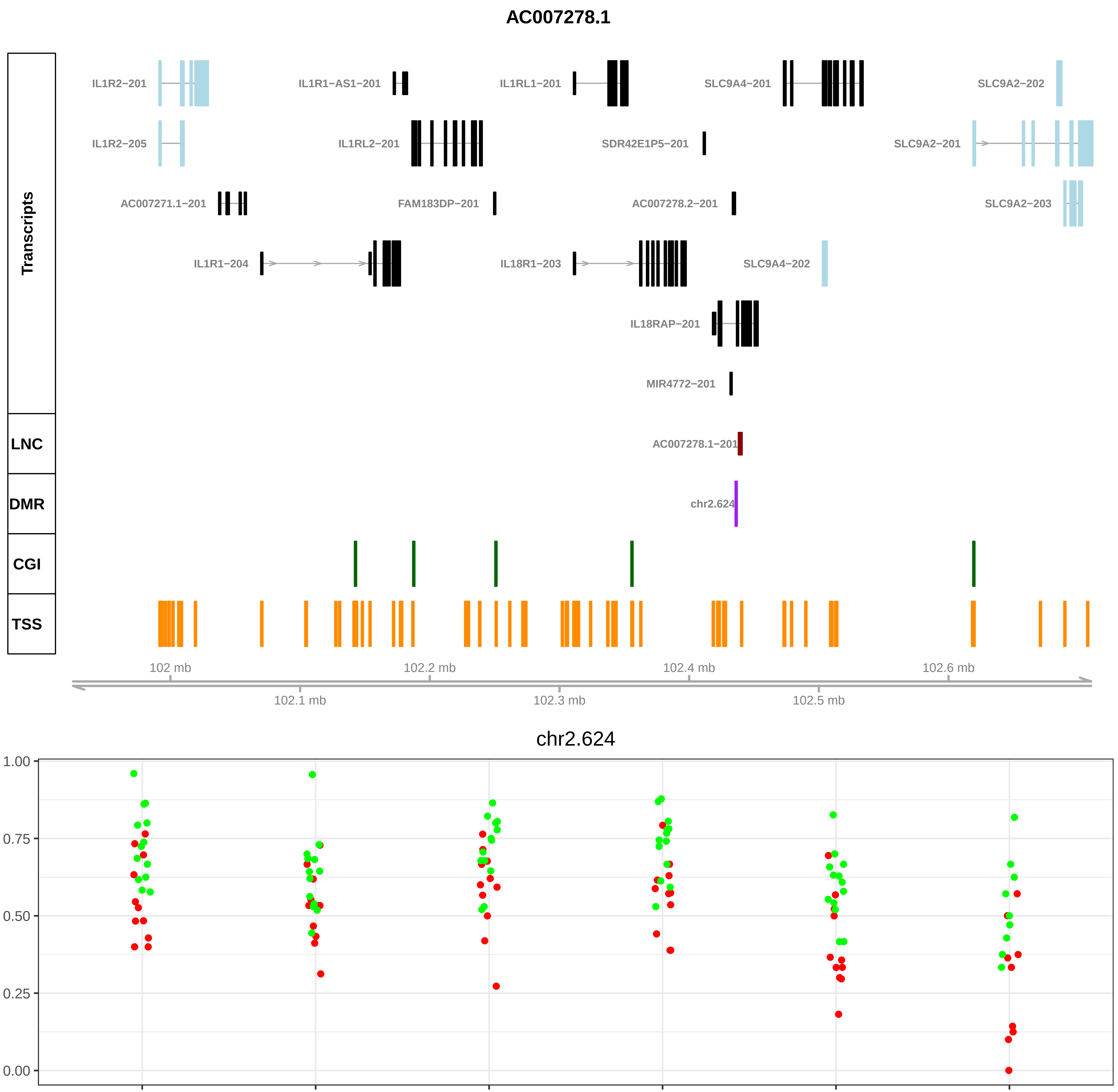

AC007278.1
Transcripts
LNC
DMR
CGI
TSS
IL1R2−201
IL1R1−AS1−201
IL1RL1−201
SLC9A4−201
SLC9A2−202
IL1R2−205
IL1RL2−201
SDR42E1P5−201
SLC9A2−201
AC007271.1−201
FAM183DP−201
AC007278.2−201
SLC9A2−203
IL1R1−204
IL18R1−203
SLC9A4−202
IL18RAP−201
MIR4772−201
AC007278.1−201
chr2.624
102 mb
102.2 mb
102.4 mb
102.6 mb
102.1 mb
102.3 mb
102.5 mb
chr2.624
1.00
0.75
0.50
0.25
0.00

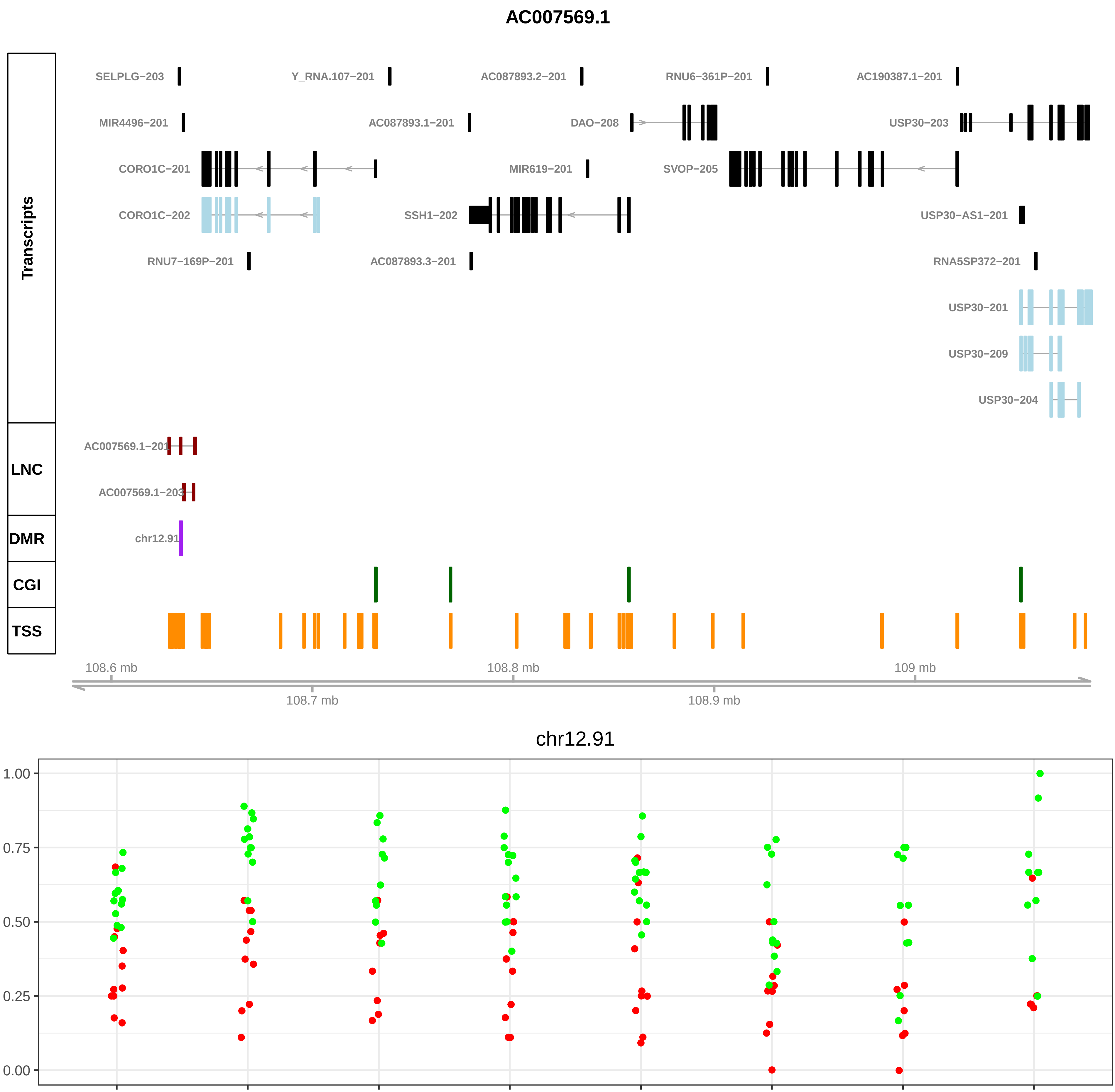

AC007569.1
Transcripts
SELPLG−203
Y_RNA.107−201
AC087893.2−201
RNU6−361P−201
AC190387.1−201
MIR4496−201
AC087893.1−201
DAO−208
USP30−203
CORO1C−201
MIR619−201
SVOP−205
CORO1C−202
SSH1−202
USP30−AS1−201
RNU7−169P−201
AC087893.3−201
RNA5SP372−201
USP30−201
USP30−209
USP30−204
LNC
AC007569.1−201
AC007569.1−203
DMR
chr12.91
CGI
TSS
108.6 mb
108.8 mb
109 mb
108.7 mb
108.9 mb
chr12.91
1.00
0.75
0.50
0.25
0.00

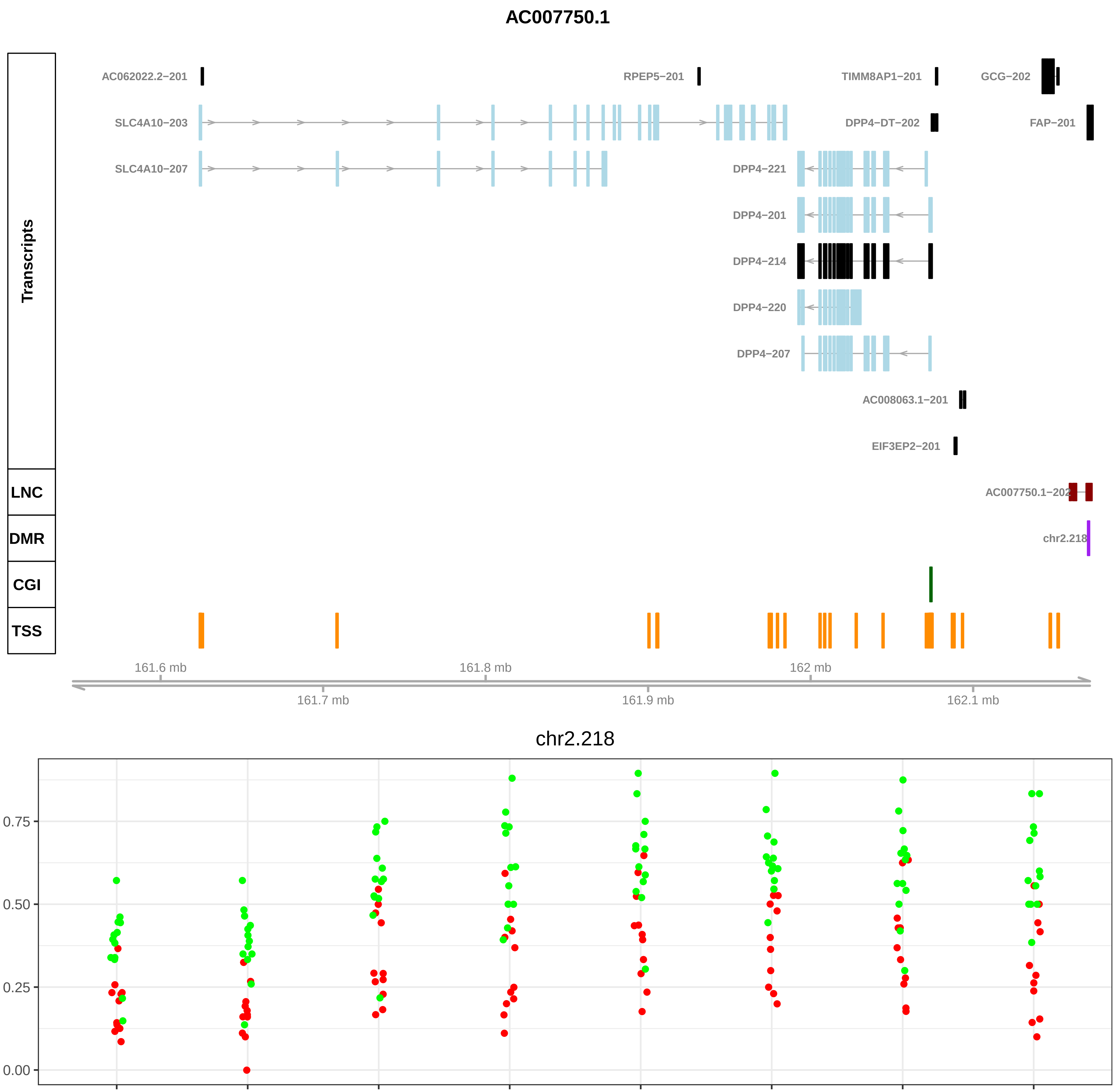
AC007750.1
Transcripts
AC062022.2−201
RPEP5−201
TIMM8AP1−201
GCG−202
SLC4A10−203
DPP4−DT−202
FAP−201
SLC4A10−207
DPP4−221
DPP4−201
DPP4−214
DPP4−220
DPP4−207
AC008063.1−201
EIF3EP2−201
LNC
AC007750.1−202
DMR
chr2.218
CGI
TSS
161.6 mb
161.8 mb
162 mb
161.7 mb
161.9 mb
162.1 mb
chr2.218
0.75
0.50
0.25
0.00

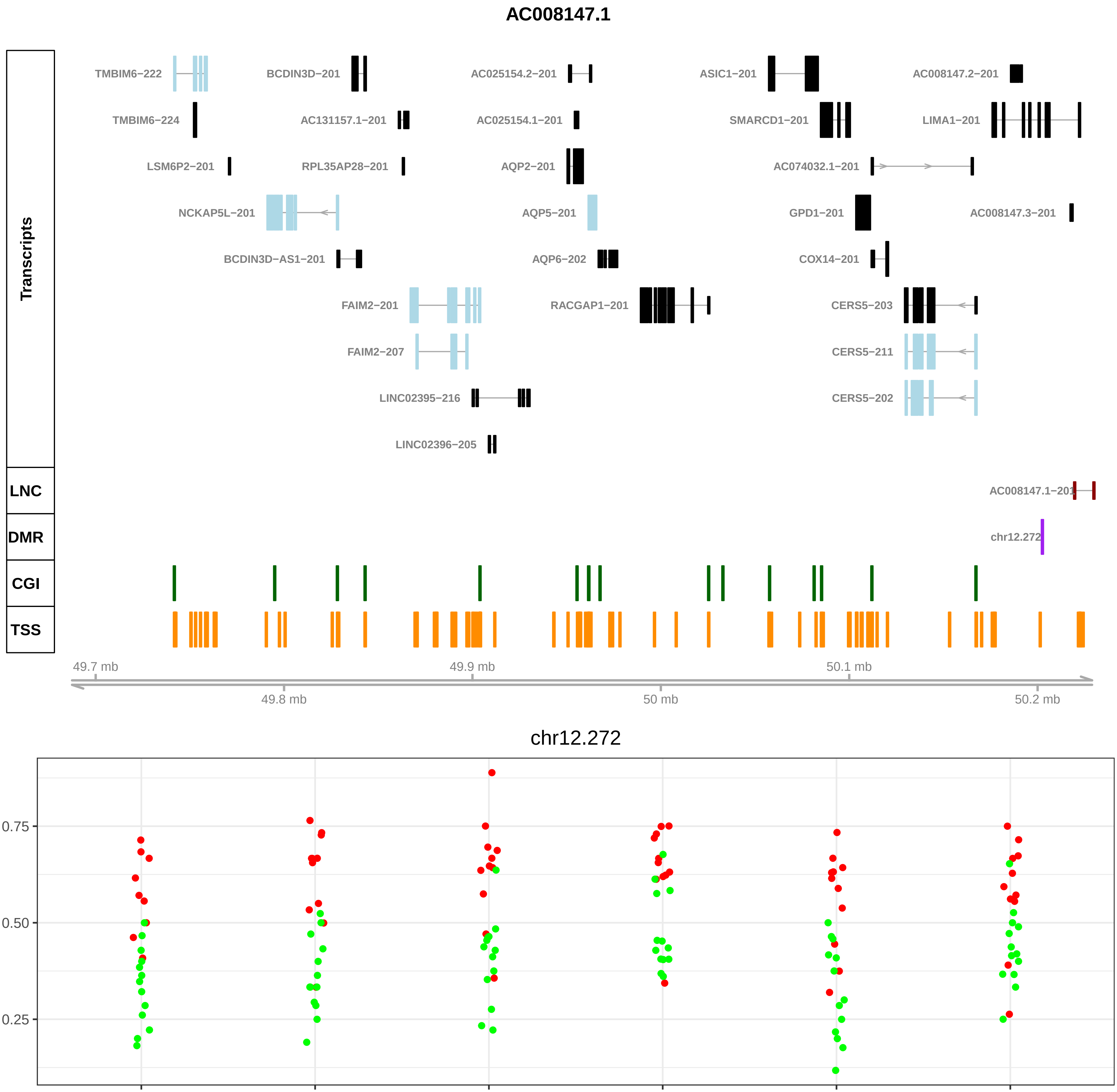
AC008147.1
Transcripts
TMBIM6−222
BCDIN3D−201
AC025154.2−201
ASIC1−201
AC008147.2−201
TMBIM6−224
AC131157.1−201
AC025154.1−201
SMARCD1−201
LIMA1−201
LSM6P2−201
RPL35AP28−201
AQP2−201
AC074032.1−201
NCKAP5L−201
AQP5−201
GPD1−201
AC008147.3−201
BCDIN3D−AS1−201
AQP6−202
COX14−201
FAIM2−201
RACGAP1−201
CERS5−203
FAIM2−207
CERS5−211
LINC02395−216
CERS5−202
LINC02396−205
LNC
AC008147.1−201
DMR
chr12.272
CGI
TSS
49.7 mb
49.9 mb
50.1 mb
49.8 mb
50 mb
50.2 mb
chr12.272
0.75
0.50
0.25

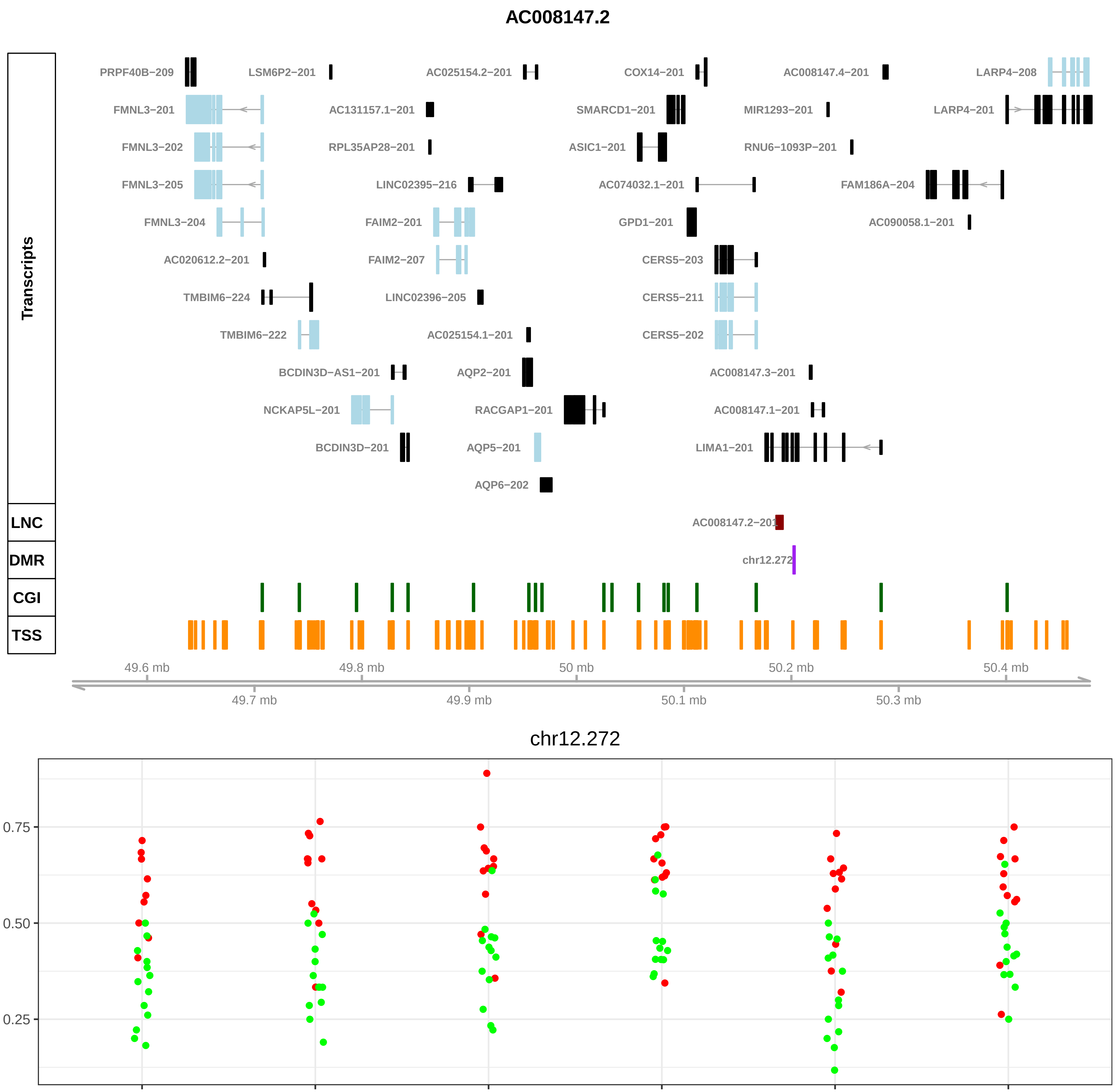

AC008147.2
Transcripts
PRPF40B−209
LSM6P2−201
AC025154.2−201
COX14−201
AC008147.4−201
LARP4−208
FMNL3−201
AC131157.1−201
SMARCD1−201
MIR1293−201
LARP4−201
FMNL3−202
RPL35AP28−201
ASIC1−201
RNU6−1093P−201
FMNL3−205
LINC02395−216
AC074032.1−201
FAM186A−204
FMNL3−204
FAIM2−201
GPD1−201
AC090058.1−201
AC020612.2−201
FAIM2−207
CERS5−203
TMBIM6−224
LINC02396−205
CERS5−211
TMBIM6−222
AC025154.1−201
CERS5−202
BCDIN3D−AS1−201
AQP2−201
AC008147.3−201
NCKAP5L−201
RACGAP1−201
AC008147.1−201
BCDIN3D−201
AQP5−201
LIMA1−201
AQP6−202
LNC
AC008147.2−201
DMR
chr12.272
CGI
TSS
49.6 mb
49.8 mb
50 mb
50.2 mb
50.4 mb
49.7 mb
49.9 mb
50.1 mb
50.3 mb
chr12.272
0.75
0.50
0.25

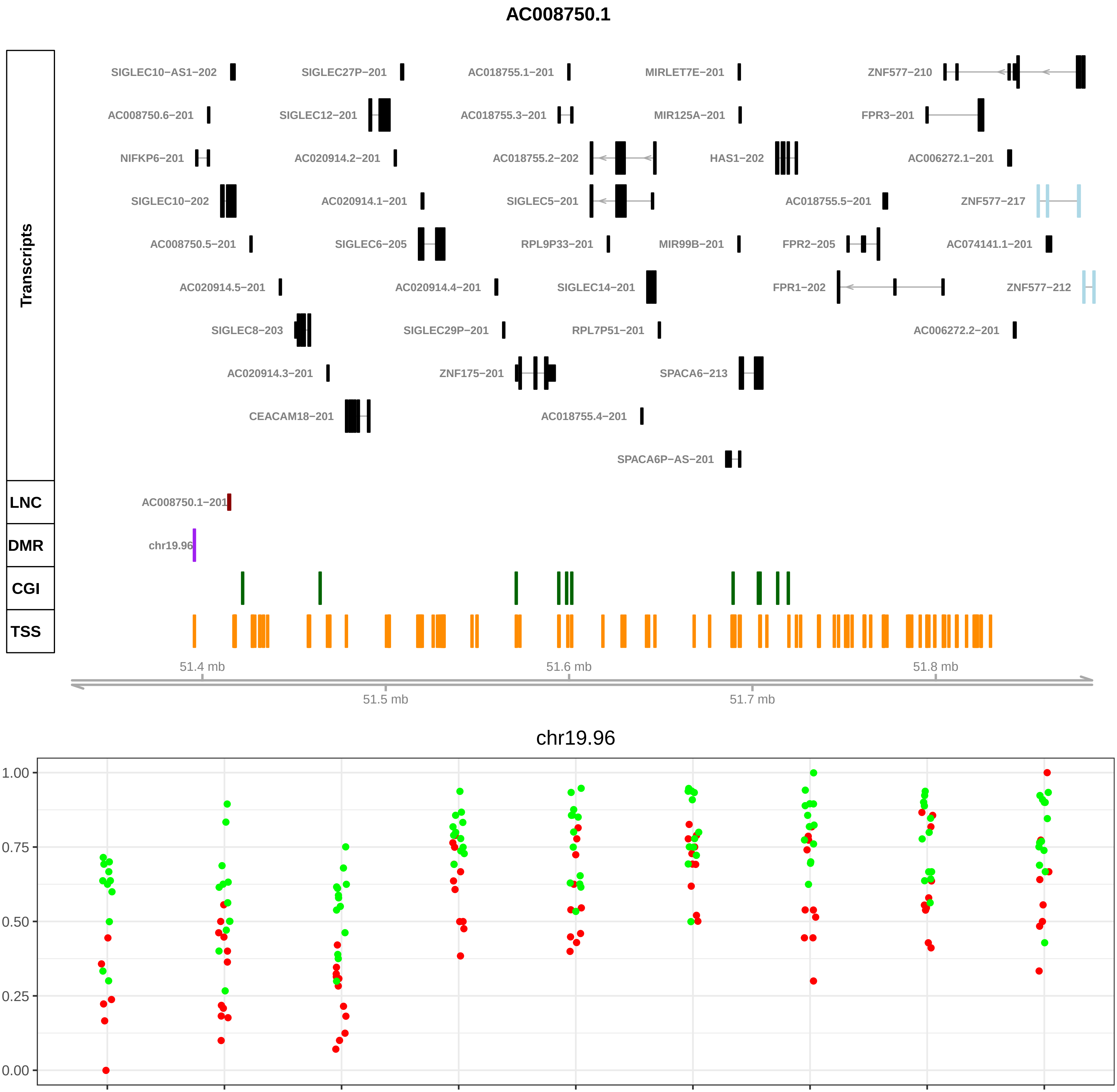

AC008750.1
Transcripts
SIGLEC10−AS1−202
SIGLEC27P−201
AC018755.1−201
MIRLET7E−201
ZNF577−210
AC008750.6−201
SIGLEC12−201
AC018755.3−201
MIR125A−201
FPR3−201
NIFKP6−201
AC020914.2−201
AC018755.2−202
HAS1−202
AC006272.1−201
SIGLEC10−202
AC020914.1−201
SIGLEC5−201
AC018755.5−201
ZNF577−217
AC008750.5−201
SIGLEC6−205
RPL9P33−201
MIR99B−201
FPR2−205
AC074141.1−201
AC020914.5−201
AC020914.4−201
SIGLEC14−201
FPR1−202
ZNF577−212
SIGLEC8−203
SIGLEC29P−201
RPL7P51−201
AC006272.2−201
AC020914.3−201
ZNF175−201
SPACA6−213
CEACAM18−201
AC018755.4−201
SPACA6P−AS−201
LNC
AC008750.1−201
DMR
chr19.96
CGI
TSS
51.4 mb
51.6 mb
51.8 mb
51.5 mb
51.7 mb
chr19.96
1.00
0.75
0.50
0.25
0.00

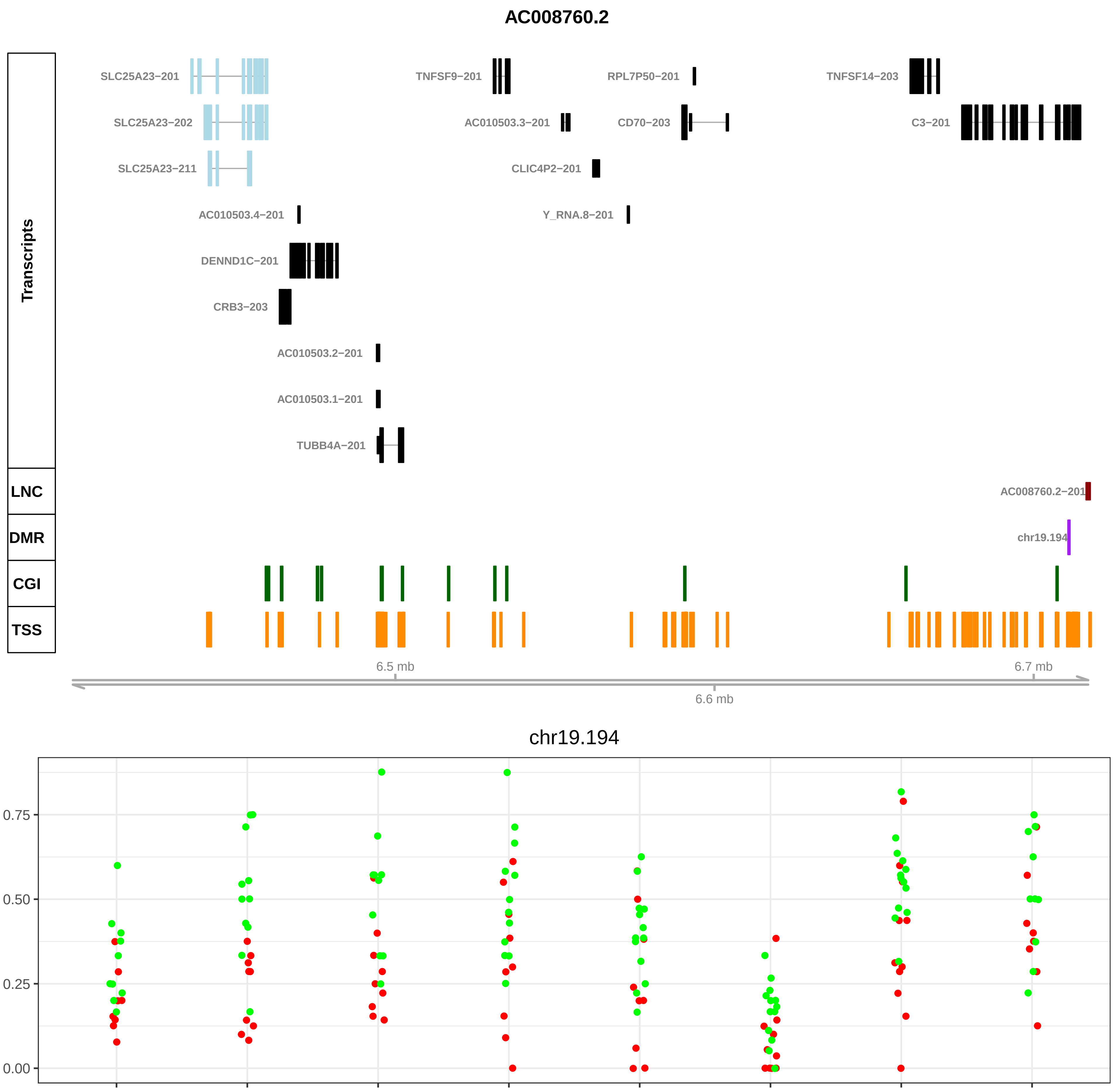

AC008760.2
Transcripts
SLC25A23−201
SLC25A23−202
SLC25A23−211
AC010503.4−201
DENND1C−201
CRB3−203
AC010503.2−201
AC010503.1−201
TUBB4A−201
TNFSF9−201
AC010503.3−201
CLIC4P2−201
Y_RNA.8−201
RPL7P50−201
CD70−203
TNFSF14−203
C3−201
LNC
AC008760.2−201
DMR
chr19.194
CGI
TSS
6.5 mb
6.6 mb
6.7 mb
chr19.194
0.75
0.50
0.25
0.00

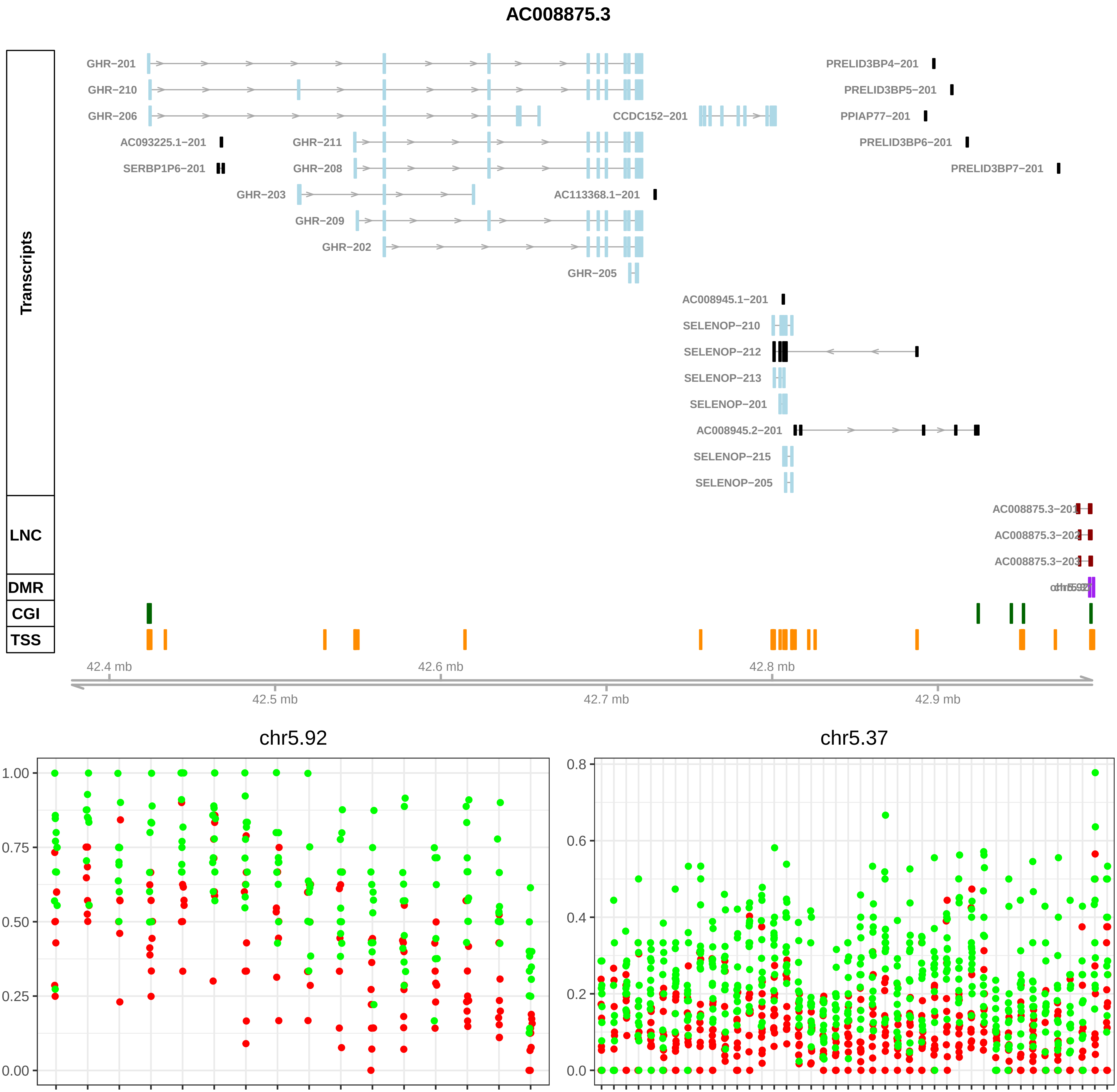
AC008875.3
Transcripts
GHR−201
GHR−210
GHR−206
AC093225.1−201
SERBP1P6−201
GHR−211
GHR−208
GHR−203
GHR−209
GHR−202
GHR−205
CCDC152−201
AC113368.1−201
PRELID3BP4−201
PRELID3BP5−201
PPIAP77−201
PRELID3BP6−201
PRELID3BP7−201
AC008945.1−201
SELENOP−210
SELENOP−212
SELENOP−213
SELENOP−201
AC008945.2−201
SELENOP−215
SELENOP−205
LNC
AC008875.3−201
AC008875.3−202
AC008875.3−203
DMR
CGI
TSS
42.4 mb
42.6 mb
42.8 mb
42.5 mb
42.7 mb
42.9 mb
chr5.92
1.00
0.75
0.50
0.25
0.00
chr5.37
0.8
0.6
0.4
0.2
0.0

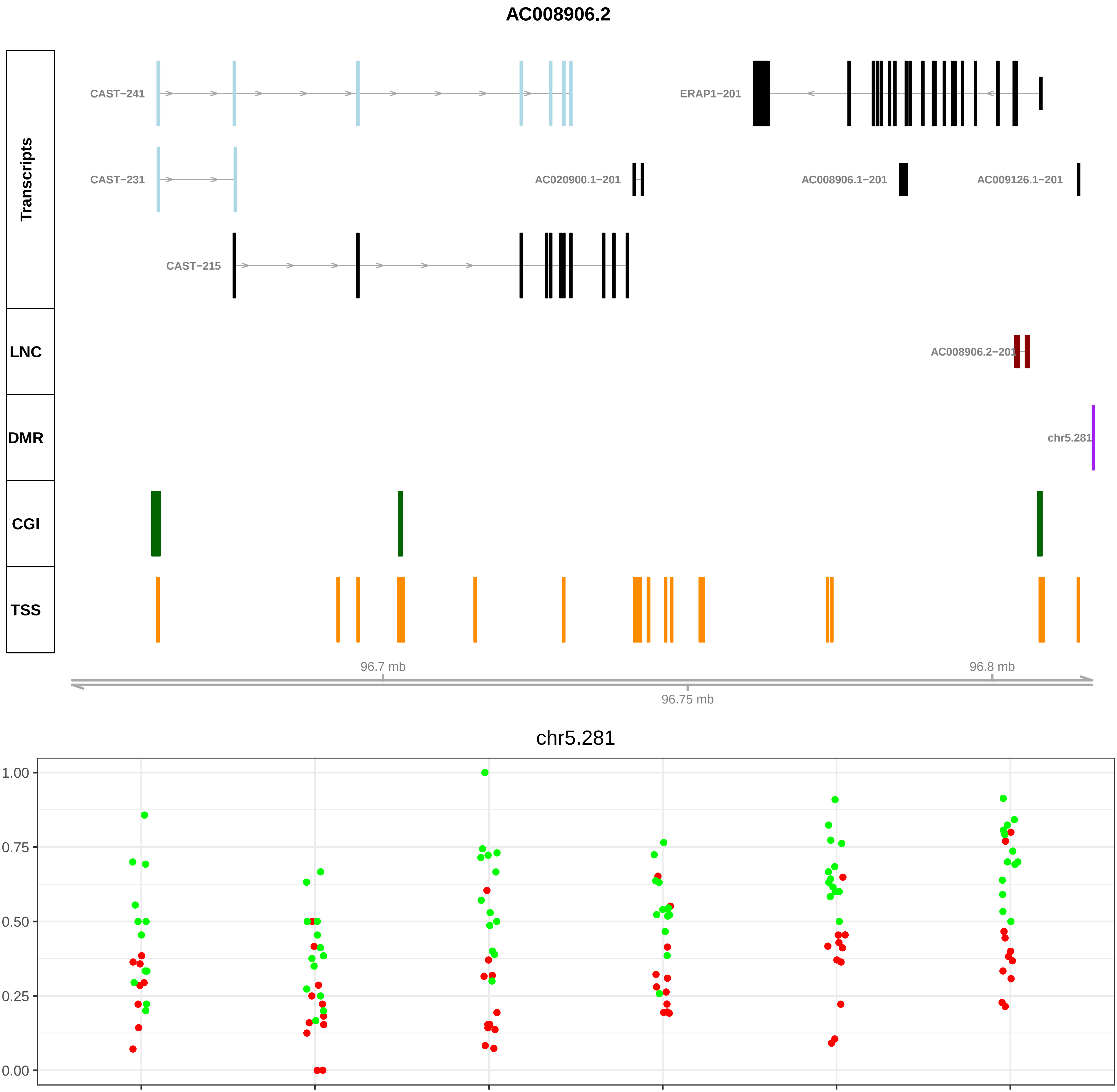
AC008906.2
Transcripts
CAST−241
ERAP1−201
CAST−231
AC020900.1−201
AC008906.1−201
AC009126.1−201
CAST−215
LNC
AC008906.2−201
DMR
chr5.281
CGI
TSS
96.7 mb
96.8 mb
96.75 mb
chr5.281
1.00
0.75
0.50
0.25
0.00

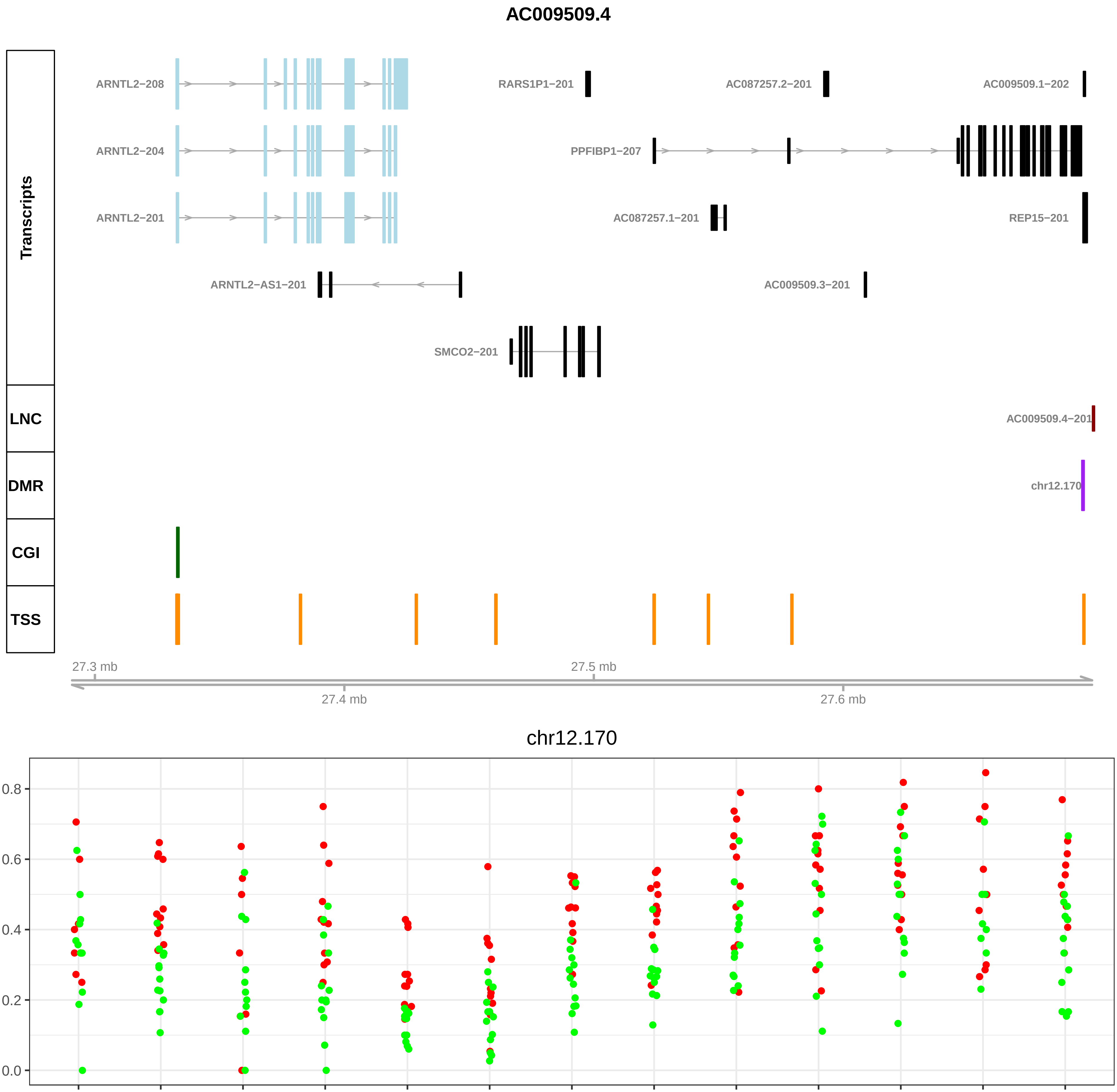

AC009509.4
Transcripts
ARNTL2−208
ARNTL2−204
ARNTL2−201
ARNTL2−AS1−201
SMCO2−201
RARS1P1−201
PPFIBP1−207
AC087257.1−201
AC087257.2−201
AC009509.3−201
AC009509.1−202
REP15−201
LNC
AC009509.4−201
DMR
chr12.170
CGI
TSS
27.3 mb
27.4 mb
27.5 mb
27.6 mb
chr12.170
0.0
0.2
0.4
0.6
0.8

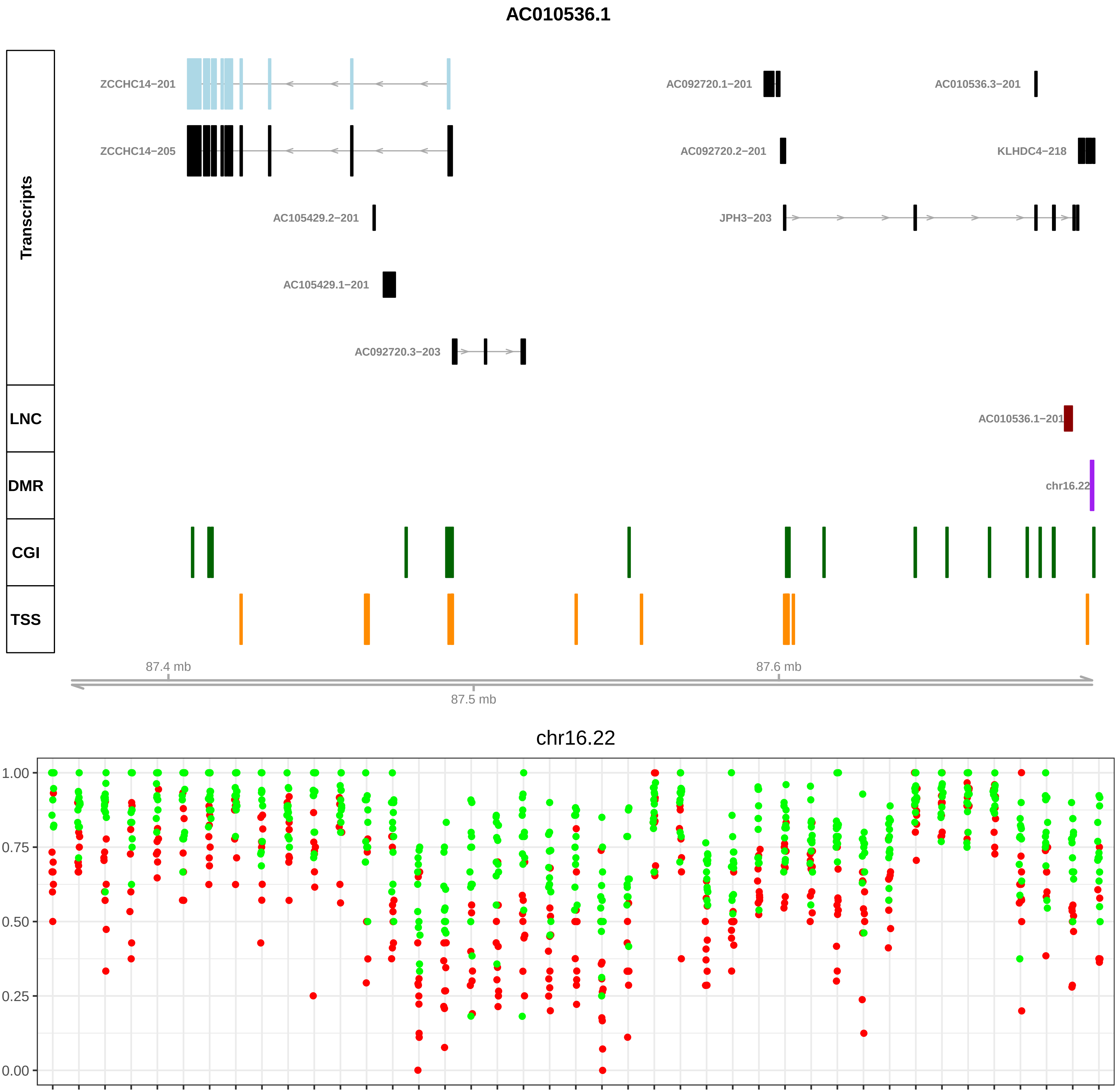

AC010536.1
Transcripts
ZCCHC14−201
ZCCHC14−205
AC105429.2−201
AC105429.1−201
AC092720.3−203
AC092720.1−201
AC092720.2−201
JPH3−203
AC010536.3−201
KLHDC4−218
LNC
AC010536.1−201
DMR
chr16.22
CGI
TSS
87.4 mb
87.5 mb
87.6 mb
chr16.22
1.00
0.75
0.50
0.25
0.00

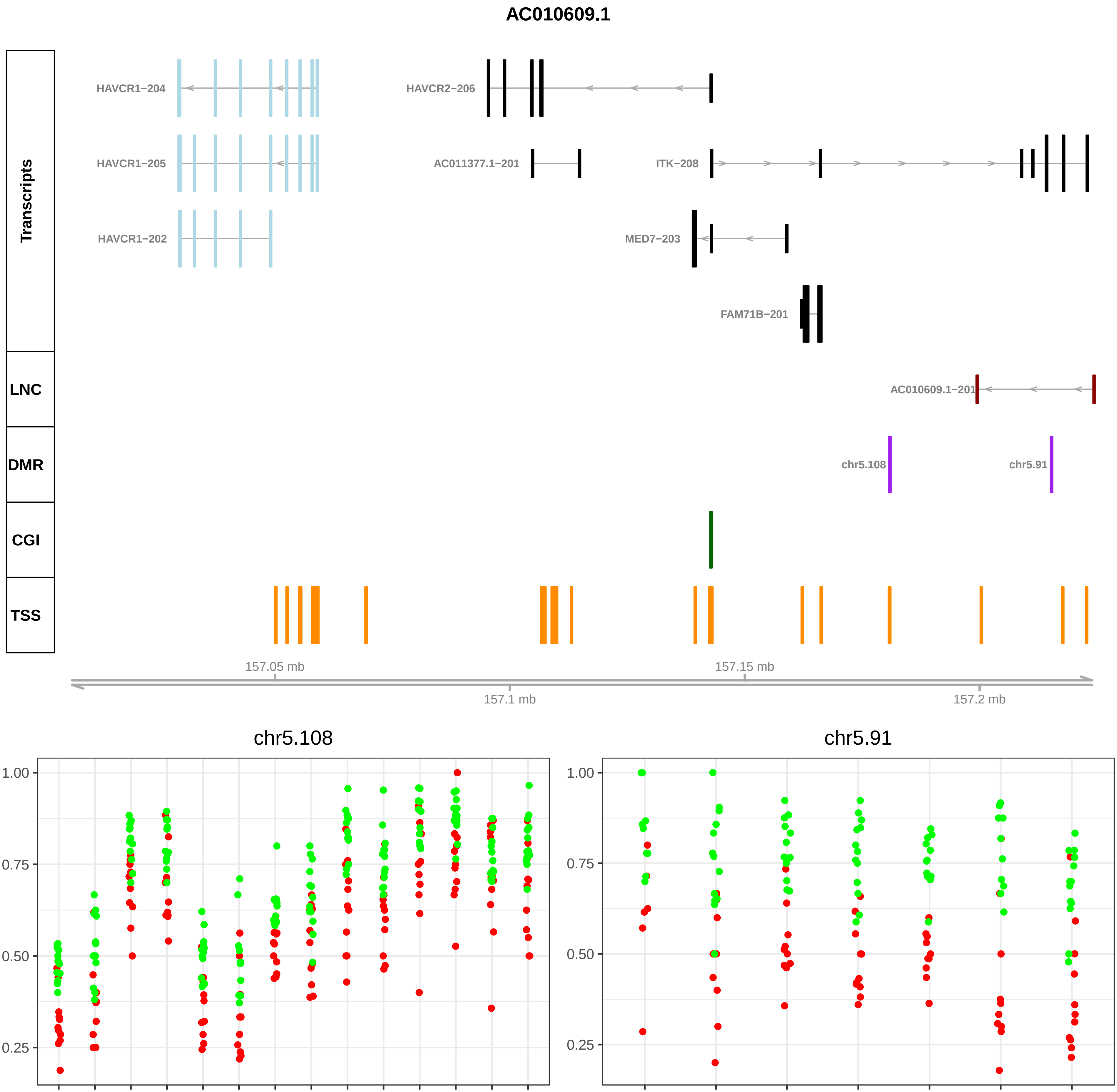
AC010609.1
Transcripts
HAVCR1−204
HAVCR2−206
HAVCR1−205
AC011377.1−201
ITK−208
HAVCR1−202
MED7−203
FAM71B−201
LNC
AC010609.1−201
DMR
chr5.108
chr5.91
CGI
TSS
157.05 mb
157.15 mb
157.1 mb
157.2 mb
chr5.108
chr5.91
1.00
0.75
0.50
0.25

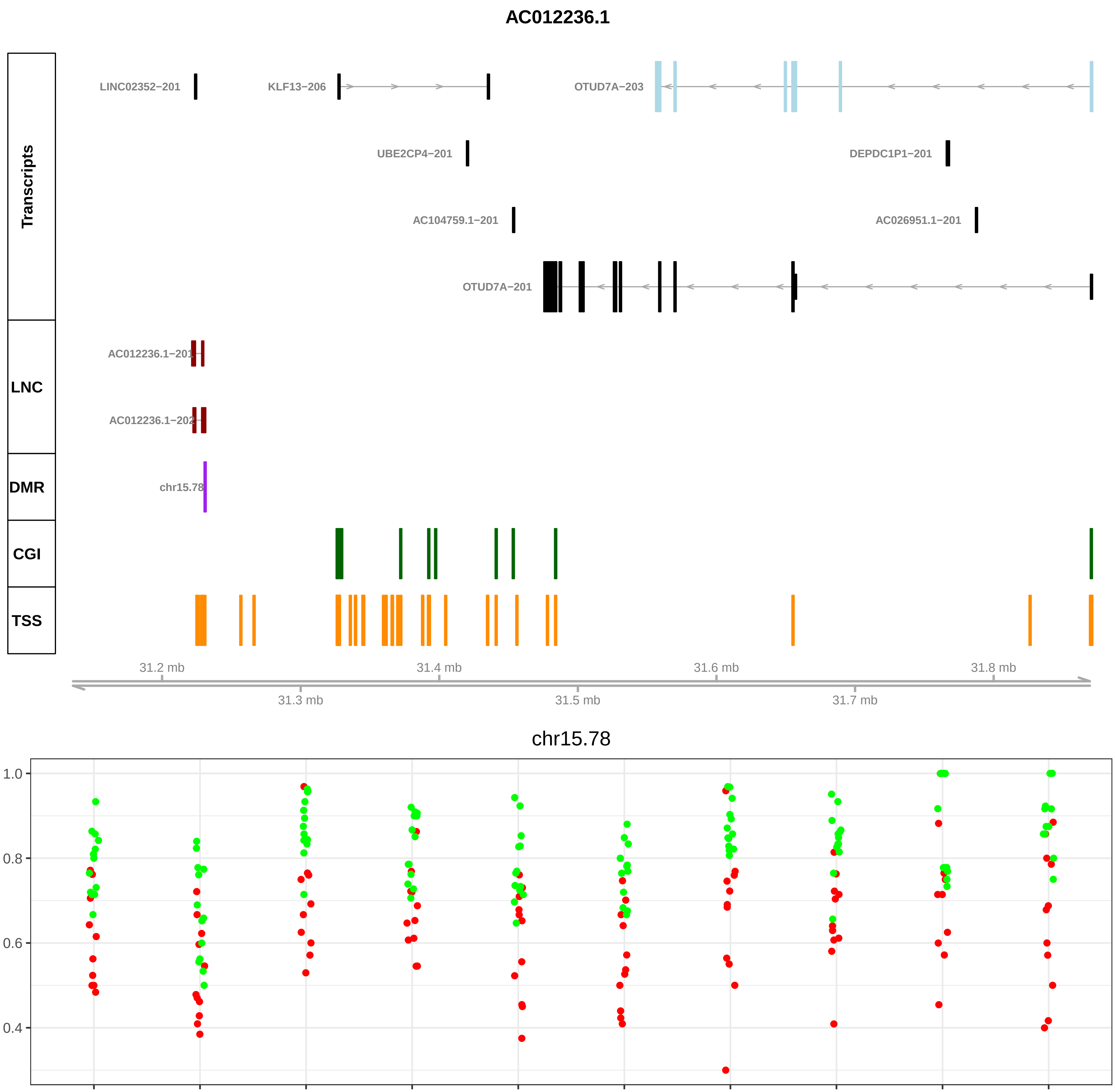
AC012236.1
Transcripts
LINC02352−201
KLF13−206
OTUD7A−203
UBE2CP4−201
DEPDC1P1−201
AC104759.1−201
AC026951.1−201
OTUD7A−201
LNC
AC012236.1−201
AC012236.1−202
DMR
chr15.78
CGI
TSS
31.2 mb
31.4 mb
31.6 mb
31.8 mb
31.3 mb
31.5 mb
31.7 mb
chr15.78
1.0
0.8
0.6
0.4

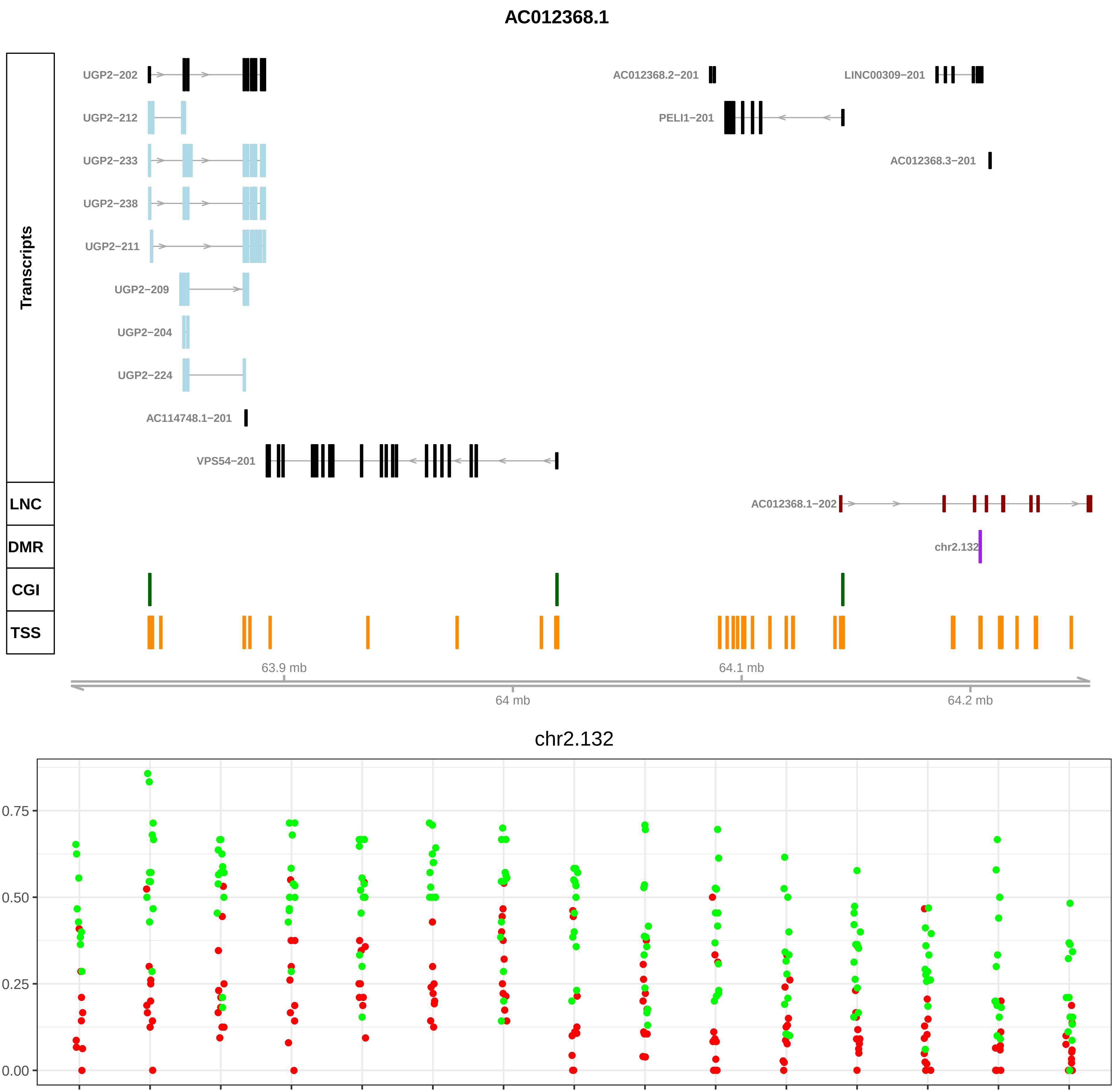
AC012368.1
Transcripts
UGP2−202
UGP2−212
UGP2−233
UGP2−238
UGP2−211
UGP2−209
UGP2−204
UGP2−224
AC114748.1−201
VPS54−201
AC012368.2−201
PELI1−201
LINC00309−201
AC012368.3−201
LNC
AC012368.1−202
DMR
chr2.132
CGI
TSS
63.9 mb
64 mb
64.1 mb
64.2 mb
chr2.132
0.75
0.50
0.25
0.00

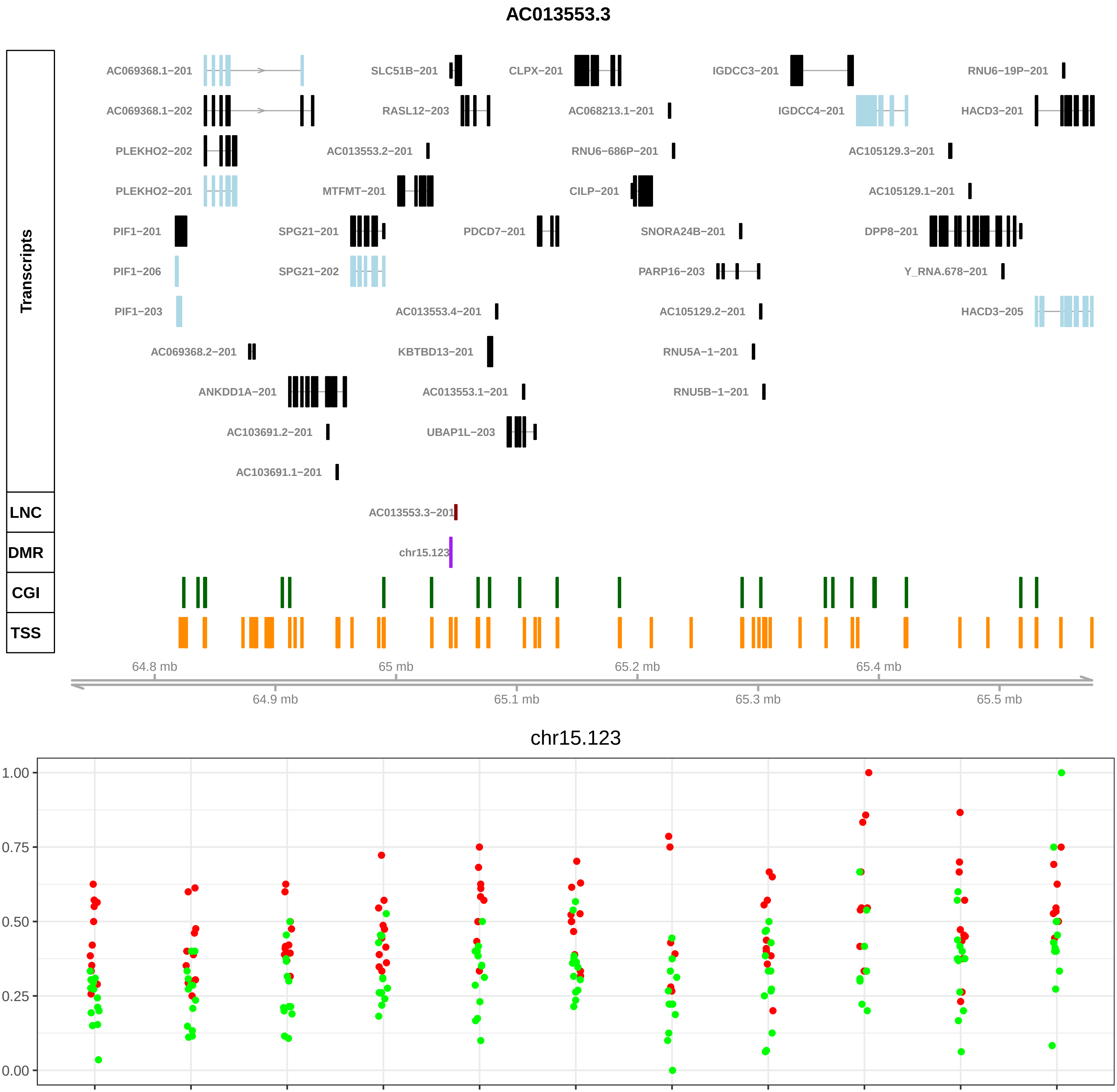

AC013553.3
Transcripts
AC069368.1−201
AC069368.1−202
PLEKHO2−202
PLEKHO2−201
PIF1−201
PIF1−206
PIF1−203
AC069368.2−201
ANKDD1A−201
AC103691.2−201
AC103691.1−201
SLC51B−201
RASL12−203
AC013553.2−201
MTFMT−201
SPG21−201
SPG21−202
AC013553.4−201
KBTBD13−201
AC013553.1−201
UBAP1L−203
CLPX−201
AC068213.1−201
RNU6−686P−201
CILP−201
PDCD7−201
SNORA24B−201
PARP16−203
AC105129.2−201
RNU5A−1−201
RNU5B−1−201
IGDCC3−201
IGDCC4−201
AC105129.3−201
AC105129.1−201
DPP8−201
Y_RNA.678−201
RNU6−19P−201
HACD3−201
HACD3−205
LNC
AC013553.3−201
DMR
chr15.123
CGI
TSS
64.8 mb
65 mb
65.2 mb
65.4 mb
64.9 mb
65.1 mb
65.3 mb
65.5 mb
chr15.123
1.00
0.75
0.50
0.25
0.00

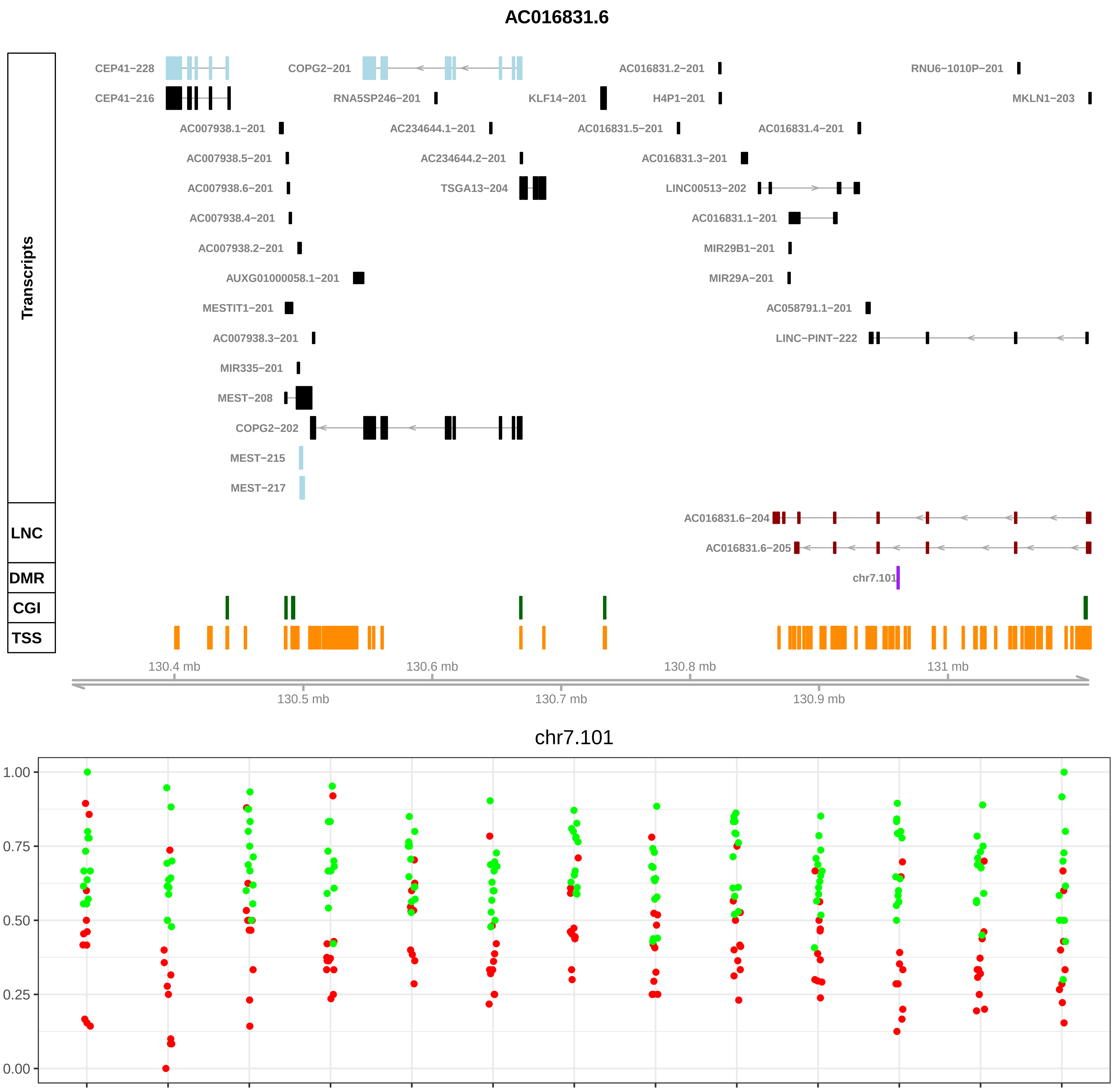

AC016831.6
Transcripts
LNC
DMR
CGI
TSS
CEP41−228
CEP41−216
COPG2−201
RNA5SP246−201
KLF14−201
AC016831.2−201
H4P1−201
RNU6−1010P−201
MKLN1−203
AC007938.1−201
AC234644.1−201
AC016831.5−201
AC016831.4−201
AC007938.5−201
AC234644.2−201
AC016831.3−201
AC007938.6−201
TSGA13−204
LINC00513−202
AC007938.4−201
AC016831.1−201
AC007938.2−201
MIR29B1−201
AUXG01000058.1−201
MIR29A−201
MESTIT1−201
AC058791.1−201
AC007938.3−201
LINC−PINT−222
MIR335−201
MEST−208
COPG2−202
MEST−215
MEST−217
AC016831.6−204
AC016831.6−205
chr7.101
130.4 mb
130.6 mb
130.8 mb
131 mb
130.5 mb
130.7 mb
130.9 mb
chr7.101
1.00
0.75
0.50
0.25
0.00

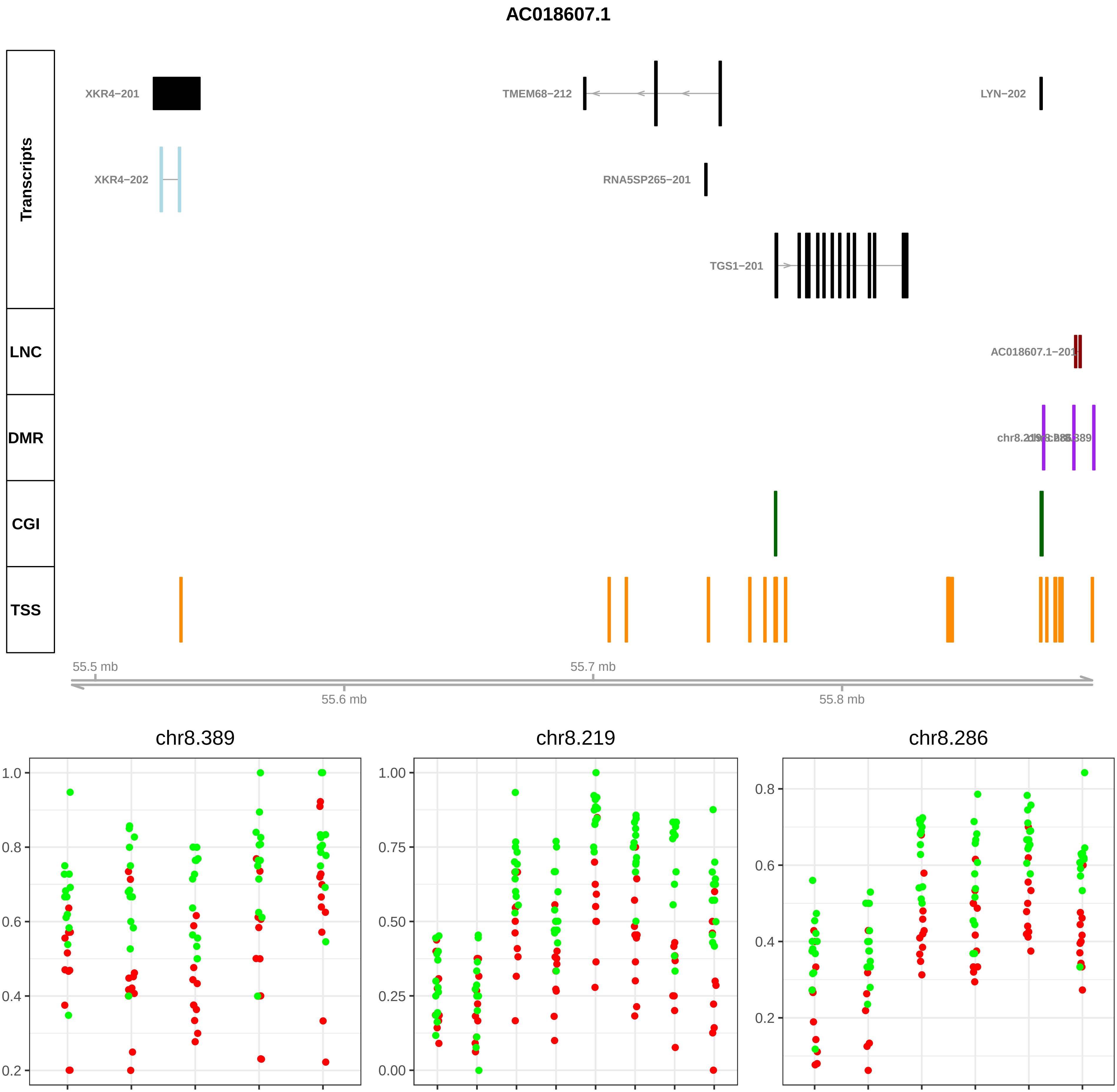
AC018607.1
Transcripts
LNC
DMR
CGI
TSS
XKR4−201
TMEM68−212
LYN−202
XKR4−202
RNA5SP265−201
TGS1−201
AC018607.1−201
55.5 mb
55.6 mb
55.7 mb
55.8 mb
chr8.389
chr8.219
chr8.286

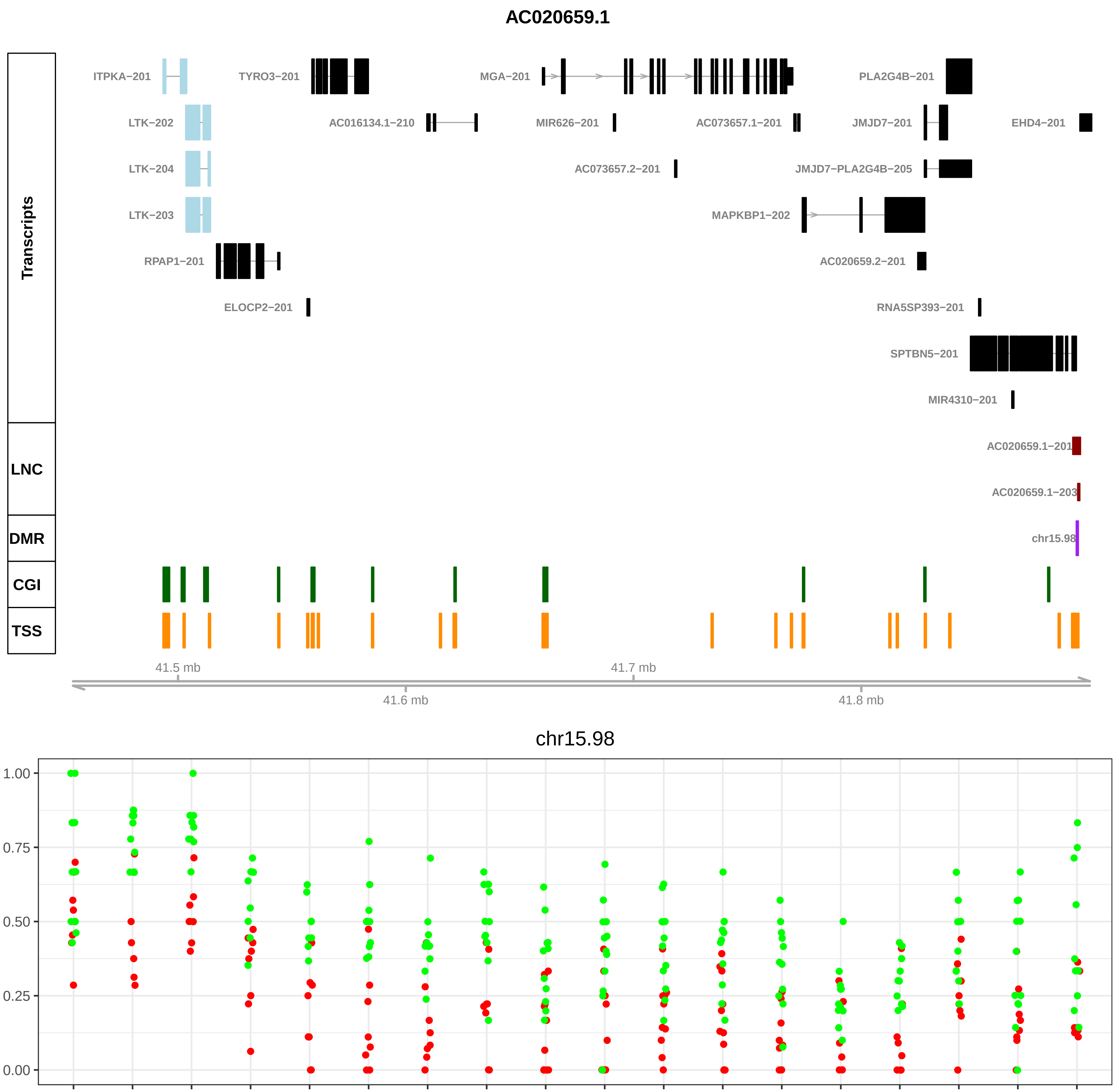

AC020659.1
Transcripts
LNC
DMR
CGI
TSS
ITPKA−201
TYRO3−201
MGA−201
PLA2G4B−201
LTK−202
AC016134.1−210
MIR626−201
AC073657.1−201
JMJD7−201
EHD4−201
LTK−204
AC073657.2−201
JMJD7−PLA2G4B−205
LTK−203
MAPKBP1−202
RPAP1−201
AC020659.2−201
ELOCP2−201
RNA5SP393−201
SPTBN5−201
MIR4310−201
AC020659.1−201
AC020659.1−203
chr15.98
41.5 mb
41.6 mb
41.7 mb
41.8 mb
chr15.98
1.00
0.75
0.50
0.25
0.00

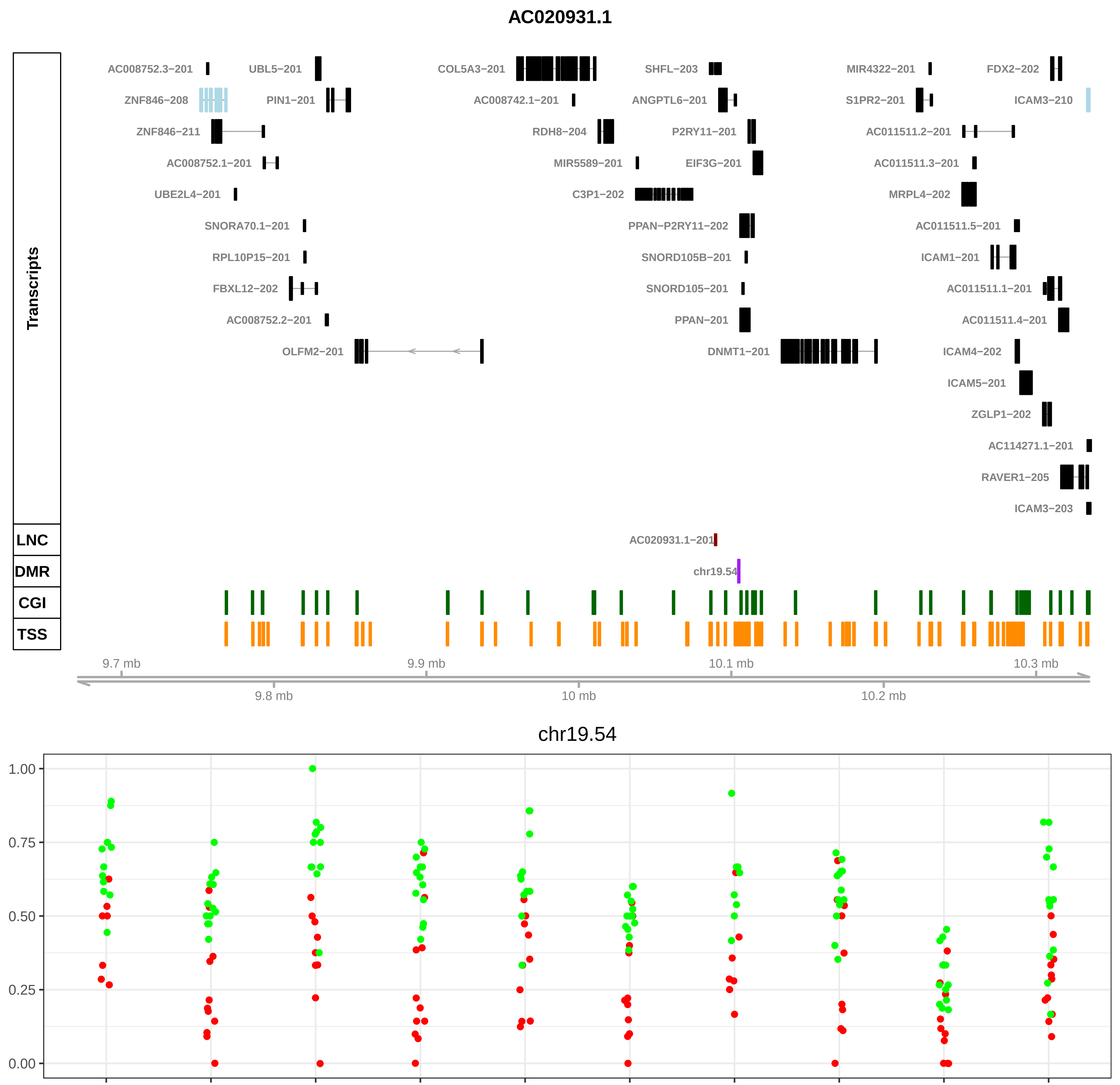

AC020931.1
Transcripts
LNC
DMR
CGI
TSS
AC008752.3−201
UBL5−201
COL5A3−201
SHFL−203
MIR4322−201
FDX2−202
ZNF846−208
PIN1−201
AC008742.1−201
ANGPTL6−201
S1PR2−201
ICAM3−210
ZNF846−211
RDH8−204
P2RY11−201
AC011511.2−201
AC008752.1−201
MIR5589−201
EIF3G−201
AC011511.3−201
UBE2L4−201
C3P1−202
MRPL4−202
SNORA70.1−201
PPAN−P2RY11−202
AC011511.5−201
RPL10P15−201
SNORD105B−201
ICAM1−201
FBXL12−202
SNORD105−201
AC011511.1−201
AC008752.2−201
PPAN−201
AC011511.4−201
OLFM2−201
DNMT1−201
ICAM4−202
ICAM5−201
ZGLP1−202
AC114271.1−201
RAVER1−205
ICAM3−203
AC020931.1−201
chr19.54
9.7 mb
9.8 mb
9.9 mb
10 mb
10.1 mb
10.2 mb
10.3 mb
chr19.54
1.00
0.75
0.50
0.25
0.00

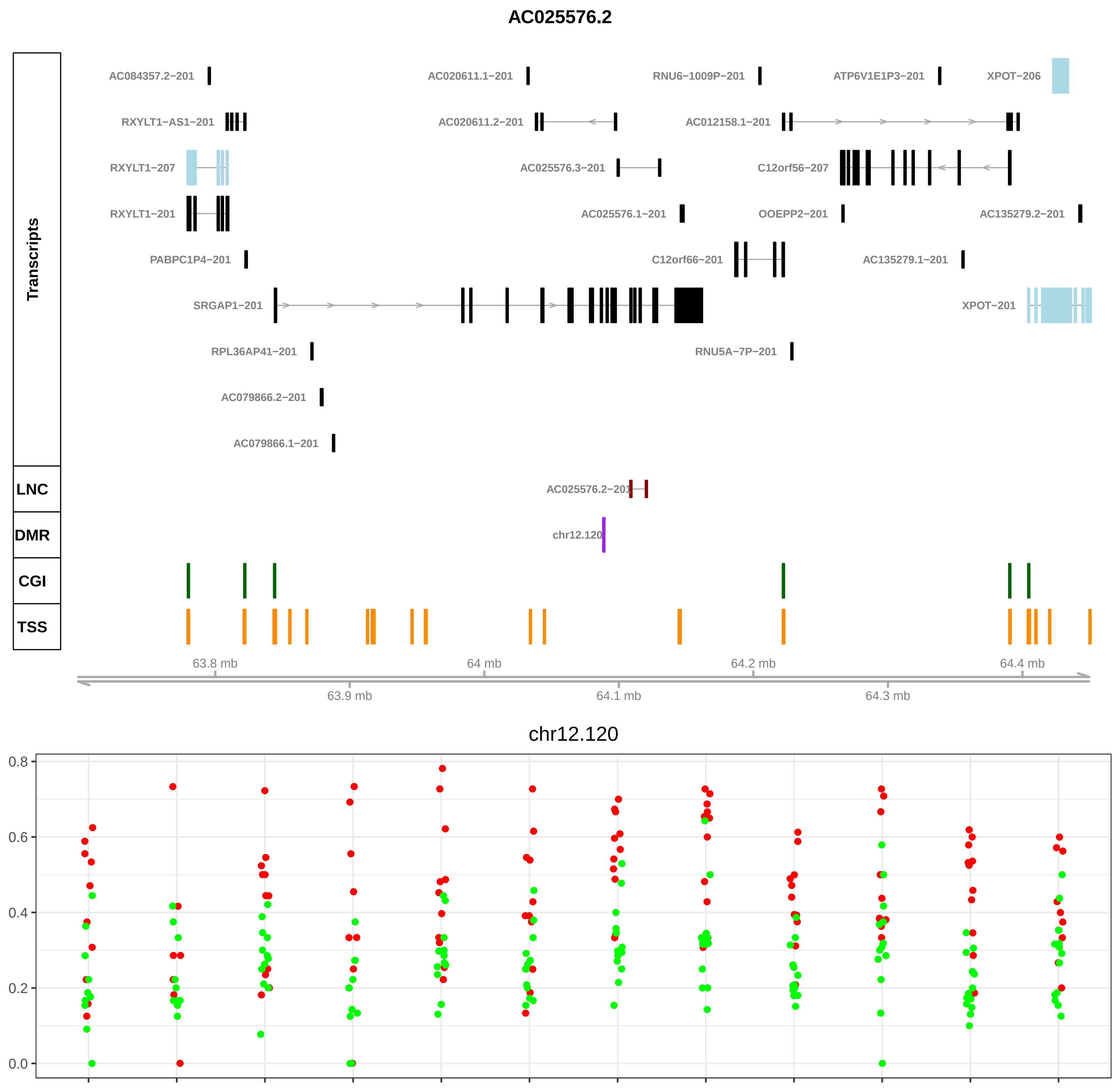
AC025576.2
Transcripts
LNC
DMR
CGI
TSS
AC084357.2−201
AC020611.1−201
RNU6−1009P−201
ATP6V1E1P3−201
XPOT−206
RXYLT1−AS1−201
AC020611.2−201
AC012158.1−201
RXYLT1−207
AC025576.3−201
C12orf56−207
RXYLT1−201
AC025576.1−201
OOEPP2−201
AC135279.2−201
PABPC1P4−201
C12orf66−201
AC135279.1−201
SRGAP1−201
XPOT−201
RPL36AP41−201
RNU5A−7P−201
AC079866.2−201
AC079866.1−201
AC025576.2−201
chr12.120
63.8 mb
64 mb
64.2 mb
64.4 mb
63.9 mb
64.1 mb
64.3 mb
chr12.120
0.8
0.6
0.4
0.2
0.0

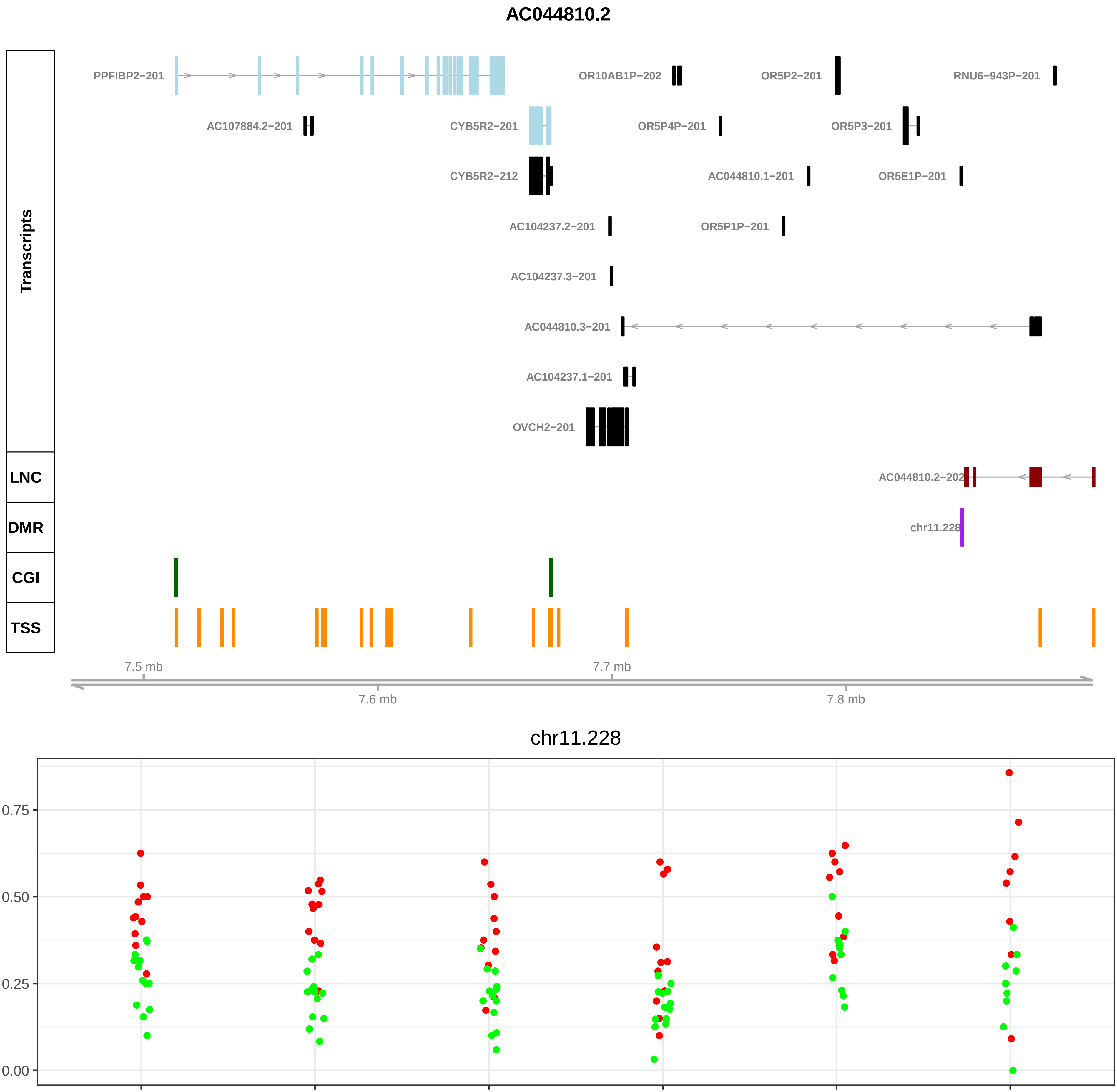

AC044810.2
Transcripts
PPFIBP2−201
OR10AB1P−202
OR5P2−201
RNU6−943P−201
AC107884.2−201
CYB5R2−201
OR5P4P−201
OR5P3−201
CYB5R2−212
AC044810.1−201
OR5E1P−201
AC104237.2−201
OR5P1P−201
AC104237.3−201
AC044810.3−201
AC104237.1−201
OVCH2−201
LNC
AC044810.2−202
DMR
chr11.228
CGI
TSS
7.5 mb
7.7 mb
7.6 mb
7.8 mb
chr11.228
0.75
0.50
0.25
0.00

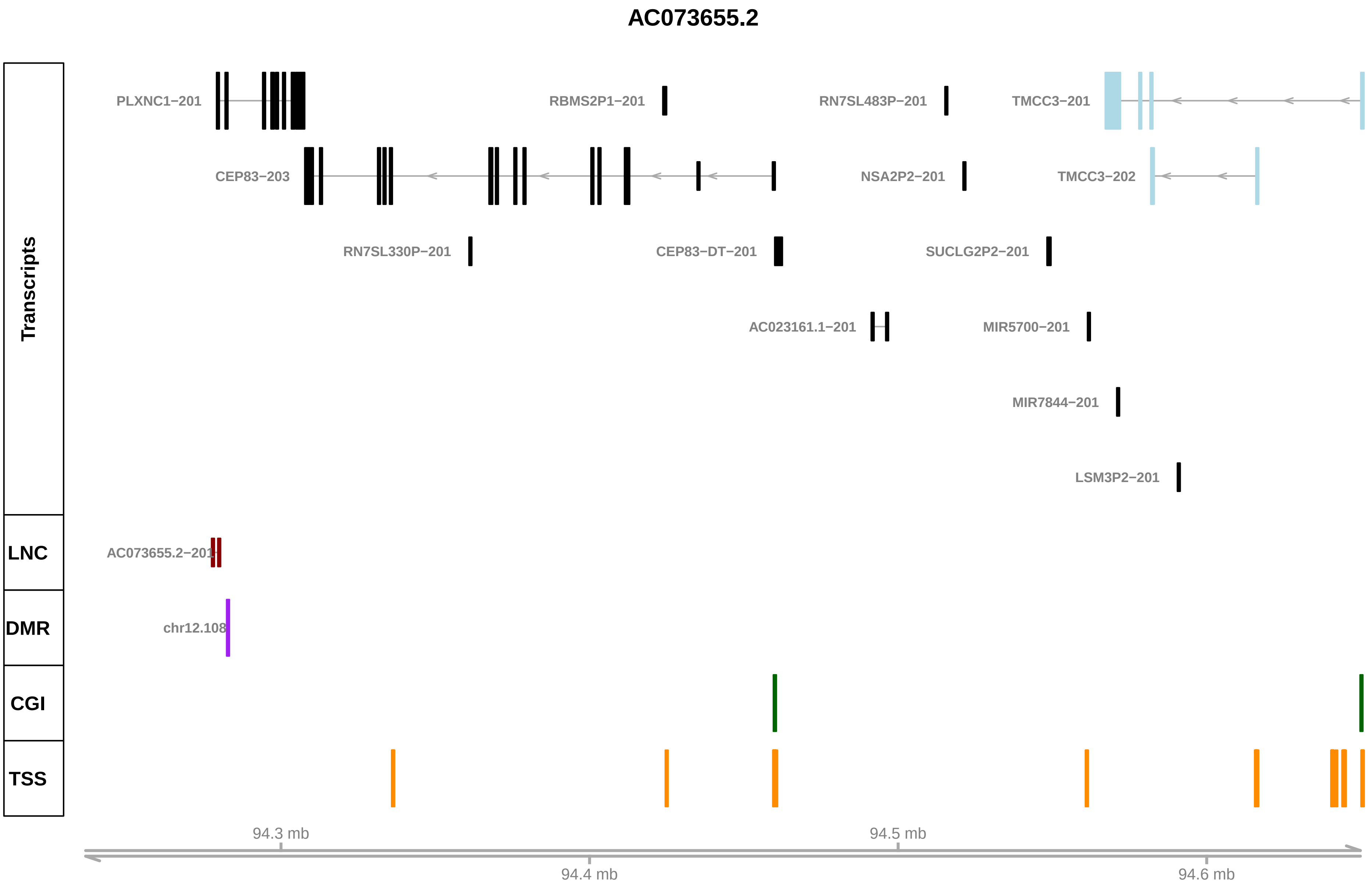

AC073655.2
Transcripts
PLXNC1−201
RBMS2P1−201
RN7SL483P−201
TMCC3−201
CEP83−203
NSA2P2−201
TMCC3−202
RN7SL330P−201
CEP83−DT−201
SUCLG2P2−201
AC023161.1−201
MIR5700−201
MIR7844−201
LSM3P2−201
LNC
AC073655.2−201
DMR
chr12.108
CGI
TSS
94.3 mb
94.5 mb
94.4 mb
94.6 mb

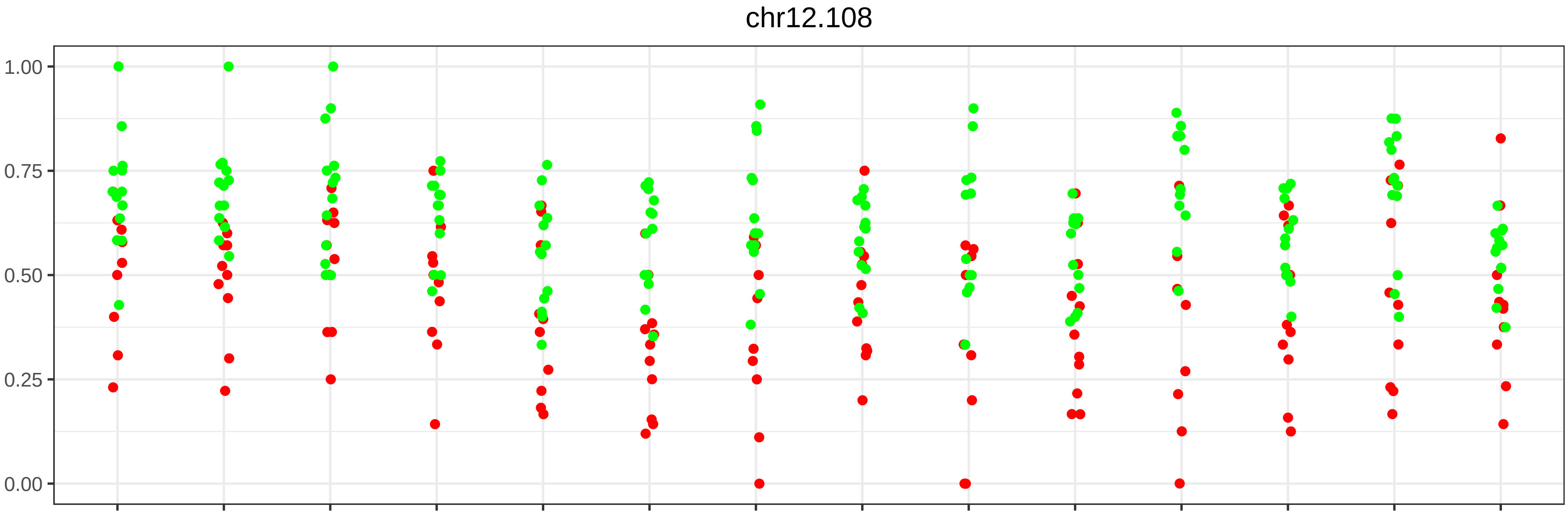

chr12.108
1.00
0.75
0.50
0.25
0.00

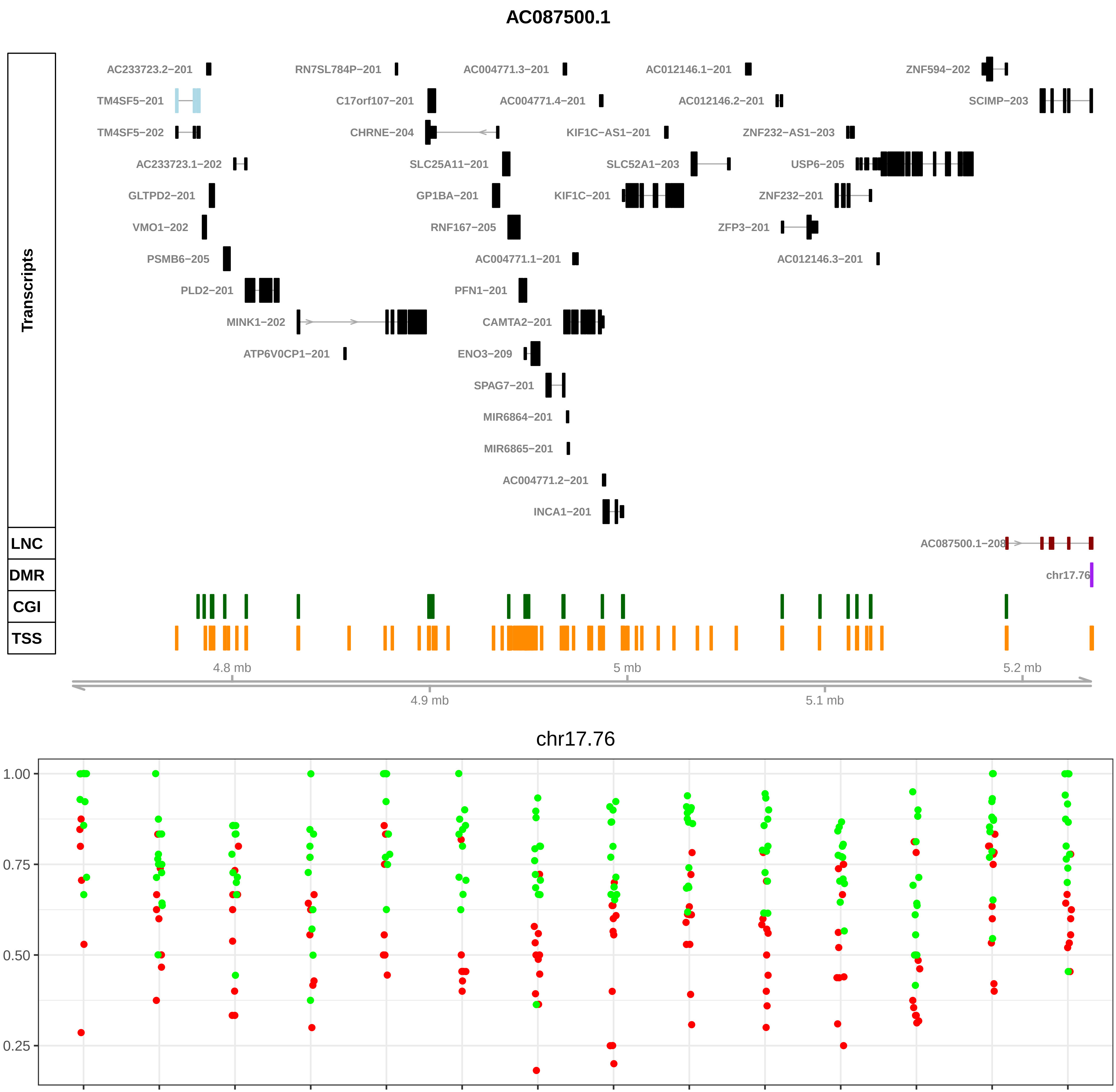

AC087500.1
Transcripts
AC233723.2−201
RN7SL784P−201
AC004771.3−201
AC012146.1−201
ZNF594−202
TM4SF5−201
C17orf107−201
AC004771.4−201
AC012146.2−201
SCIMP−203
TM4SF5−202
CHRNE−204
KIF1C−AS1−201
ZNF232−AS1−203
AC233723.1−202
SLC25A11−201
SLC52A1−203
USP6−205
GLTPD2−201
GP1BA−201
KIF1C−201
ZNF232−201
VMO1−202
RNF167−205
ZFP3−201
PSMB6−205
AC004771.1−201
AC012146.3−201
PLD2−201
PFN1−201
MINK1−202
CAMTA2−201
ATP6V0CP1−201
ENO3−209
SPAG7−201
MIR6864−201
MIR6865−201
AC004771.2−201
INCA1−201
LNC
AC087500.1−208
DMR
chr17.76
CGI
TSS
4.8 mb
5 mb
5.2 mb
4.9 mb
5.1 mb
chr17.76
1.00
0.75
0.50
0.25

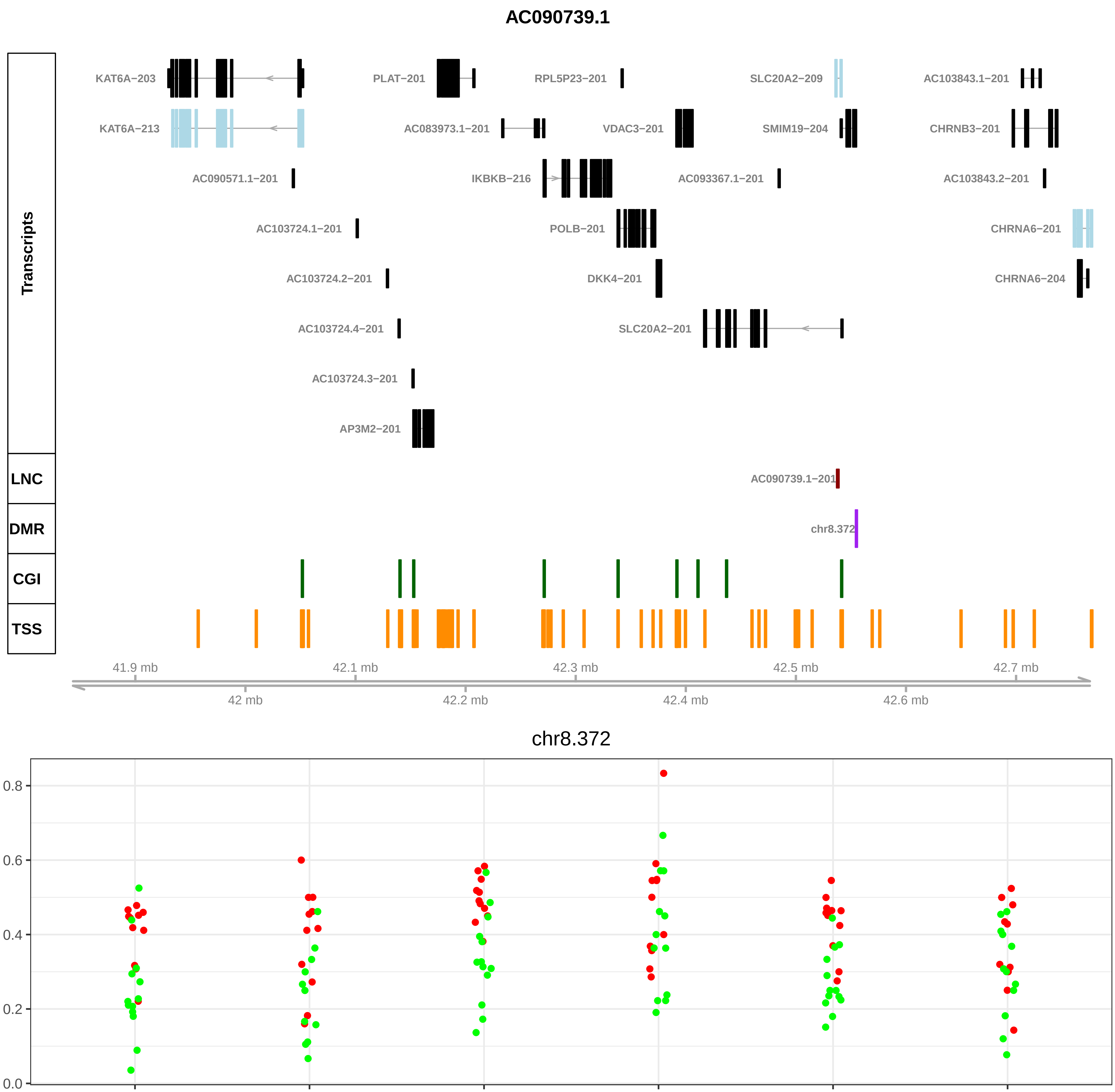

AC090739.1
Transcripts
KAT6A−203
PLAT−201
RPL5P23−201
SLC20A2−209
AC103843.1−201
KAT6A−213
AC083973.1−201
VDAC3−201
SMIM19−204
CHRNB3−201
AC090571.1−201
IKBKB−216
AC093367.1−201
AC103843.2−201
AC103724.1−201
POLB−201
CHRNA6−201
AC103724.2−201
DKK4−201
CHRNA6−204
AC103724.4−201
SLC20A2−201
AC103724.3−201
AP3M2−201
LNC
AC090739.1−201
DMR
chr8.372
CGI
TSS
41.9 mb
42.1 mb
42.3 mb
42.5 mb
42.7 mb
42 mb
42.2 mb
42.4 mb
42.6 mb
chr8.372
0.8
0.6
0.4
0.2
0.0

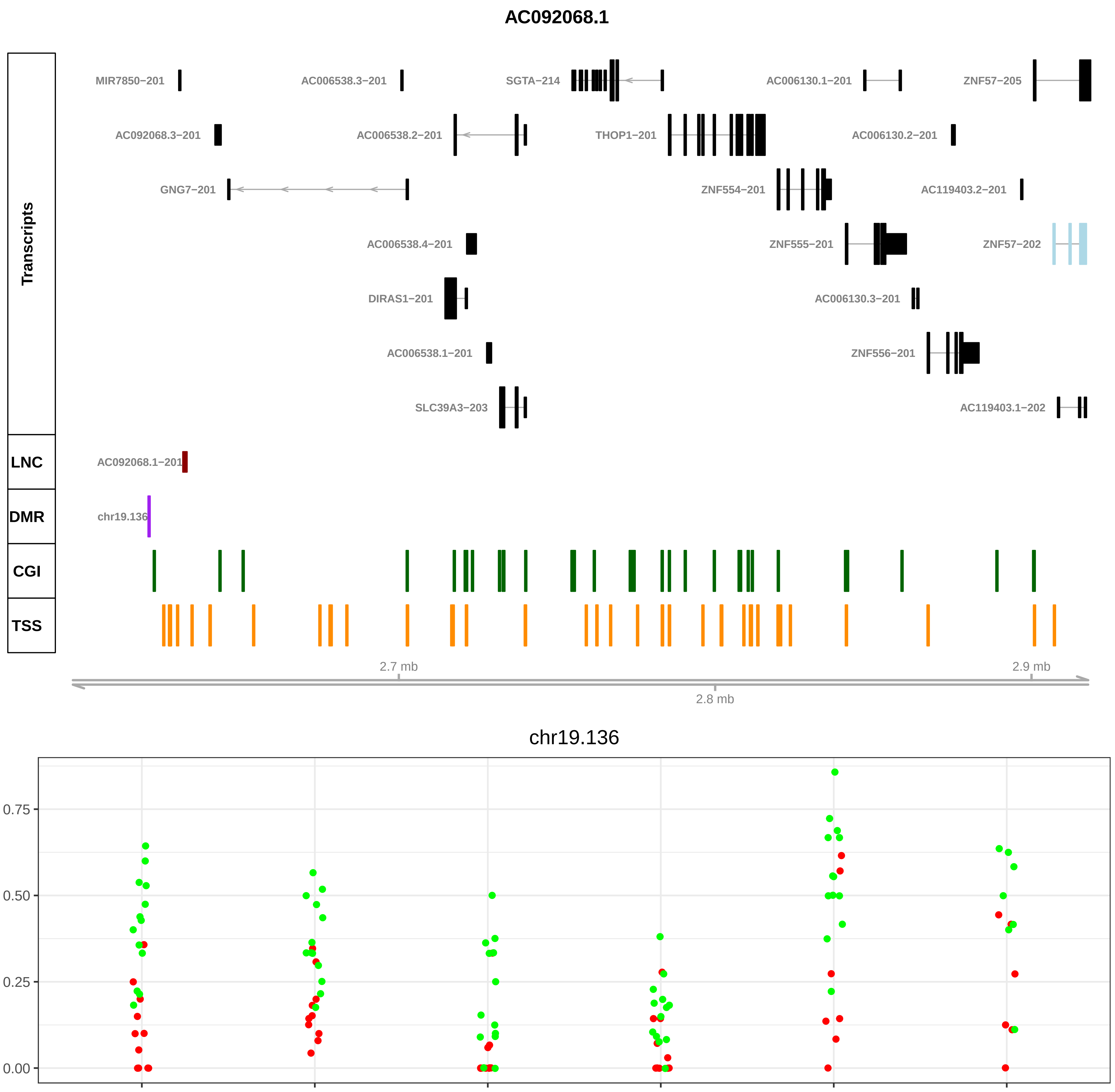

AC092068.1
Transcripts
MIR7850−201
AC006538.3−201
SGTA−214
AC006130.1−201
ZNF57−205
AC092068.3−201
AC006538.2−201
THOP1−201
AC006130.2−201
GNG7−201
ZNF554−201
AC119403.2−201
AC006538.4−201
ZNF555−201
ZNF57−202
DIRAS1−201
AC006130.3−201
AC006538.1−201
ZNF556−201
SLC39A3−203
AC119403.1−202
LNC
AC092068.1−201
DMR
chr19.136
CGI
TSS
2.7 mb
2.8 mb
2.9 mb
chr19.136
0.75
0.50
0.25
0.00

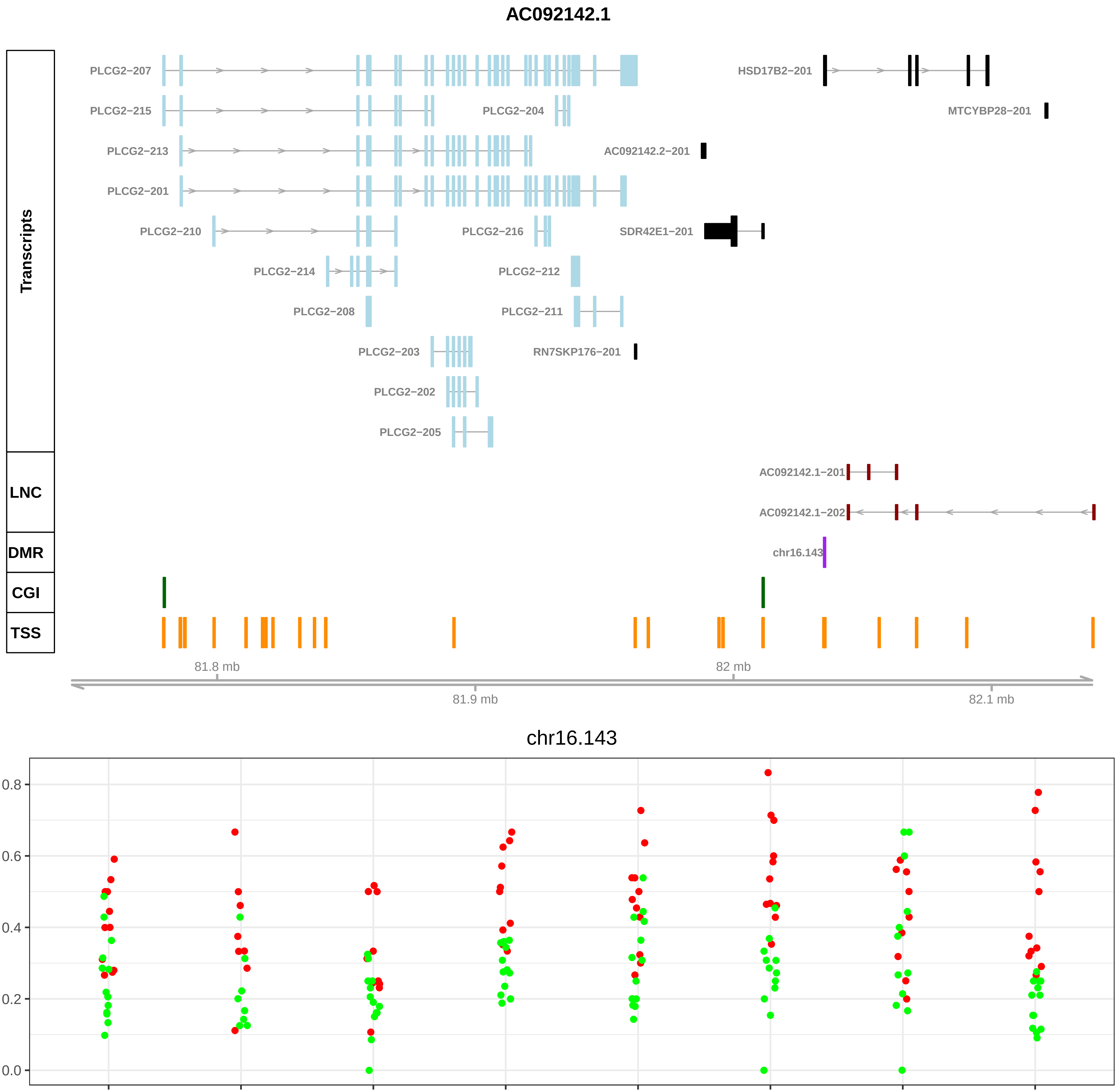

AC092142.1
Transcripts
PLCG2−207
HSD17B2−201
PLCG2−215
PLCG2−204
MTCYBP28−201
PLCG2−213
AC092142.2−201
PLCG2−201
PLCG2−210
PLCG2−216
SDR42E1−201
PLCG2−214
PLCG2−212
PLCG2−208
PLCG2−211
PLCG2−203
RN7SKP176−201
PLCG2−202
PLCG2−205
LNC
AC092142.1−201
AC092142.1−202
DMR
chr16.143
CGI
TSS
81.8 mb
82 mb
81.9 mb
82.1 mb
chr16.143
0.8
0.6
0.4
0.2
0.0

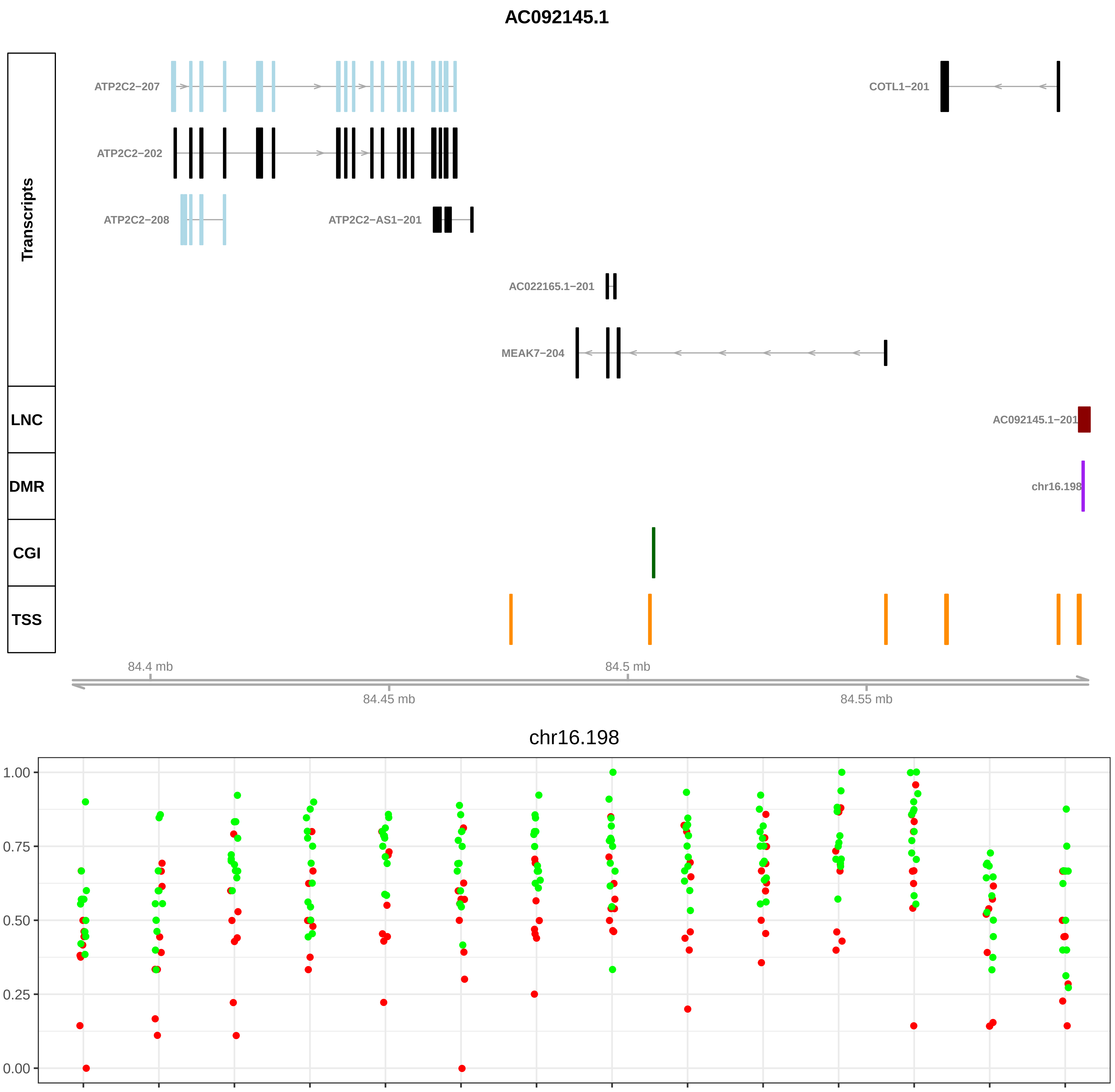
AC092145.1
Transcripts
ATP2C2−207
ATP2C2−202
ATP2C2−208
ATP2C2−AS1−201
COTL1−201
AC022165.1−201
MEAK7−204
LNC
AC092145.1−201
DMR
chr16.198
CGI
TSS
84.4 mb
84.5 mb
84.45 mb
84.55 mb
chr16.198
1.00
0.75
0.50
0.25
0.00

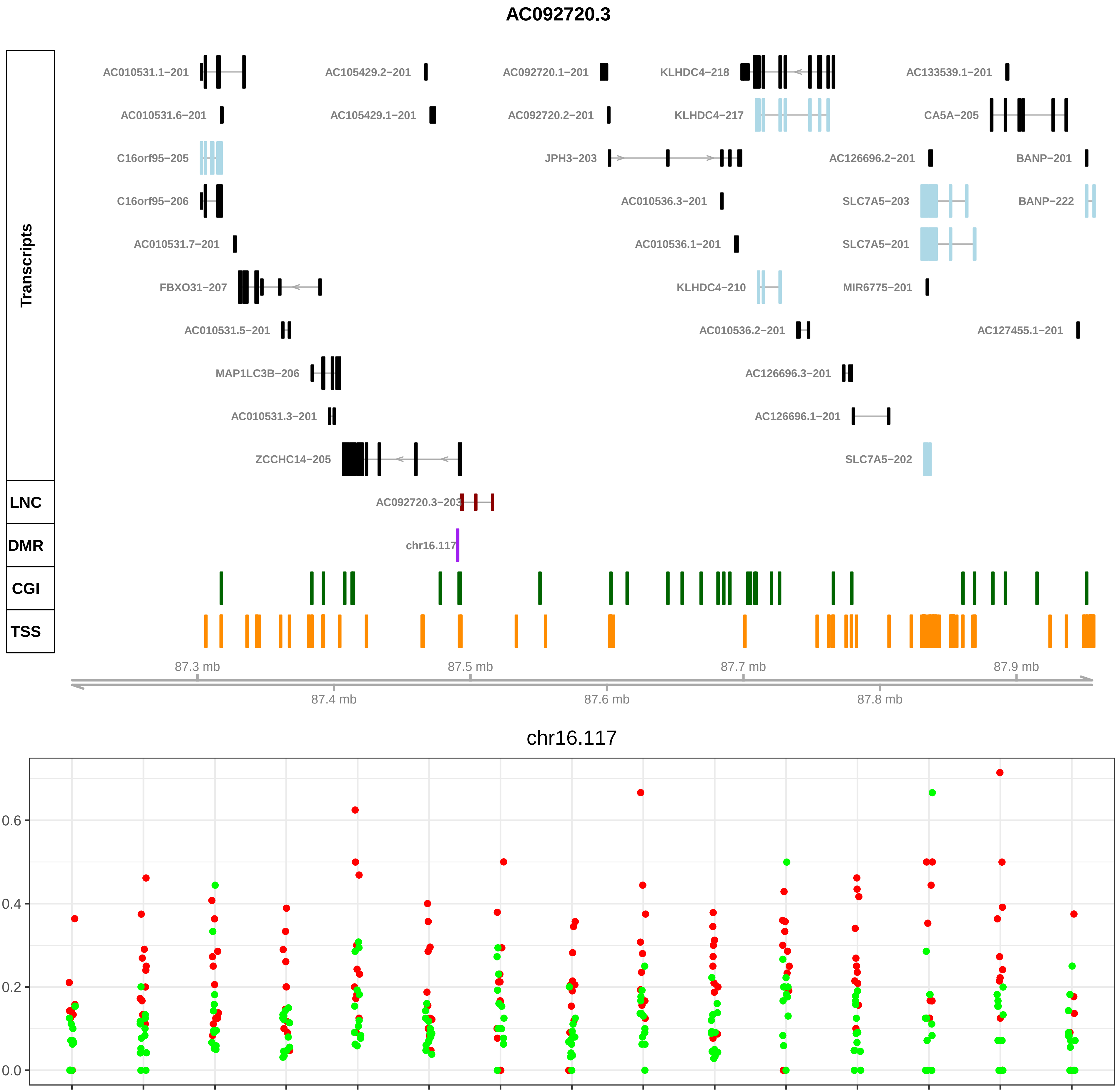

AC092720.3
Transcripts
AC010531.1−201
AC105429.2−201
AC092720.1−201
KLHDC4−218
AC133539.1−201
AC010531.6−201
AC105429.1−201
AC092720.2−201
KLHDC4−217
CA5A−205
C16orf95−205
JPH3−203
AC126696.2−201
BANP−201
C16orf95−206
AC010536.3−201
SLC7A5−203
BANP−222
AC010531.7−201
AC010536.1−201
SLC7A5−201
FBXO31−207
KLHDC4−210
MIR6775−201
AC010531.5−201
AC010536.2−201
AC127455.1−201
MAP1LC3B−206
AC126696.3−201
AC010531.3−201
AC126696.1−201
ZCCHC14−205
SLC7A5−202
LNC
AC092720.3−203
DMR
chr16.117
CGI
TSS
87.3 mb
87.5 mb
87.7 mb
87.9 mb
87.4 mb
87.6 mb
87.8 mb
chr16.117
0.6
0.4
0.2
0.0

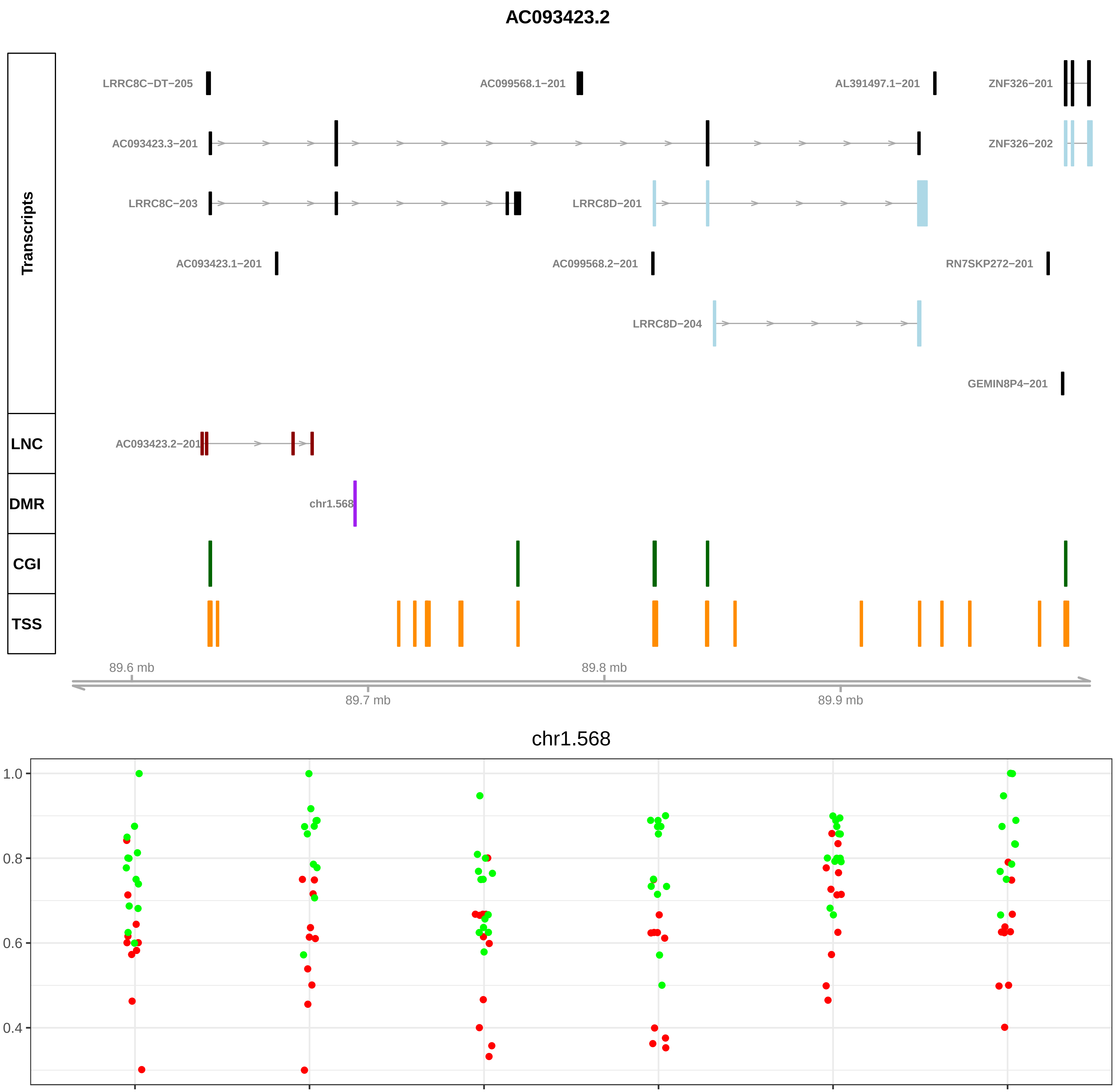

AC093423.2
Transcripts
LRRC8C−DT−205
AC099568.1−201
AL391497.1−201
ZNF326−201
AC093423.3−201
ZNF326−202
LRRC8C−203
LRRC8D−201
AC093423.1−201
AC099568.2−201
RN7SKP272−201
LRRC8D−204
GEMIN8P4−201
LNC
AC093423.2−201
DMR
chr1.568
CGI
TSS
89.6 mb
89.8 mb
89.7 mb
89.9 mb
chr1.568
1.0
0.8
0.6
0.4

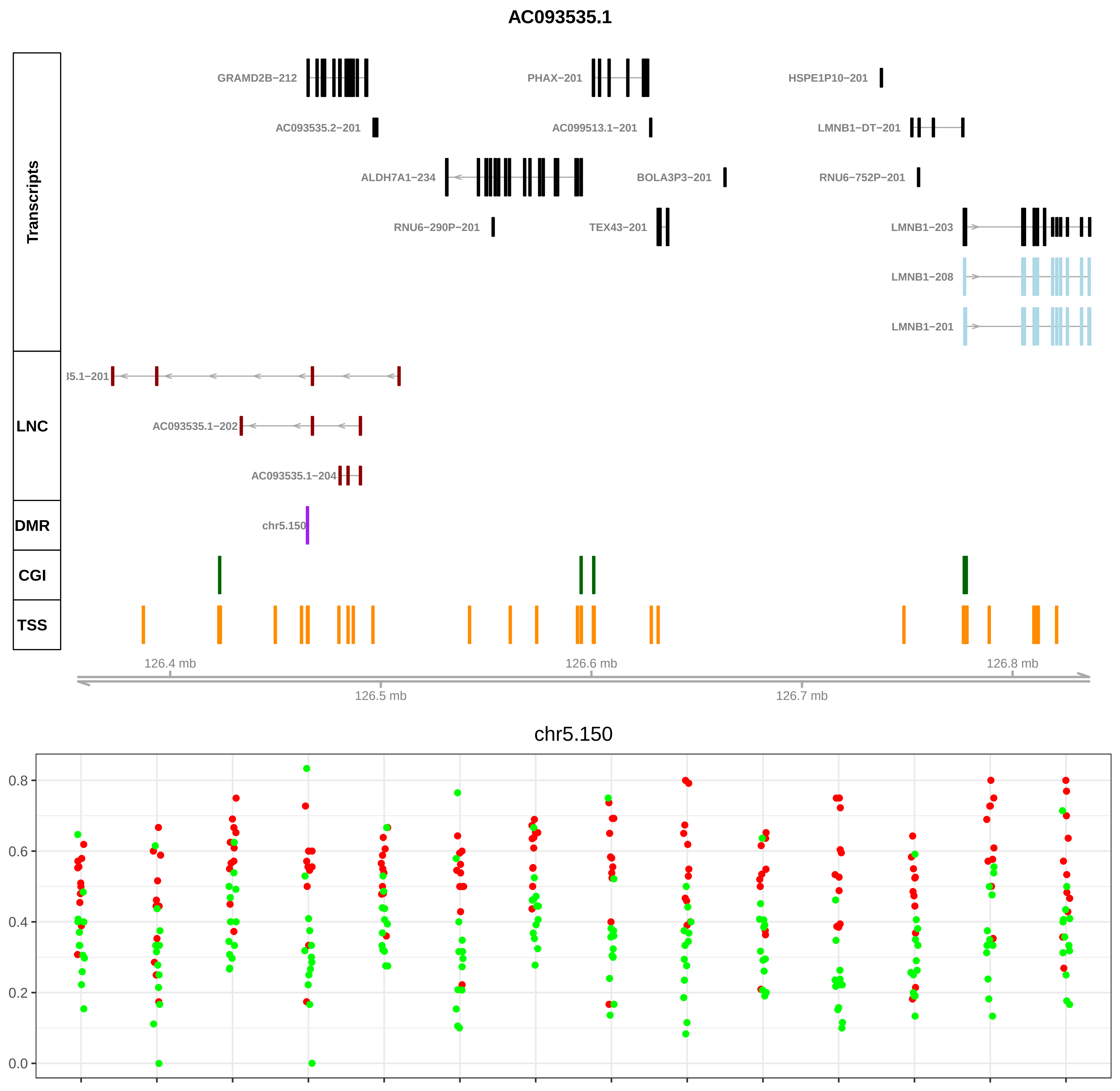

AC093535.1
Transcripts
GRAMD2B−212
PHAX−201
HSPE1P10−201
AC093535.2−201
AC099513.1−201
LMNB1−DT−201
ALDH7A1−234
BOLA3P3−201
RNU6−752P−201
RNU6−290P−201
TEX43−201
LMNB1−203
LMNB1−208
LMNB1−201
LNC
35.1−201
AC093535.1−202
AC093535.1−204
DMR
chr5.150
CGI
TSS
126.4 mb
126.6 mb
126.8 mb
126.5 mb
126.7 mb
chr5.150
0.8
0.6
0.4
0.2
0.0

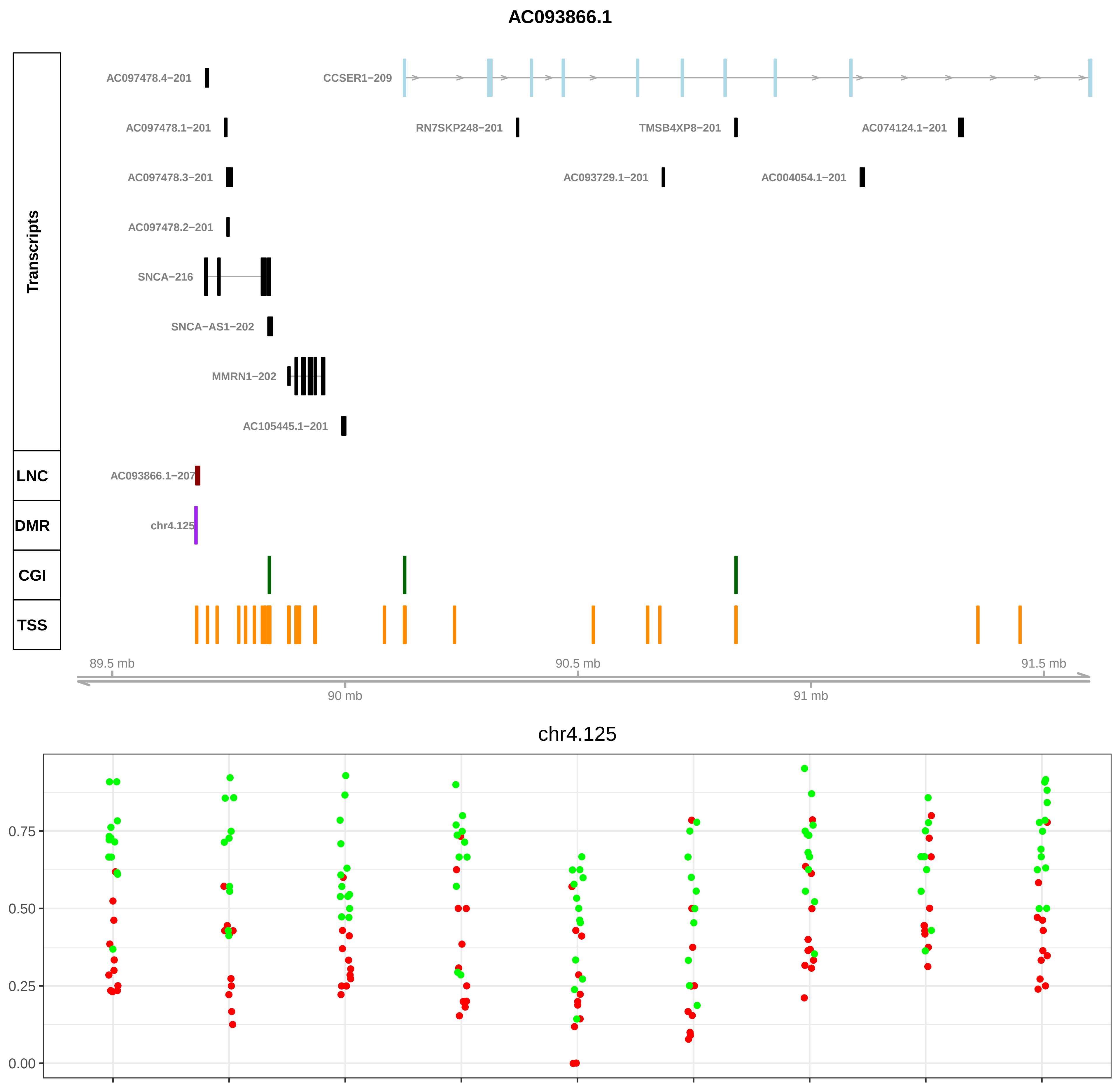

AC093866.1
Transcripts
AC097478.4−201
CCSER1−209
AC097478.1−201
RN7SKP248−201
TMSB4XP8−201
AC074124.1−201
AC097478.3−201
AC093729.1−201
AC004054.1−201
AC097478.2−201
SNCA−216
SNCA−AS1−202
MMRN1−202
AC105445.1−201
LNC
AC093866.1−207
DMR
chr4.125
CGI
TSS
89.5 mb
90.5 mb
91.5 mb
90 mb
91 mb
chr4.125
0.75
0.50
0.25
0.00

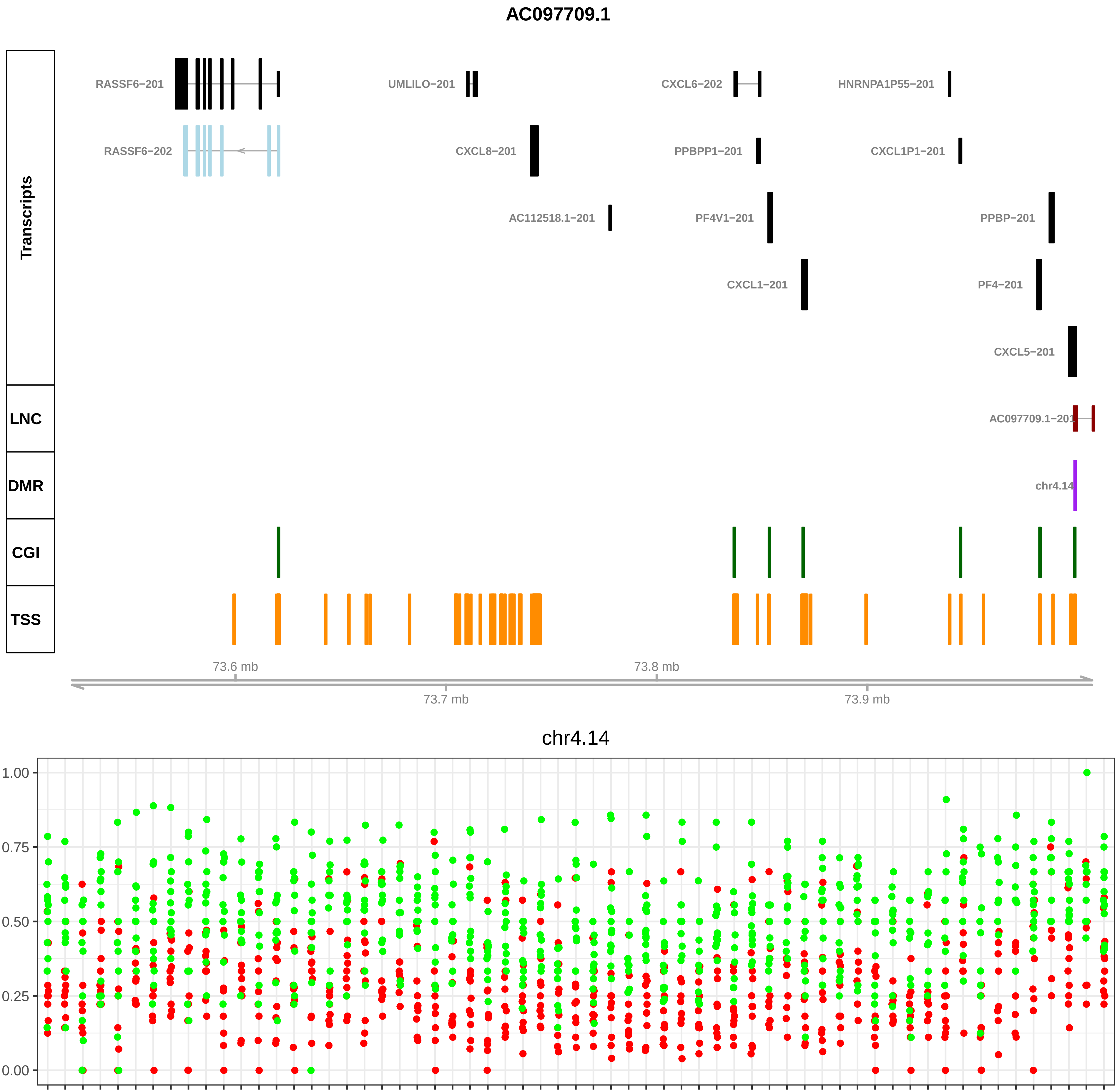
AC097709.1
Transcripts
RASSF6−201
UMLILO−201
CXCL6−202
HNRNPA1P55−201
RASSF6−202
CXCL8−201
PPBPP1−201
CXCL1P1−201
AC112518.1−201
PF4V1−201
PPBP−201
CXCL1−201
PF4−201
CXCL5−201
LNC
AC097709.1−201
DMR
chr4.14
CGI
TSS
73.6 mb
73.8 mb
73.7 mb
73.9 mb
chr4.14
1.00
0.75
0.50
0.25
0.00

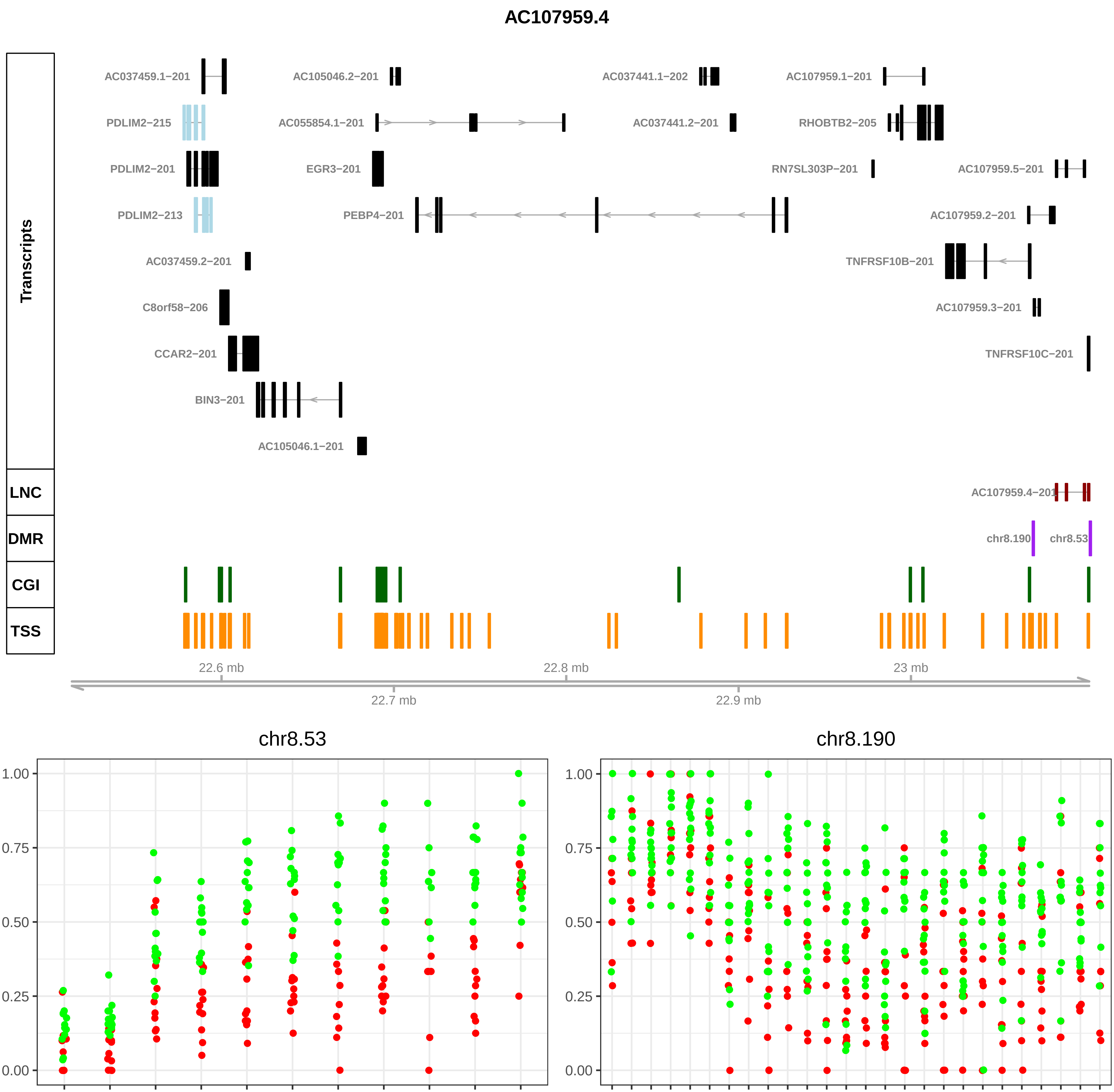

AC107959.4
Transcripts
AC037459.1−201
AC105046.2−201
AC037441.1−202
AC107959.1−201
PDLIM2−215
AC055854.1−201
AC037441.2−201
RHOBTB2−205
PDLIM2−201
EGR3−201
RN7SL303P−201
AC107959.5−201
PDLIM2−213
PEBP4−201
AC107959.2−201
AC037459.2−201
TNFRSF10B−201
C8orf58−206
AC107959.3−201
CCAR2−201
TNFRSF10C−201
BIN3−201
AC105046.1−201
LNC
AC107959.4−201
DMR
chr8.190
chr8.53
CGI
TSS
22.6 mb
22.7 mb
22.8 mb
22.9 mb
23 mb
chr8.53
chr8.190
1.00
0.75
0.50
0.25
0.00

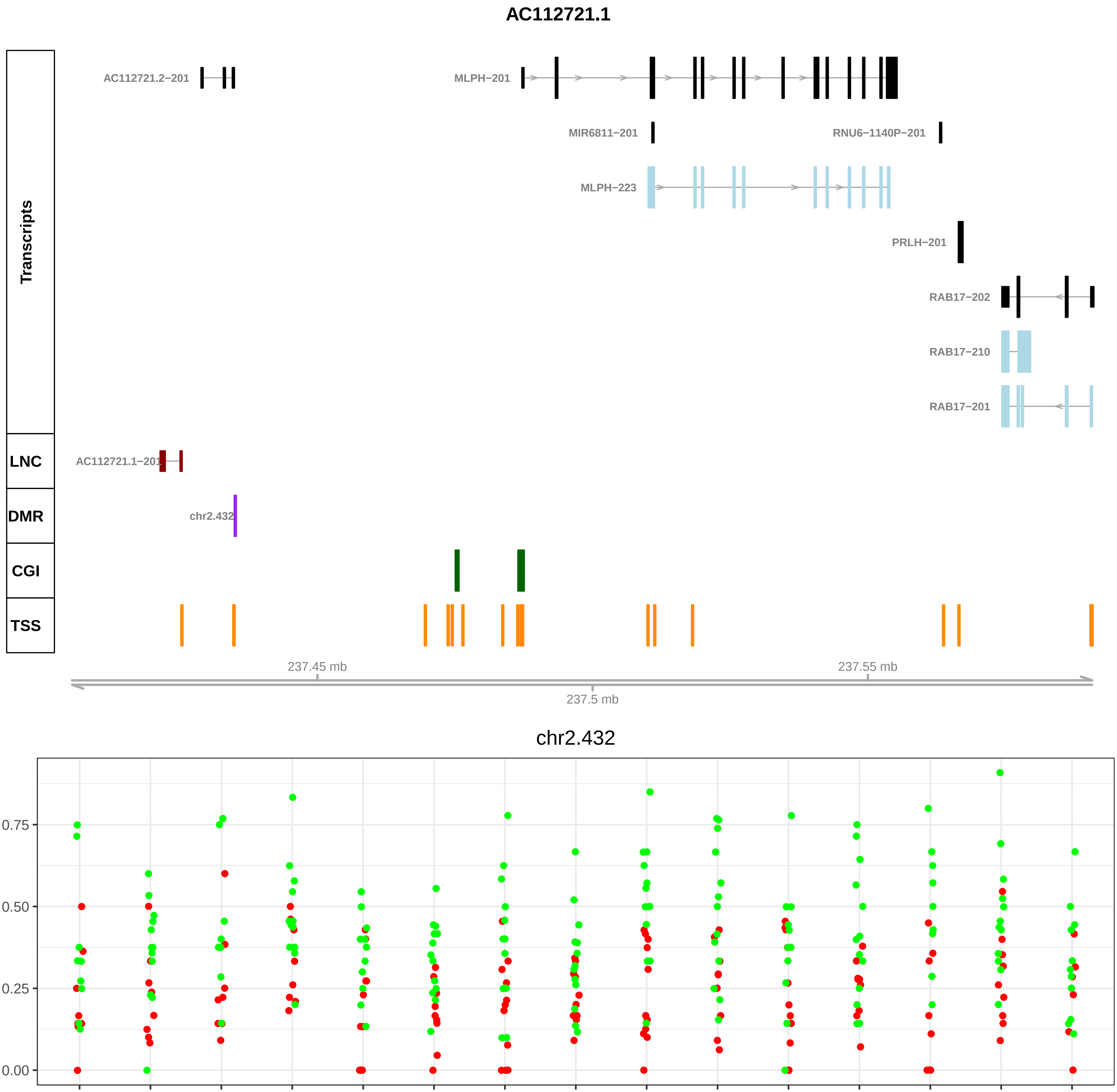

AC112721.1
Transcripts
AC112721.2−201
MLPH−201
MIR6811−201
RNU6−1140P−201
MLPH−223
PRLH−201
RAB17−202
RAB17−210
RAB17−201
LNC
AC112721.1−201
DMR
chr2.432
CGI
TSS
237.45 mb
237.55 mb
237.5 mb
chr2.432
0.75
0.50
0.25
0.00

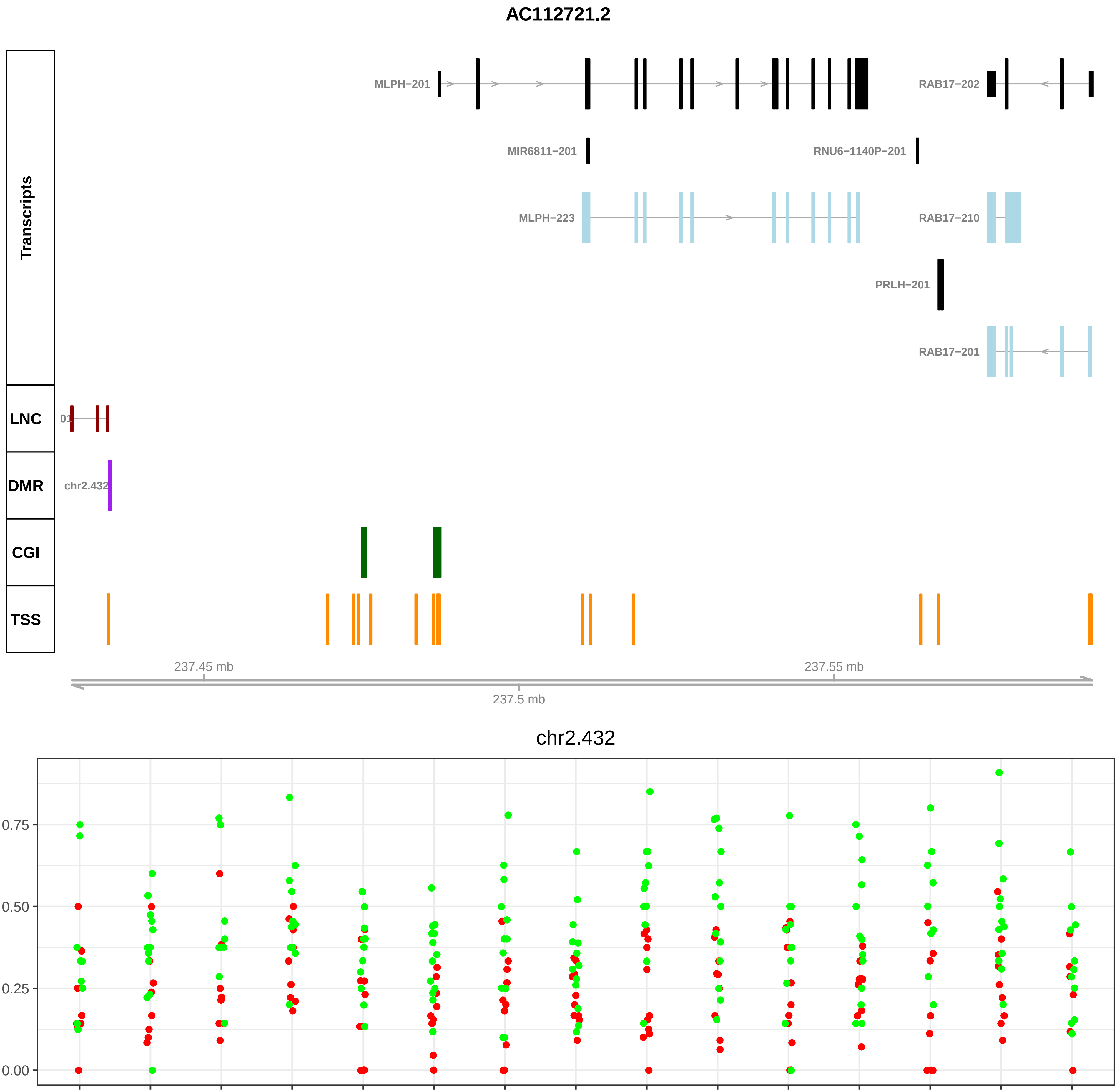

AC112721.2
Transcripts
MLPH−201
RAB17−202
MIR6811−201
RNU6−1140P−201
MLPH−223
RAB17−210
PRLH−201
RAB17−201
LNC
01
DMR
chr2.432
CGI
TSS
237.45 mb
237.5 mb
237.55 mb
chr2.432
0.75
0.50
0.25
0.00

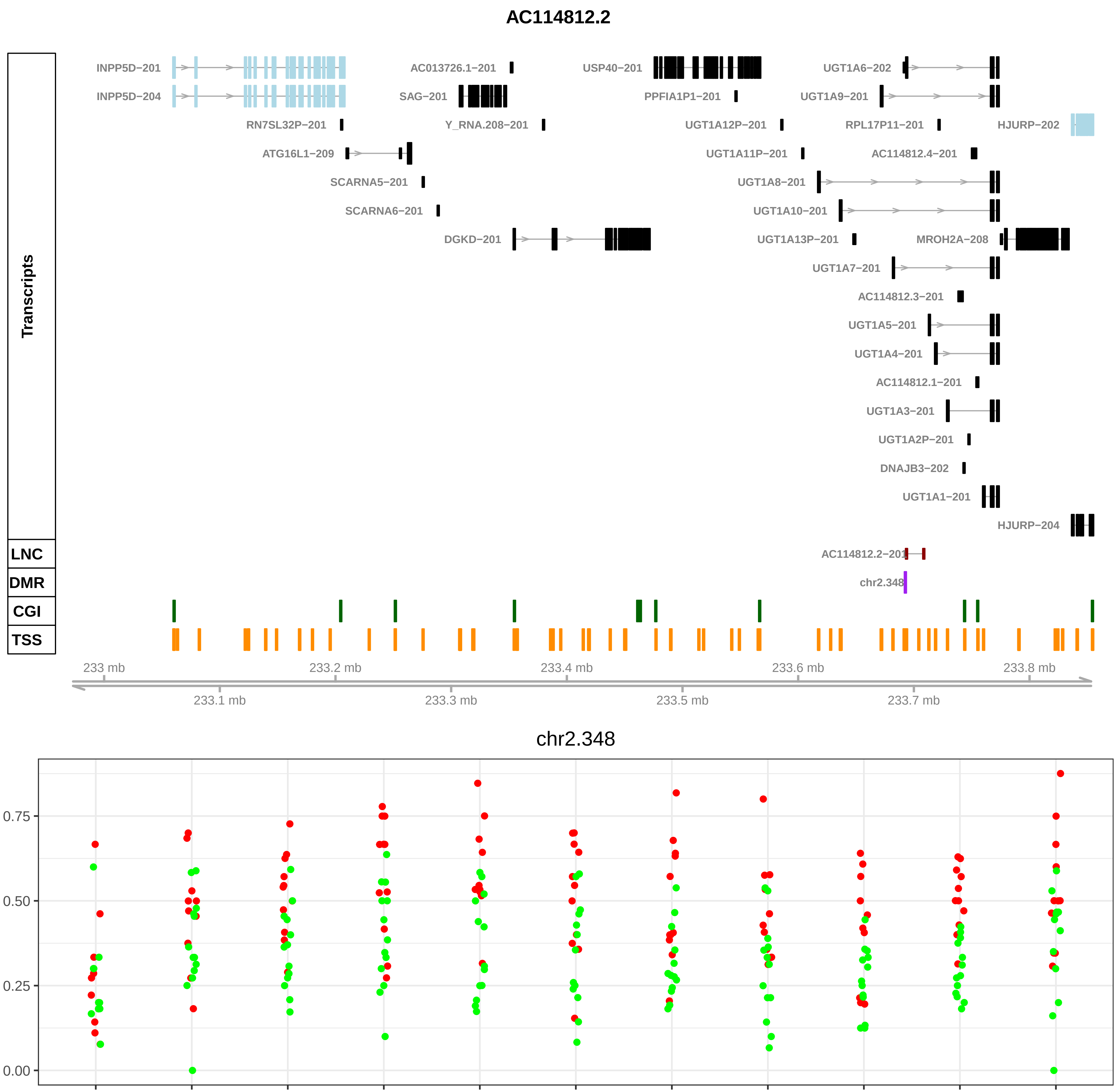

AC114812.2
Transcripts
LNC
DMR
CGI
TSS
INPP5D−201
INPP5D−204
RN7SL32P−201
ATG16L1−209
SCARNA5−201
SCARNA6−201
AC013726.1−201
SAG−201
Y_RNA.208−201
DGKD−201
USP40−201
PPFIA1P1−201
UGT1A12P−201
UGT1A11P−201
UGT1A8−201
UGT1A10−201
UGT1A13P−201
UGT1A6−202
UGT1A9−201
RPL17P11−201
AC114812.4−201
MROH2A−208
UGT1A7−201
AC114812.3−201
UGT1A5−201
UGT1A4−201
AC114812.1−201
UGT1A3−201
UGT1A2P−201
DNAJB3−202
UGT1A1−201
HJURP−202
HJURP−204
AC114812.2−201
chr2.348
233 mb
233.1 mb
233.2 mb
233.3 mb
233.4 mb
233.5 mb
233.6 mb
233.7 mb
233.8 mb
chr2.348
0.00
0.25
0.50
0.75

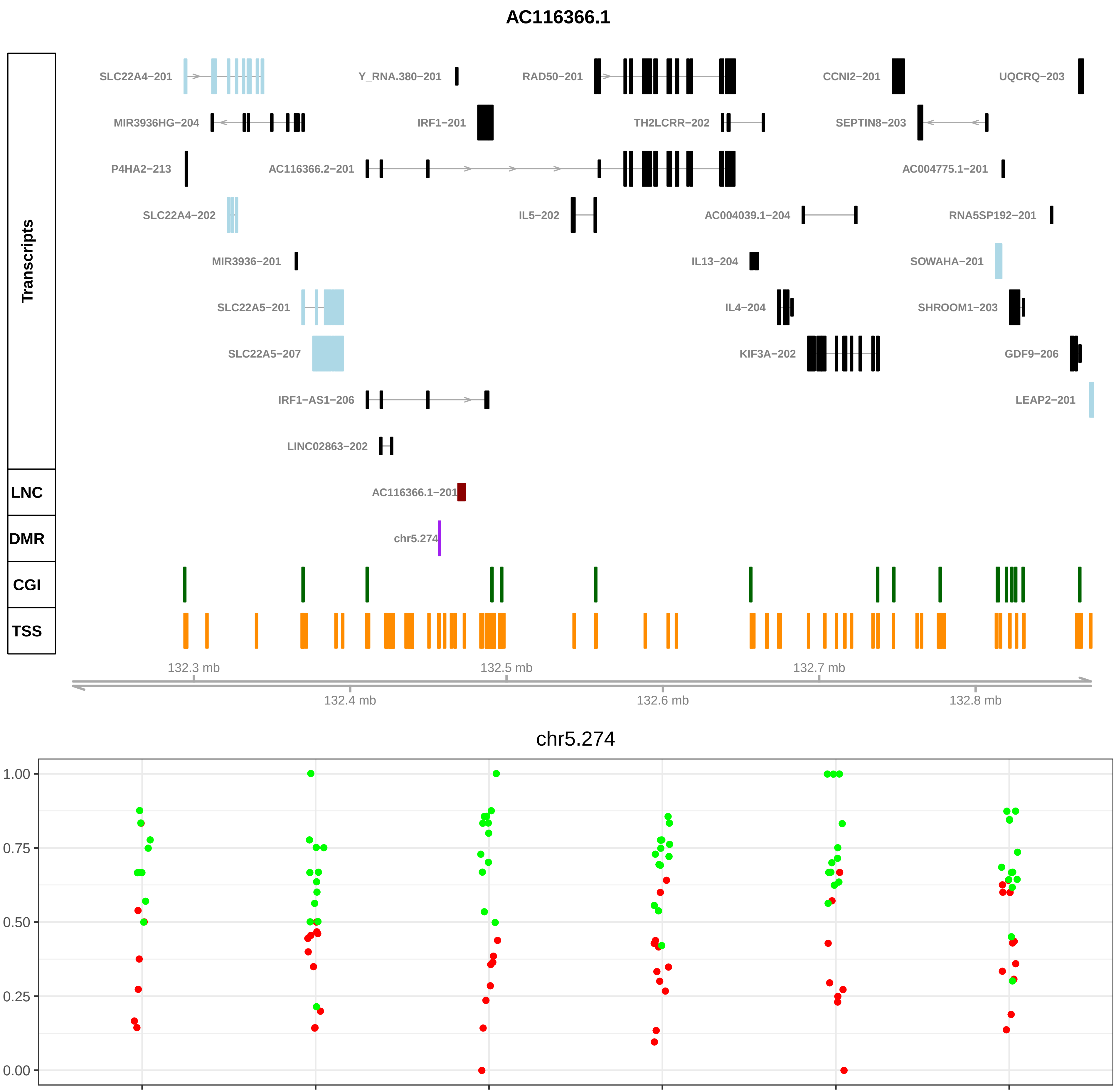
AC116366.1
Transcripts
SLC22A4−201
Y_RNA.380−201
RAD50−201
CCNI2−201
UQCRQ−203
MIR3936HG−204
IRF1−201
TH2LCRR−202
SEPTIN8−203
P4HA2−213
AC116366.2−201
AC004775.1−201
SLC22A4−202
IL5−202
AC004039.1−204
RNA5SP192−201
MIR3936−201
IL13−204
SOWAHA−201
SLC22A5−201
IL4−204
SHROOM1−203
SLC22A5−207
KIF3A−202
GDF9−206
IRF1−AS1−206
LEAP2−201
LINC02863−202
LNC
AC116366.1−201
DMR
chr5.274
CGI
TSS
132.3 mb
132.5 mb
132.7 mb
132.4 mb
132.6 mb
132.8 mb
chr5.274
1.00
0.75
0.50
0.25
0.00

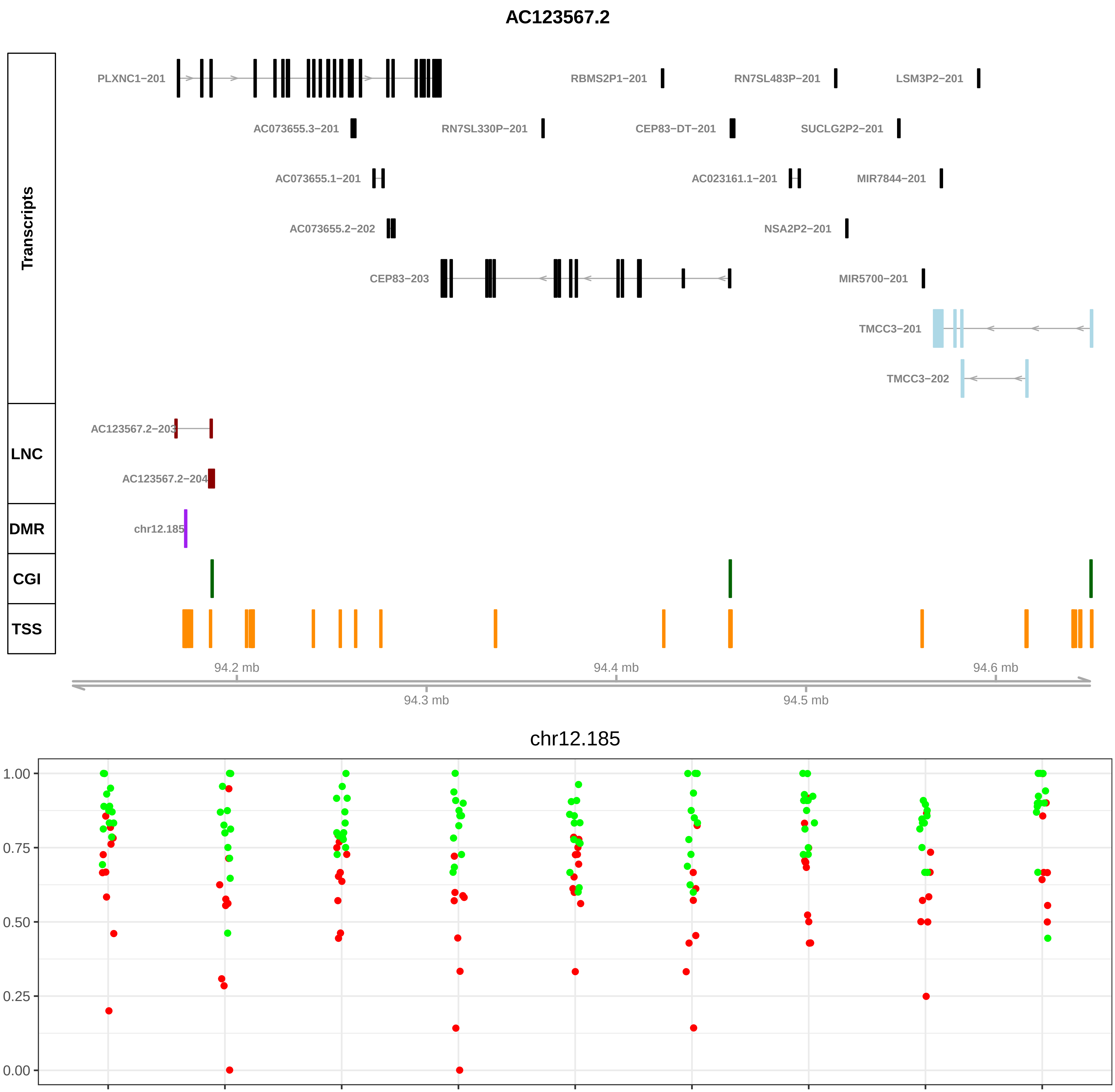
AC123567.2
Transcripts
PLXNC1−201
RBMS2P1−201
RN7SL483P−201
LSM3P2−201
AC073655.3−201
RN7SL330P−201
CEP83−DT−201
SUCLG2P2−201
AC073655.1−201
AC023161.1−201
MIR7844−201
AC073655.2−202
NSA2P2−201
CEP83−203
MIR5700−201
TMCC3−201
TMCC3−202
LNC
AC123567.2−203
AC123567.2−204
DMR
chr12.185
CGI
TSS
94.2 mb
94.4 mb
94.6 mb
94.3 mb
94.5 mb
chr12.185
1.00
0.75
0.50
0.25
0.00

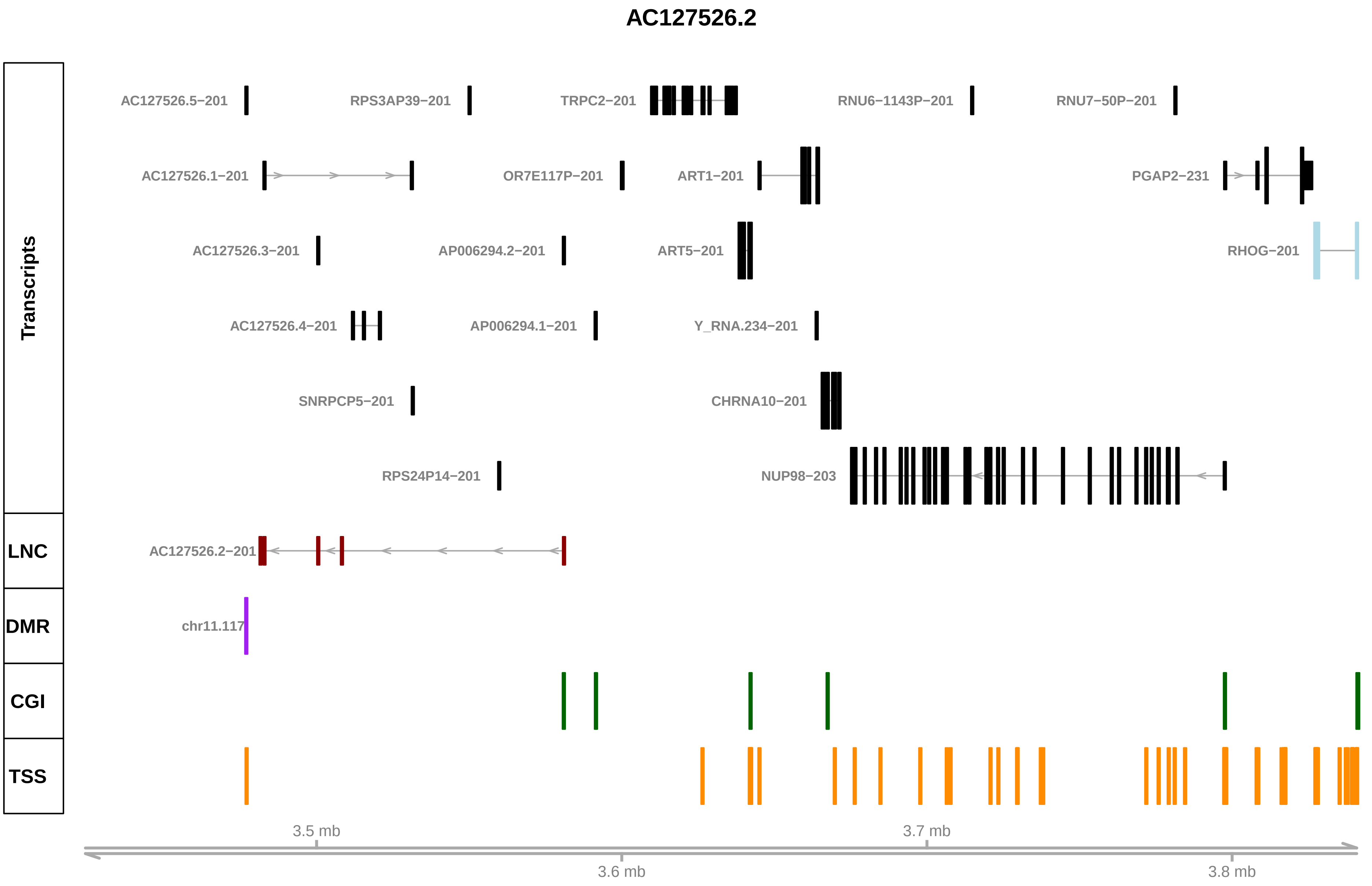

AC127526.2
Transcripts
AC127526.5−201
RPS3AP39−201
TRPC2−201
RNU6−1143P−201
RNU7−50P−201
AC127526.1−201
OR7E117P−201
ART1−201
PGAP2−231
AC127526.3−201
AP006294.2−201
ART5−201
RHOG−201
AC127526.4−201
AP006294.1−201
Y_RNA.234−201
SNRPCP5−201
CHRNA10−201
RPS24P14−201
NUP98−203
LNC
AC127526.2−201
DMR
chr11.117
CGI
TSS
3.5 mb
3.7 mb
3.6 mb
3.8 mb

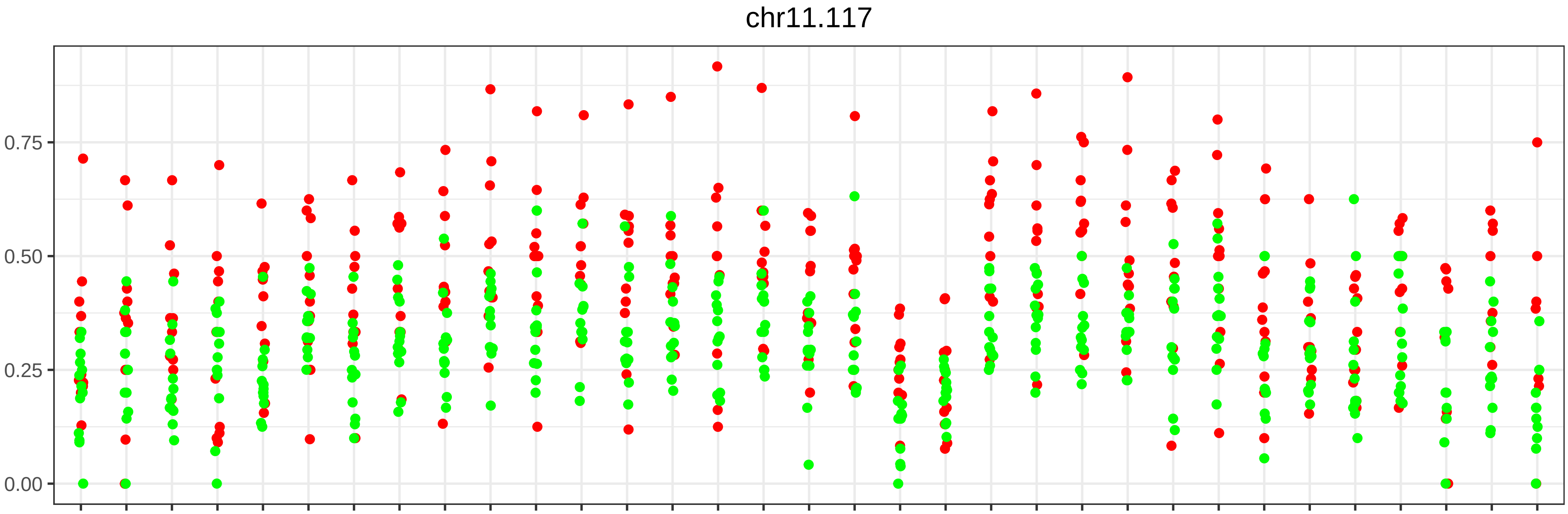

chr11.117
0.75
0.50
0.25
0.00

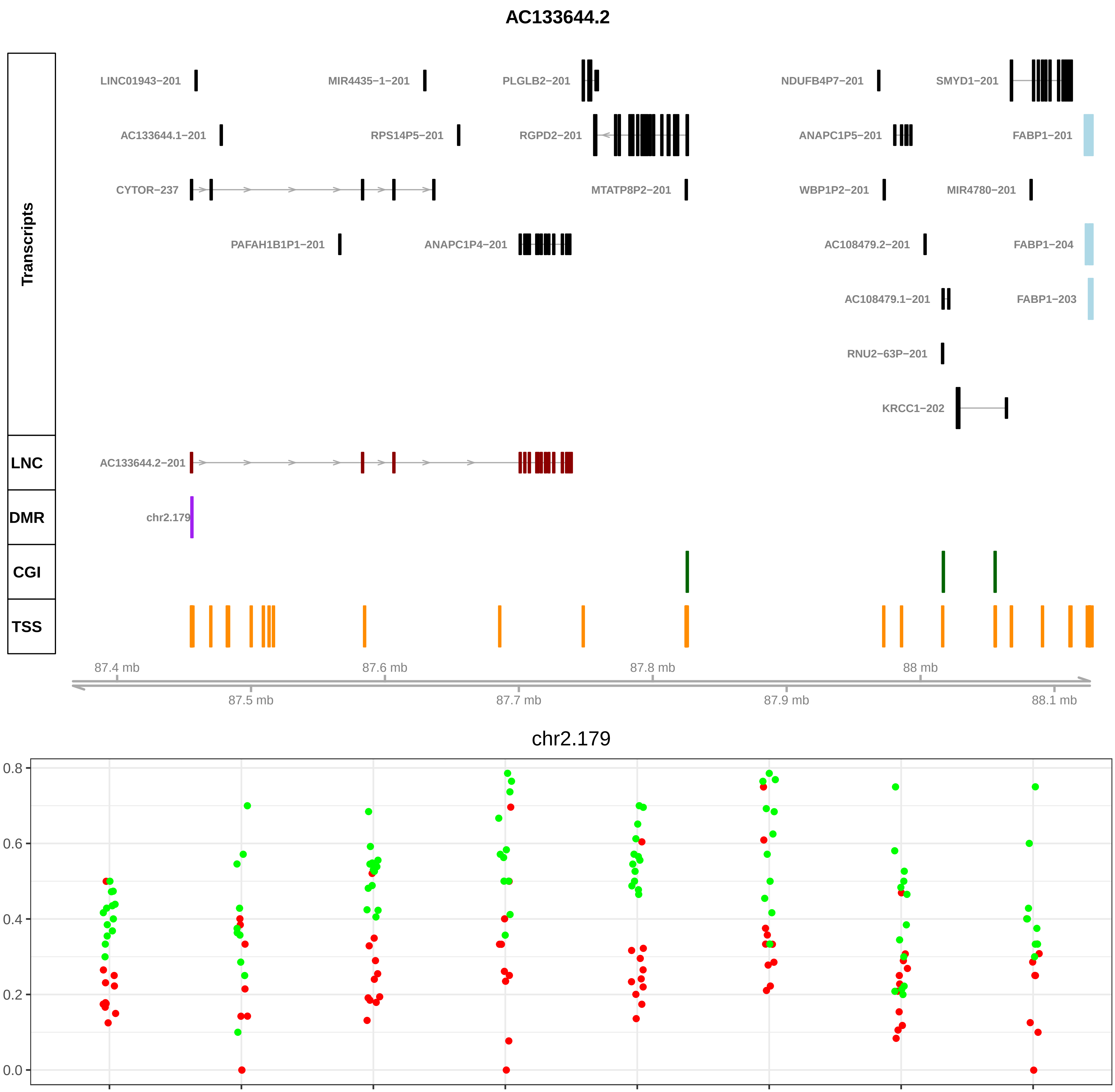

AC133644.2
Transcripts
LINC01943−201
MIR4435−1−201
PLGLB2−201
NDUFB4P7−201
SMYD1−201
AC133644.1−201
RPS14P5−201
RGPD2−201
ANAPC1P5−201
FABP1−201
CYTOR−237
MTATP8P2−201
WBP1P2−201
MIR4780−201
PAFAH1B1P1−201
ANAPC1P4−201
AC108479.2−201
FABP1−204
AC108479.1−201
FABP1−203
RNU2−63P−201
KRCC1−202
LNC
AC133644.2−201
DMR
chr2.179
CGI
TSS
87.4 mb
87.6 mb
87.8 mb
88 mb
87.5 mb
87.7 mb
87.9 mb
88.1 mb
chr2.179
0.8
0.6
0.4
0.2
0.0

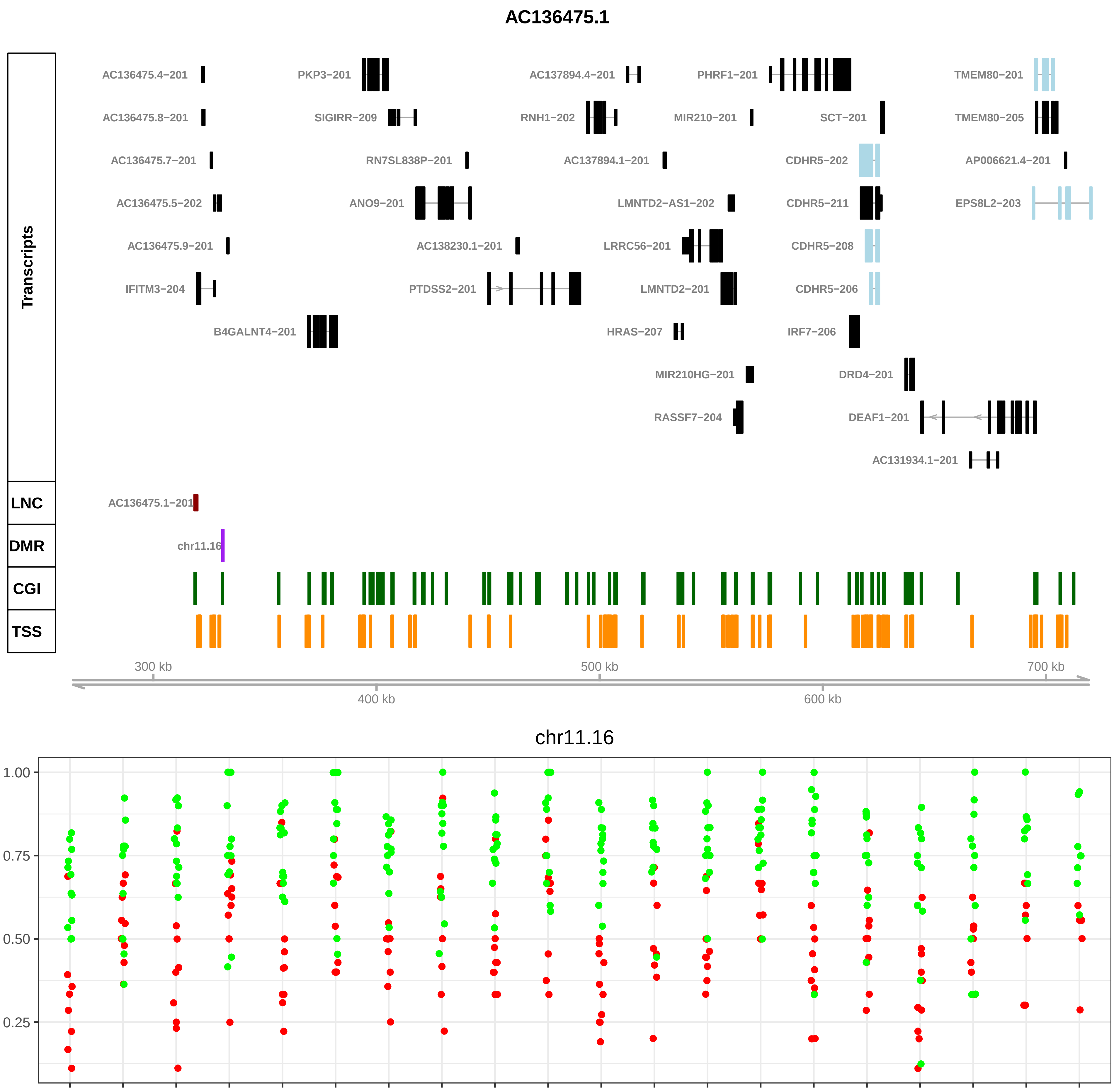

AC136475.1
Transcripts
AC136475.4−201
AC136475.8−201
AC136475.7−201
AC136475.5−202
AC136475.9−201
IFITM3−204
B4GALNT4−201
PKP3−201
SIGIRR−209
RN7SL838P−201
ANO9−201
AC138230.1−201
PTDSS2−201
AC137894.4−201
RNH1−202
AC137894.1−201
LMNTD2−AS1−202
LRRC56−201
LMNTD2−201
HRAS−207
MIR210HG−201
RASSF7−204
PHRF1−201
MIR210−201
SCT−201
CDHR5−202
CDHR5−211
CDHR5−208
CDHR5−206
IRF7−206
DRD4−201
DEAF1−201
AC131934.1−201
TMEM80−201
TMEM80−205
AP006621.4−201
EPS8L2−203
LNC
AC136475.1−201
DMR
chr11.16
CGI
TSS
300 kb
400 kb
500 kb
600 kb
700 kb
chr11.16
1.00
0.75
0.50
0.25

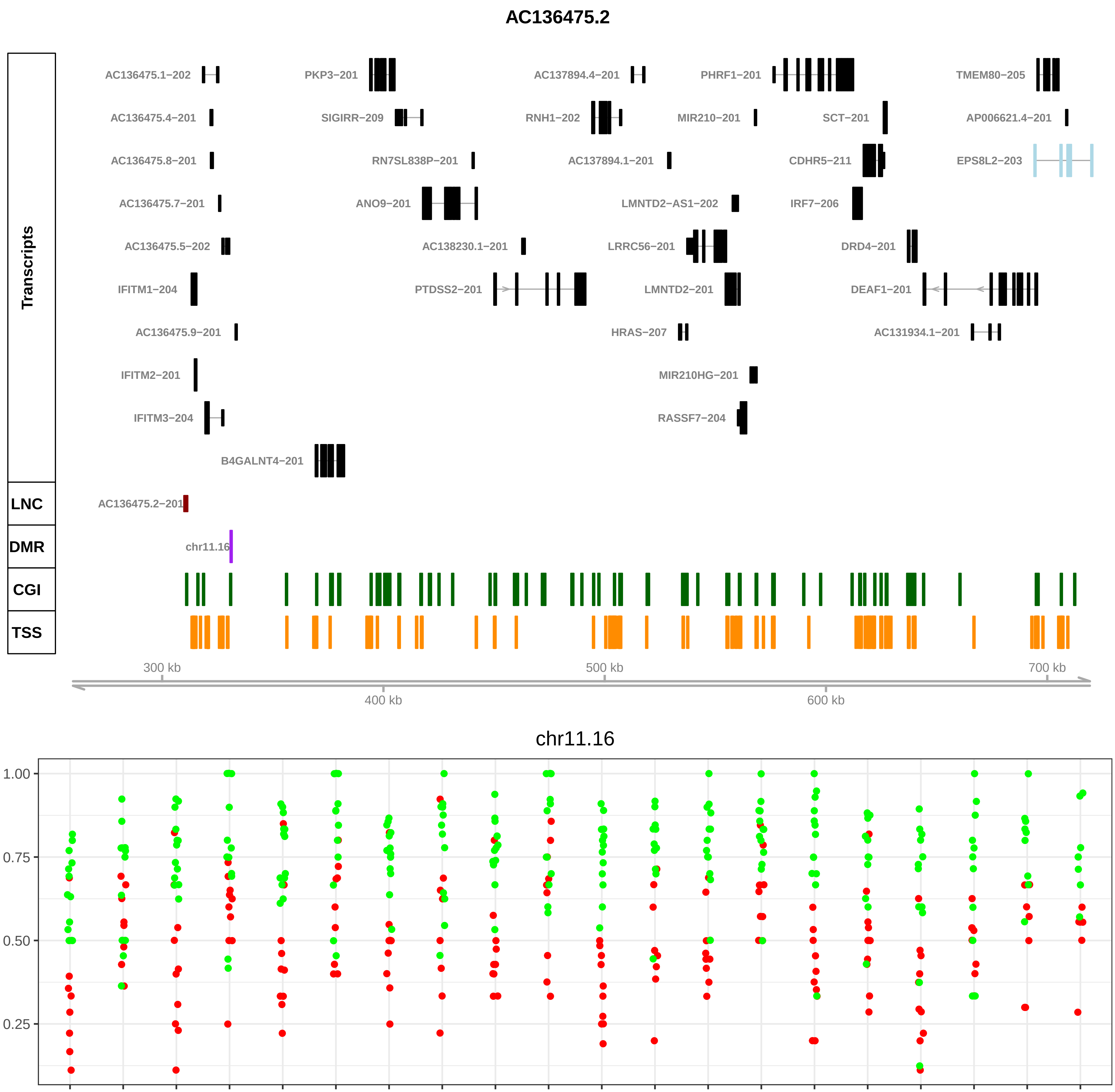

AC136475.2
Transcripts
LNC
DMR
CGI
TSS
AC136475.1−202
PKP3−201
AC137894.4−201
PHRF1−201
TMEM80−205
AC136475.4−201
SIGIRR−209
RNH1−202
MIR210−201
SCT−201
AP006621.4−201
AC136475.8−201
RN7SL838P−201
AC137894.1−201
CDHR5−211
EPS8L2−203
AC136475.7−201
ANO9−201
LMNTD2−AS1−202
IRF7−206
AC136475.5−202
AC138230.1−201
LRRC56−201
DRD4−201
IFITM1−204
PTDSS2−201
LMNTD2−201
DEAF1−201
AC136475.9−201
HRAS−207
AC131934.1−201
IFITM2−201
MIR210HG−201
IFITM3−204
RASSF7−204
B4GALNT4−201
AC136475.2−201
chr11.16
300 kb
500 kb
700 kb
400 kb
600 kb
chr11.16
1.00
0.75
0.50
0.25

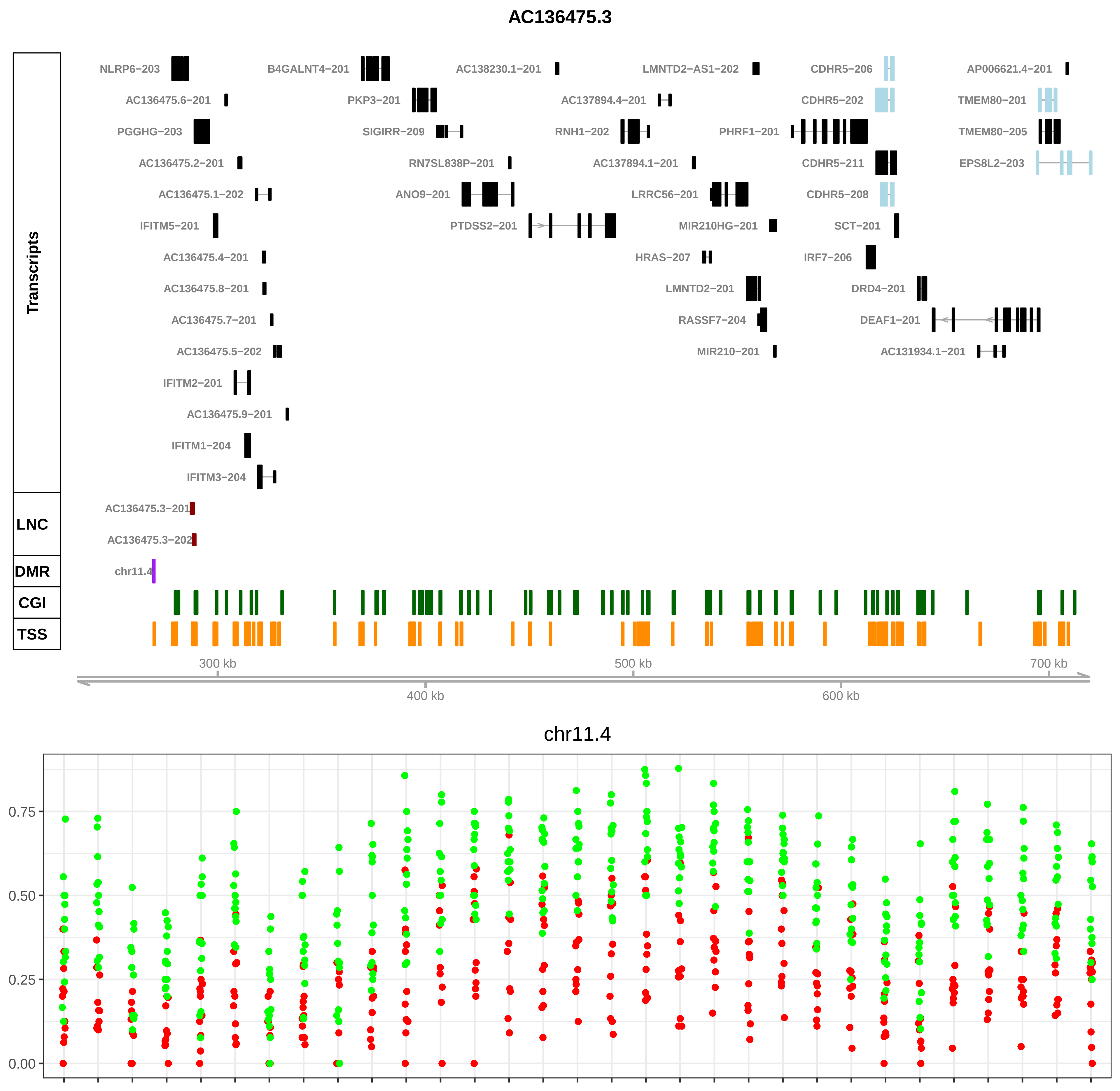

AC136475.3
Transcripts
NLRP6−203
B4GALNT4−201
AC138230.1−201
LMNTD2−AS1−202
CDHR5−206
AP006621.4−201
AC136475.6−201
PKP3−201
AC137894.4−201
CDHR5−202
TMEM80−201
PGGHG−203
SIGIRR−209
RNH1−202
PHRF1−201
TMEM80−205
AC136475.2−201
RN7SL838P−201
AC137894.1−201
CDHR5−211
EPS8L2−203
AC136475.1−202
ANO9−201
LRRC56−201
CDHR5−208
IFITM5−201
PTDSS2−201
MIR210HG−201
SCT−201
AC136475.4−201
HRAS−207
IRF7−206
AC136475.8−201
LMNTD2−201
DRD4−201
AC136475.7−201
RASSF7−204
DEAF1−201
AC136475.5−202
MIR210−201
AC131934.1−201
IFITM2−201
AC136475.9−201
IFITM1−204
IFITM3−204
LNC
AC136475.3−201
AC136475.3−202
DMR
chr11.4
CGI
TSS
300 kb
400 kb
500 kb
600 kb
700 kb
chr11.4
0.75
0.50
0.25
0.00

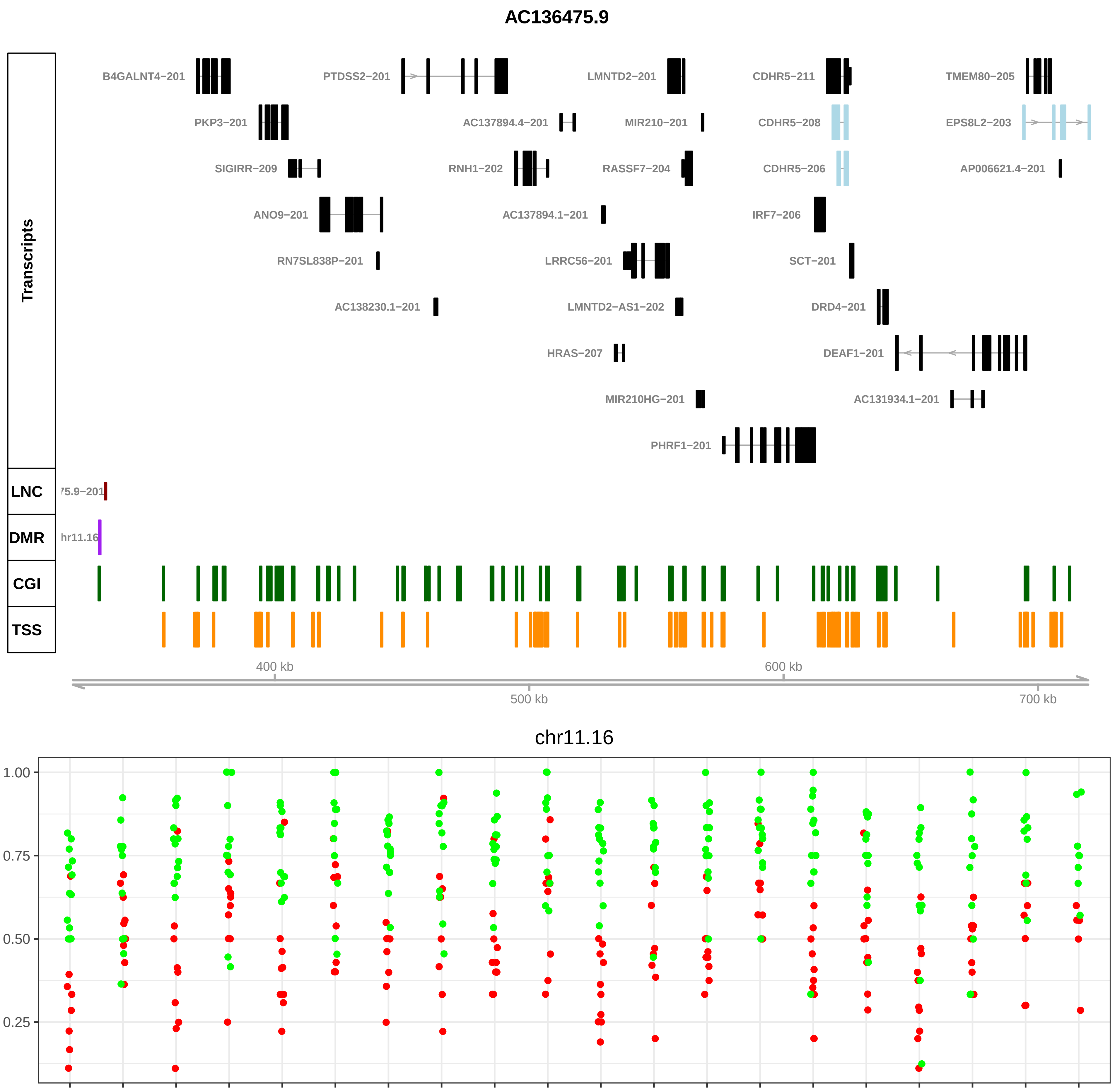
AC136475.9
Transcripts
B4GALNT4−201
PTDSS2−201
LMNTD2−201
CDHR5−211
TMEM80−205
PKP3−201
AC137894.4−201
MIR210−201
CDHR5−208
EPS8L2−203
SIGIRR−209
RNH1−202
RASSF7−204
CDHR5−206
AP006621.4−201
ANO9−201
AC137894.1−201
IRF7−206
RN7SL838P−201
LRRC56−201
SCT−201
AC138230.1−201
LMNTD2−AS1−202
DRD4−201
HRAS−207
DEAF1−201
MIR210HG−201
AC131934.1−201
PHRF1−201
LNC
75.9−201
DMR
hr11.16
CGI
TSS
400 kb
600 kb
500 kb
700 kb
chr11.16
1.00
0.75
0.50
0.25

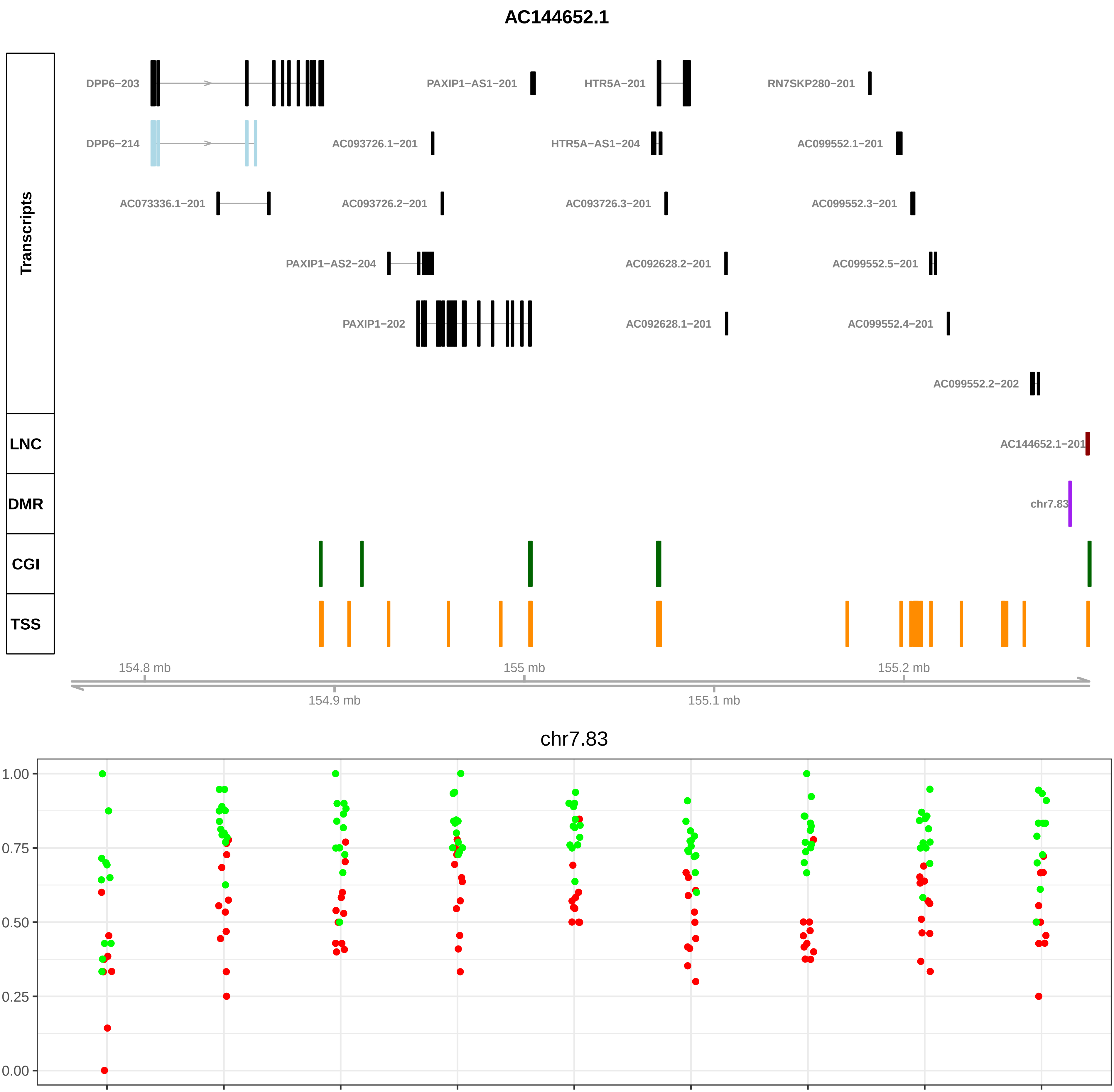

AC144652.1
Transcripts
DPP6−203
PAXIP1−AS1−201
HTR5A−201
RN7SKP280−201
DPP6−214
AC093726.1−201
HTR5A−AS1−204
AC099552.1−201
AC073336.1−201
AC093726.2−201
AC093726.3−201
AC099552.3−201
PAXIP1−AS2−204
AC092628.2−201
AC099552.5−201
PAXIP1−202
AC092628.1−201
AC099552.4−201
AC099552.2−202
LNC
AC144652.1−201
DMR
chr7.83
CGI
TSS
154.8 mb
155 mb
155.2 mb
154.9 mb
155.1 mb
chr7.83
1.00
0.75
0.50
0.25
0.00

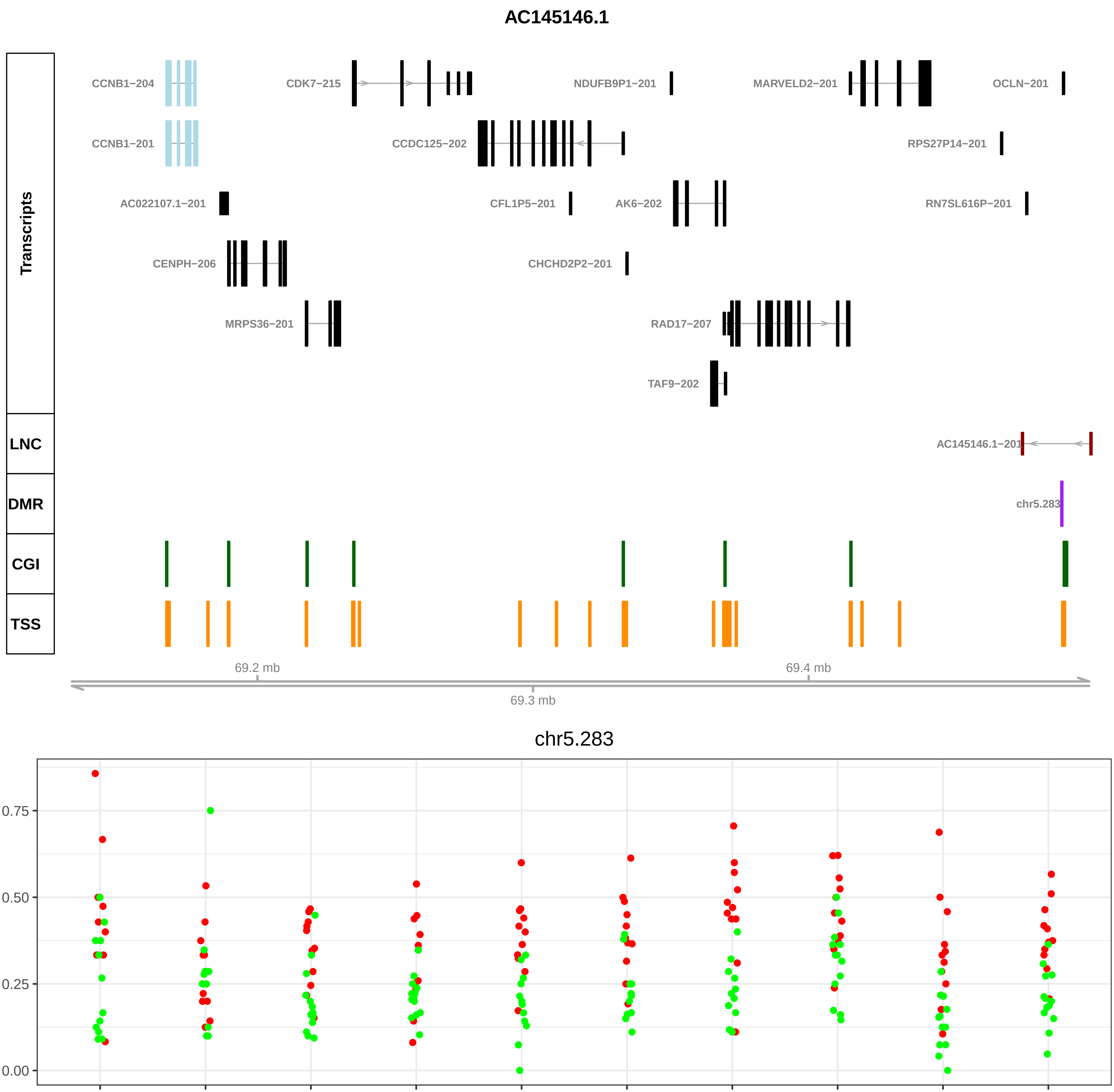
AC145146.1
Transcripts
CCNB1−204
CDK7−215
NDUFB9P1−201
MARVELD2−201
OCLN−201
CCNB1−201
CCDC125−202
RPS27P14−201
AC022107.1−201
CFL1P5−201
AK6−202
RN7SL616P−201
CENPH−206
CHCHD2P2−201
MRPS36−201
RAD17−207
TAF9−202
LNC
AC145146.1−201
DMR
chr5.283
CGI
TSS
69.2 mb
69.3 mb
69.4 mb
chr5.283
0.75
0.50
0.25
0.00

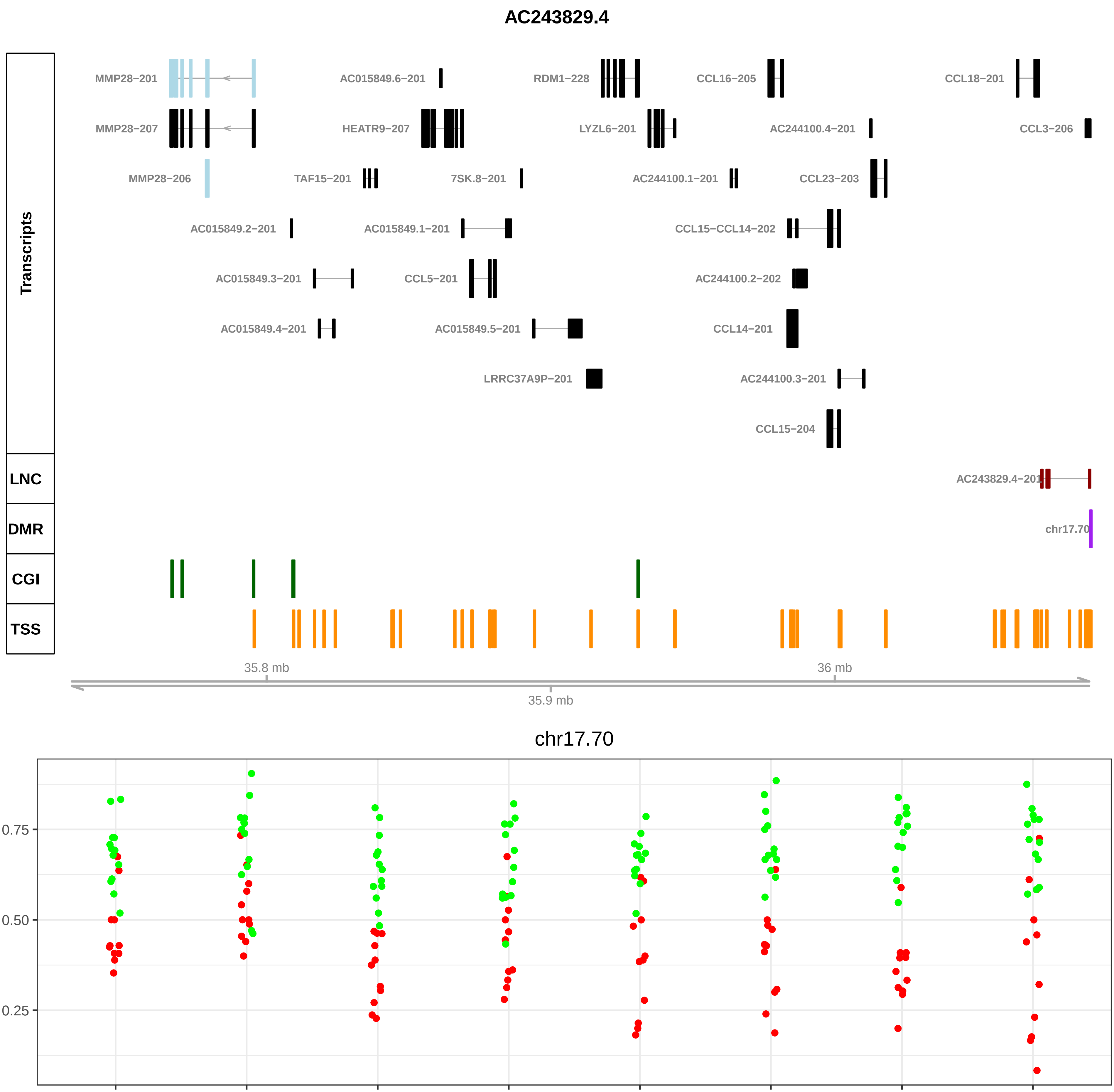

AC243829.4
Transcripts
MMP28−201
AC015849.6−201
RDM1−228
CCL16−205
CCL18−201
MMP28−207
HEATR9−207
LYZL6−201
AC244100.4−201
CCL3−206
MMP28−206
TAF15−201
7SK.8−201
AC244100.1−201
CCL23−203
AC015849.2−201
AC015849.1−201
CCL15−CCL14−202
AC015849.3−201
CCL5−201
AC244100.2−202
AC015849.4−201
AC015849.5−201
CCL14−201
LRRC37A9P−201
AC244100.3−201
CCL15−204
LNC
AC243829.4−201
DMR
chr17.70
CGI
TSS
35.8 mb
36 mb
35.9 mb
chr17.70
0.75
0.50
0.25

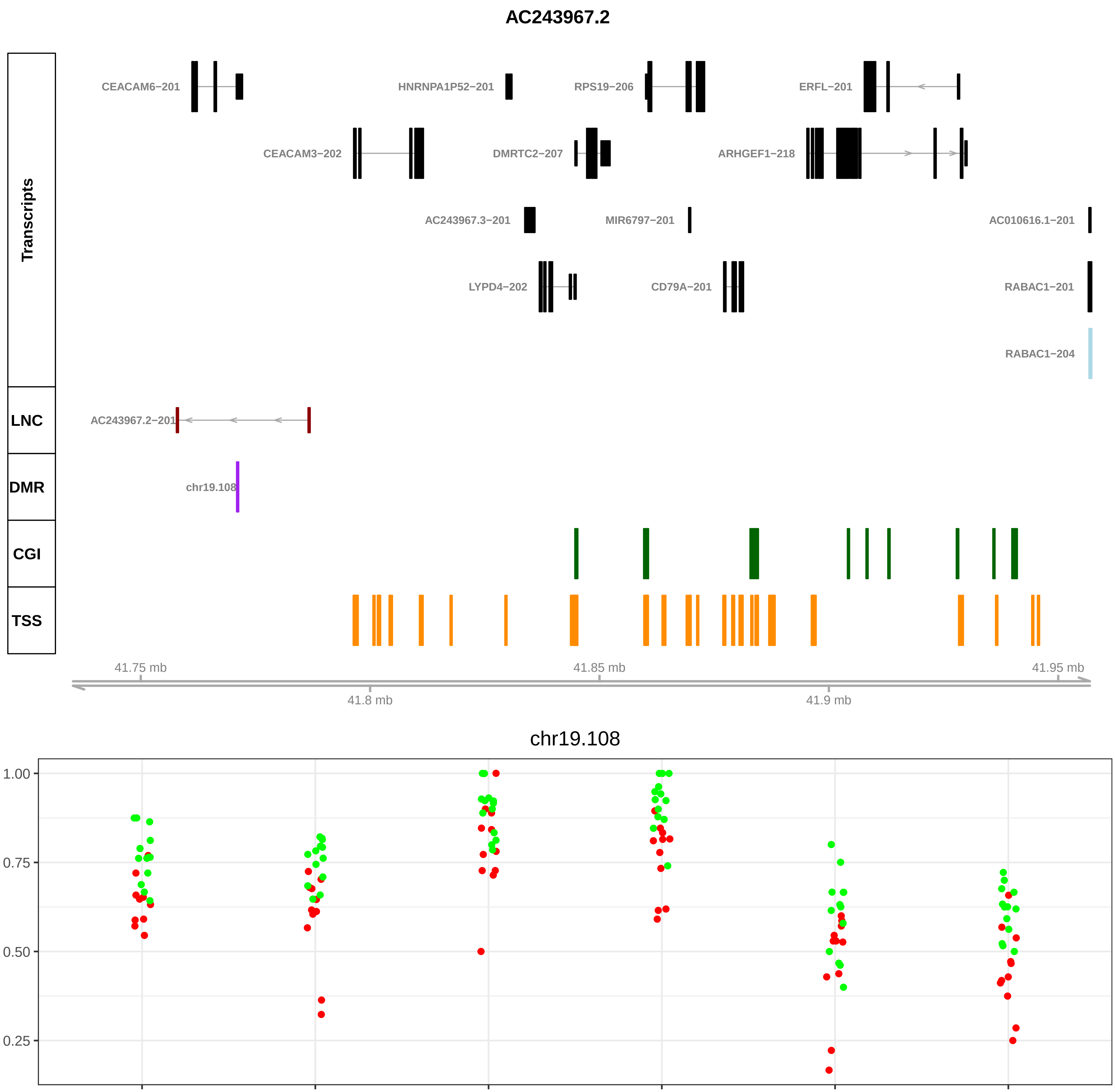
AC243967.2
Transcripts
LNC
DMR
CGI
TSS
CEACAM6−201
HNRNPA1P52−201
RPS19−206
ERFL−201
CEACAM3−202
DMRTC2−207
ARHGEF1−218
AC243967.3−201
MIR6797−201
AC010616.1−201
LYPD4−202
CD79A−201
RABAC1−201
RABAC1−204
AC243967.2−201
chr19.108
41.75 mb
41.85 mb
41.95 mb
41.8 mb
41.9 mb
chr19.108
1.00
0.75
0.50
0.25

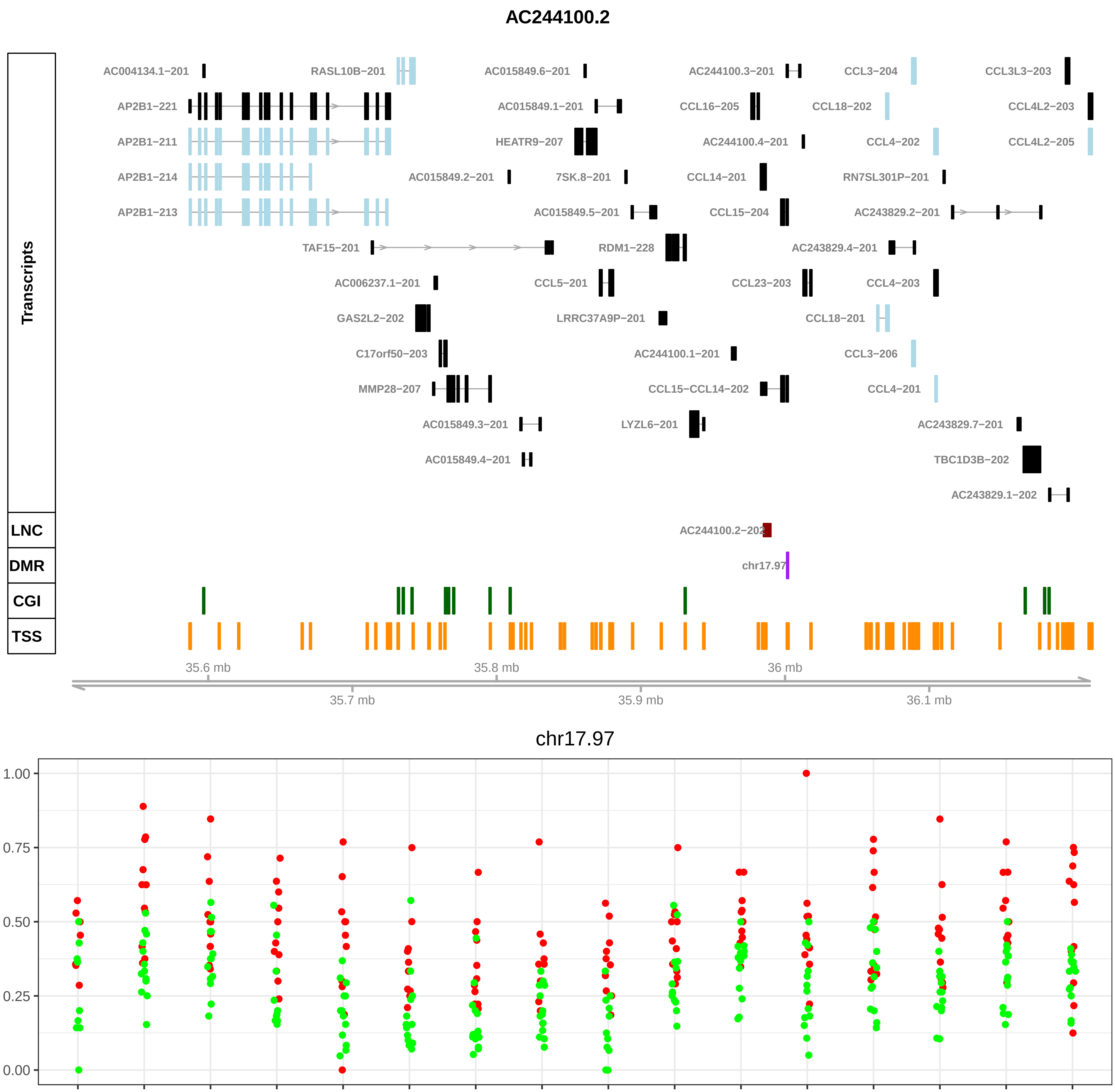
AC244100.2
Transcripts
LNC
DMR
CGI
TSS
AC004134.1−201
RASL10B−201
AC015849.6−201
AC244100.3−201
CCL3−204
CCL3L3−203
AP2B1−221
AC015849.1−201
CCL16−205
CCL18−202
CCL4L2−203
AP2B1−211
HEATR9−207
AC244100.4−201
CCL4−202
CCL4L2−205
AP2B1−214
AC015849.2−201
7SK.8−201
CCL14−201
RN7SL301P−201
AP2B1−213
AC015849.5−201
CCL15−204
AC243829.2−201
TAF15−201
RDM1−228
AC243829.4−201
AC006237.1−201
CCL5−201
CCL23−203
CCL4−203
GAS2L2−202
LRRC37A9P−201
CCL18−201
C17orf50−203
AC244100.1−201
CCL3−206
MMP28−207
CCL15−CCL14−202
CCL4−201
AC015849.3−201
LYZL6−201
AC243829.7−201
AC015849.4−201
TBC1D3B−202
AC243829.1−202
AC244100.2−202
chr17.97
35.6 mb
35.8 mb
36 mb
35.7 mb
35.9 mb
36.1 mb
chr17.97
1.00
0.75
0.50
0.25
0.00

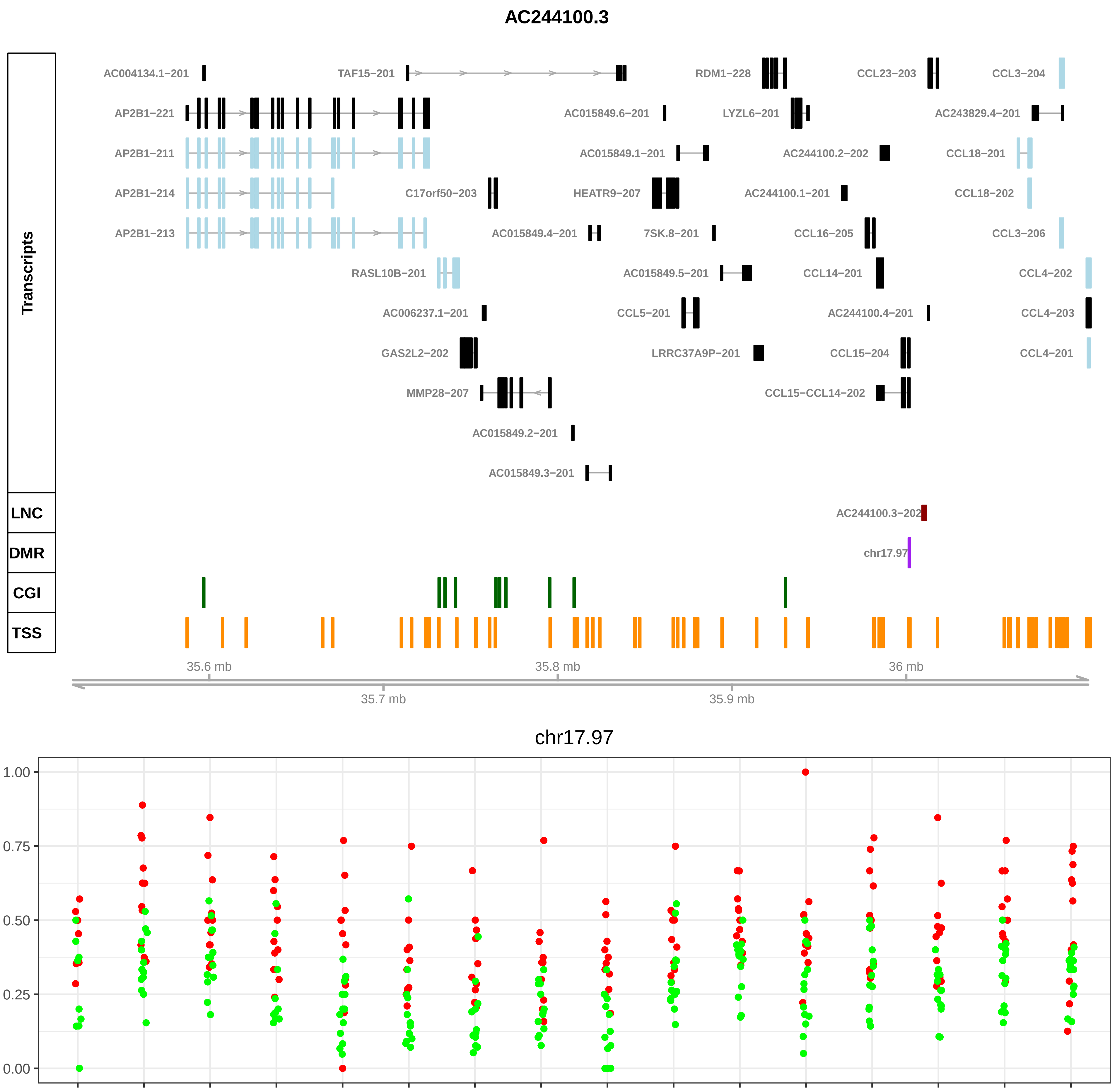
AC244100.3
Transcripts
AC004134.1−201
TAF15−201
RDM1−228
CCL23−203
CCL3−204
AP2B1−221
AC015849.6−201
LYZL6−201
AC243829.4−201
AP2B1−211
AC015849.1−201
AC244100.2−202
CCL18−201
AP2B1−214
C17orf50−203
HEATR9−207
AC244100.1−201
CCL18−202
AP2B1−213
AC015849.4−201
7SK.8−201
CCL16−205
CCL3−206
RASL10B−201
AC015849.5−201
CCL14−201
CCL4−202
AC006237.1−201
CCL5−201
AC244100.4−201
CCL4−203
GAS2L2−202
LRRC37A9P−201
CCL15−204
CCL4−201
MMP28−207
CCL15−CCL14−202
AC015849.2−201
AC015849.3−201
LNC
AC244100.3−202
DMR
chr17.97
CGI
TSS
35.6 mb
35.8 mb
36 mb
35.7 mb
35.9 mb
chr17.97
1.00
0.75
0.50
0.25
0.00

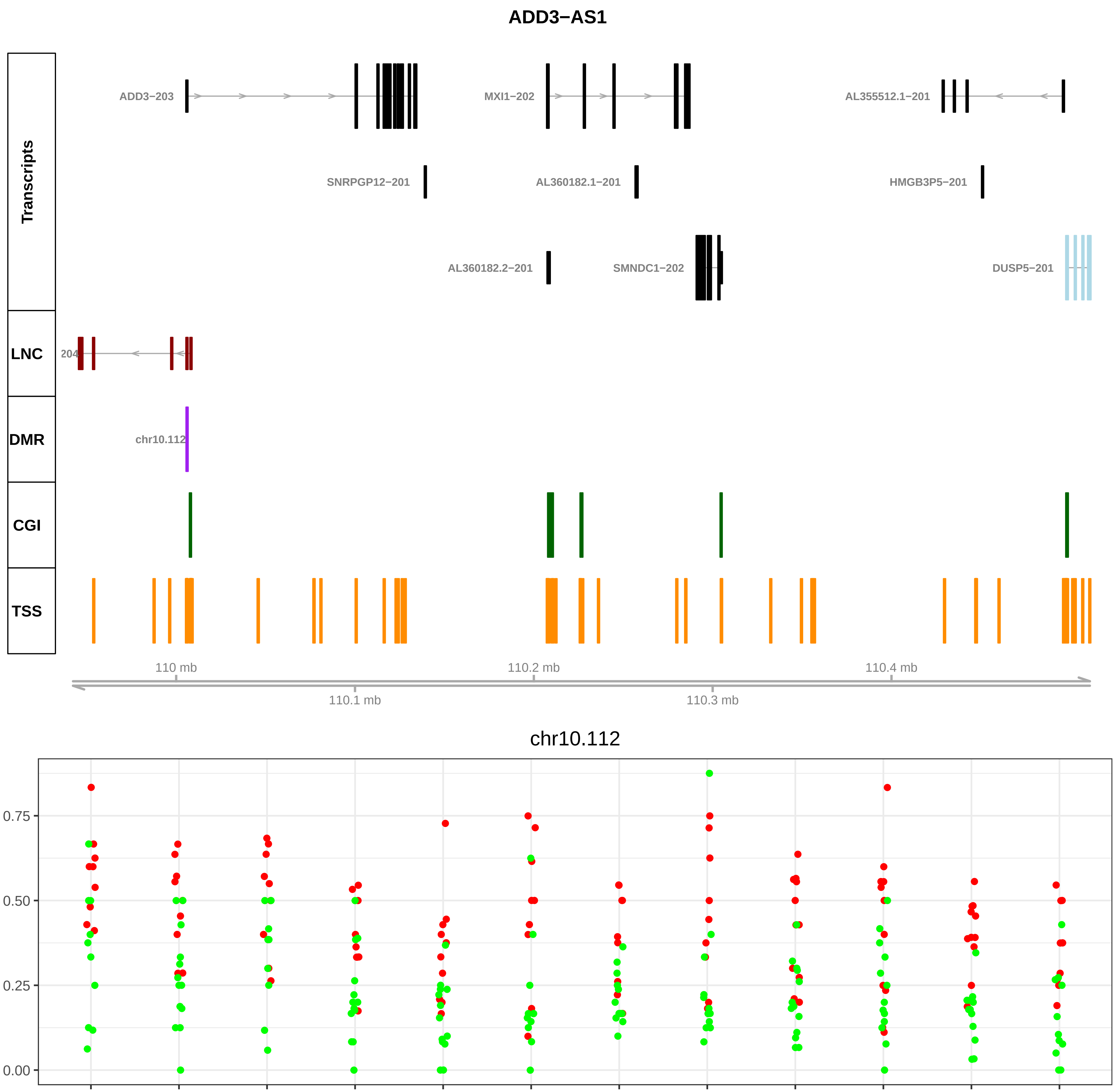
ADD3−AS1
Transcripts
ADD3−203
MXI1−202
AL355512.1−201
SNRPGP12−201
AL360182.1−201
HMGB3P5−201
AL360182.2−201
SMNDC1−202
DUSP5−201
LNC
204
DMR
chr10.112
CGI
TSS
110 mb
110.1 mb
110.2 mb
110.3 mb
110.4 mb
chr10.112
0.00
0.25
0.50
0.75

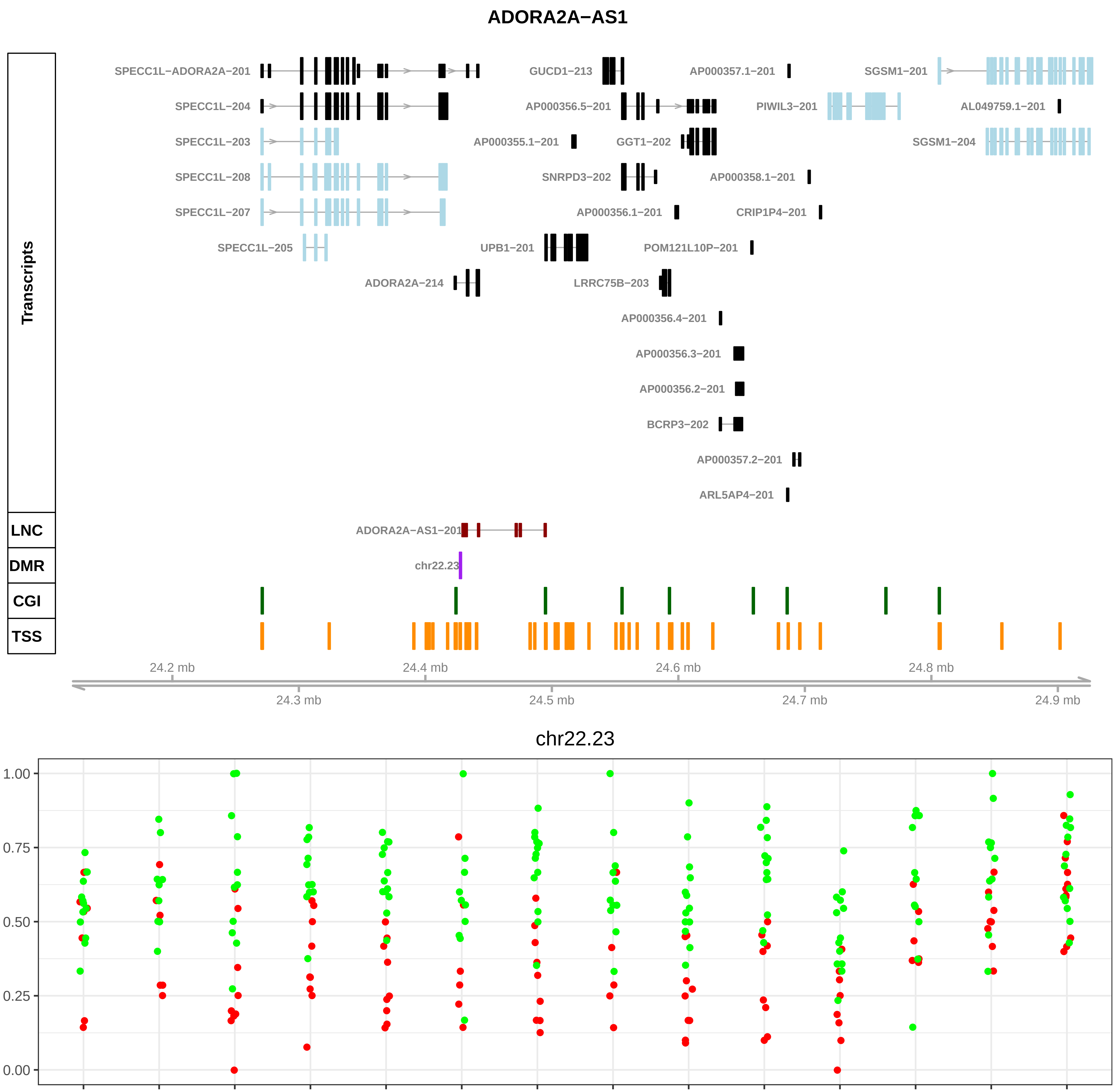

ADORA2A−AS1
Transcripts
SPECC1L−ADORA2A−201
SPECC1L−204
SPECC1L−203
SPECC1L−208
SPECC1L−207
SPECC1L−205
ADORA2A−214
GUCD1−213
AP000356.5−201
AP000355.1−201
GGT1−202
SNRPD3−202
AP000356.1−201
UPB1−201
LRRC75B−203
AP000356.4−201
AP000356.3−201
AP000356.2−201
BCRP3−202
AP000357.2−201
ARL5AP4−201
AP000357.1−201
PIWIL3−201
AP000358.1−201
CRIP1P4−201
POM121L10P−201
SGSM1−201
AL049759.1−201
SGSM1−204
LNC
ADORA2A−AS1−201
DMR
chr22.23
CGI
TSS
24.2 mb
24.4 mb
24.6 mb
24.8 mb
24.3 mb
24.5 mb
24.7 mb
24.9 mb
chr22.23
1.00
0.75
0.50
0.25
0.00

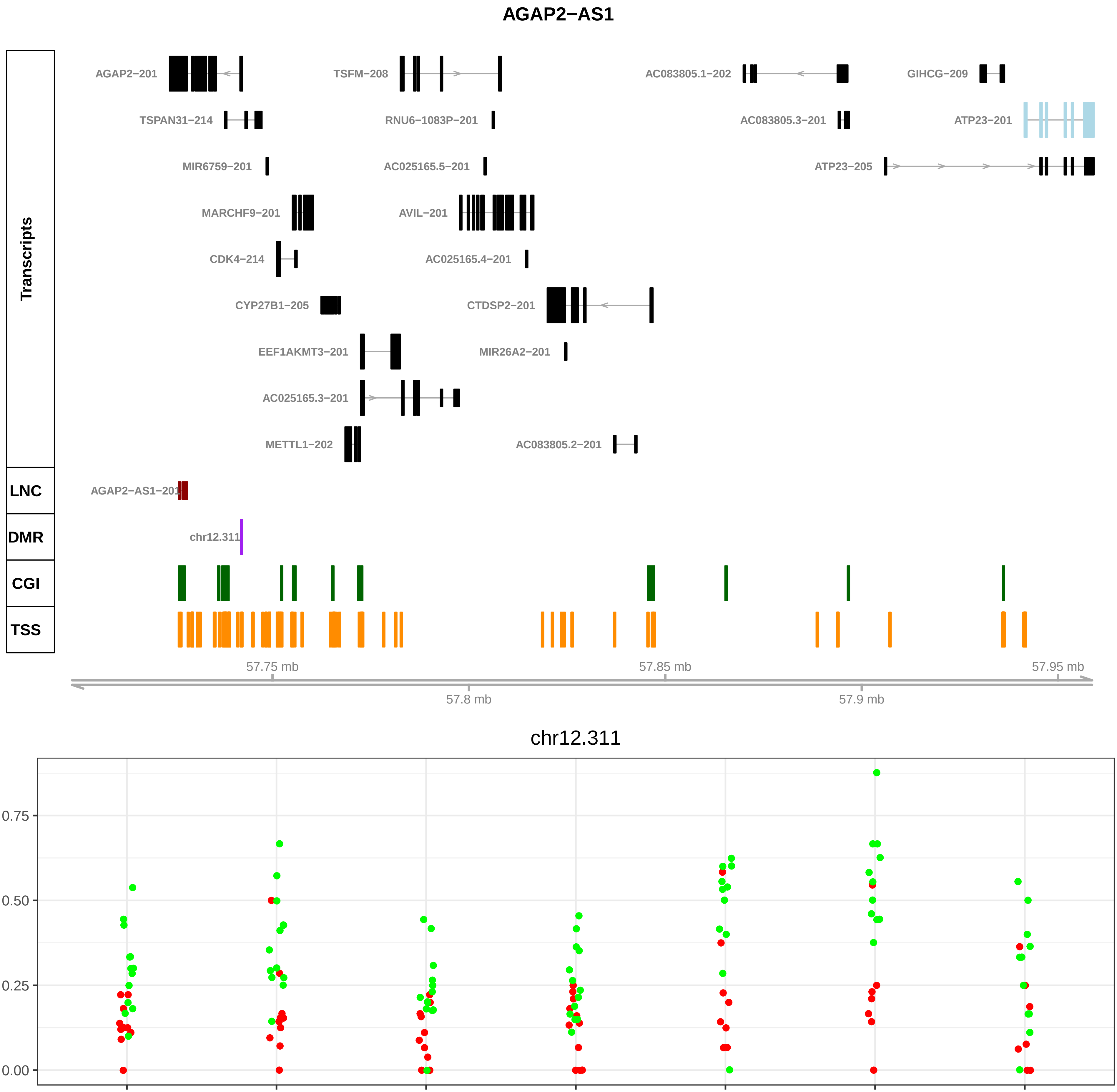
AGAP2−AS1
Transcripts
AGAP2−201
TSFM−208
AC083805.1−202
GIHCG−209
TSPAN31−214
RNU6−1083P−201
AC083805.3−201
ATP23−201
MIR6759−201
AC025165.5−201
ATP23−205
MARCHF9−201
AVIL−201
CDK4−214
AC025165.4−201
CYP27B1−205
CTDSP2−201
EEF1AKMT3−201
MIR26A2−201
AC025165.3−201
METTL1−202
AC083805.2−201
LNC
AGAP2−AS1−201
DMR
chr12.311
CGI
TSS
57.75 mb
57.85 mb
57.95 mb
57.8 mb
57.9 mb
chr12.311
0.75
0.50
0.25
0.00

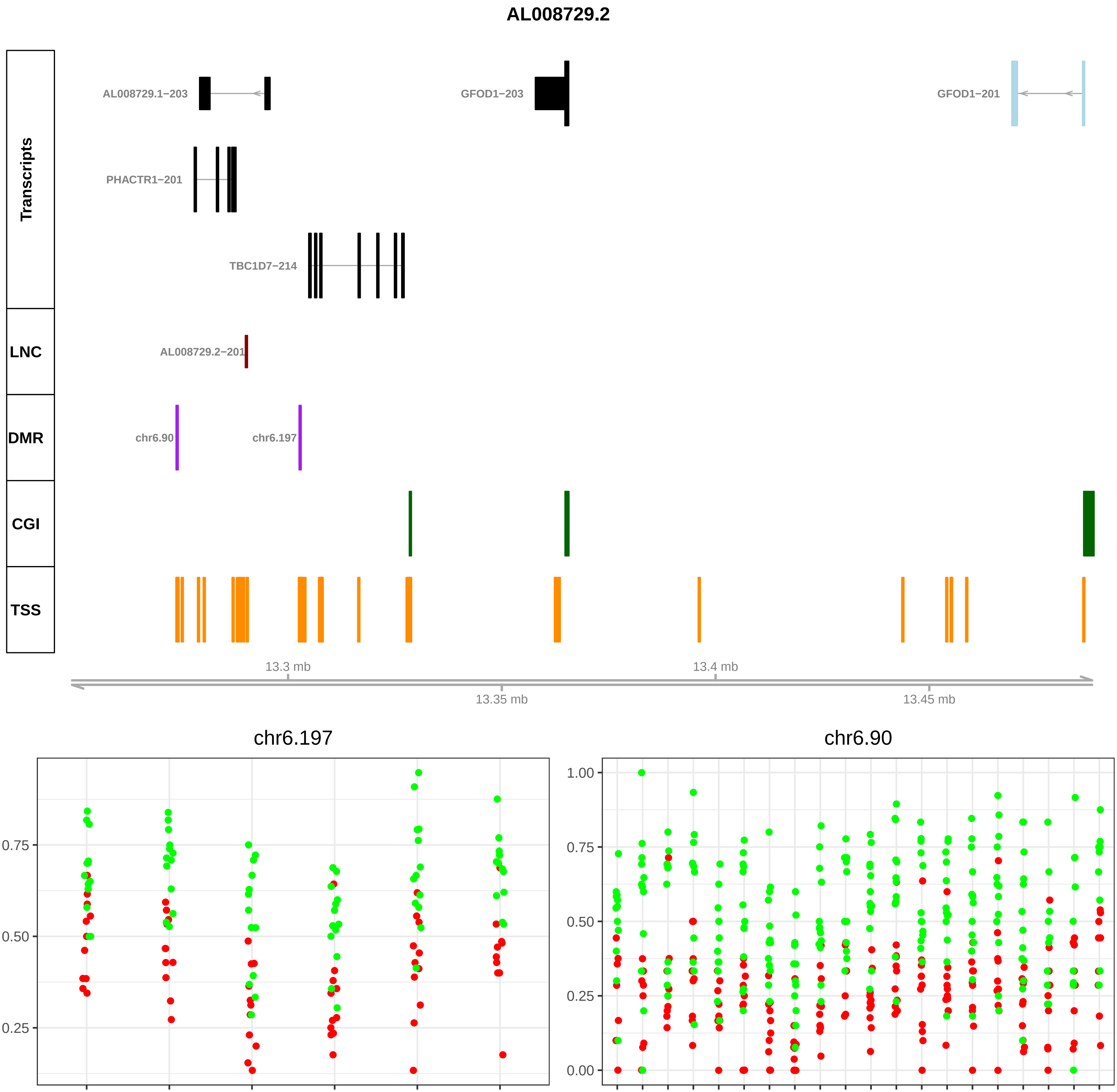
AL008729.2
Transcripts
LNC
DMR
CGI
TSS
AL008729.1−203
GFOD1−203
GFOD1−201
PHACTR1−201
TBC1D7−214
AL008729.2−201
chr6.90
chr6.197
13.3 mb
13.4 mb
13.35 mb
13.45 mb
chr6.197
chr6.90
0.75
0.50
0.25
1.00
0.75
0.50
0.25
0.00

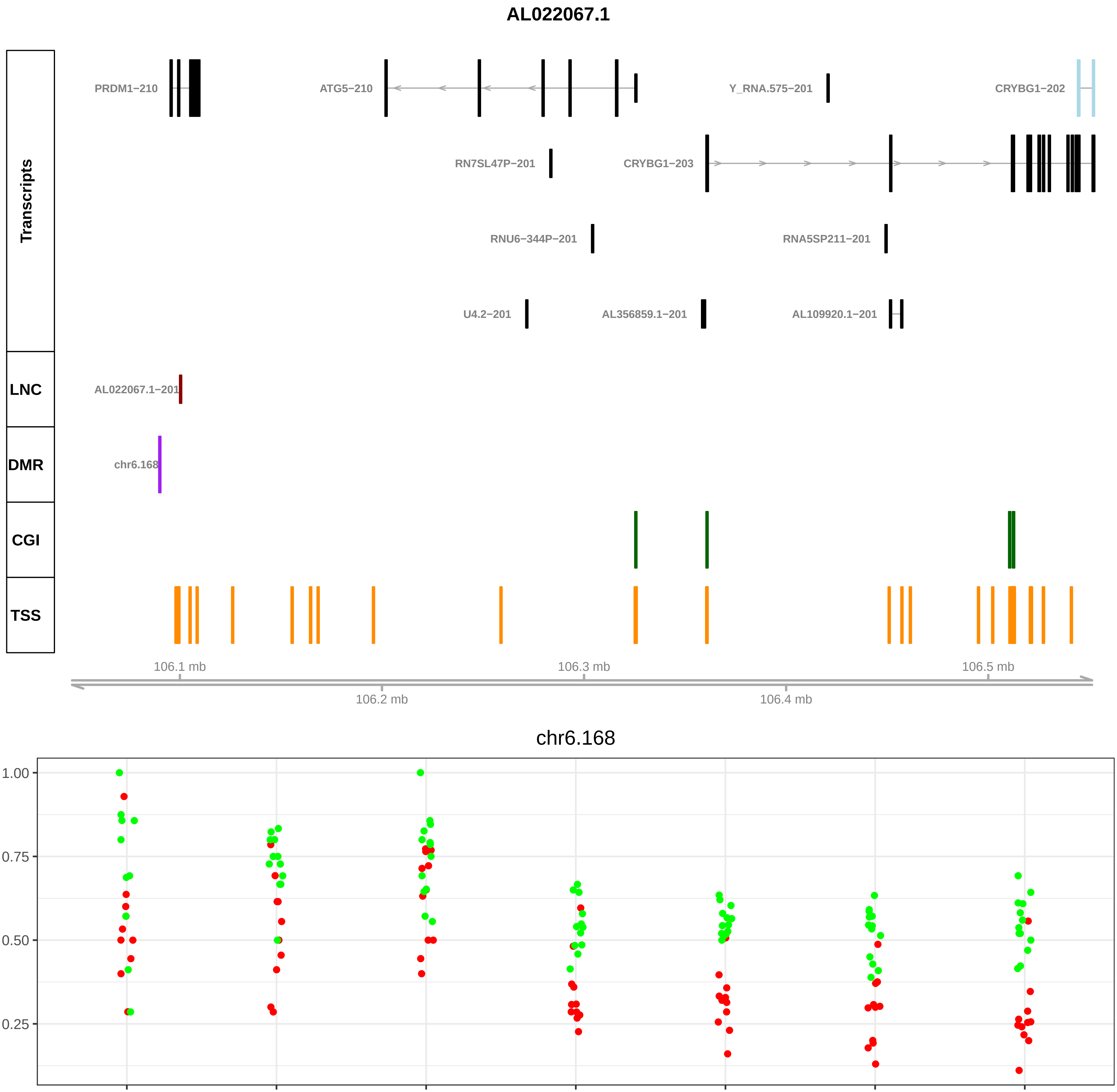
AL022067.1
Transcripts
PRDM1−210
ATG5−210
Y_RNA.575−201
CRYBG1−202
RN7SL47P−201
CRYBG1−203
RNU6−344P−201
RNA5SP211−201
U4.2−201
AL356859.1−201
AL109920.1−201
LNC
AL022067.1−201
DMR
chr6.168
CGI
TSS
106.1 mb
106.3 mb
106.5 mb
106.2 mb
106.4 mb
chr6.168
1.00
0.75
0.50
0.25

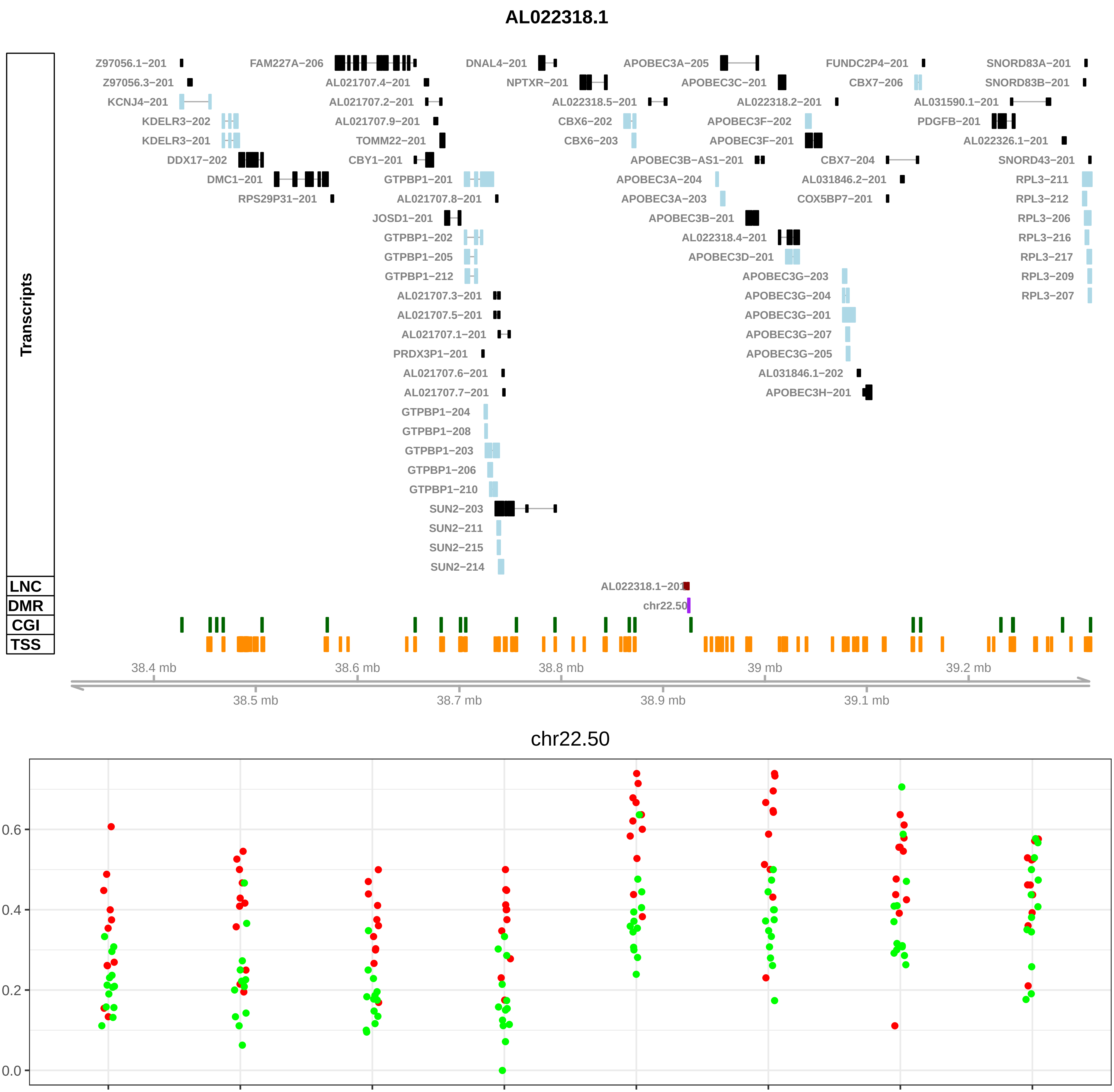
AL022318.1
Transcripts
LNC
DMR
CGI
TSS
AL022318.1-201
chr22.50
38.4 mb
38.5 mb
38.6 mb
38.7 mb
38.8 mb
38.9 mb
39 mb
39.1 mb
39.2 mb
chr22.50
0.0
0.2
0.4
0.6

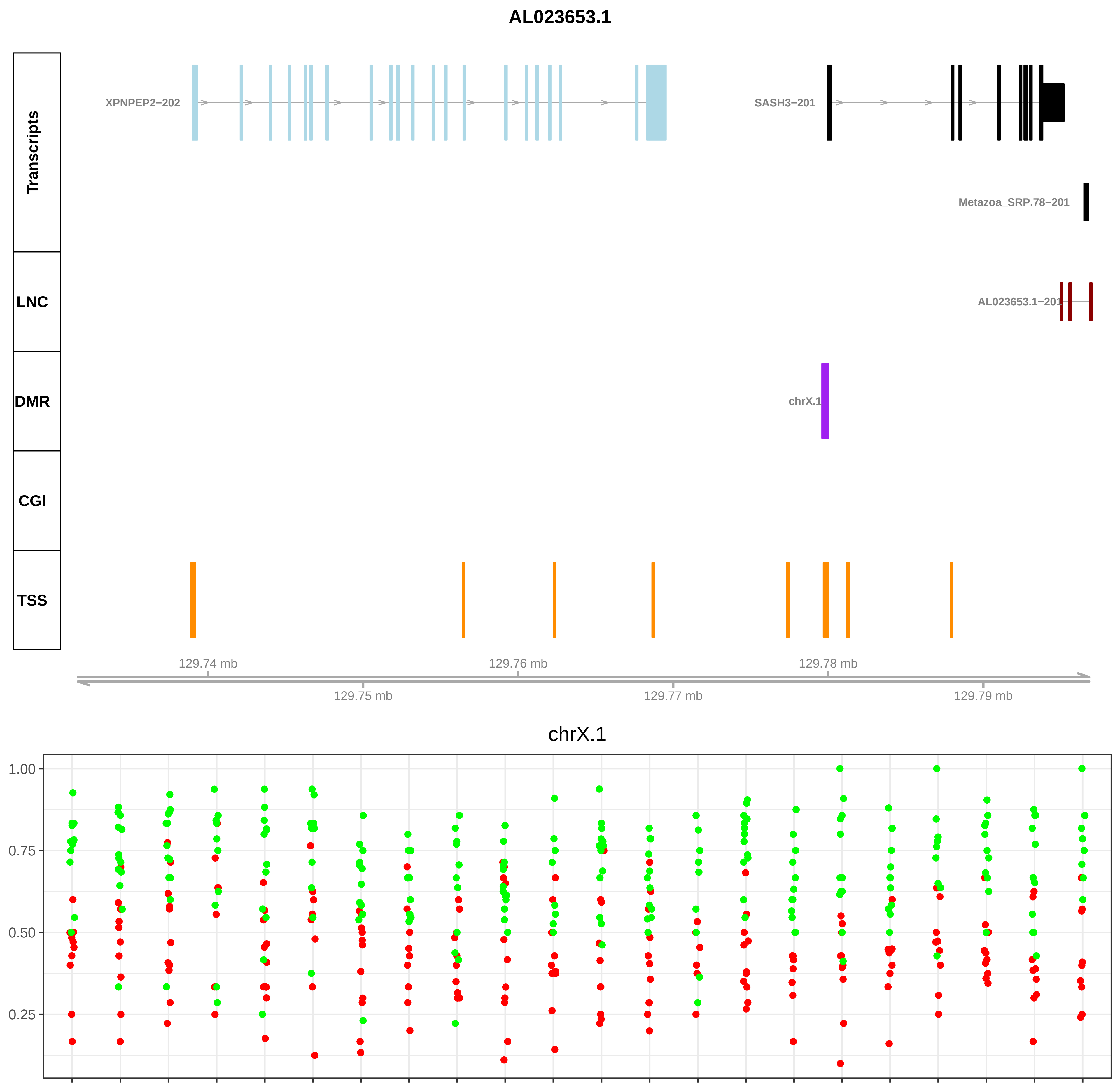
AL023653.1
Transcripts
XPNPEP2−202
SASH3−201
Metazoa_SRP.78−201
LNC
AL023653.1−201
DMR
chrX.1
CGI
TSS
129.74 mb
129.76 mb
129.78 mb
129.75 mb
129.77 mb
129.79 mb
chrX.1
1.00
0.75
0.50
0.25

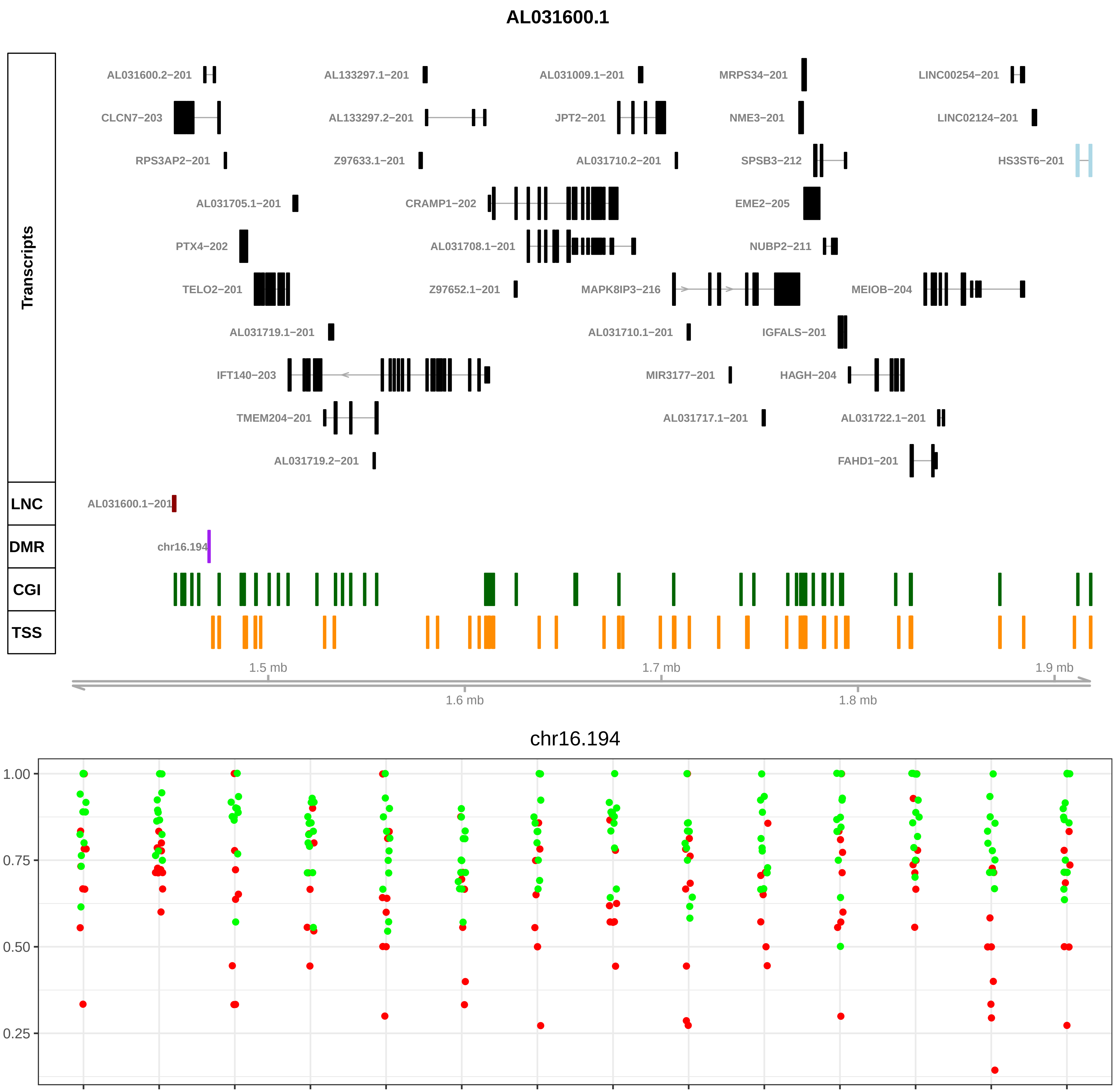
AL031600.1
Transcripts
AL031600.2−201
AL133297.1−201
AL031009.1−201
MRPS34−201
LINC00254−201
CLCN7−203
AL133297.2−201
JPT2−201
NME3−201
LINC02124−201
RPS3AP2−201
Z97633.1−201
AL031710.2−201
SPSB3−212
HS3ST6−201
AL031705.1−201
CRAMP1−202
EME2−205
PTX4−202
AL031708.1−201
NUBP2−211
TELO2−201
Z97652.1−201
MAPK8IP3−216
MEIOB−204
AL031719.1−201
AL031710.1−201
IGFALS−201
IFT140−203
MIR3177−201
HAGH−204
TMEM204−201
AL031717.1−201
AL031722.1−201
AL031719.2−201
FAHD1−201
LNC
AL031600.1−201
DMR
chr16.194
CGI
TSS
1.5 mb
1.6 mb
1.7 mb
1.8 mb
1.9 mb
chr16.194
1.00
0.75
0.50
0.25

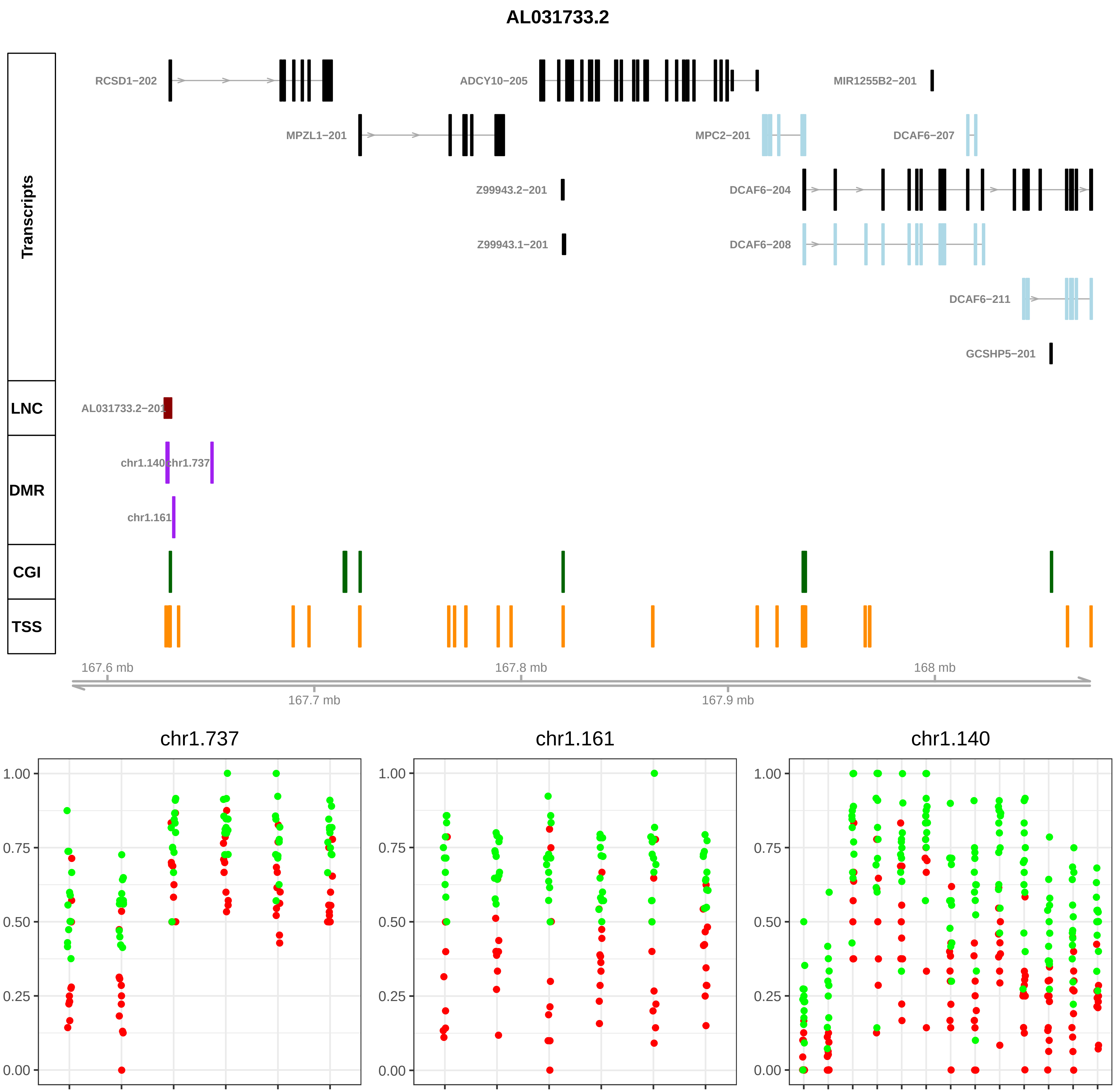

AL031733.2
Transcripts
RCSD1−202
ADCY10−205
MIR1255B2−201
MPZL1−201
MPC2−201
DCAF6−207
Z99943.2−201
DCAF6−204
Z99943.1−201
DCAF6−208
DCAF6−211
GCSHP5−201
LNC
AL031733.2−201
DMR
chr1.140
chr1.737
chr1.161
CGI
TSS
167.6 mb
167.7 mb
167.8 mb
167.9 mb
168 mb
chr1.737
chr1.161
chr1.140
1.00
0.75
0.50
0.25
0.00

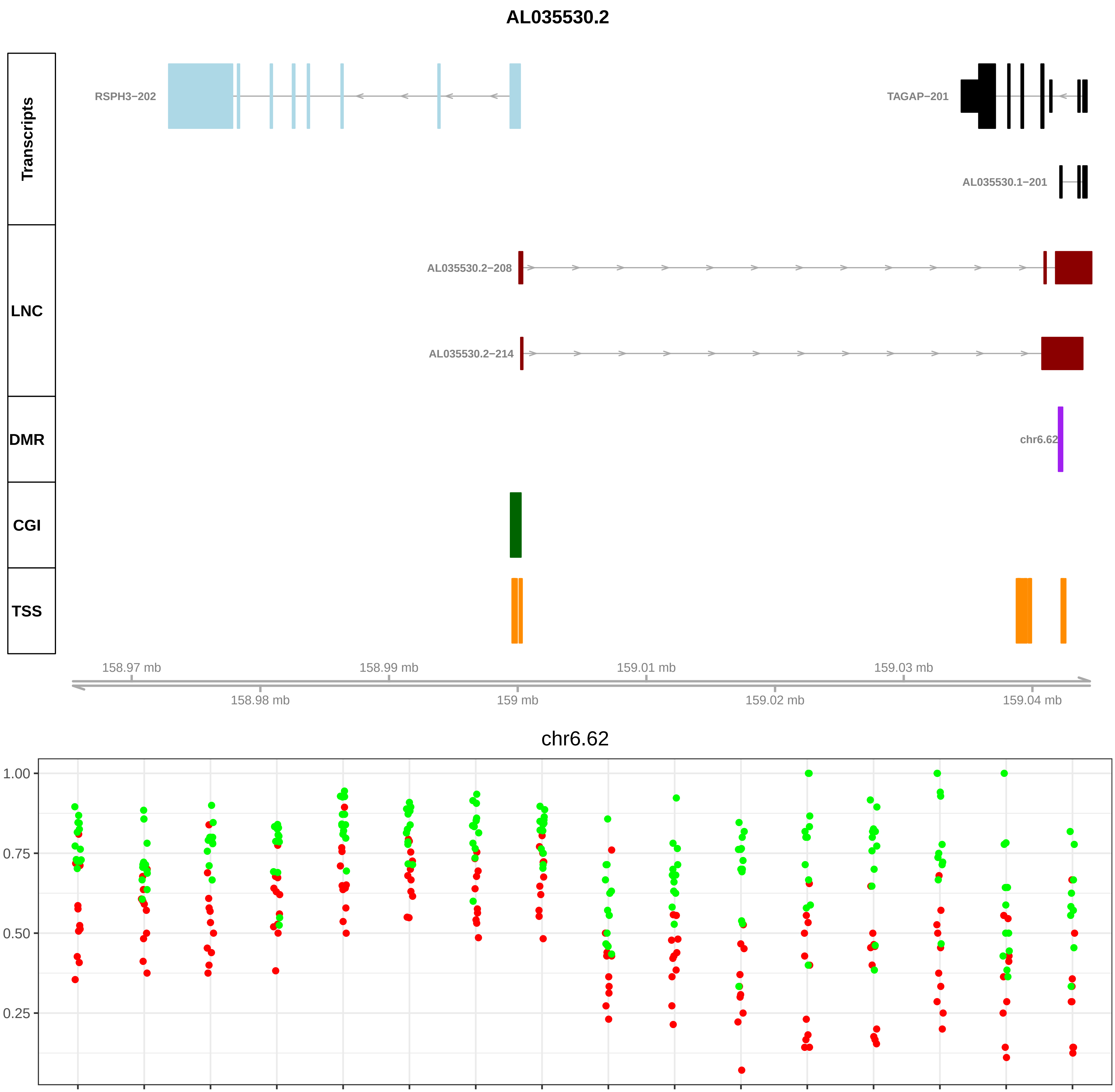

AL035530.2
Transcripts
LNC
DMR
CGI
TSS
RSPH3−202
TAGAP−201
AL035530.1−201
AL035530.2−208
AL035530.2−214
chr6.62
158.97 mb
158.98 mb
158.99 mb
159 mb
159.01 mb
159.02 mb
159.03 mb
159.04 mb
chr6.62
1.00
0.75
0.50
0.25

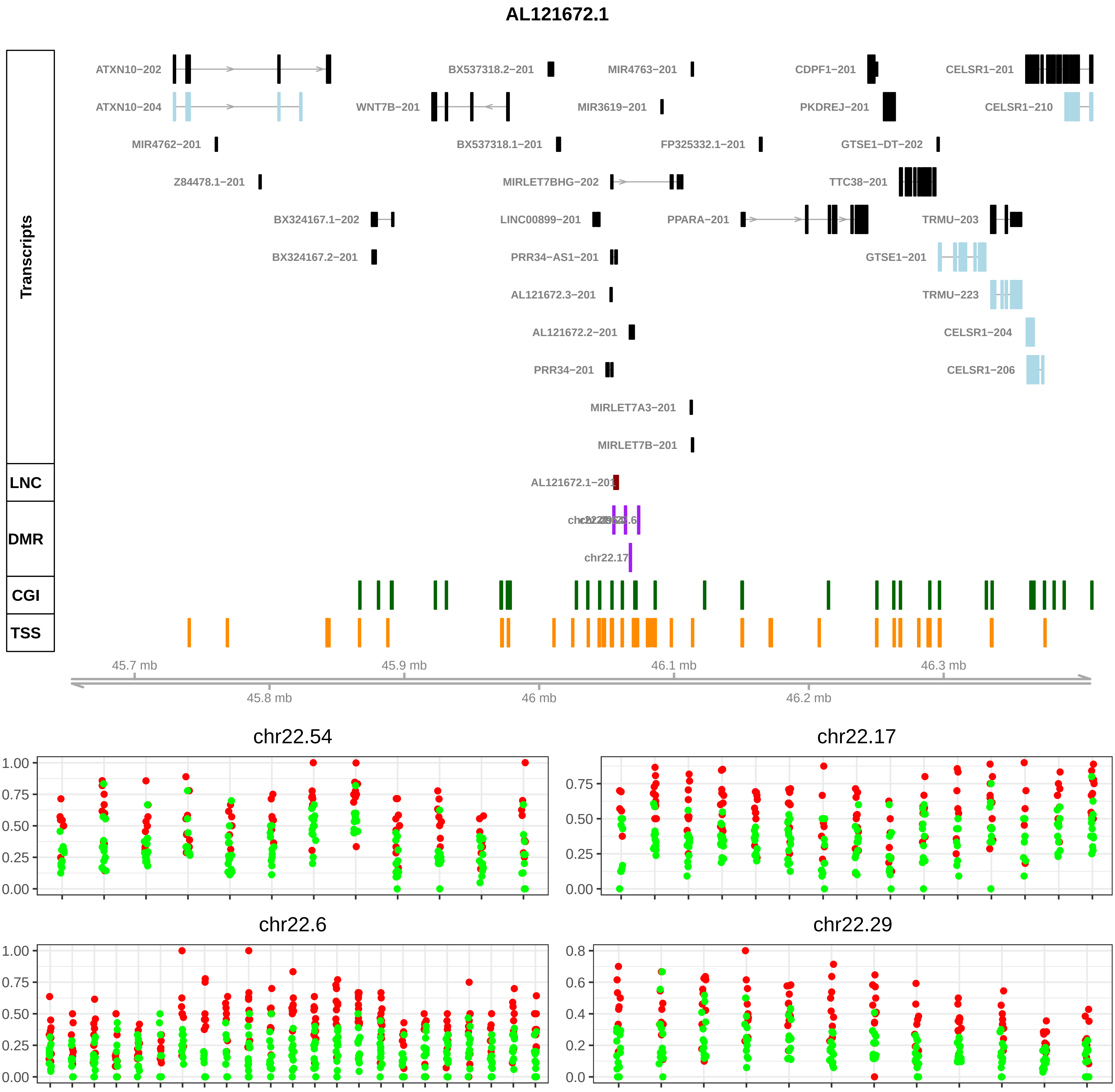
AL121672.1
Transcripts
LNC
DMR
CGI
TSS
ATXN10−202
ATXN10−204
MIR4762−201
Z84478.1−201
BX324167.1−202
BX324167.2−201
BX537318.2−201
WNT7B−201
BX537318.1−201
MIRLET7BHG−202
LINC00899−201
PRR34−AS1−201
AL121672.3−201
AL121672.2−201
PRR34−201
MIRLET7A3−201
MIRLET7B−201
MIR4763−201
MIR3619−201
FP325332.1−201
PPARA−201
CDPF1−201
PKDREJ−201
GTSE1−DT−202
TTC38−201
GTSE1−201
CELSR1−201
CELSR1−210
TRMU−203
TRMU−223
CELSR1−204
CELSR1−206
AL121672.1−201
chr22.17
45.7 mb
45.8 mb
45.9 mb
46 mb
46.1 mb
46.2 mb
46.3 mb
chr22.54
chr22.17
chr22.6
chr22.29
1.00
0.75
0.50
0.25
0.00
0.8
0.6
0.4
0.2
0.0

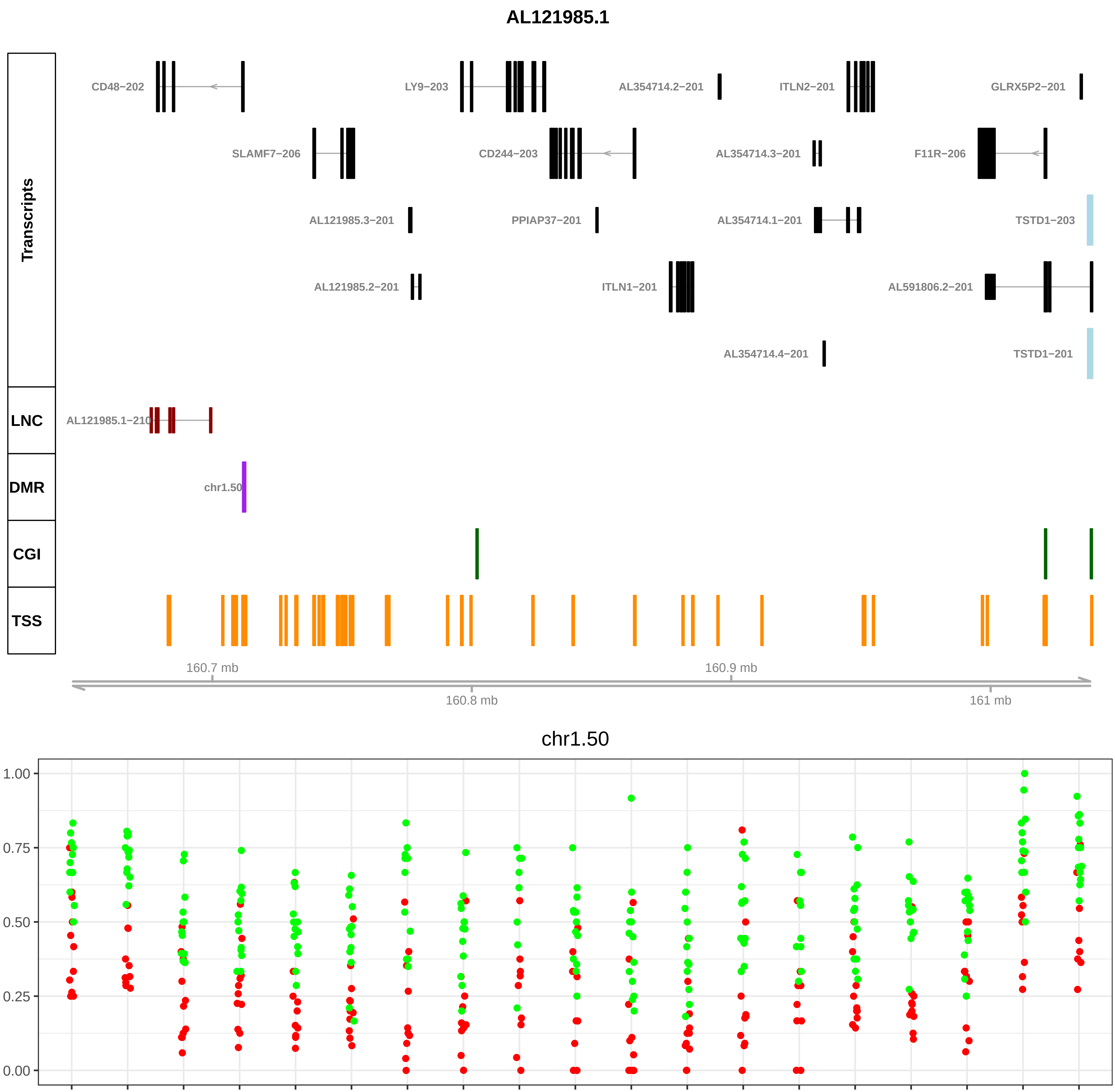
AL121985.1
Transcripts
CD48−202
LY9−203
AL354714.2−201
ITLN2−201
GLRX5P2−201
SLAMF7−206
CD244−203
AL354714.3−201
F11R−206
AL121985.3−201
PPIAP37−201
AL354714.1−201
TSTD1−203
AL121985.2−201
ITLN1−201
AL591806.2−201
AL354714.4−201
TSTD1−201
LNC
AL121985.1−210
DMR
chr1.50
CGI
TSS
160.7 mb
160.9 mb
160.8 mb
161 mb
chr1.50
1.00
0.75
0.50
0.25
0.00

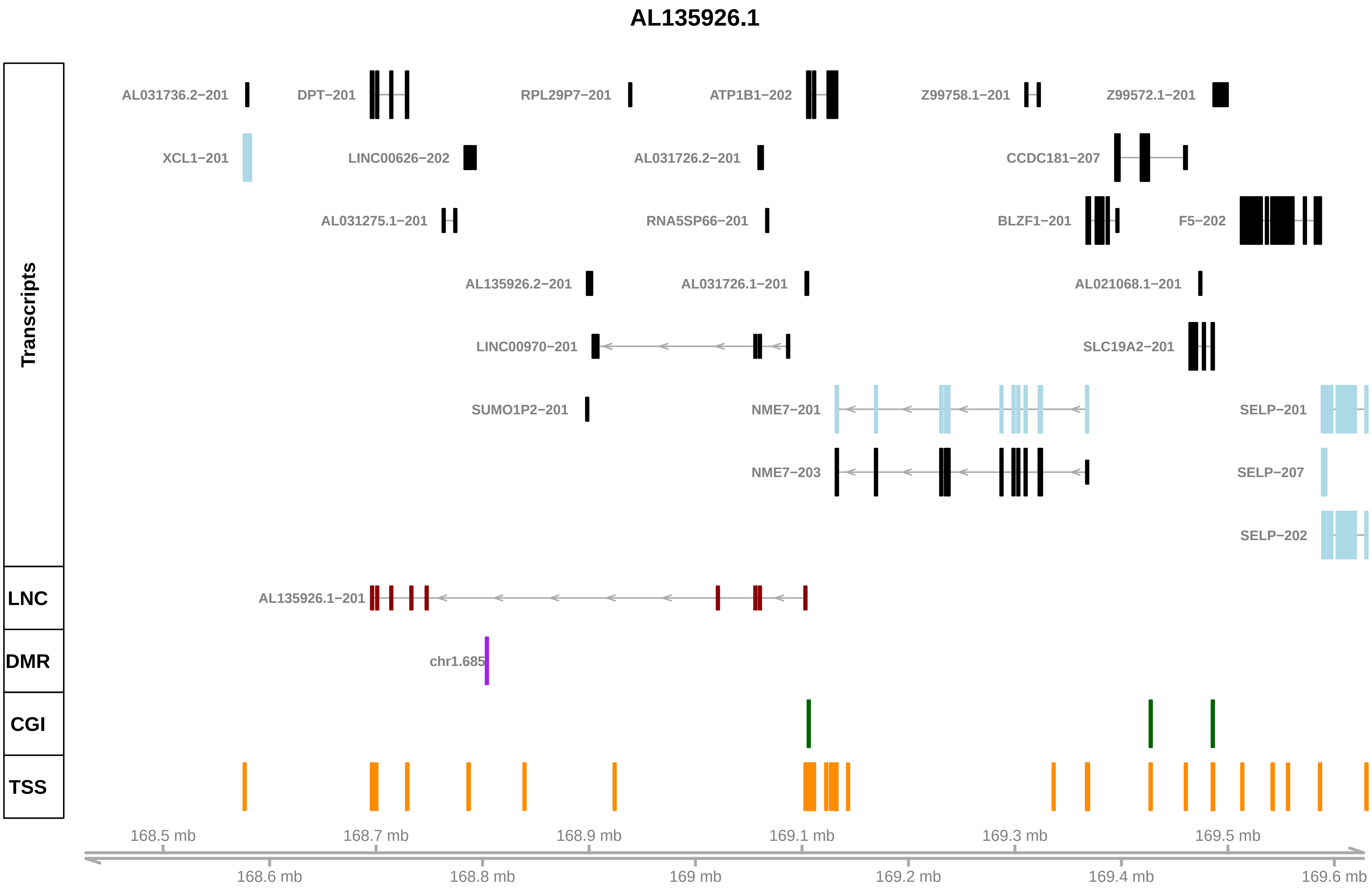

AL135926.1
Transcripts
AL031736.2−201
DPT−201
RPL29P7−201
ATP1B1−202
Z99758.1−201
Z99572.1−201
XCL1−201
LINC00626−202
AL031726.2−201
CCDC181−207
AL031275.1−201
RNA5SP66−201
BLZF1−201
F5−202
AL135926.2−201
AL031726.1−201
AL021068.1−201
LINC00970−201
SLC19A2−201
SUMO1P2−201
NME7−201
SELP−201
NME7−203
SELP−207
SELP−202
LNC
AL135926.1−201
DMR
chr1.685
CGI
TSS
168.5 mb
168.7 mb
168.9 mb
169.1 mb
169.3 mb
169.5 mb
168.6 mb
168.8 mb
169 mb
169.2 mb
169.4 mb
169.6 mb

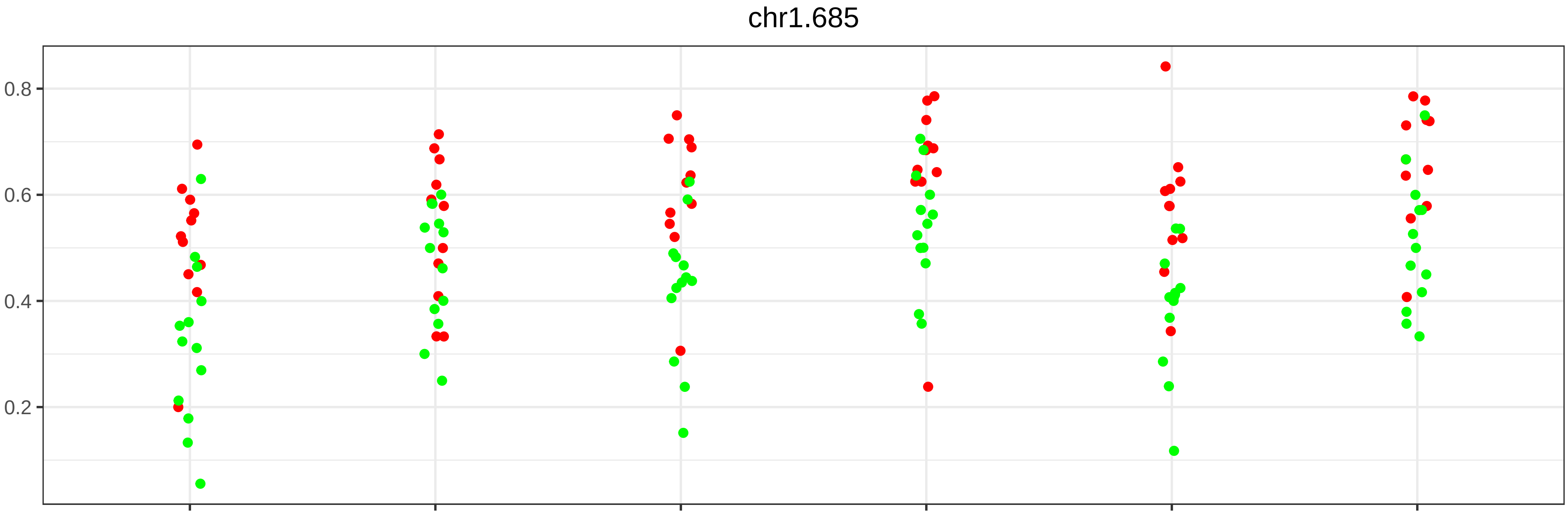

chr1.685
0.8
0.6
0.4
0.2

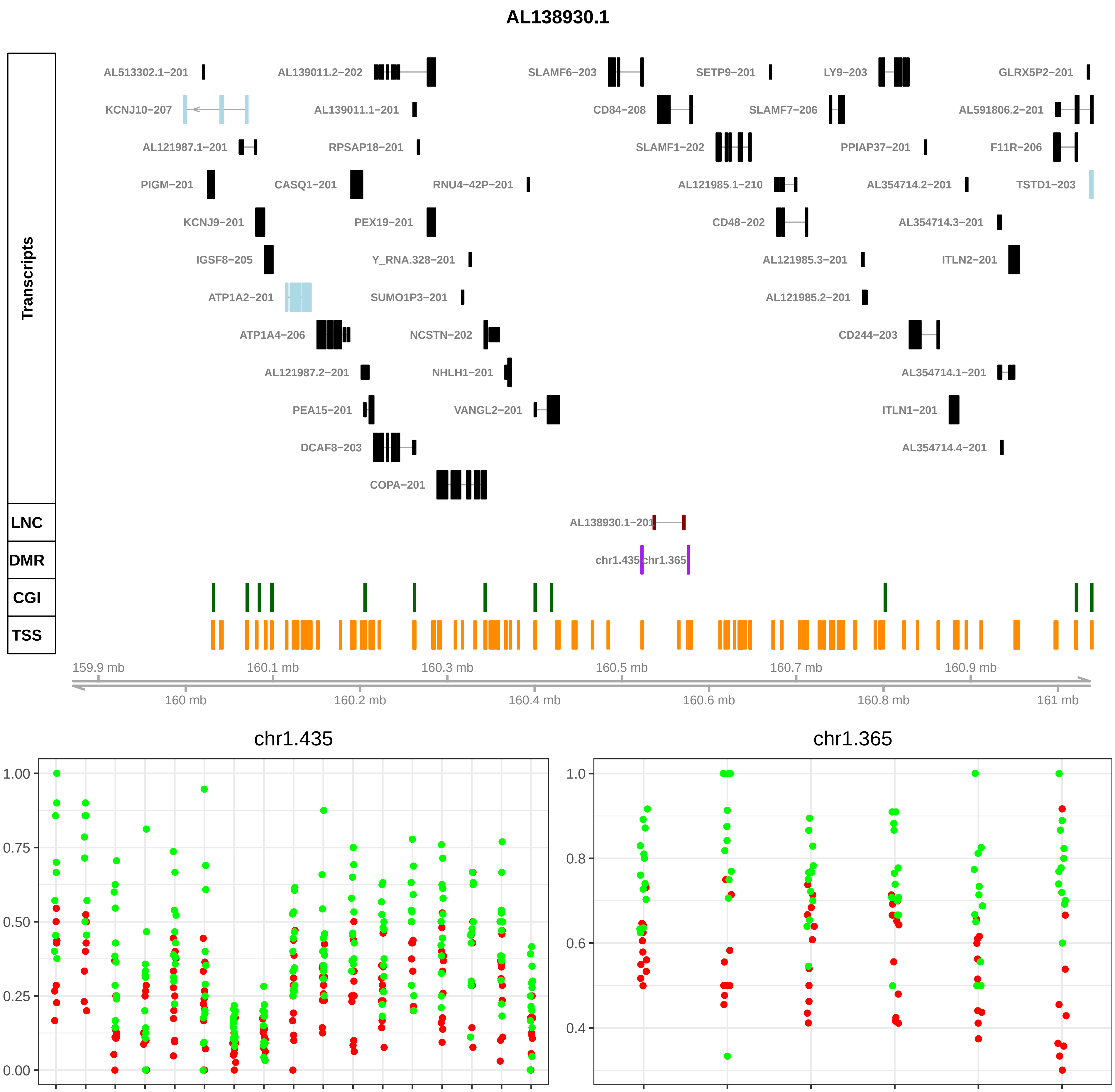

AL138930.1
Transcripts
AL513302.1−201
AL139011.2−202
SLAMF6−203
SETP9−201
LY9−203
GLRX5P2−201
KCNJ10−207
AL139011.1−201
CD84−208
SLAMF7−206
AL591806.2−201
AL121987.1−201
RPSAP18−201
SLAMF1−202
PPIAP37−201
F11R−206
PIGM−201
CASQ1−201
RNU4−42P−201
AL121985.1−210
AL354714.2−201
TSTD1−203
KCNJ9−201
PEX19−201
CD48−202
AL354714.3−201
IGSF8−205
Y_RNA.328−201
AL121985.3−201
ITLN2−201
ATP1A2−201
SUMO1P3−201
AL121985.2−201
ATP1A4−206
NCSTN−202
CD244−203
AL121987.2−201
NHLH1−201
AL354714.1−201
PEA15−201
VANGL2−201
ITLN1−201
DCAF8−203
AL354714.4−201
COPA−201
LNC
AL138930.1−201
DMR
chr1.435
chr1.365
CGI
TSS
159.9 mb
160.1 mb
160.3 mb
160.5 mb
160.7 mb
160.9 mb
160 mb
160.2 mb
160.4 mb
160.6 mb
160.8 mb
161 mb
chr1.435
1.00
0.75
0.50
0.25
0.00
chr1.365
1.0
0.8
0.6
0.4

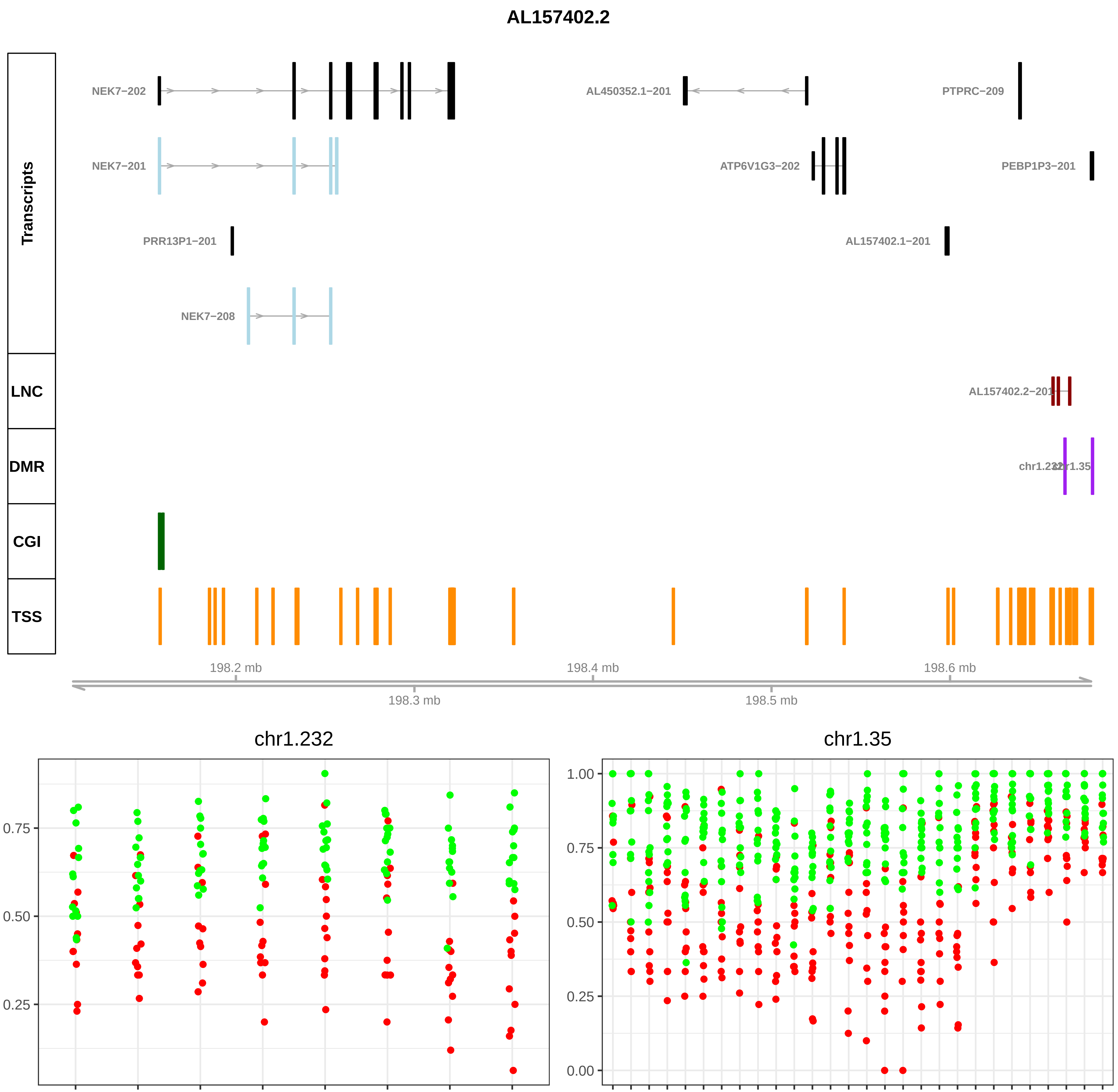

AL157402.2
Transcripts
NEK7−202
AL450352.1−201
PTPRC−209
NEK7−201
ATP6V1G3−202
PEBP1P3−201
PRR13P1−201
AL157402.1−201
NEK7−208
LNC
AL157402.2−201
DMR
chr1.232
chr1.35
CGI
TSS
198.2 mb
198.4 mb
198.6 mb
198.3 mb
198.5 mb
chr1.232
0.75
0.50
0.25
chr1.35
1.00
0.75
0.50
0.25
0.00

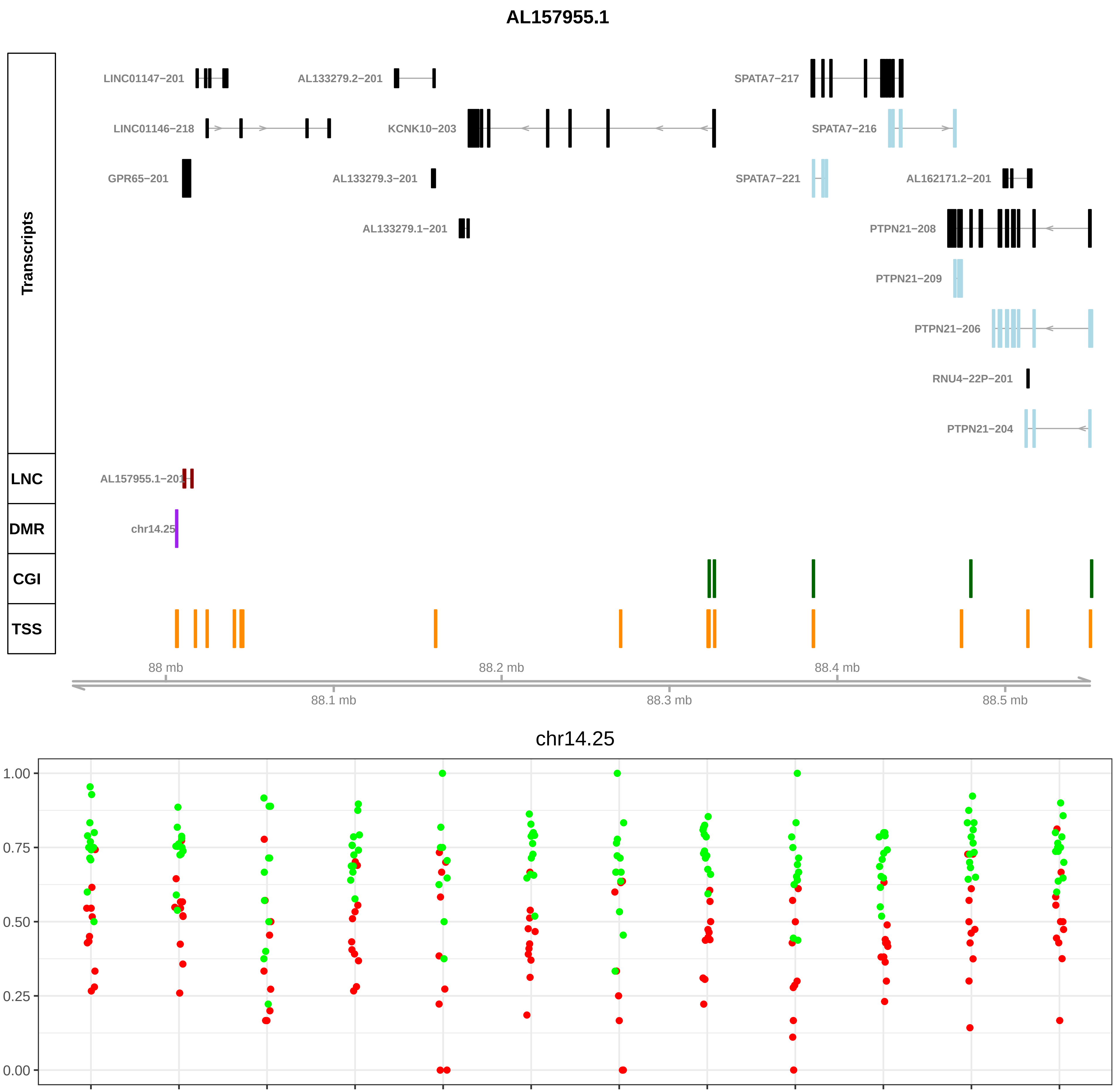

AL157955.1
Transcripts
LINC01147−201
AL133279.2−201
SPATA7−217
LINC01146−218
KCNK10−203
SPATA7−216
GPR65−201
AL133279.3−201
SPATA7−221
AL162171.2−201
AL133279.1−201
PTPN21−208
PTPN21−209
PTPN21−206
RNU4−22P−201
PTPN21−204
LNC
AL157955.1−201
DMR
chr14.25
CGI
TSS
88 mb
88.2 mb
88.4 mb
88.1 mb
88.3 mb
88.5 mb
chr14.25
1.00
0.75
0.50
0.25
0.00

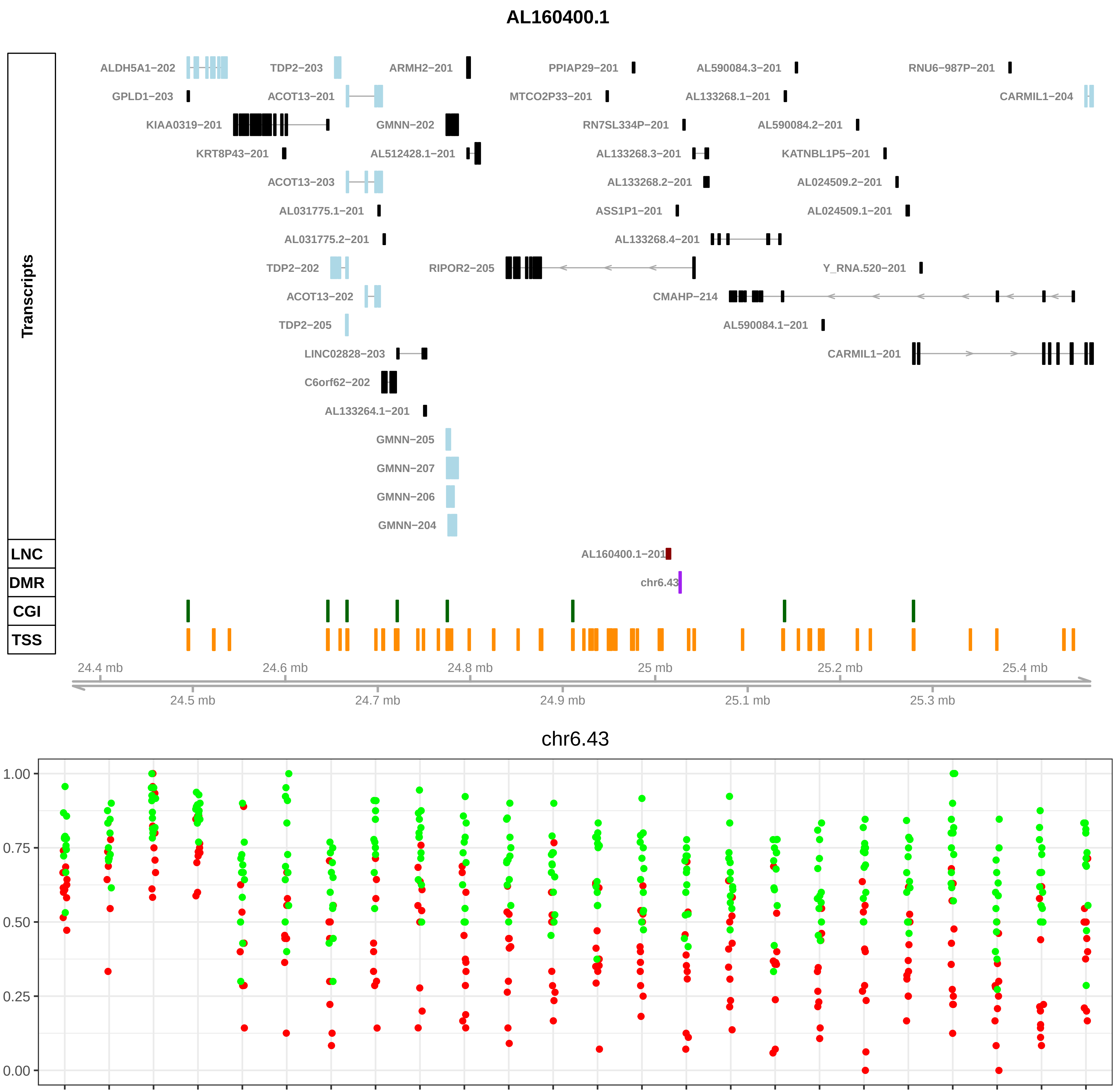

AL160400.1
Transcripts
ALDH5A1−202
TDP2−203
ARMH2−201
PPIAP29−201
AL590084.3−201
RNU6−987P−201
GPLD1−203
ACOT13−201
MTCO2P33−201
AL133268.1−201
CARMIL1−204
KIAA0319−201
GMNN−202
RN7SL334P−201
AL590084.2−201
KRT8P43−201
AL512428.1−201
AL133268.3−201
KATNBL1P5−201
ACOT13−203
AL133268.2−201
AL024509.2−201
AL031775.1−201
ASS1P1−201
AL024509.1−201
AL031775.2−201
AL133268.4−201
TDP2−202
RIPOR2−205
Y_RNA.520−201
ACOT13−202
CMAHP−214
TDP2−205
AL590084.1−201
LINC02828−203
CARMIL1−201
C6orf62−202
AL133264.1−201
GMNN−205
GMNN−207
GMNN−206
GMNN−204
LNC
AL160400.1−201
DMR
chr6.43
CGI
TSS
24.4 mb
24.6 mb
24.8 mb
25 mb
25.2 mb
25.4 mb
24.5 mb
24.7 mb
24.9 mb
25.1 mb
25.3 mb
chr6.43
1.00
0.75
0.50
0.25
0.00

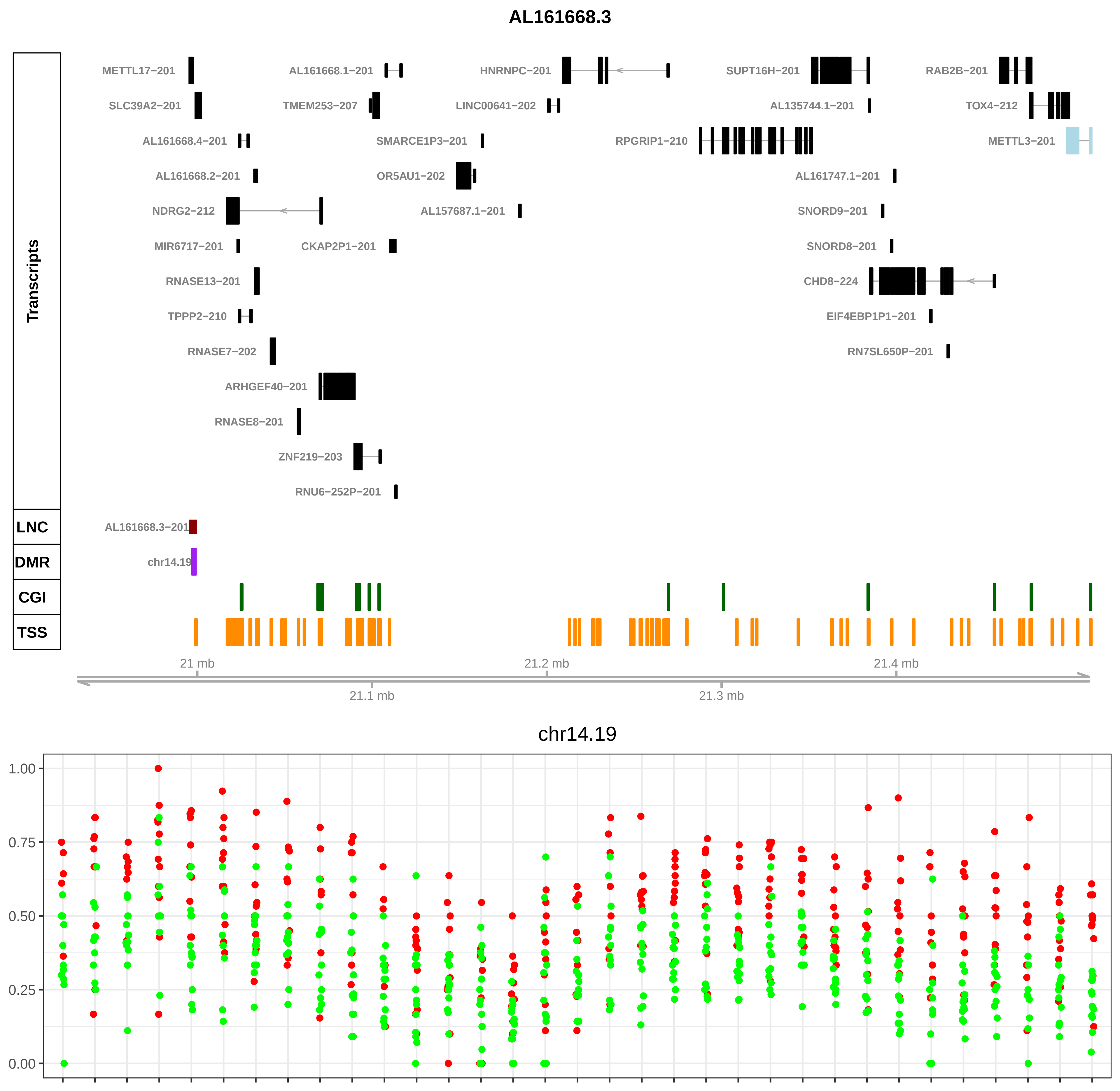
AL161668.3
Transcripts
METTL17−201
SLC39A2−201
AL161668.4−201
AL161668.2−201
NDRG2−212
MIR6717−201
RNASE13−201
TPPP2−210
RNASE7−202
ARHGEF40−201
RNASE8−201
ZNF219−203
RNU6−252P−201
AL161668.1−201
TMEM253−207
SMARCE1P3−201
OR5AU1−202
AL157687.1−201
CKAP2P1−201
HNRNPC−201
LINC00641−202
SUPT16H−201
AL135744.1−201
RPGRIP1−210
AL161747.1−201
SNORD9−201
SNORD8−201
CHD8−224
EIF4EBP1P1−201
RN7SL650P−201
RAB2B−201
TOX4−212
METTL3−201
LNC
AL161668.3−201
DMR
chr14.19
CGI
TSS
21 mb
21.1 mb
21.2 mb
21.3 mb
21.4 mb
chr14.19
1.00
0.75
0.50
0.25
0.00

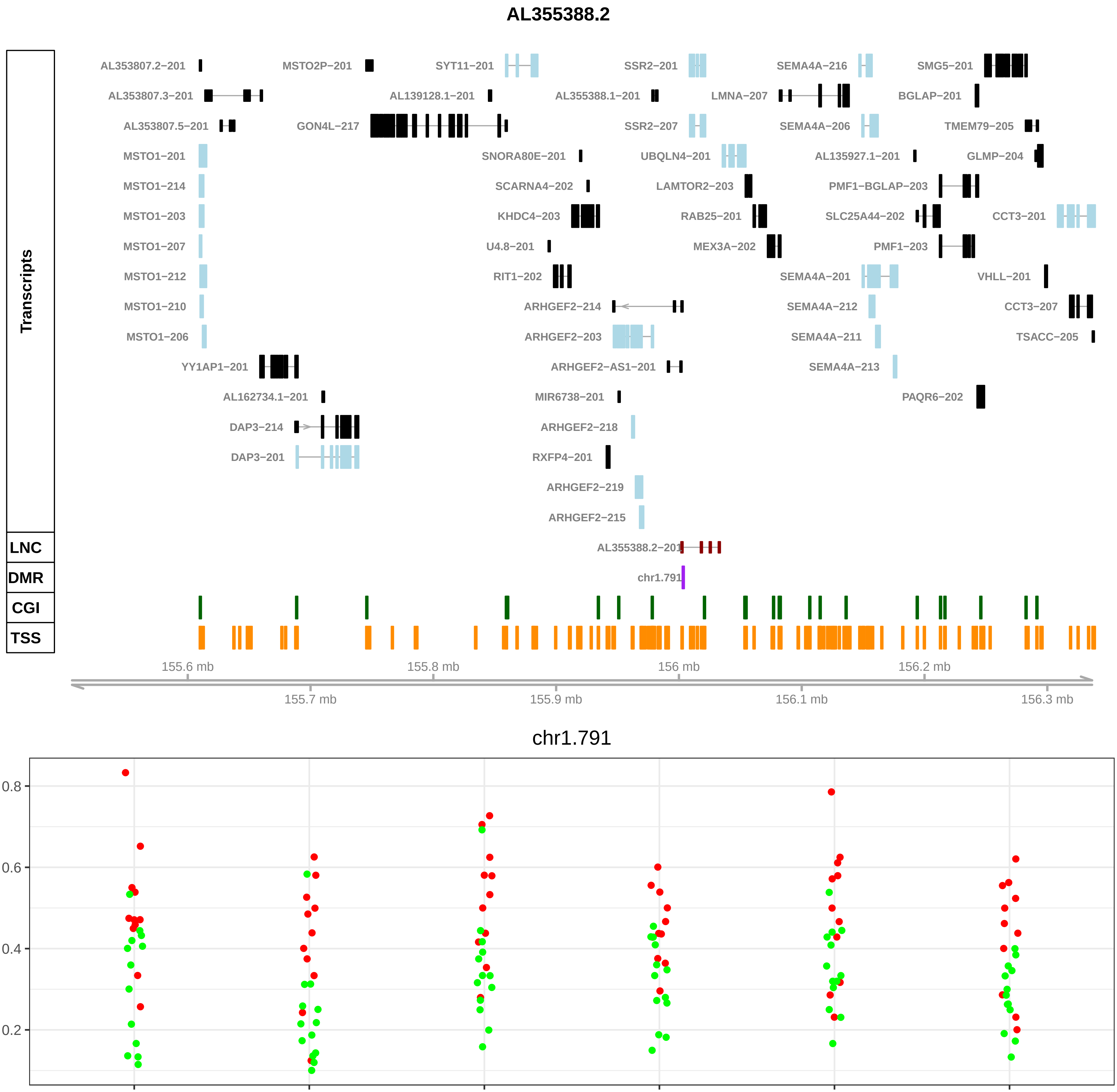

AL355388.2
Transcripts
LNC
DMR
CGI
TSS
AL353807.2−201
MSTO2P−201
SYT11−201
SSR2−201
SEMA4A−216
SMG5−201
AL353807.3−201
AL139128.1−201
AL355388.1−201
LMNA−207
BGLAP−201
AL353807.5−201
GON4L−217
SSR2−207
SEMA4A−206
TMEM79−205
MSTO1−201
SNORA80E−201
UBQLN4−201
AL135927.1−201
GLMP−204
MSTO1−214
SCARNA4−202
LAMTOR2−203
PMF1−BGLAP−203
MSTO1−203
KHDC4−203
RAB25−201
SLC25A44−202
CCT3−201
MSTO1−207
U4.8−201
MEX3A−202
PMF1−203
MSTO1−212
RIT1−202
SEMA4A−201
VHLL−201
MSTO1−210
ARHGEF2−214
SEMA4A−212
CCT3−207
MSTO1−206
ARHGEF2−203
SEMA4A−211
TSACC−205
YY1AP1−201
ARHGEF2−AS1−201
SEMA4A−213
AL162734.1−201
MIR6738−201
PAQR6−202
DAP3−214
ARHGEF2−218
DAP3−201
RXFP4−201
ARHGEF2−219
ARHGEF2−215
AL355388.2−201
chr1.791
155.6 mb
155.8 mb
156 mb
156.2 mb
155.7 mb
155.9 mb
156.1 mb
156.3 mb
chr1.791
0.8
0.6
0.4
0.2

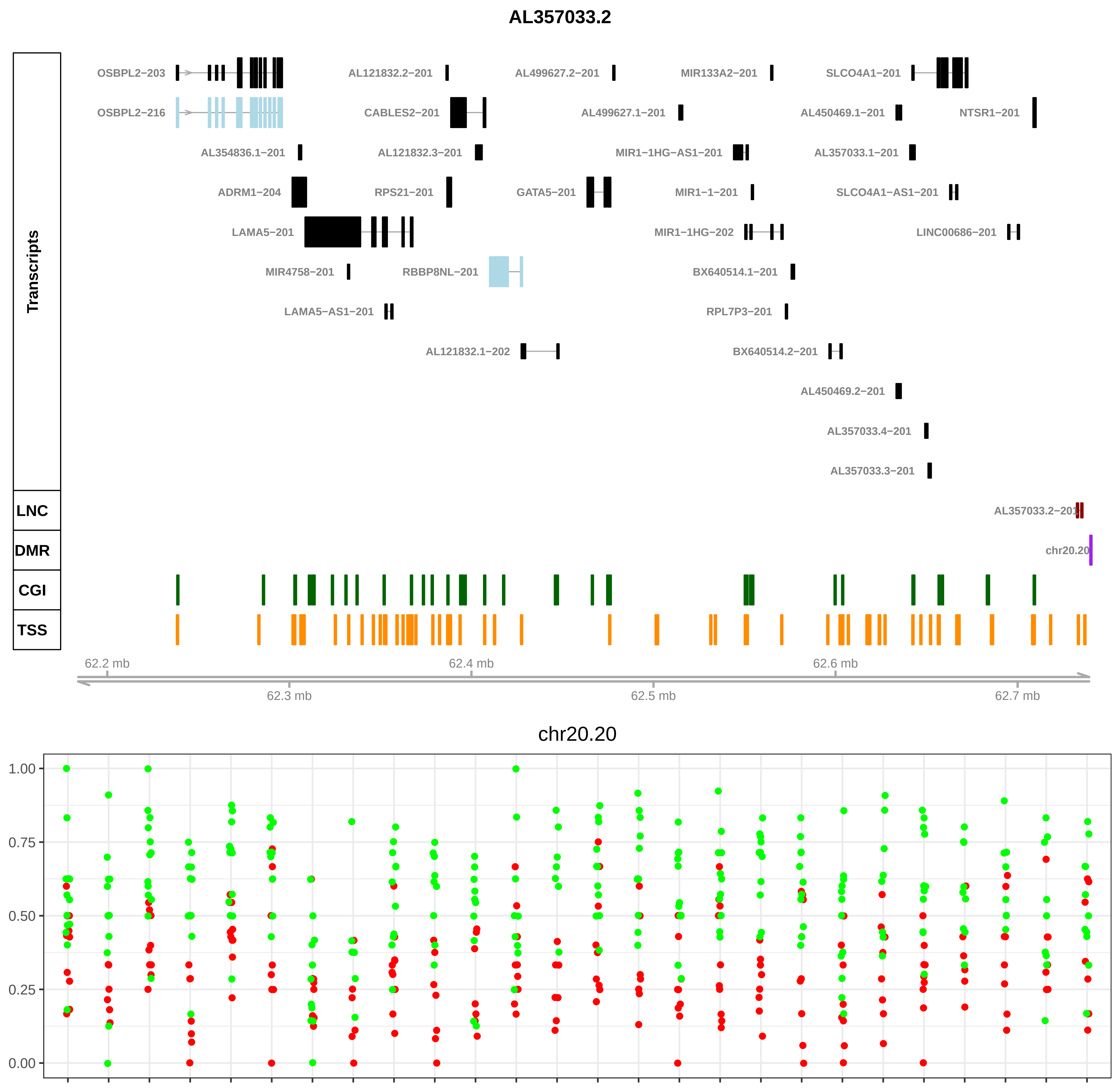
AL357033.2
Transcripts
OSBPL2−203
OSBPL2−216
AL354836.1−201
ADRM1−204
LAMA5−201
MIR4758−201
LAMA5−AS1−201
AL121832.2−201
CABLES2−201
AL121832.3−201
RPS21−201
RBBP8NL−201
AL121832.1−202
AL499627.2−201
AL499627.1−201
GATA5−201
MIR133A2−201
MIR1−1HG−AS1−201
MIR1−1−201
MIR1−1HG−202
BX640514.1−201
RPL7P3−201
BX640514.2−201
AL450469.2−201
AL357033.4−201
AL357033.3−201
SLCO4A1−201
AL450469.1−201
NTSR1−201
AL357033.1−201
SLCO4A1−AS1−201
LINC00686−201
LNC
AL357033.2−201
DMR
chr20.20
CGI
TSS
62.2 mb
62.3 mb
62.4 mb
62.5 mb
62.6 mb
62.7 mb
chr20.20
1.00
0.75
0.50
0.25
0.00

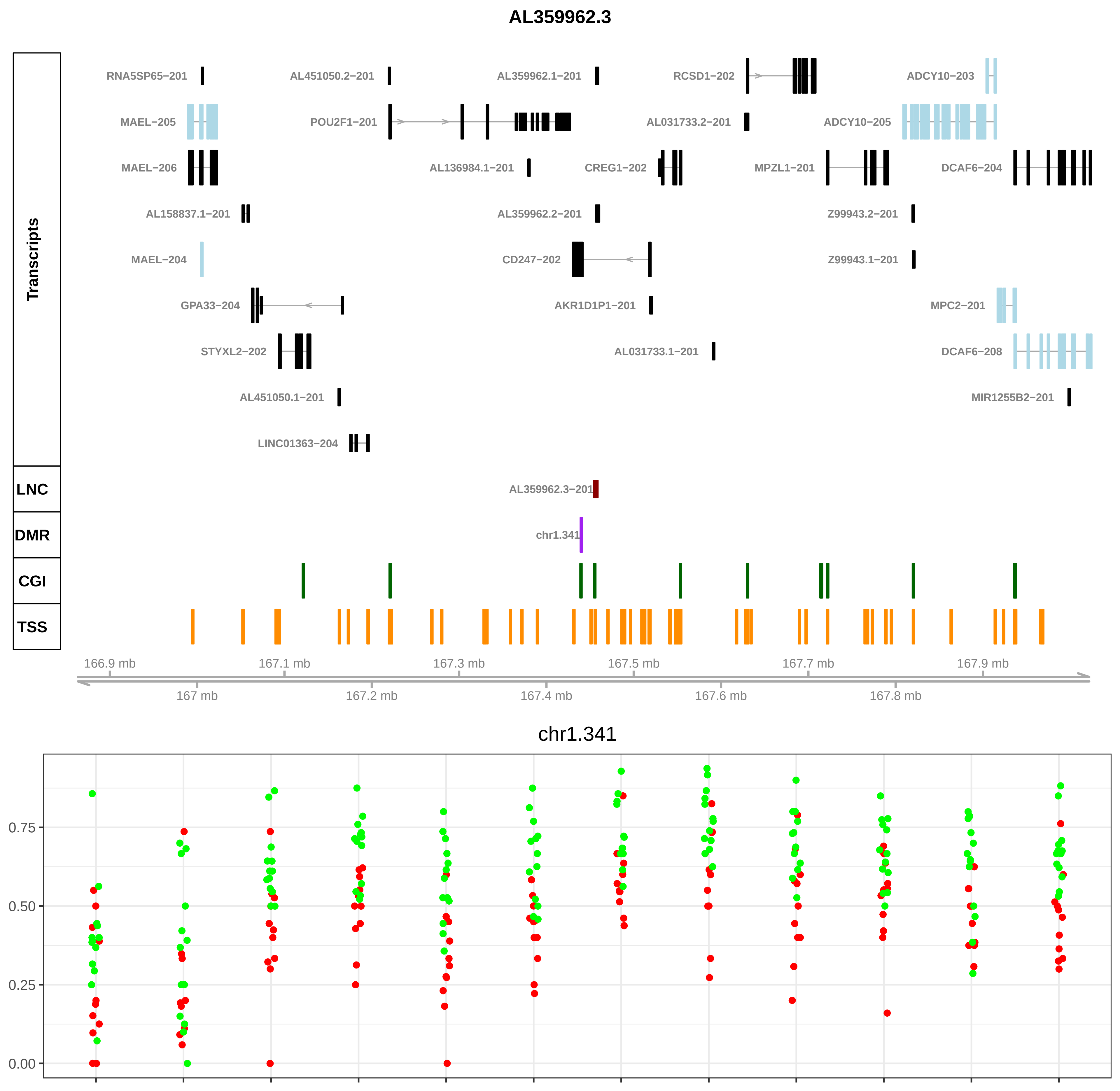

AL359962.3
Transcripts
LNC
DMR
CGI
TSS
RNA5SP65−201
AL451050.2−201
AL359962.1−201
RCSD1−202
ADCY10−203
MAEL−205
POU2F1−201
AL031733.2−201
ADCY10−205
MAEL−206
AL136984.1−201
CREG1−202
MPZL1−201
DCAF6−204
AL158837.1−201
AL359962.2−201
Z99943.2−201
MAEL−204
CD247−202
Z99943.1−201
GPA33−204
AKR1D1P1−201
MPC2−201
STYXL2−202
AL031733.1−201
DCAF6−208
AL451050.1−201
MIR1255B2−201
LINC01363−204
AL359962.3−201
chr1.341
166.9 mb
167.1 mb
167.3 mb
167.5 mb
167.7 mb
167.9 mb
167 mb
167.2 mb
167.4 mb
167.6 mb
167.8 mb
chr1.341
0.75
0.50
0.25
0.00

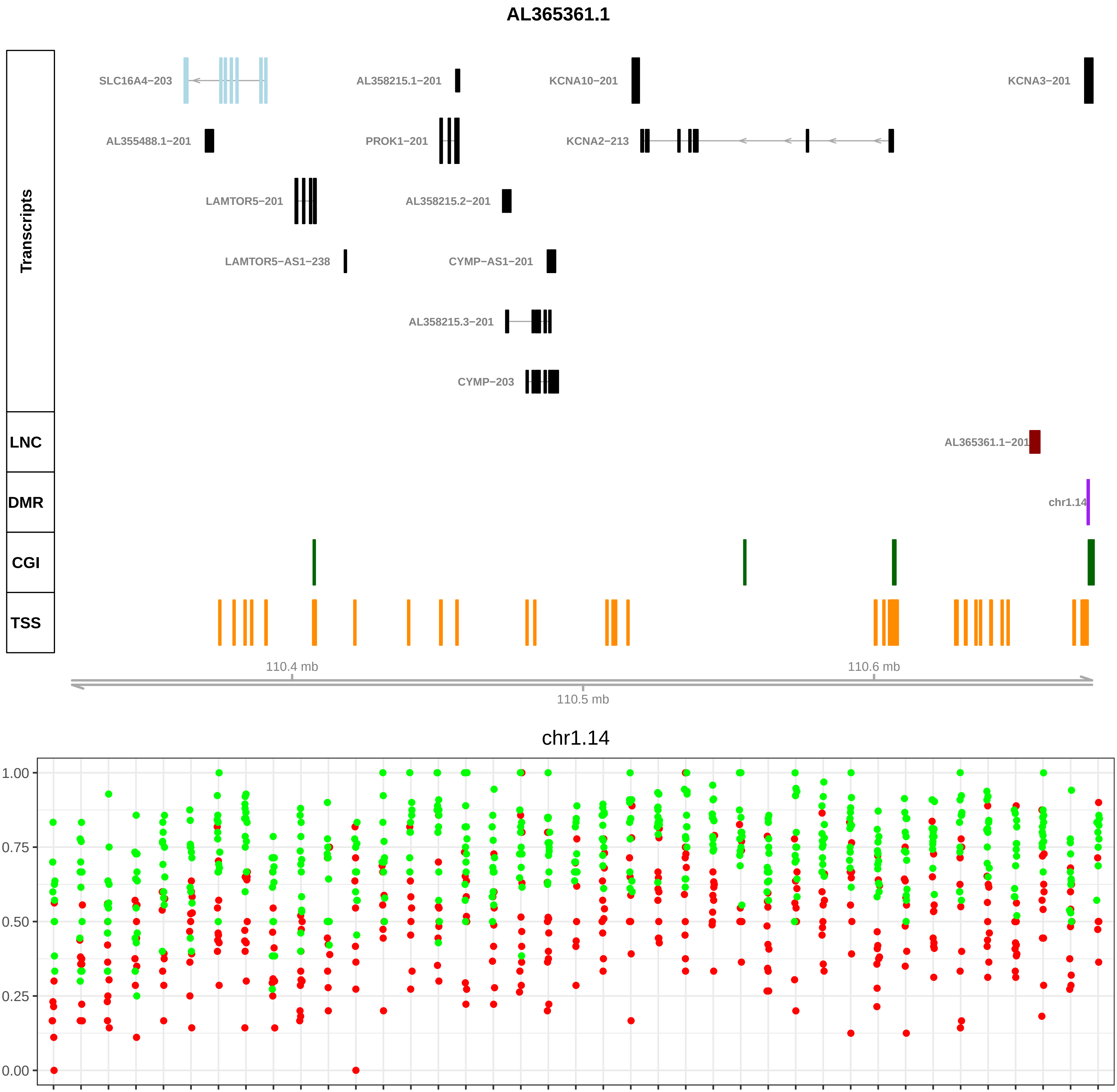

AL365361.1
Transcripts
SLC16A4−203
AL358215.1−201
KCNA10−201
KCNA3−201
AL355488.1−201
PROK1−201
KCNA2−213
LAMTOR5−201
AL358215.2−201
LAMTOR5−AS1−238
CYMP−AS1−201
AL358215.3−201
CYMP−203
LNC
AL365361.1−201
DMR
chr1.14
CGI
TSS
110.4 mb
110.6 mb
110.5 mb
chr1.14
1.00
0.75
0.50
0.25
0.00

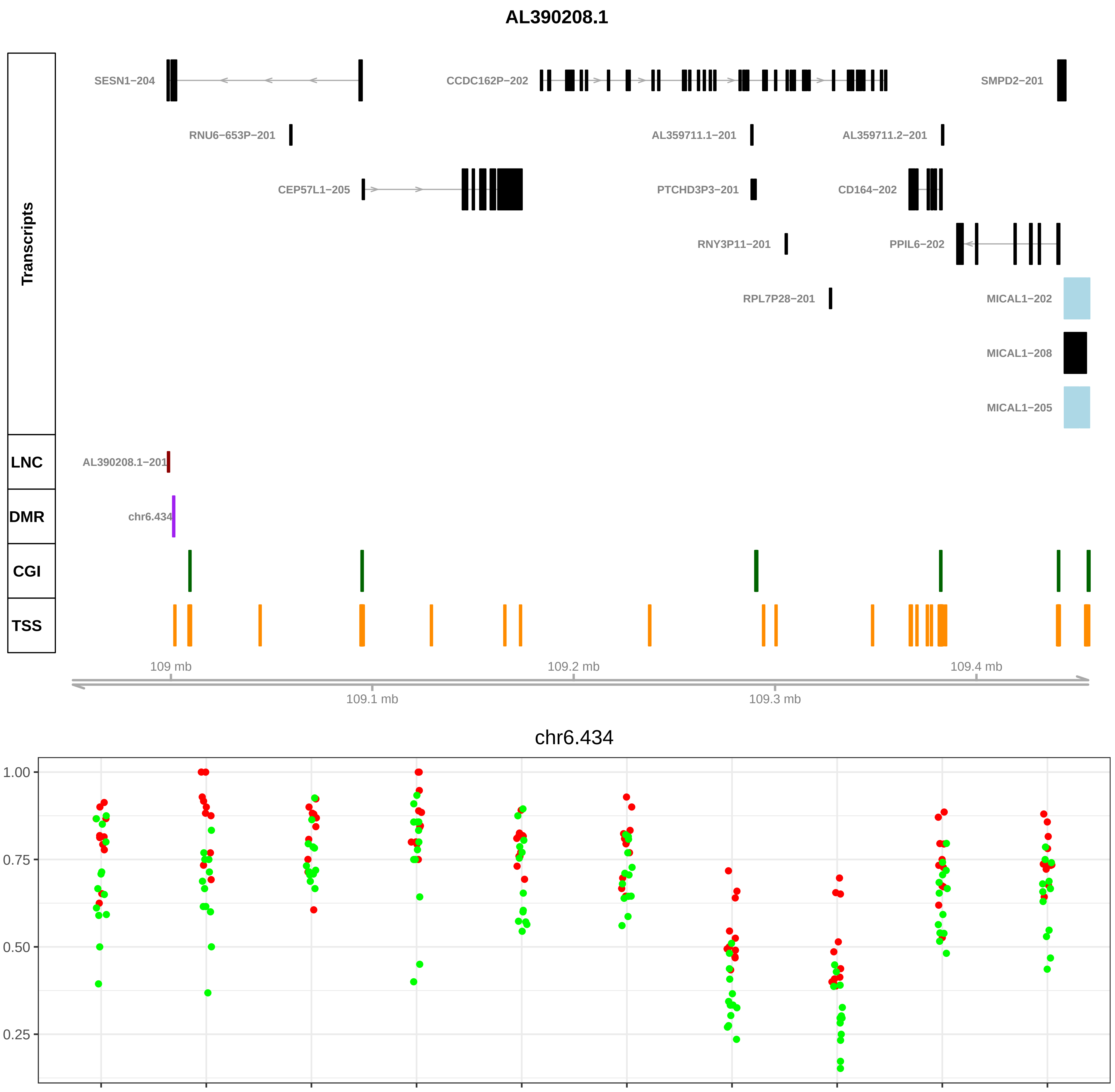

AL390208.1
Transcripts
SESN1−204
CCDC162P−202
SMPD2−201
RNU6−653P−201
AL359711.1−201
AL359711.2−201
CEP57L1−205
PTCHD3P3−201
CD164−202
RNY3P11−201
PPIL6−202
RPL7P28−201
MICAL1−202
MICAL1−208
MICAL1−205
LNC
AL390208.1−201
DMR
chr6.434
CGI
TSS
109 mb
109.1 mb
109.2 mb
109.3 mb
109.4 mb
chr6.434
1.00
0.75
0.50
0.25

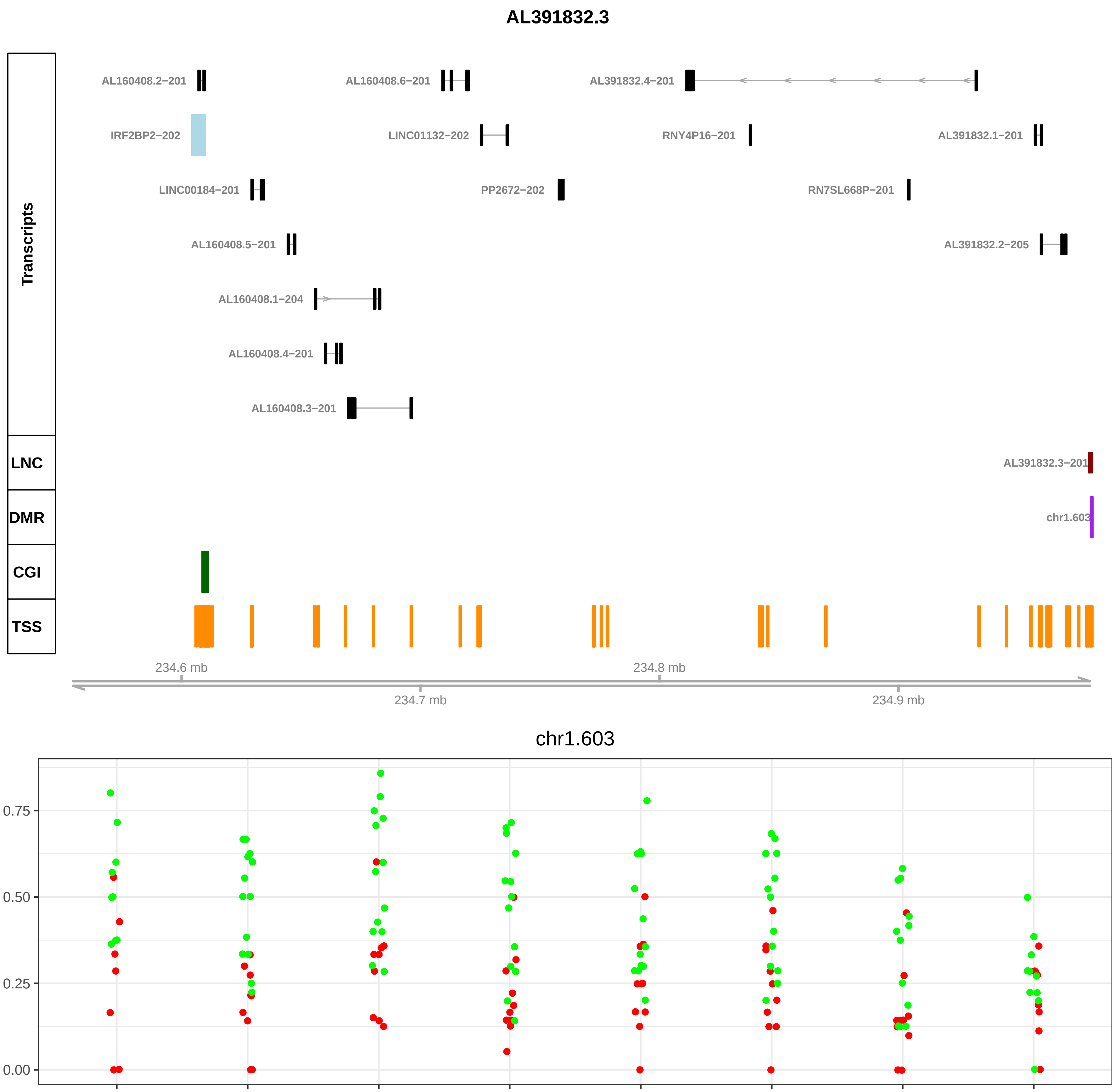

AL391832.3
Transcripts
AL160408.2−201
AL160408.6−201
AL391832.4−201
IRF2BP2−202
LINC01132−202
RNY4P16−201
AL391832.1−201
LINC00184−201
PP2672−202
RN7SL668P−201
AL160408.5−201
AL391832.2−205
AL160408.1−204
AL160408.4−201
AL160408.3−201
LNC
AL391832.3−201
DMR
chr1.603
CGI
TSS
234.6 mb
234.8 mb
234.7 mb
234.9 mb
chr1.603
0.75
0.50
0.25
0.00

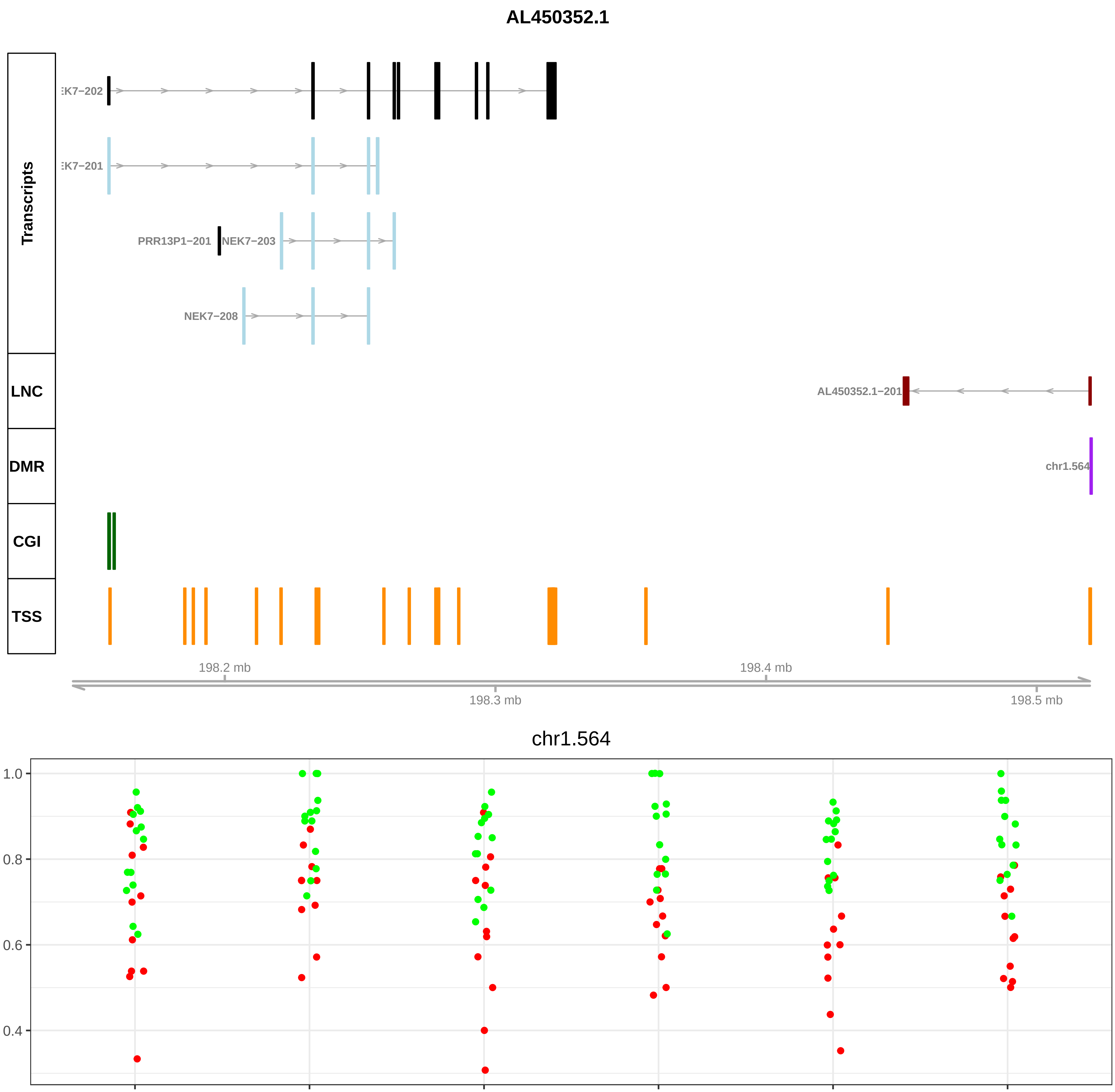

AL450352.1
Transcripts
LNC
DMR
CGI
TSS
EK7−202
EK7−201
PRR13P1−201
NEK7−203
NEK7−208
AL450352.1−201
chr1.564
198.2 mb
198.4 mb
198.3 mb
198.5 mb
chr1.564
1.0
0.8
0.6
0.4

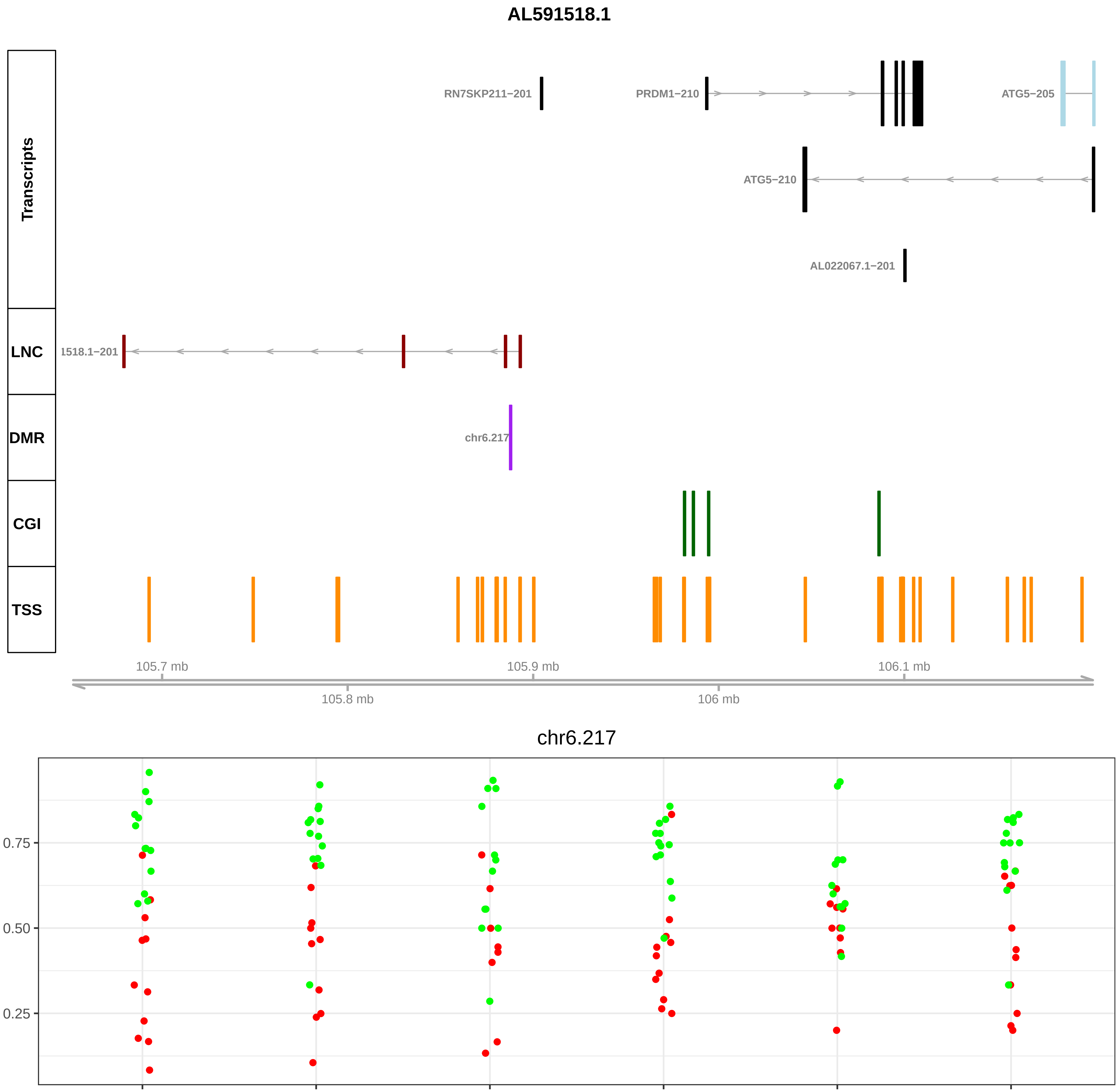

AL591518.1
Transcripts
RN7SKP211−201
PRDM1−210
ATG5−205
ATG5−210
AL022067.1−201
LNC
1518.1−201
DMR
chr6.217
CGI
TSS
105.7 mb
105.9 mb
106.1 mb
105.8 mb
106 mb
chr6.217
0.75
0.50
0.25

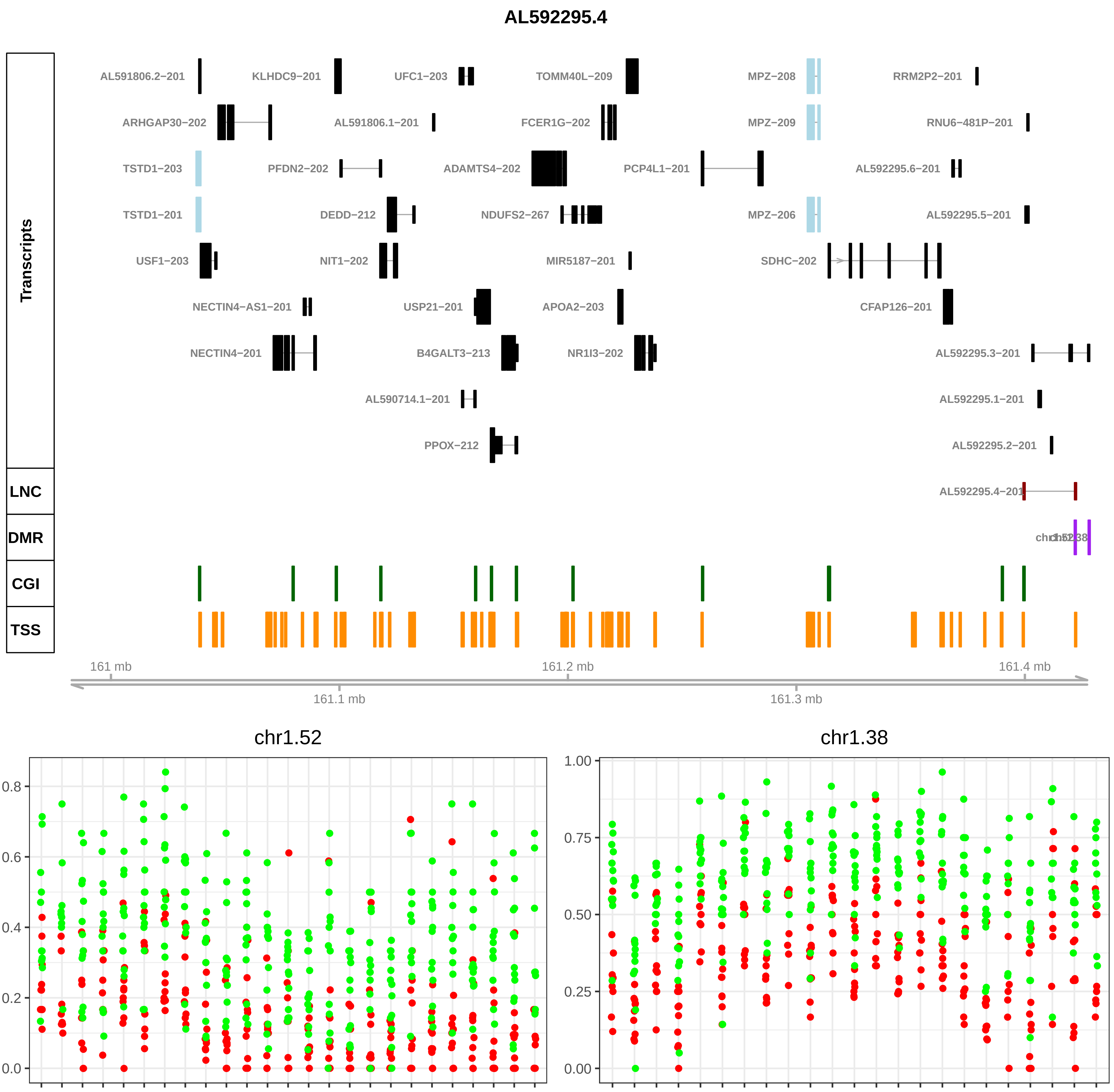
AL592295.4
Transcripts
AL591806.2−201
KLHDC9−201
UFC1−203
TOMM40L−209
MPZ−208
RRM2P2−201
ARHGAP30−202
AL591806.1−201
FCER1G−202
MPZ−209
RNU6−481P−201
TSTD1−203
PFDN2−202
ADAMTS4−202
PCP4L1−201
AL592295.6−201
TSTD1−201
DEDD−212
NDUFS2−267
MPZ−206
AL592295.5−201
USF1−203
NIT1−202
MIR5187−201
SDHC−202
NECTIN4−AS1−201
USP21−201
APOA2−203
CFAP126−201
NECTIN4−201
B4GALT3−213
NR1I3−202
AL592295.3−201
AL590714.1−201
AL592295.1−201
PPOX−212
AL592295.2−201
LNC
AL592295.4−201
DMR
CGI
TSS
161 mb
161.1 mb
161.2 mb
161.3 mb
161.4 mb
chr1.52
0.0
0.2
0.4
0.6
0.8
chr1.38
0.00
0.25
0.50
0.75
1.00

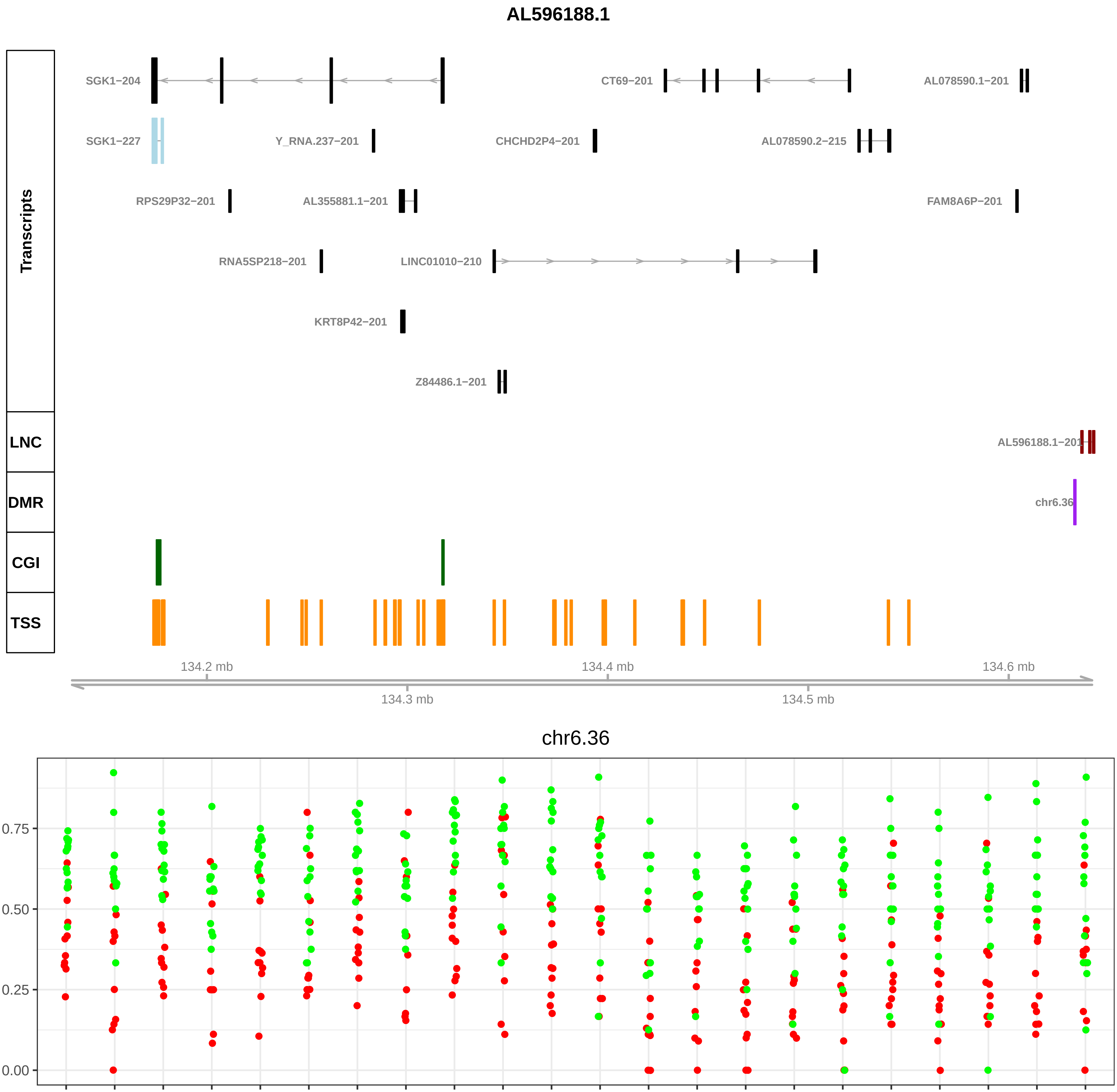

AL596188.1
Transcripts
SGK1−204
CT69−201
AL078590.1−201
SGK1−227
Y_RNA.237−201
CHCHD2P4−201
AL078590.2−215
RPS29P32−201
AL355881.1−201
FAM8A6P−201
RNA5SP218−201
LINC01010−210
KRT8P42−201
Z84486.1−201
LNC
AL596188.1−201
DMR
chr6.36
CGI
TSS
134.2 mb
134.4 mb
134.6 mb
134.3 mb
134.5 mb
chr6.36
0.75
0.50
0.25
0.00

AL603783.1

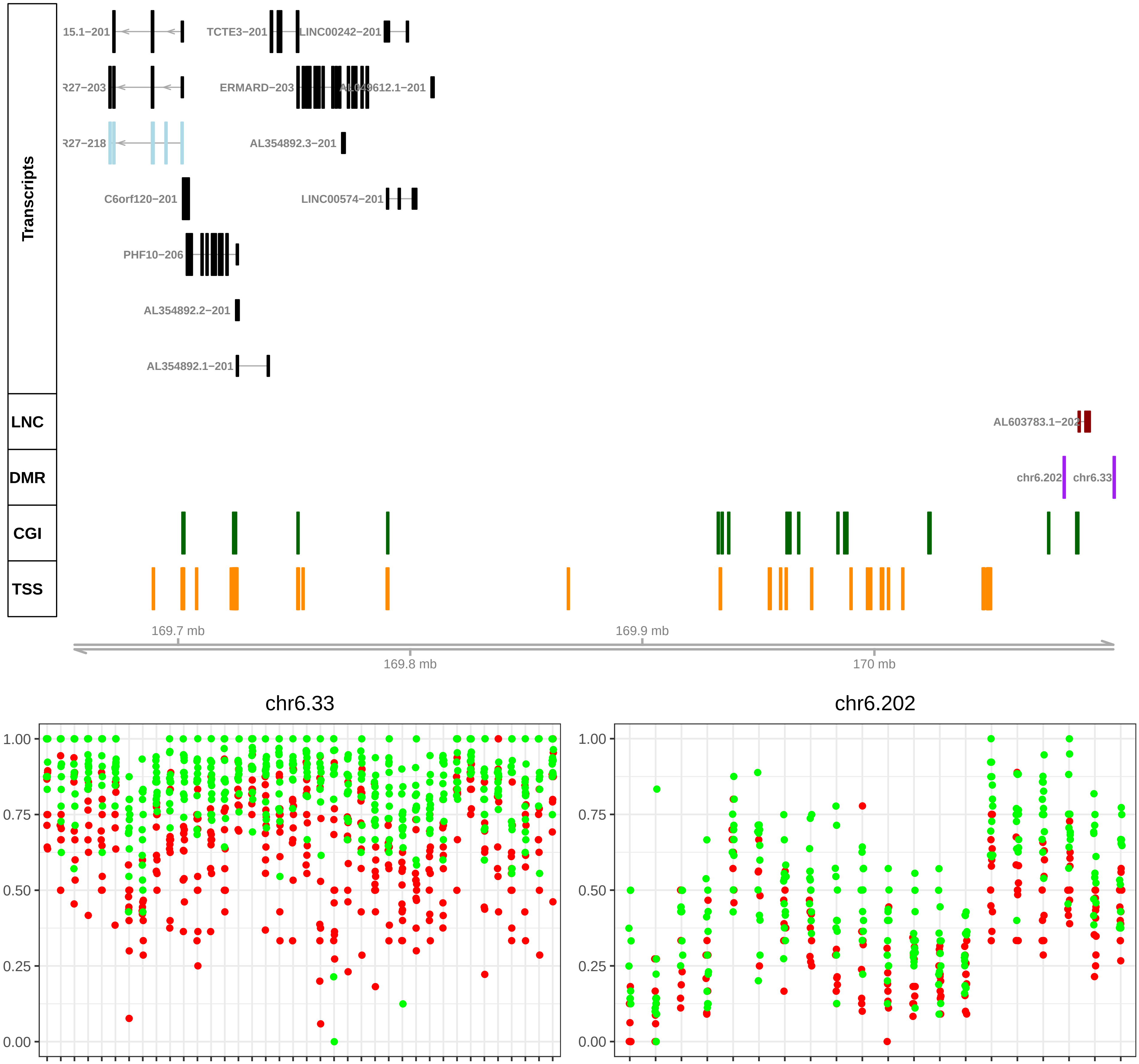

Transcripts
15.1−201
TCTE3−201
LINC00242−201
R27−203
ERMARD−203
AL049612.1−201
R27−218
AL354892.3−201
C6orf120−201
LINC00574−201
PHF10−206
AL354892.2−201
AL354892.1−201
LNC
AL603783.1−202
DMR
chr6.202
chr6.33
CGI
TSS
169.7 mb
169.8 mb
169.9 mb
170 mb
chr6.33
chr6.202
1.00
0.75
0.50
0.25
0.00

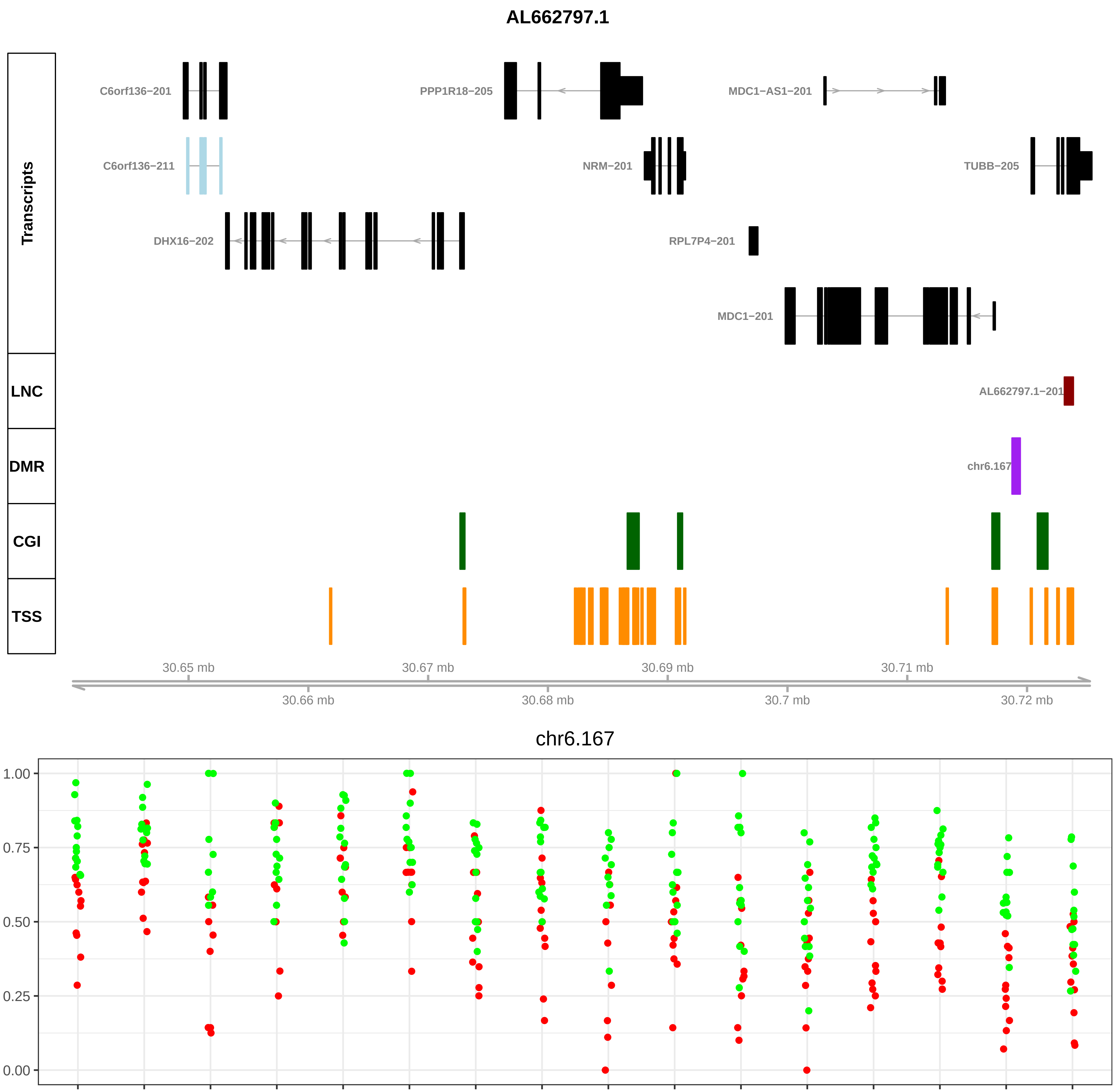
AL662797.1
Transcripts
C6orf136−201
PPP1R18−205
MDC1−AS1−201
C6orf136−211
NRM−201
TUBB−205
DHX16−202
RPL7P4−201
MDC1−201
LNC
AL662797.1−201
DMR
chr6.167
CGI
TSS
30.65 mb
30.67 mb
30.69 mb
30.71 mb
30.66 mb
30.68 mb
30.7 mb
30.72 mb
chr6.167
1.00
0.75
0.50
0.25
0.00

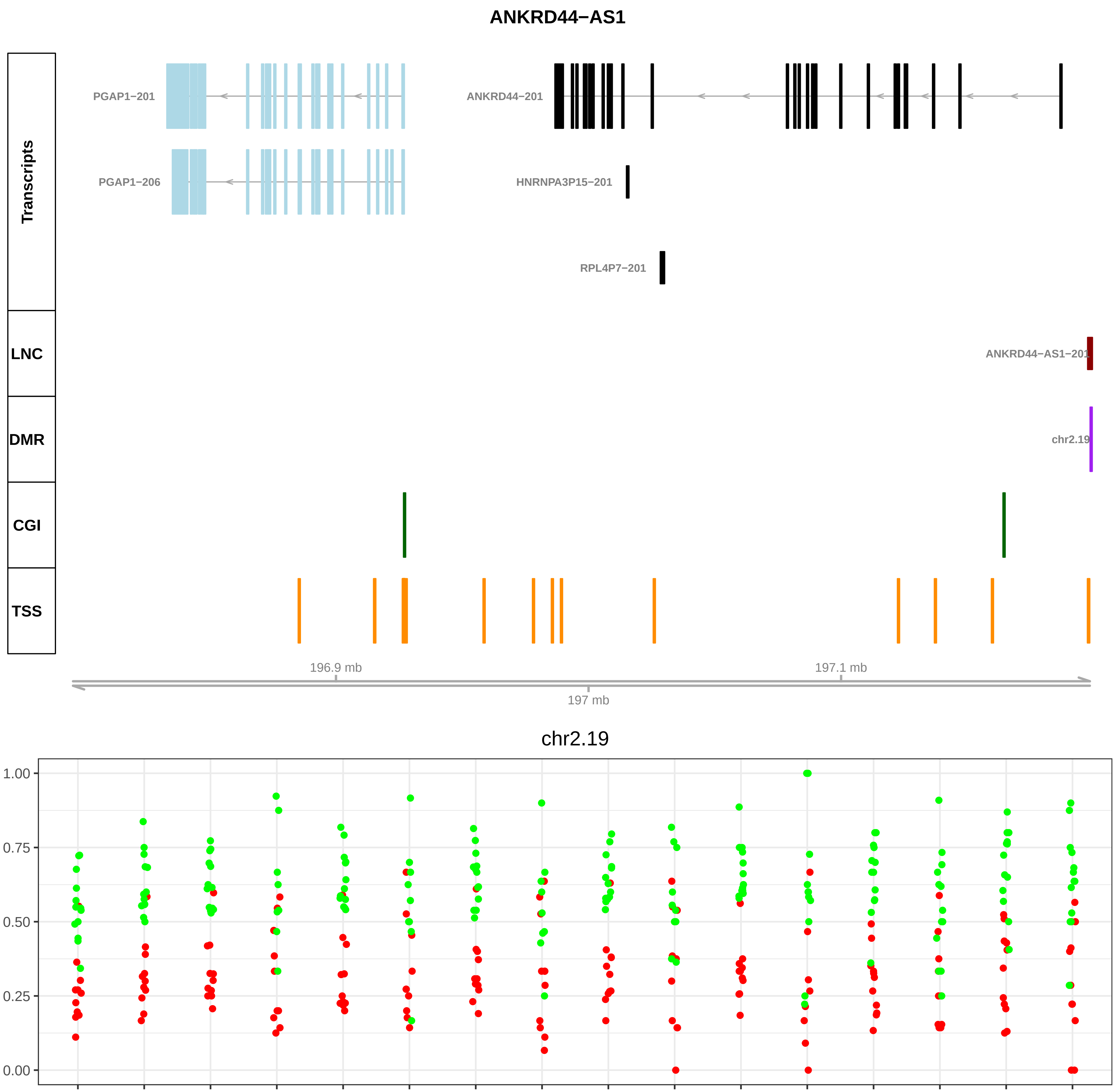
ANKRD44−AS1
Transcripts
PGAP1−201
PGAP1−206
ANKRD44−201
HNRNPA3P15−201
RPL4P7−201
LNC
ANKRD44−AS1−201
DMR
chr2.19
CGI
TSS
196.9 mb
197 mb
197.1 mb
chr2.19
1.00
0.75
0.50
0.25
0.00

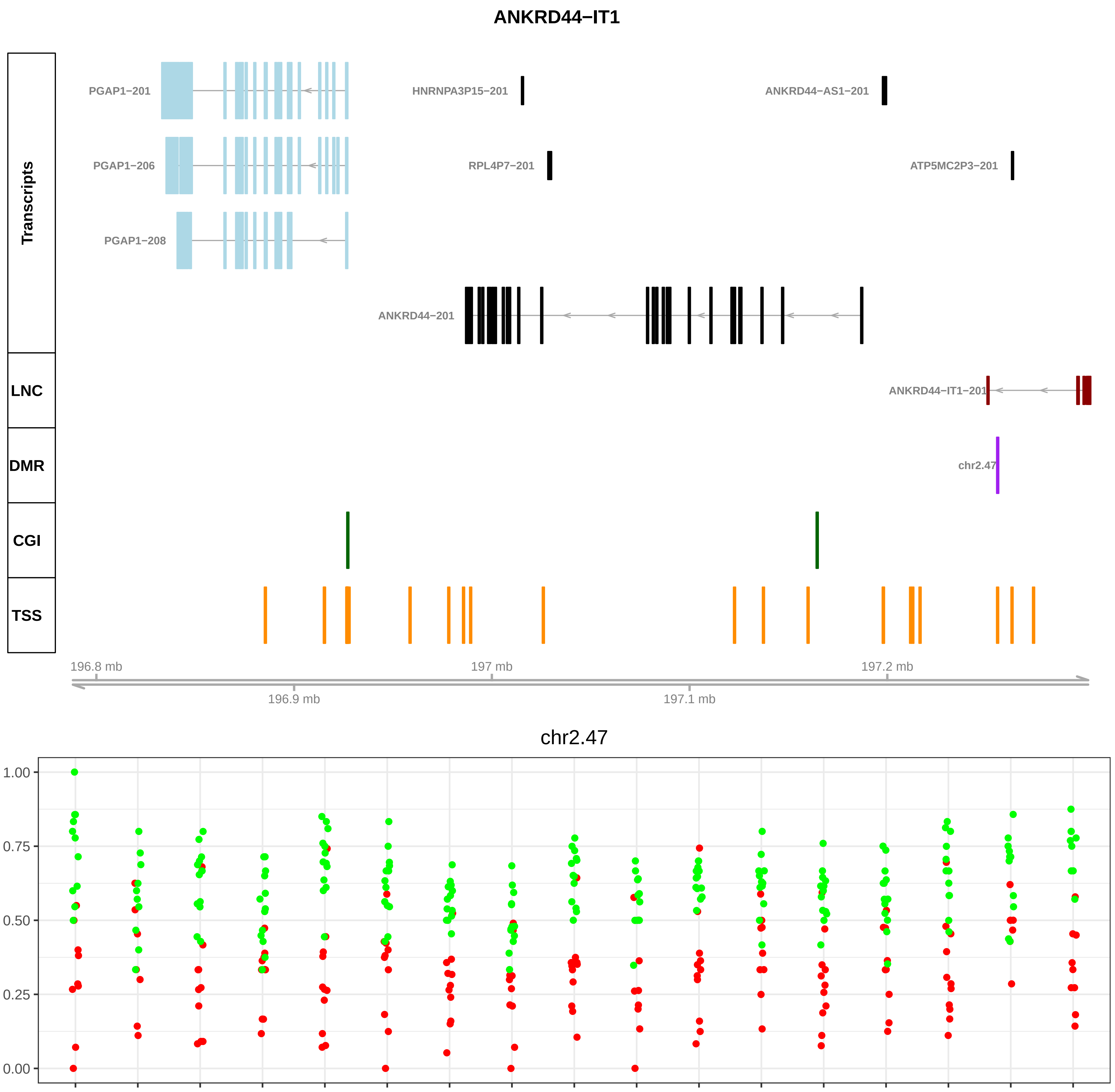
ANKRD44−IT1
Transcripts
PGAP1−201
PGAP1−206
PGAP1−208
HNRNPA3P15−201
RPL4P7−201
ANKRD44−AS1−201
ATP5MC2P3−201
ANKRD44−201
LNC
ANKRD44−IT1−201
DMR
chr2.47
CGI
TSS
196.8 mb
197 mb
197.2 mb
196.9 mb
197.1 mb
chr2.47
1.00
0.75
0.50
0.25
0.00

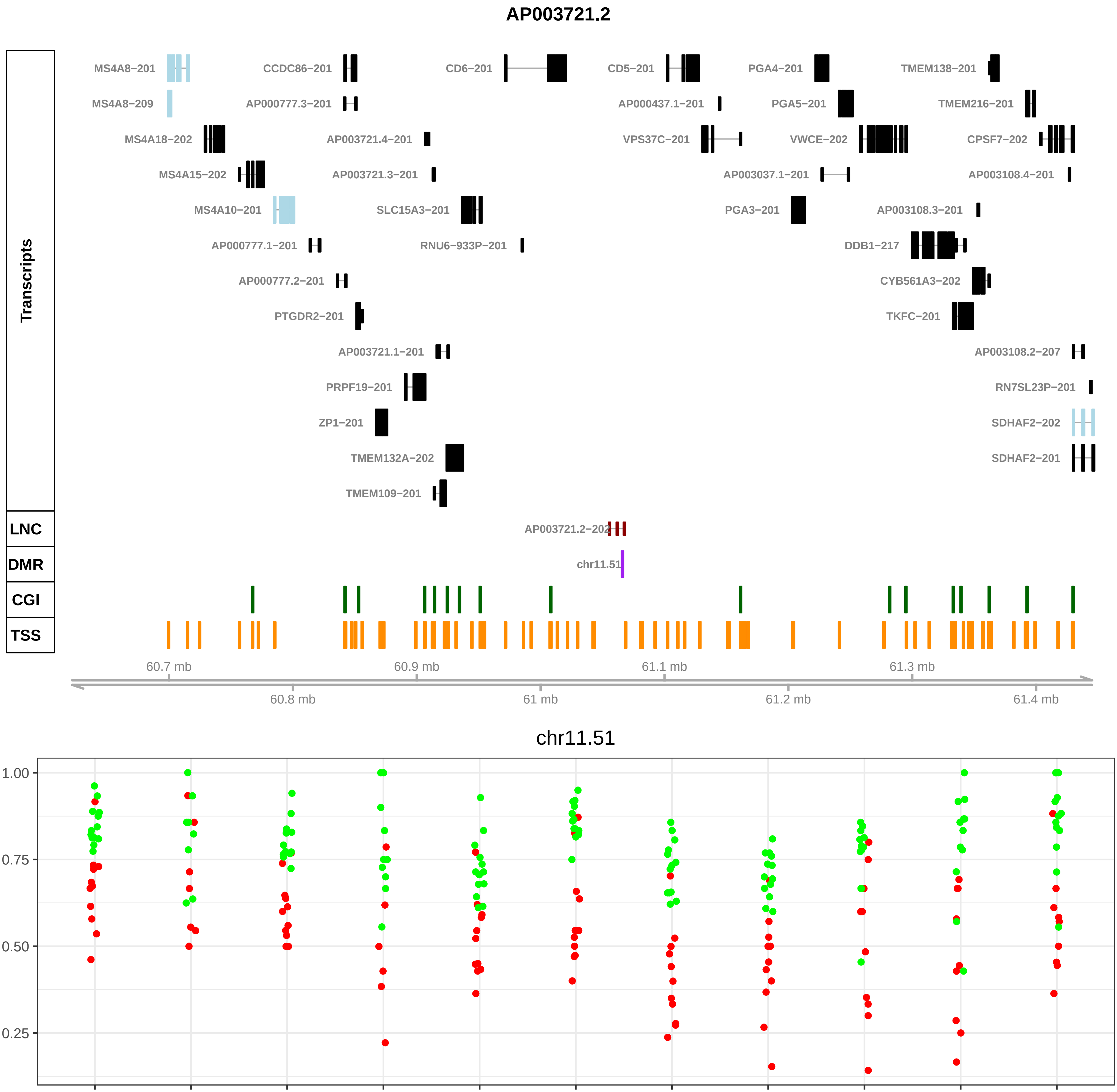
AP003721.2
Transcripts
MS4A8−201
MS4A8−209
MS4A18−202
MS4A15−202
MS4A10−201
AP000777.1−201
AP000777.2−201
PTGDR2−201
CCDC86−201
AP000777.3−201
AP003721.4−201
AP003721.3−201
SLC15A3−201
RNU6−933P−201
AP003721.1−201
PRPF19−201
ZP1−201
TMEM132A−202
TMEM109−201
CD6−201
CD5−201
AP000437.1−201
VPS37C−201
AP003037.1−201
PGA4−201
PGA5−201
VWCE−202
PGA3−201
TMEM138−201
TMEM216−201
CPSF7−202
AP003108.4−201
AP003108.3−201
DDB1−217
CYB561A3−202
TKFC−201
AP003108.2−207
RN7SL23P−201
SDHAF2−202
SDHAF2−201
LNC
AP003721.2−202
DMR
chr11.51
CGI
TSS
60.7 mb
60.8 mb
60.9 mb
61 mb
61.1 mb
61.2 mb
61.3 mb
61.4 mb
chr11.51
1.00
0.75
0.50
0.25

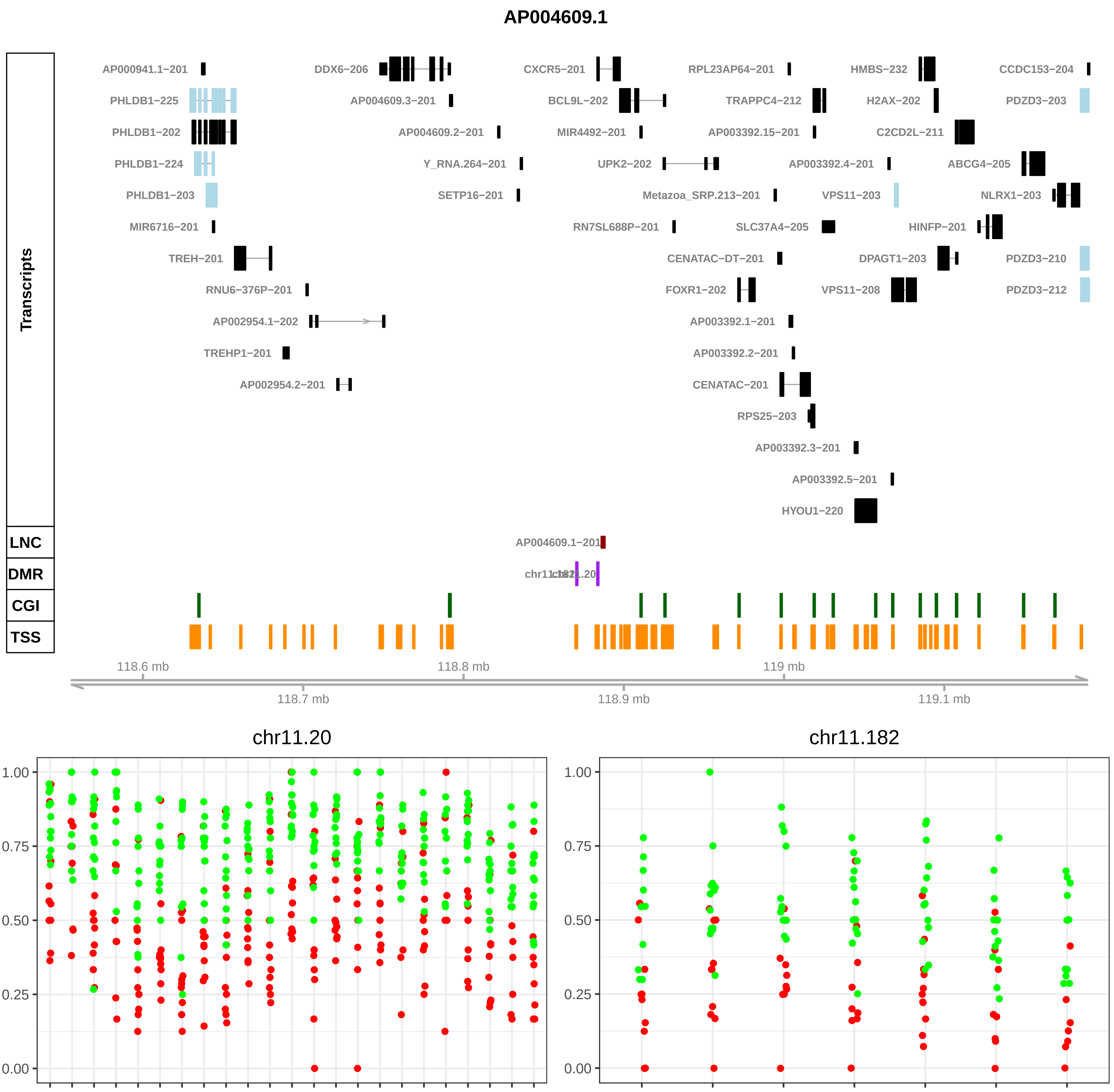
AP004609.1
Transcripts
AP000941.1−201
PHLDB1−225
PHLDB1−202
PHLDB1−224
PHLDB1−203
MIR6716−201
TREH−201
RNU6−376P−201
AP002954.1−202
TREHP1−201
AP002954.2−201
DDX6−206
AP004609.3−201
AP004609.2−201
Y_RNA.264−201
SETP16−201
CXCR5−201
BCL9L−202
MIR4492−201
UPK2−202
Metazoa_SRP.213−201
RN7SL688P−201
CENATAC−DT−201
FOXR1−202
AP003392.1−201
AP003392.2−201
CENATAC−201
RPS25−203
AP003392.3−201
AP003392.5−201
HYOU1−220
RPL23AP64−201
TRAPPC4−212
AP003392.15−201
AP003392.4−201
VPS11−205
SLC37A4−205
DPAGT1−203
VPS11−208
HMBS−232
H2AX−202
C2CD2L−211
ABCG4−205
NLRX1−203
HINFP−201
CCDC153−204
PDZD3−203
PDZD3−210
PDZD3−212
LNC
AP004609.1−201
DMR
CGI
TSS
118.6 mb
118.7 mb
118.8 mb
118.9 mb
119 mb
119.1 mb
chr11.20
chr11.182
1.00
0.75
0.50
0.25
0.00

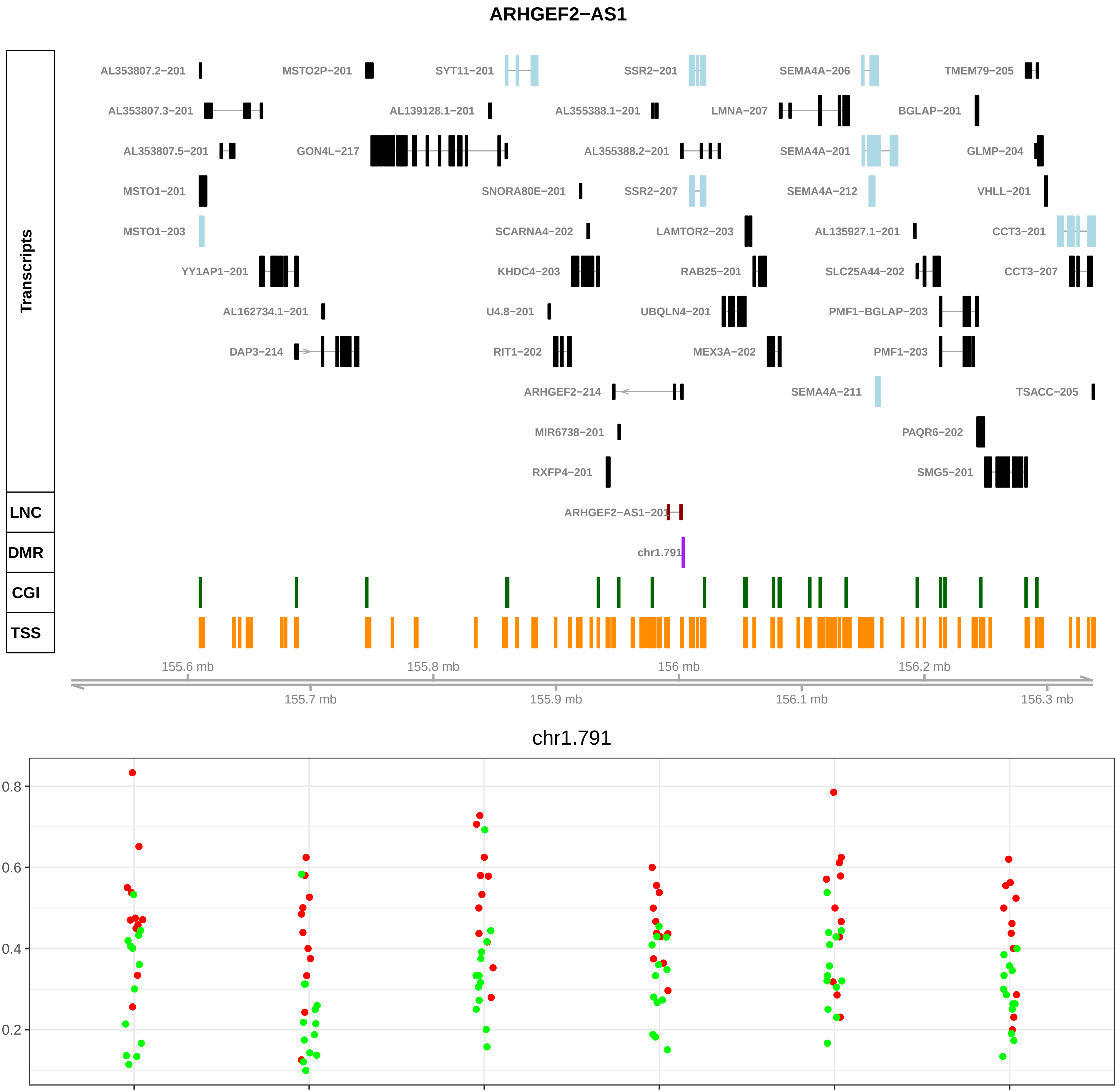
ARHGEF2−AS1
Transcripts
LNC
DMR
CGI
TSS
AL353807.2−201
MSTO2P−201
SYT11−201
SSR2−201
SEMA4A−206
TMEM79−205
AL353807.3−201
AL139128.1−201
AL355388.1−201
LMNA−207
BGLAP−201
AL353807.5−201
GON4L−217
AL355388.2−201
SEMA4A−201
GLMP−204
MSTO1−201
SNORA80E−201
SSR2−207
SEMA4A−212
VHLL−201
MSTO1−203
SCARNA4−202
LAMTOR2−203
AL135927.1−201
CCT3−201
YY1AP1−201
KHDC4−203
RAB25−201
SLC25A44−202
CCT3−207
AL162734.1−201
U4.8−201
UBQLN4−201
PMF1−BGLAP−203
DAP3−214
RIT1−202
MEX3A−202
PMF1−203
ARHGEF2−214
SEMA4A−211
TSACC−205
MIR6738−201
PAQR6−202
RXFP4−201
SMG5−201
ARHGEF2−AS1−201
chr1.791
155.6 mb
155.8 mb
156 mb
156.2 mb
155.7 mb
155.9 mb
156.1 mb
156.3 mb
chr1.791
0.8
0.6
0.4
0.2

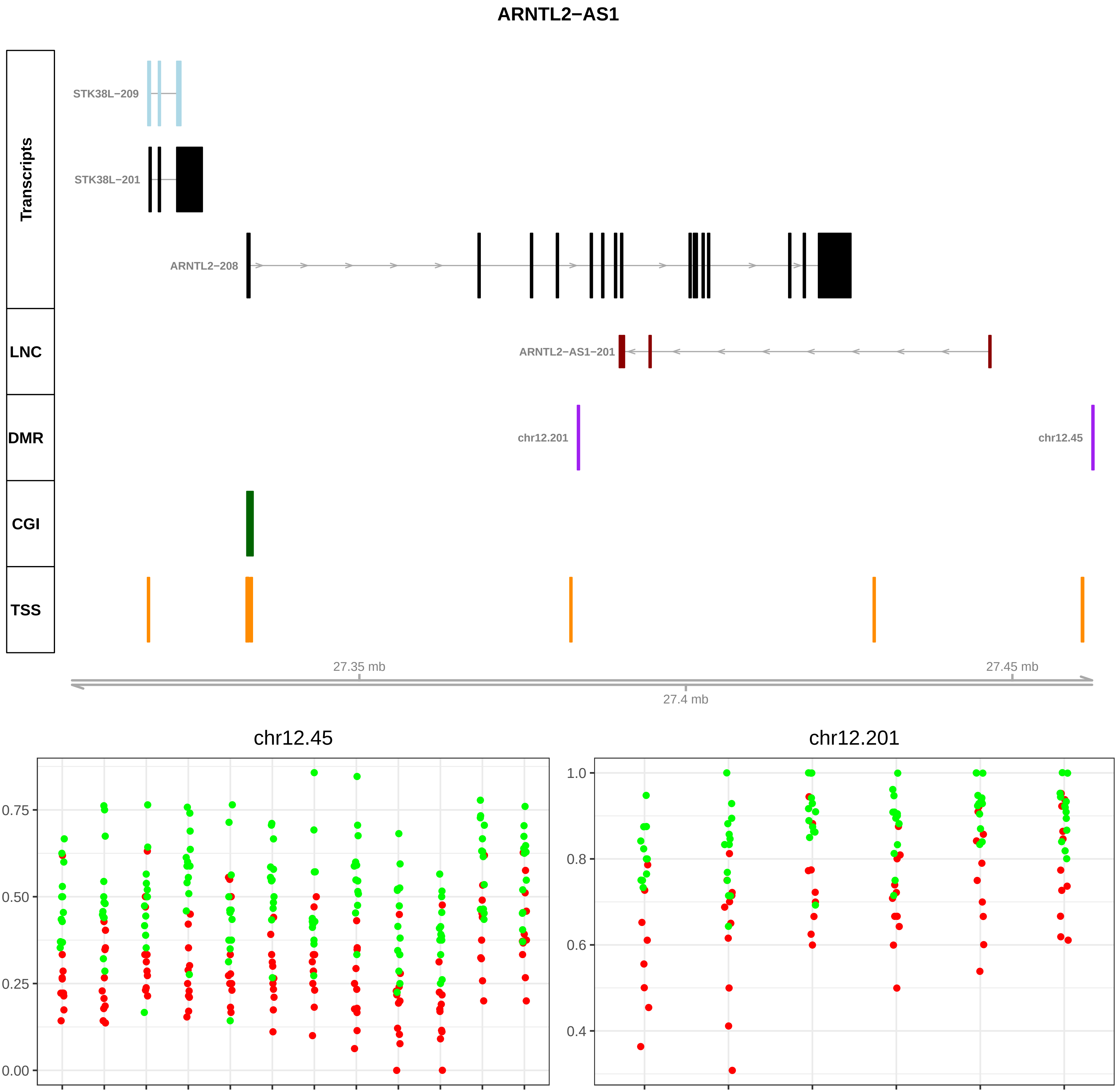
ARNTL2−AS1
Transcripts
STK38L−209
STK38L−201
ARNTL2−208
LNC
ARNTL2−AS1−201
DMR
chr12.201
chr12.45
CGI
TSS
27.35 mb
27.45 mb
27.4 mb
chr12.45
0.75
0.50
0.25
0.00
chr12.201
1.0
0.8
0.6
0.4

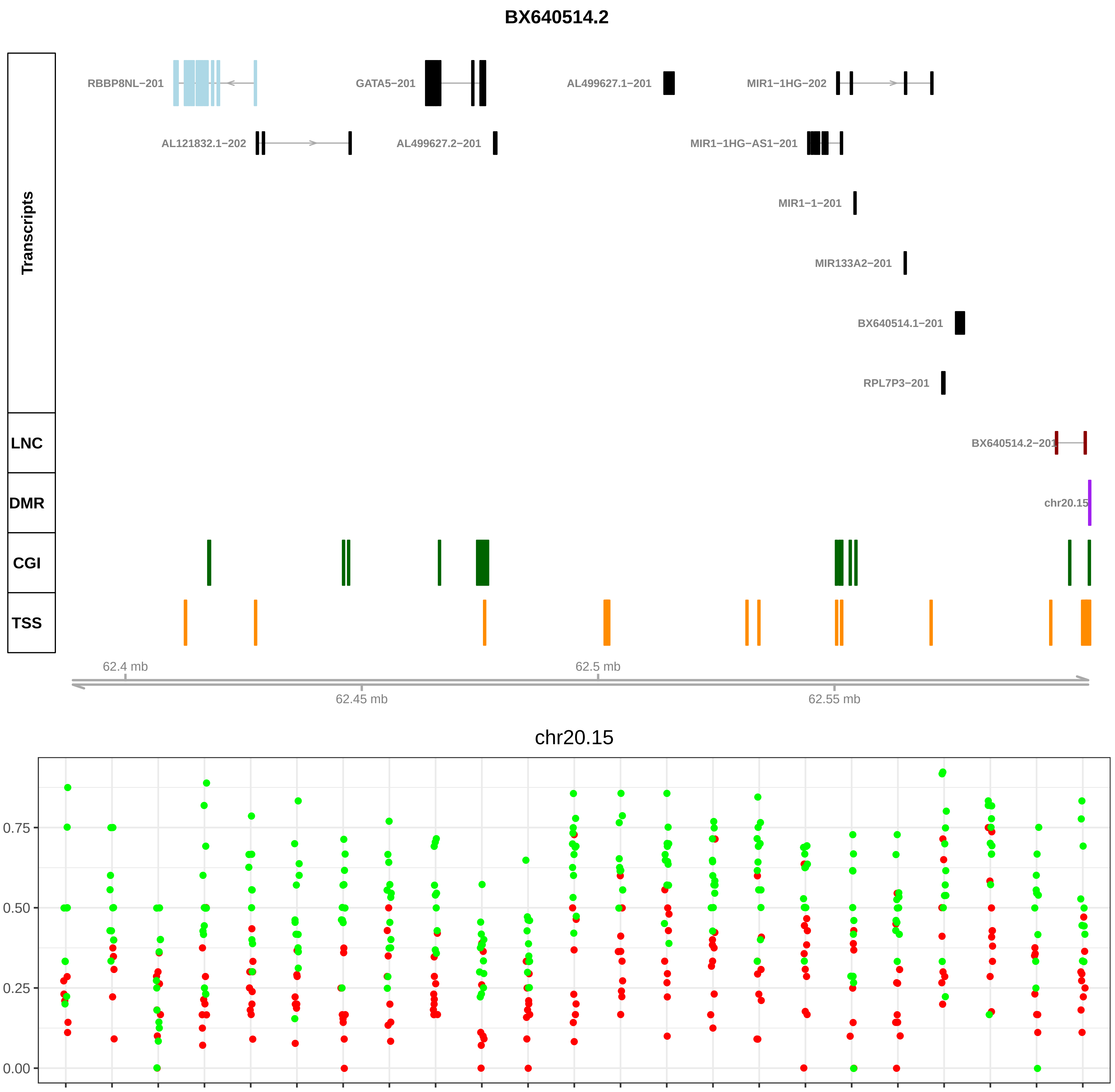
BX640514.2
Transcripts
LNC
DMR
CGI
TSS
RBBP8NL−201
GATA5−201
AL499627.1−201
MIR1−1HG−202
AL121832.1−202
AL499627.2−201
MIR1−1HG−AS1−201
MIR1−1−201
MIR133A2−201
BX640514.1−201
RPL7P3−201
BX640514.2−201
chr20.15
62.4 mb
62.5 mb
62.45 mb
62.55 mb
chr20.15
0.75
0.50
0.25
0.00

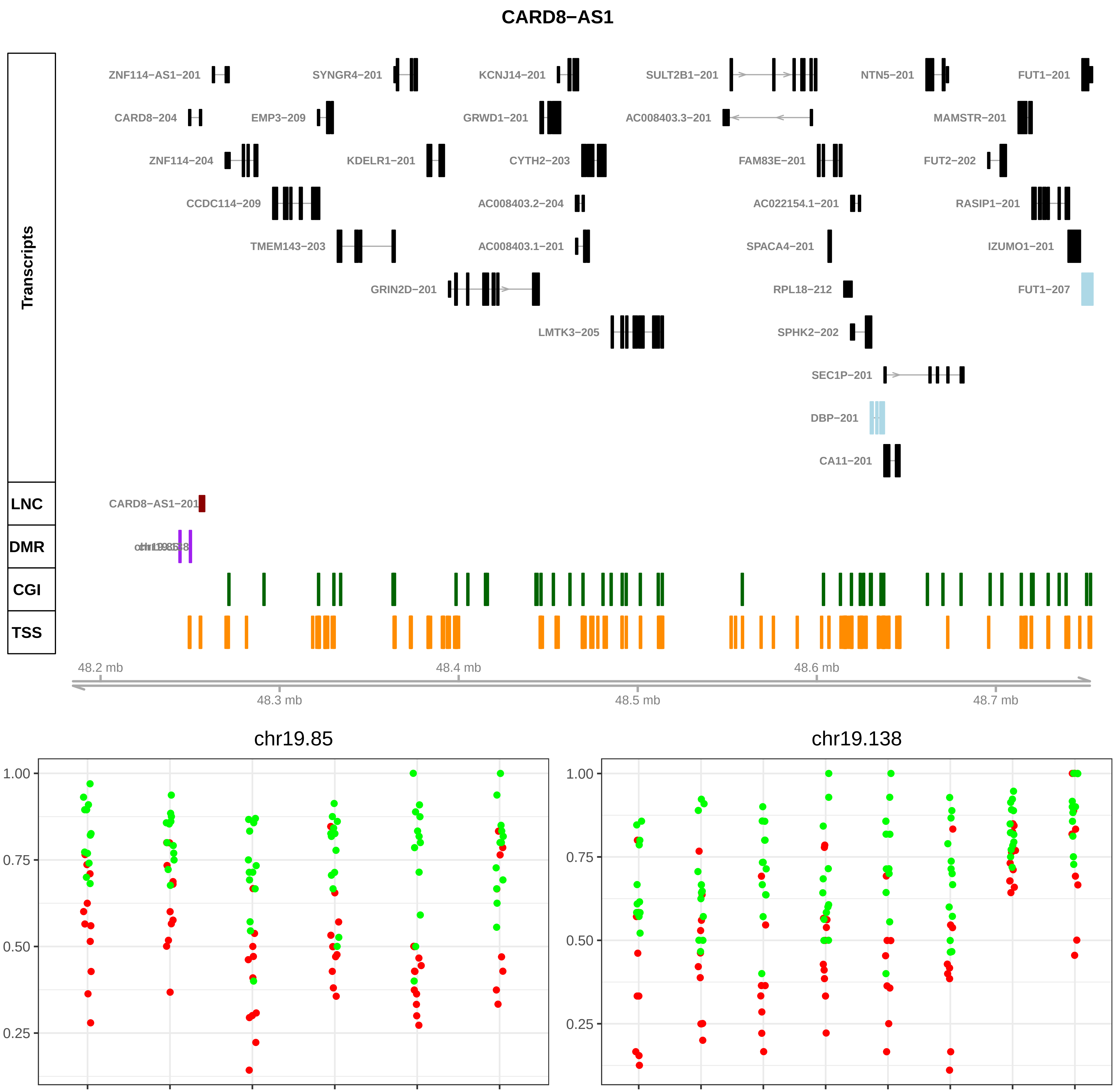

CARD8−AS1
Transcripts
ZNF114−AS1−201
SYNGR4−201
KCNJ14−201
SULT2B1−201
NTN5−201
FUT1−201
CARD8−204
EMP3−209
GRWD1−201
AC008403.3−201
MAMSTR−201
ZNF114−204
KDELR1−201
CYTH2−203
FAM83E−201
FUT2−202
CCDC114−209
AC008403.2−204
AC022154.1−201
RASIP1−201
TMEM143−203
AC008403.1−201
SPACA4−201
IZUMO1−201
GRIN2D−201
RPL18−212
FUT1−207
LMTK3−205
SPHK2−202
SEC1P−201
DBP−201
CA11−201
LNC
CARD8−AS1−201
DMR
CGI
TSS
48.2 mb
48.4 mb
48.6 mb
48.3 mb
48.5 mb
48.7 mb
chr19.85
1.00
0.75
0.50
0.25
chr19.138
1.00
0.75
0.50
0.25

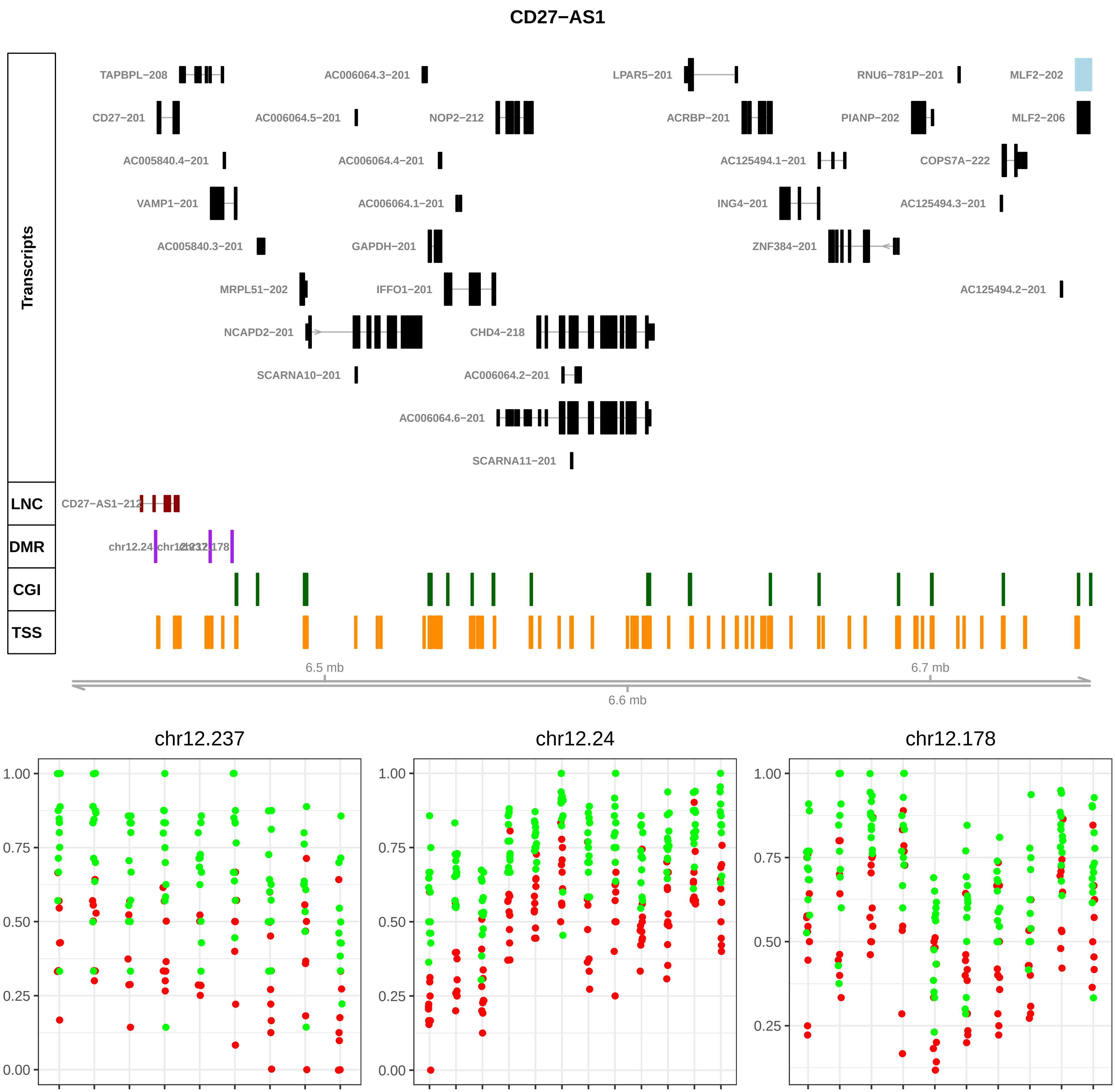

CD27−AS1
Transcripts
TAPBPL−208
AC006064.3−201
LPAR5−201
RNU6−781P−201
MLF2−202
CD27−201
AC006064.5−201
NOP2−212
ACRBP−201
PIANP−202
MLF2−206
AC005840.4−201
AC006064.4−201
AC125494.1−201
COPS7A−222
VAMP1−201
AC006064.1−201
ING4−201
AC125494.3−201
AC005840.3−201
GAPDH−201
ZNF384−201
MRPL51−202
IFFO1−201
AC125494.2−201
NCAPD2−201
CHD4−218
SCARNA10−201
AC006064.2−201
AC006064.6−201
SCARNA11−201
LNC
CD27−AS1−212
DMR
chr12.24
CGI
TSS
6.5 mb
6.7 mb
6.6 mb
chr12.237
chr12.24
chr12.178
1.00
0.75
0.50
0.25
0.00

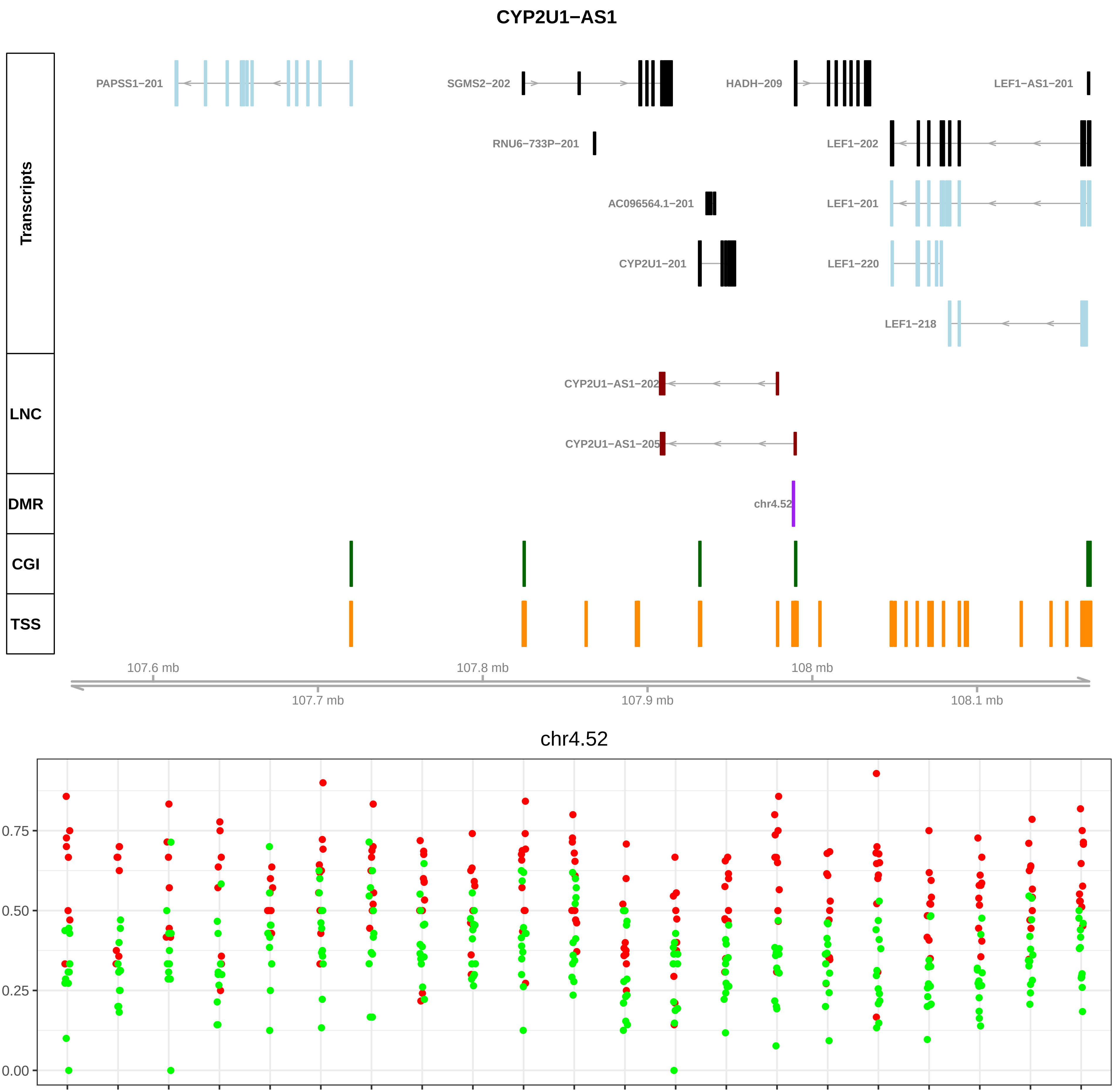
CYP2U1−AS1
Transcripts
LNC
DMR
CGI
TSS
PAPSS1−201
SGMS2−202
HADH−209
LEF1−AS1−201
RNU6−733P−201
LEF1−202
AC096564.1−201
LEF1−201
CYP2U1−201
LEF1−220
LEF1−218
CYP2U1−AS1−202
CYP2U1−AS1−205
chr4.52
107.6 mb
107.8 mb
108 mb
107.7 mb
107.9 mb
108.1 mb
chr4.52
0.75
0.50
0.25
0.00

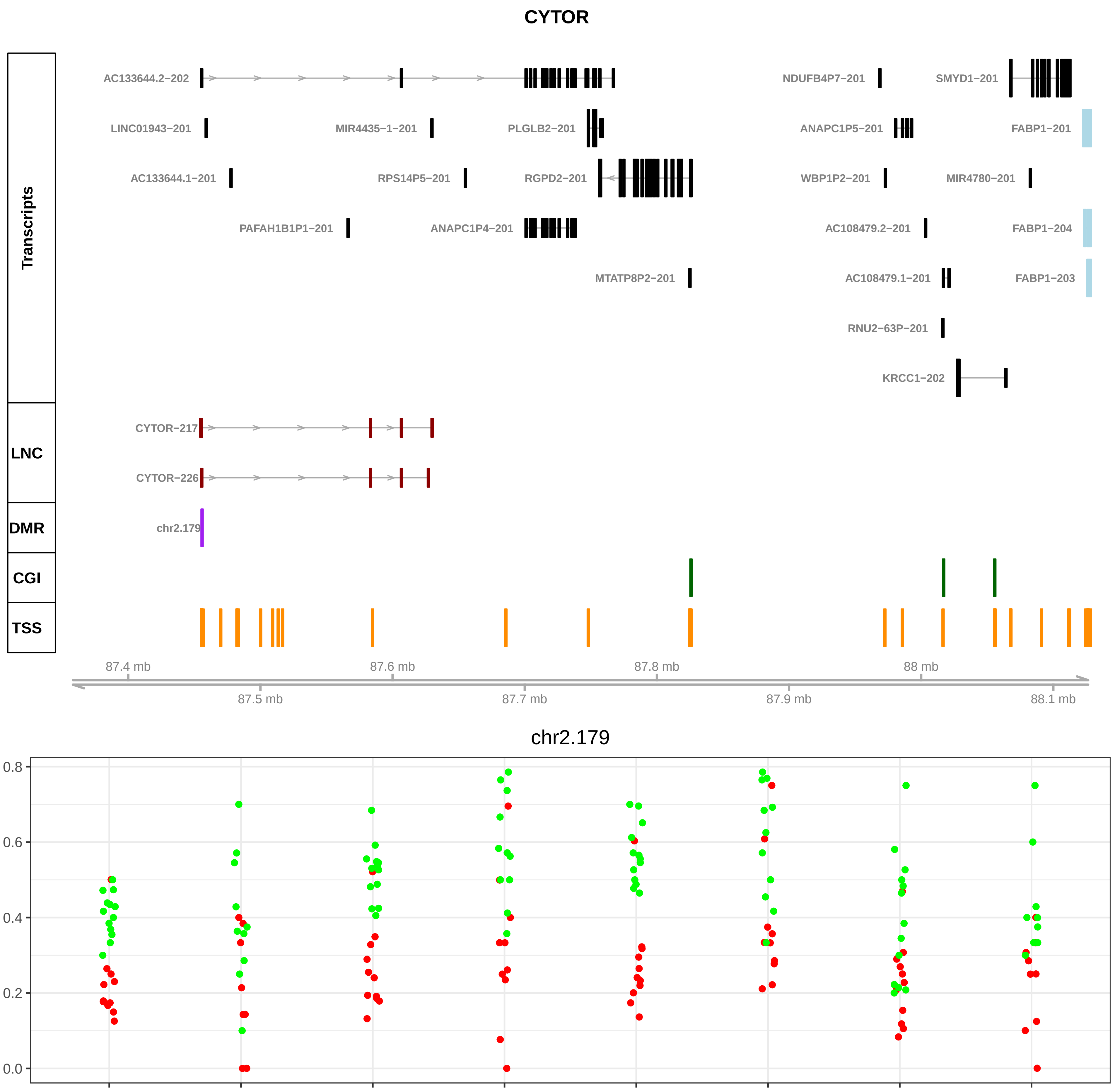

CYTOR
Transcripts
AC133644.2−202
NDUFB4P7−201
SMYD1−201
LINC01943−201
MIR4435−1−201
PLGLB2−201
ANAPC1P5−201
FABP1−201
AC133644.1−201
RPS14P5−201
RGPD2−201
WBP1P2−201
MIR4780−201
PAFAH1B1P1−201
ANAPC1P4−201
AC108479.2−201
FABP1−204
MTATP8P2−201
AC108479.1−201
FABP1−203
RNU2−63P−201
KRCC1−202
LNC
CYTOR−217
CYTOR−226
DMR
chr2.179
CGI
TSS
87.4 mb
87.6 mb
87.8 mb
88 mb
87.5 mb
87.7 mb
87.9 mb
88.1 mb
chr2.179
0.8
0.6
0.4
0.2
0.0

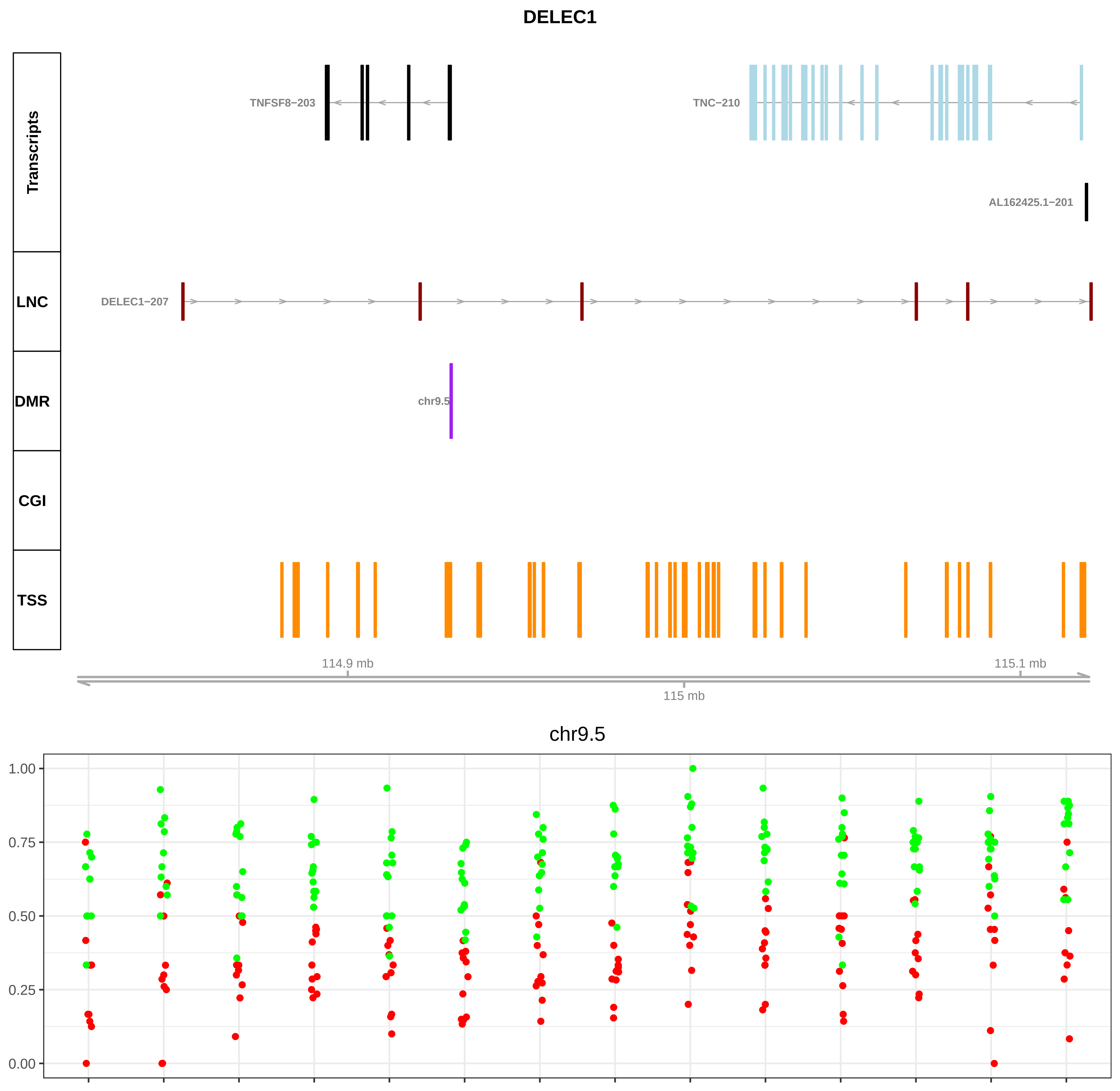
DELEC1
Transcripts
LNC
DMR
CGI
TSS
TNFSF8−203
TNC−210
AL162425.1−201
DELEC1−207
chr9.5
114.9 mb
115 mb
115.1 mb
chr9.5
1.00
0.75
0.50
0.25
0.00

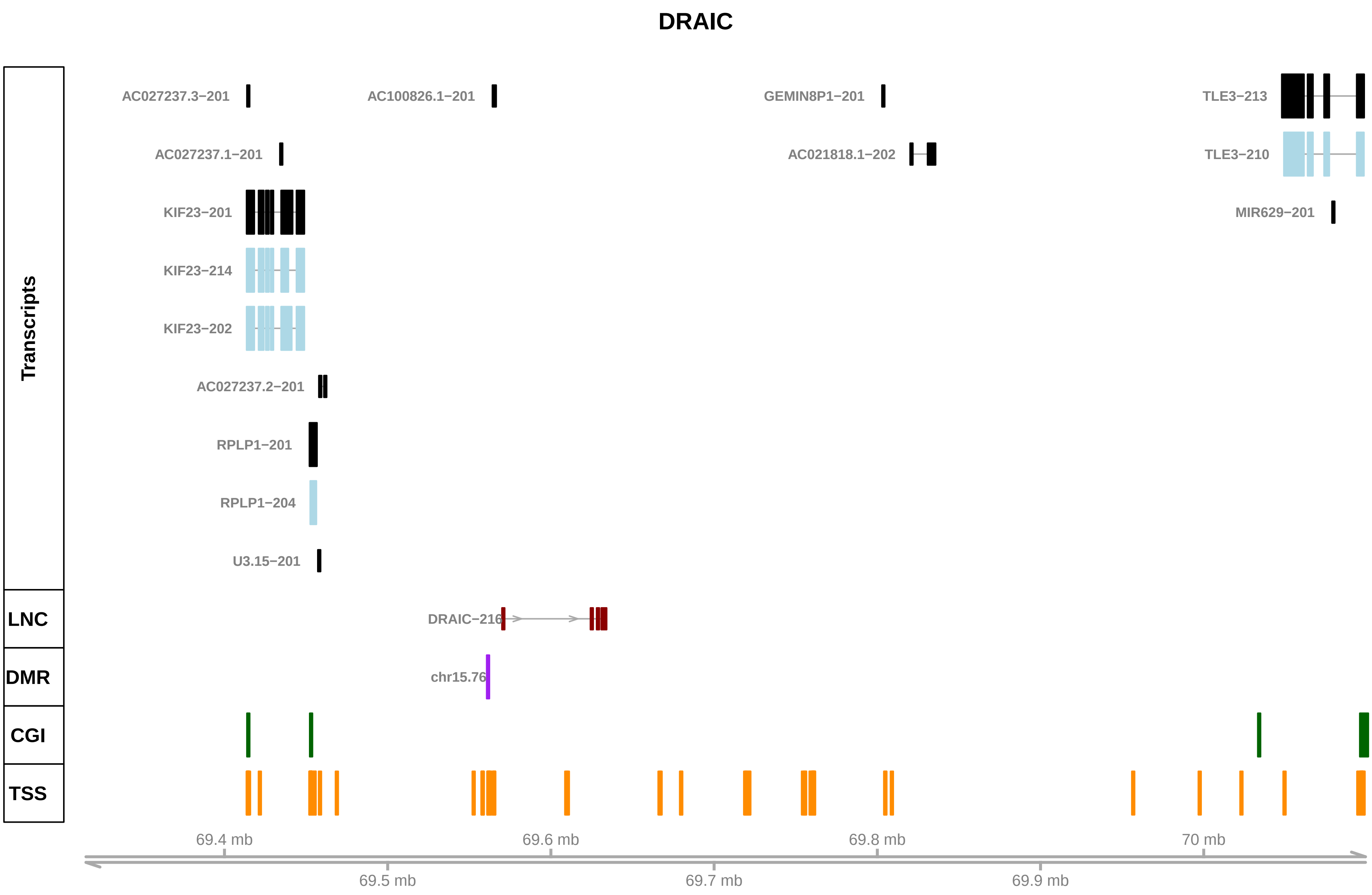

DRAIC
Transcripts
AC027237.3−201
AC100826.1−201
GEMIN8P1−201
TLE3−213
AC027237.1−201
AC021818.1−202
TLE3−210
KIF23−201
MIR629−201
KIF23−214
KIF23−202
AC027237.2−201
RPLP1−201
RPLP1−204
U3.15−201
LNC
DRAIC−216
DMR
chr15.76
CGI
TSS
69.4 mb
69.6 mb
69.8 mb
70 mb
69.5 mb
69.7 mb
69.9 mb

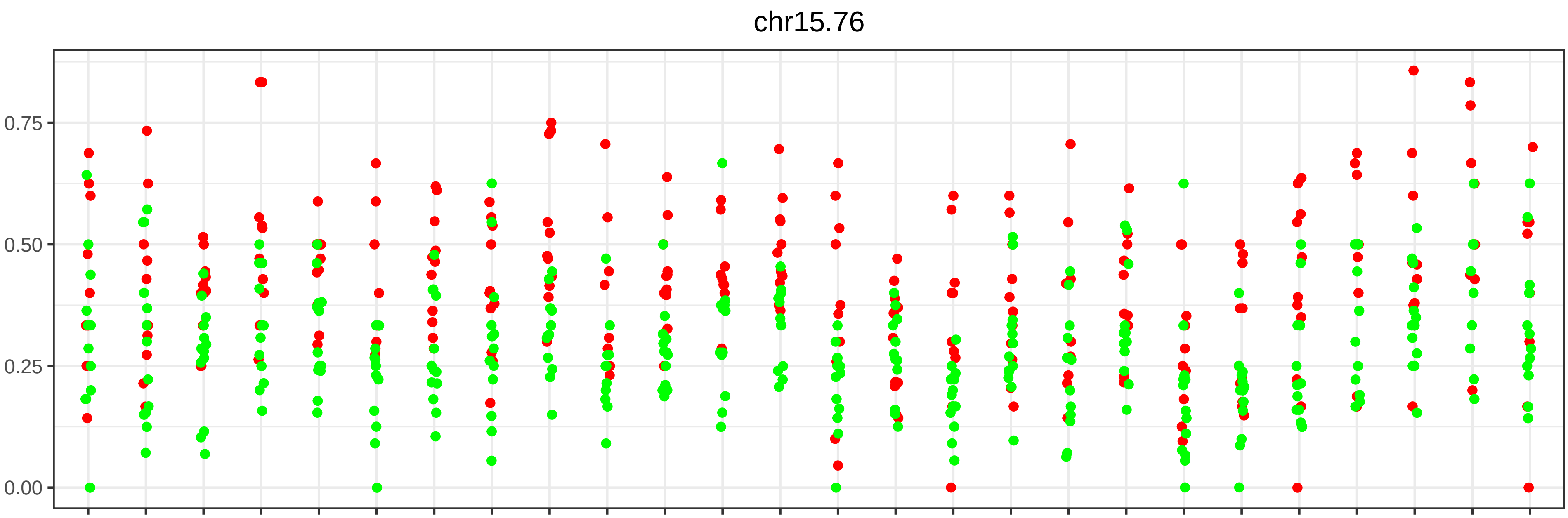

chr15.76
0.75
0.50
0.25
0.00

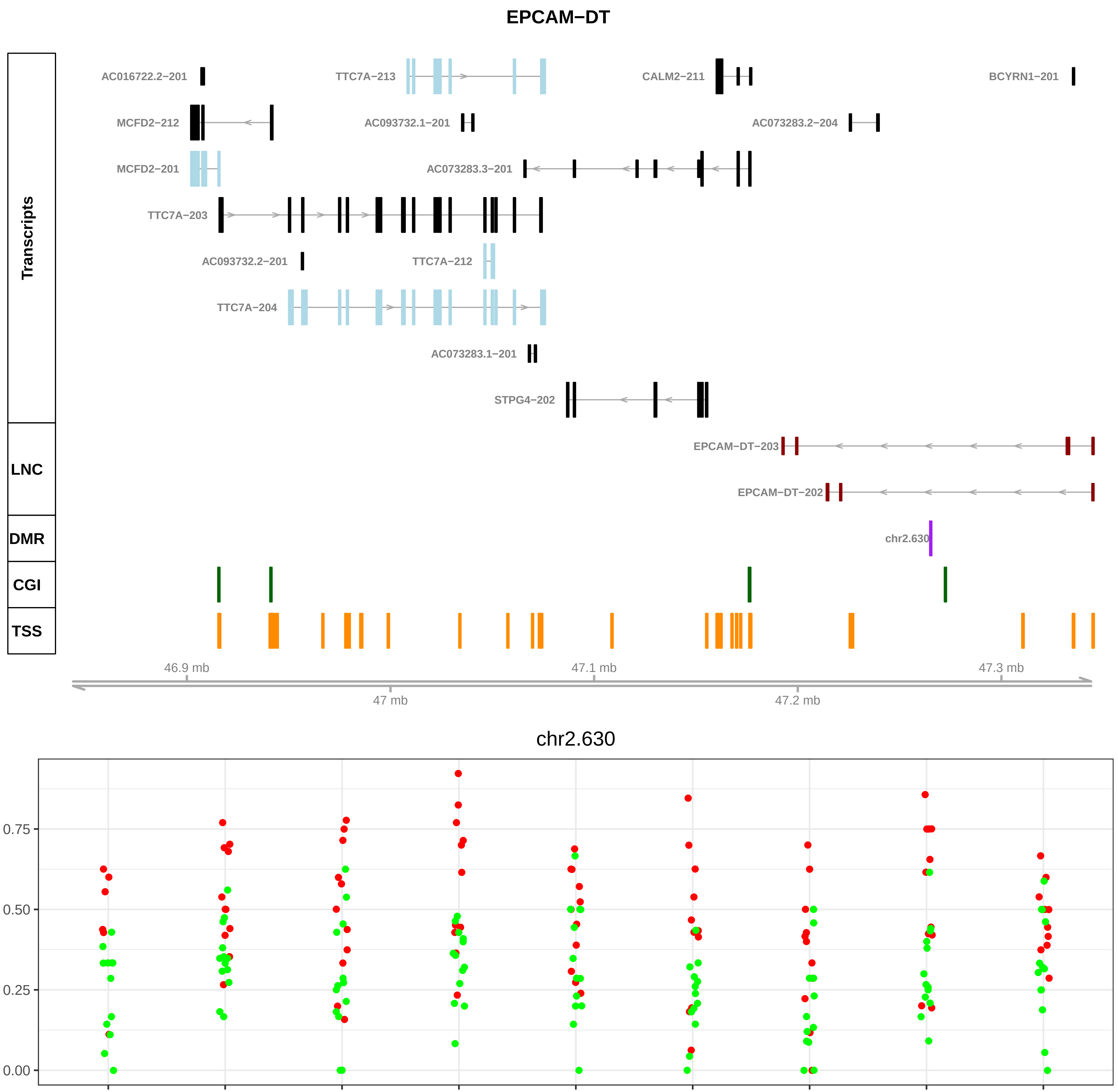
EPCAM−DT
Transcripts
LNC
DMR
CGI
TSS
AC016722.2−201
TTC7A−213
CALM2−211
BCYRN1−201
MCFD2−212
AC093732.1−201
AC073283.2−204
MCFD2−201
AC073283.3−201
TTC7A−203
AC093732.2−201
TTC7A−212
TTC7A−204
AC073283.1−201
STPG4−202
EPCAM−DT−203
EPCAM−DT−202
chr2.630
46.9 mb
47 mb
47.1 mb
47.2 mb
47.3 mb
chr2.630
0.00
0.25
0.50
0.75

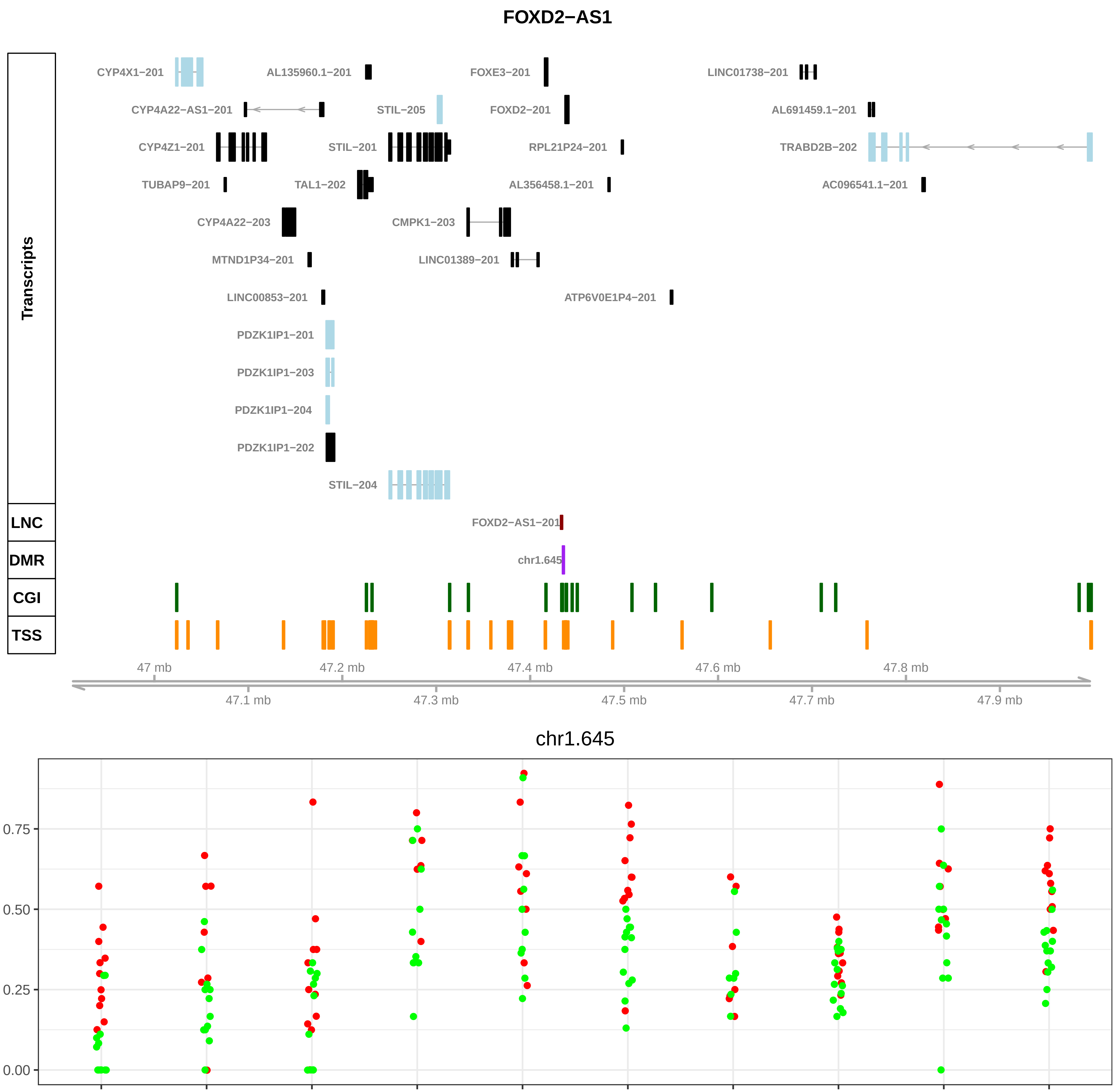
FOXD2−AS1
Transcripts
LNC
DMR
CGI
TSS
CYP4X1−201
AL135960.1−201
FOXE3−201
LINC01738−201
CYP4A22−AS1−201
STIL−205
FOXD2−201
AL691459.1−201
CYP4Z1−201
STIL−201
RPL21P24−201
TRABD2B−202
TUBAP9−201
TAL1−202
AL356458.1−201
AC096541.1−201
CYP4A22−203
CMPK1−203
MTND1P34−201
LINC01389−201
LINC00853−201
ATP6V0E1P4−201
PDZK1IP1−201
PDZK1IP1−203
PDZK1IP1−204
PDZK1IP1−202
STIL−204
FOXD2−AS1−201
chr1.645
47 mb
47.1 mb
47.2 mb
47.3 mb
47.4 mb
47.5 mb
47.6 mb
47.7 mb
47.8 mb
47.9 mb
chr1.645
0.75
0.50
0.25
0.00

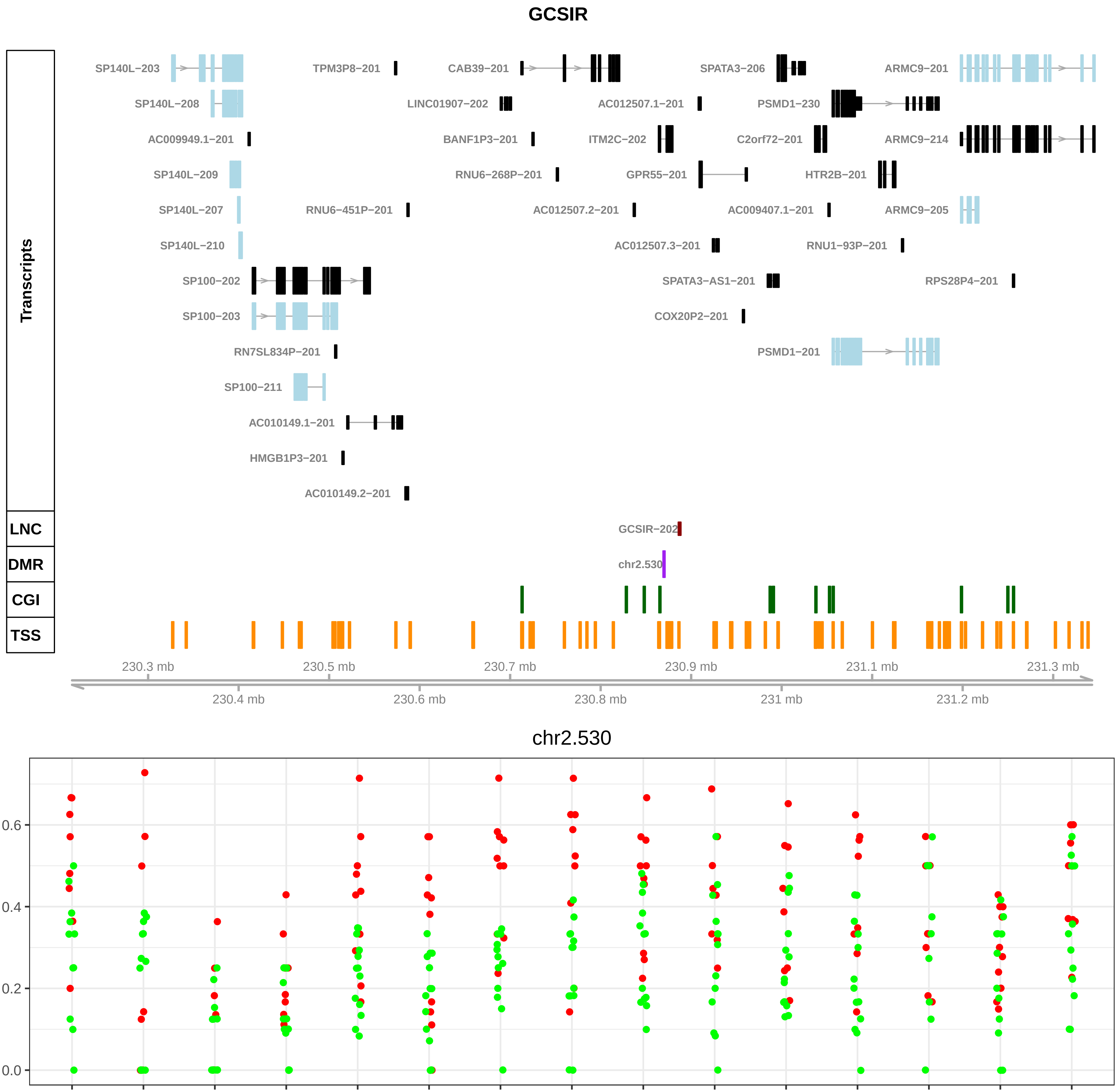

GCSIR
Transcripts
LNC
DMR
CGI
TSS
SP140L−203
TPM3P8−201
CAB39−201
SPATA3−206
ARMC9−201
SP140L−208
LINC01907−202
AC012507.1−201
PSMD1−230
AC009949.1−201
BANF1P3−201
ITM2C−202
C2orf72−201
ARMC9−214
SP140L−209
RNU6−268P−201
GPR55−201
HTR2B−201
SP140L−207
RNU6−451P−201
AC012507.2−201
AC009407.1−201
ARMC9−205
SP140L−210
AC012507.3−201
RNU1−93P−201
SP100−202
SPATA3−AS1−201
RPS28P4−201
SP100−203
COX20P2−201
RN7SL834P−201
PSMD1−201
SP100−211
AC010149.1−201
HMGB1P3−201
AC010149.2−201
GCSIR−202
chr2.530
230.3 mb
230.5 mb
230.7 mb
230.9 mb
231.1 mb
231.3 mb
230.4 mb
230.6 mb
230.8 mb
231 mb
231.2 mb
chr2.530
0.6
0.4
0.2
0.0

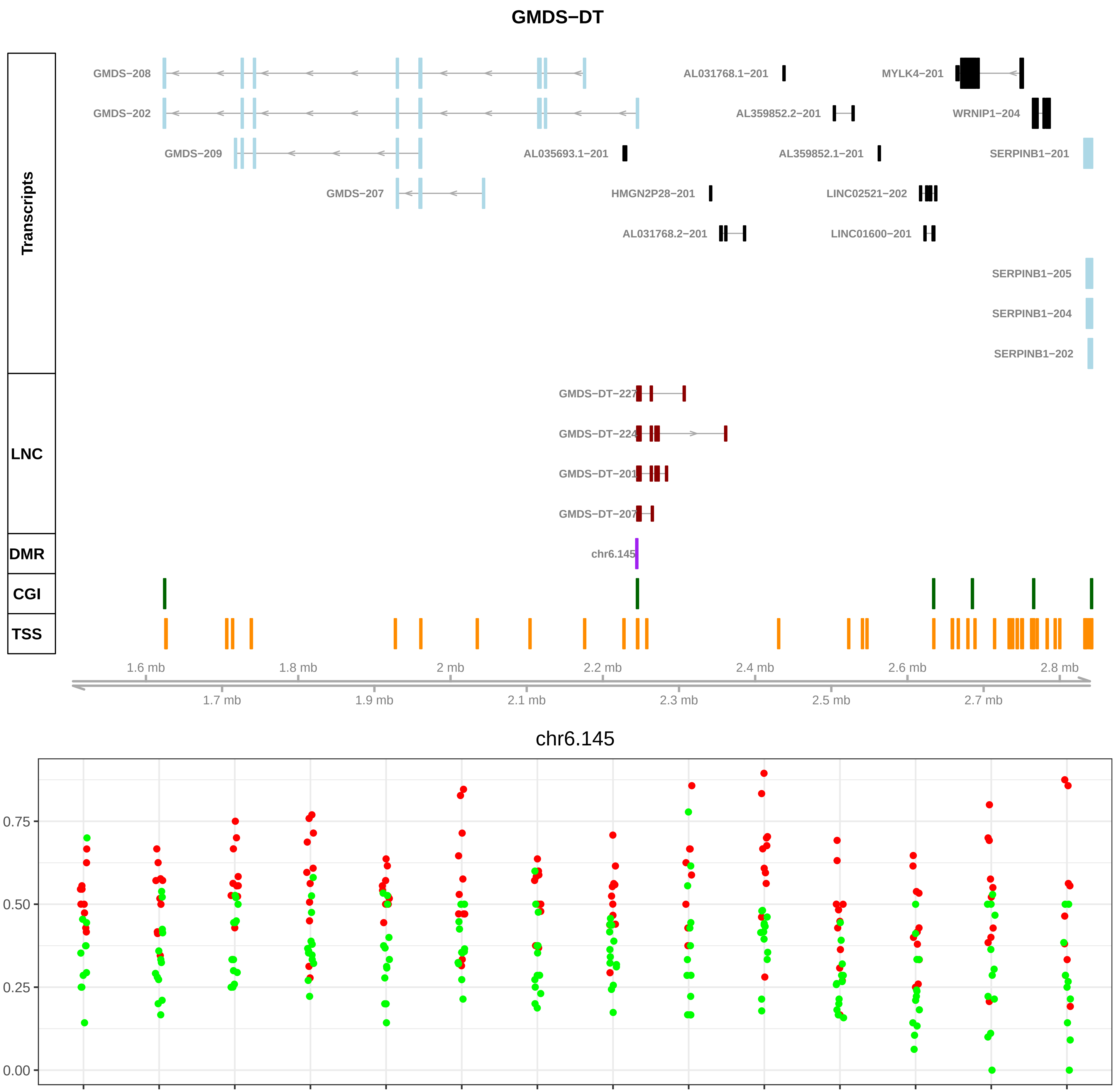
GMDS−DT
Transcripts
GMDS−208
GMDS−202
GMDS−209
GMDS−207
AL031768.1−201
AL359852.2−201
AL035693.1−201
AL359852.1−201
HMGN2P28−201
LINC02521−202
AL031768.2−201
LINC01600−201
MYLK4−201
WRNIP1−204
SERPINB1−201
SERPINB1−205
SERPINB1−204
SERPINB1−202
LNC
GMDS−DT−227
GMDS−DT−224
GMDS−DT−201
GMDS−DT−207
DMR
chr6.145
CGI
TSS
1.6 mb
1.7 mb
1.8 mb
1.9 mb
2 mb
2.1 mb
2.2 mb
2.3 mb
2.4 mb
2.5 mb
2.6 mb
2.7 mb
2.8 mb
chr6.145
0.00
0.25
0.50
0.75

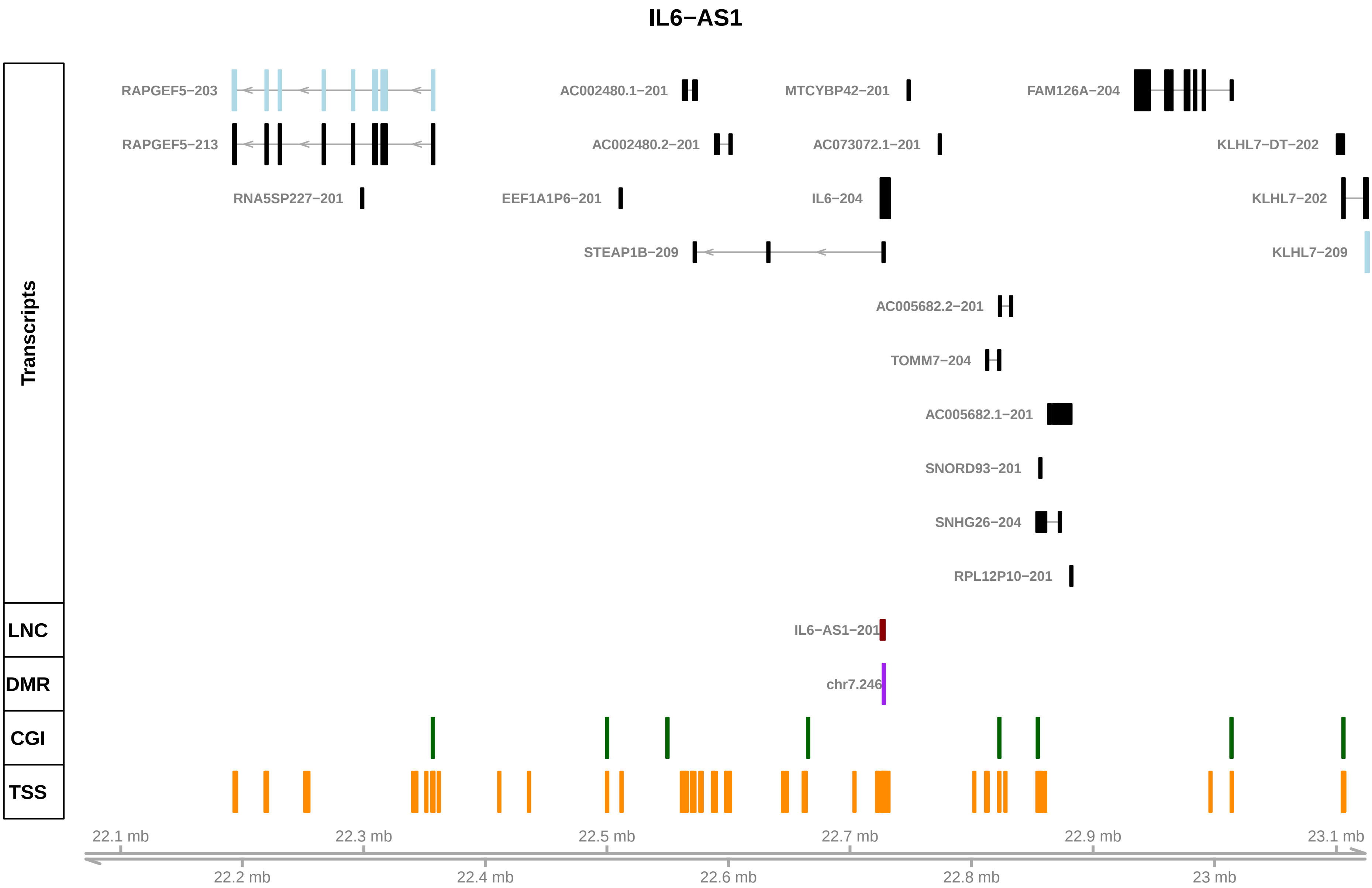
IL6−AS1
Transcripts
RAPGEF5−203
RAPGEF5−213
RNA5SP227−201
AC002480.1−201
AC002480.2−201
EEF1A1P6−201
STEAP1B−209
MTCYBP42−201
AC073072.1−201
IL6−204
AC005682.2−201
TOMM7−204
AC005682.1−201
SNORD93−201
SNHG26−204
RPL12P10−201
FAM126A−204
KLHL7−DT−202
KLHL7−202
KLHL7−209
LNC
IL6−AS1−201
DMR
chr7.246
CGI
TSS
22.1 mb
22.3 mb
22.5 mb
22.7 mb
22.9 mb
23.1 mb
22.2 mb
22.4 mb
22.6 mb
22.8 mb
23 mb

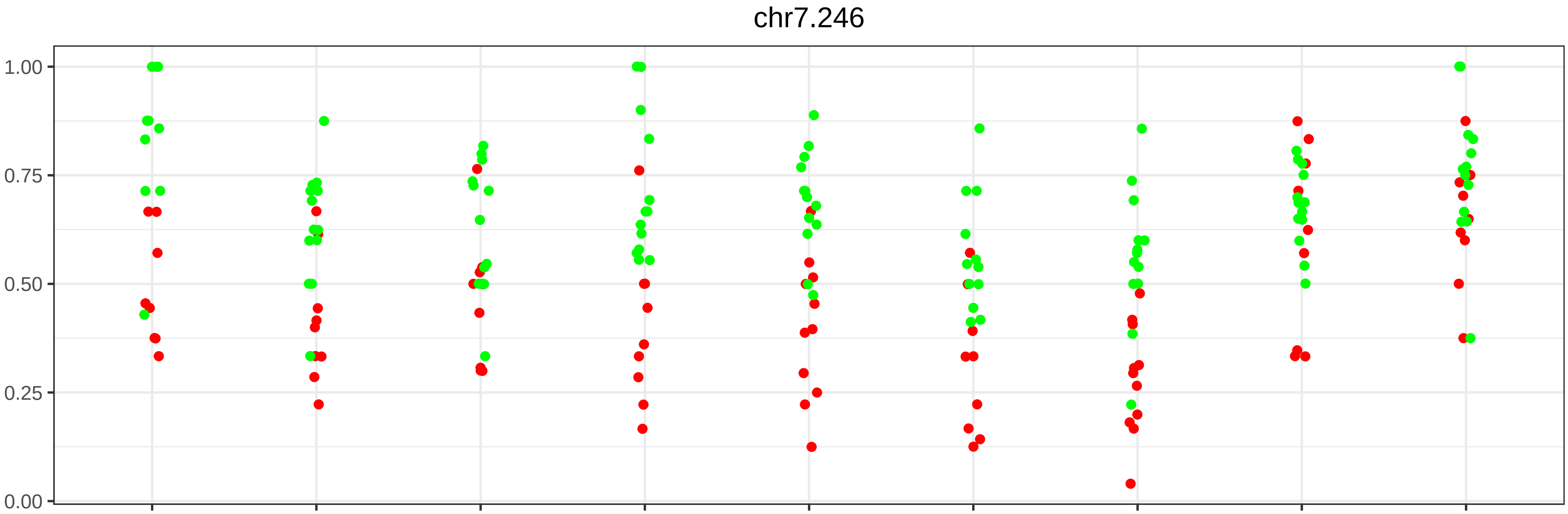
chr7.246
1.00
0.75
0.50
0.25
0.00

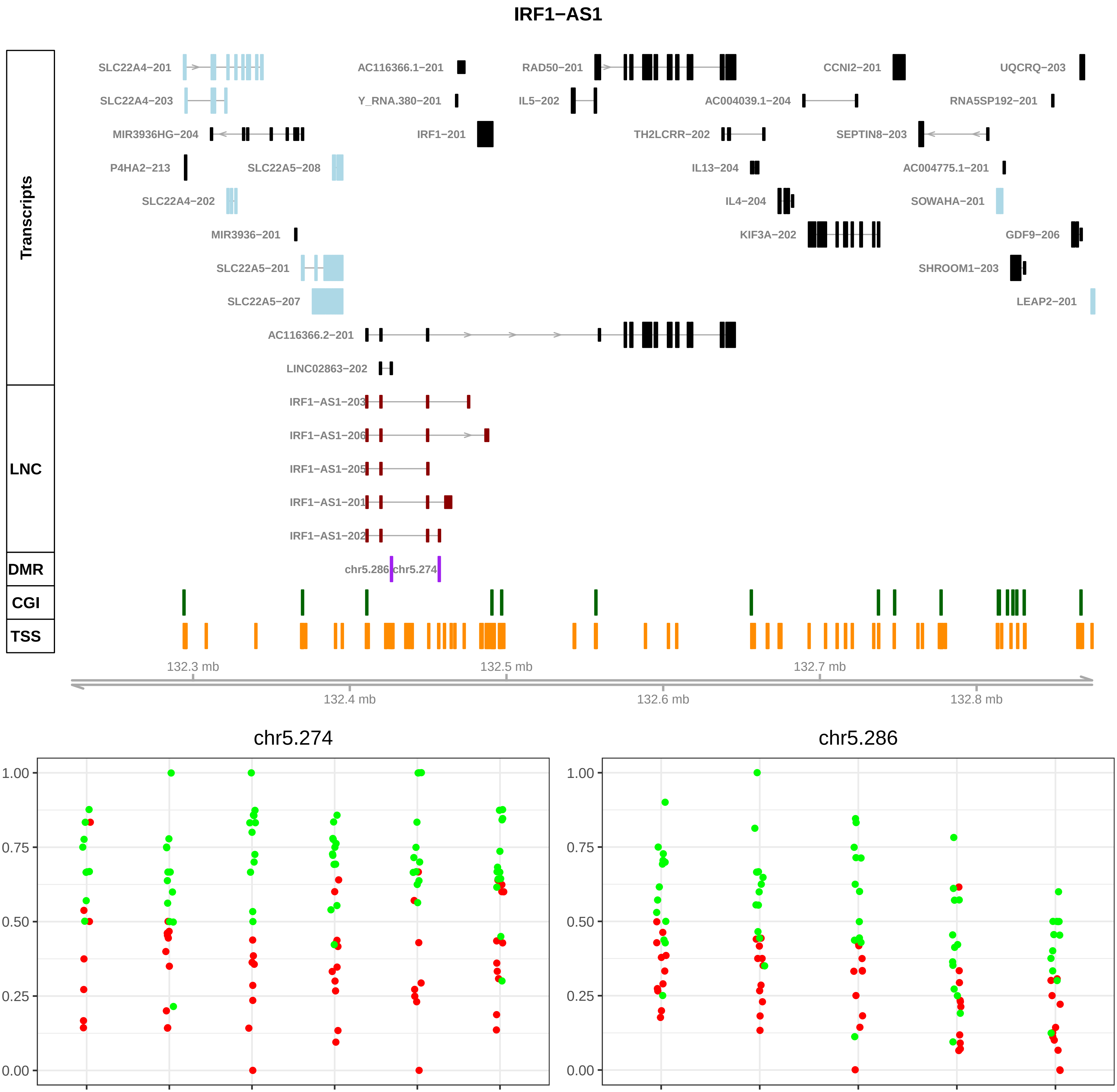

IRF1−AS1
Transcripts
LNC
DMR
CGI
TSS
SLC22A4−201
SLC22A4−203
MIR3936HG−204
P4HA2−213
SLC22A4−202
MIR3936−201
SLC22A5−201
SLC22A5−207
AC116366.1−201
Y_RNA.380−201
IRF1−201
SLC22A5−208
RAD50−201
IL5−202
TH2LCRR−202
IL13−204
IL4−204
KIF3A−202
AC004039.1−204
CCNI2−201
SEPTIN8−203
AC004775.1−201
SOWAHA−201
SHROOM1−203
UQCRQ−203
RNA5SP192−201
GDF9−206
LEAP2−201
AC116366.2−201
LINC02863−202
IRF1−AS1−203
IRF1−AS1−206
IRF1−AS1−205
IRF1−AS1−201
IRF1−AS1−202
chr5.286
chr5.274
132.3 mb
132.5 mb
132.7 mb
132.4 mb
132.6 mb
132.8 mb
chr5.274
chr5.286
1.00
0.75
0.50
0.25
0.00

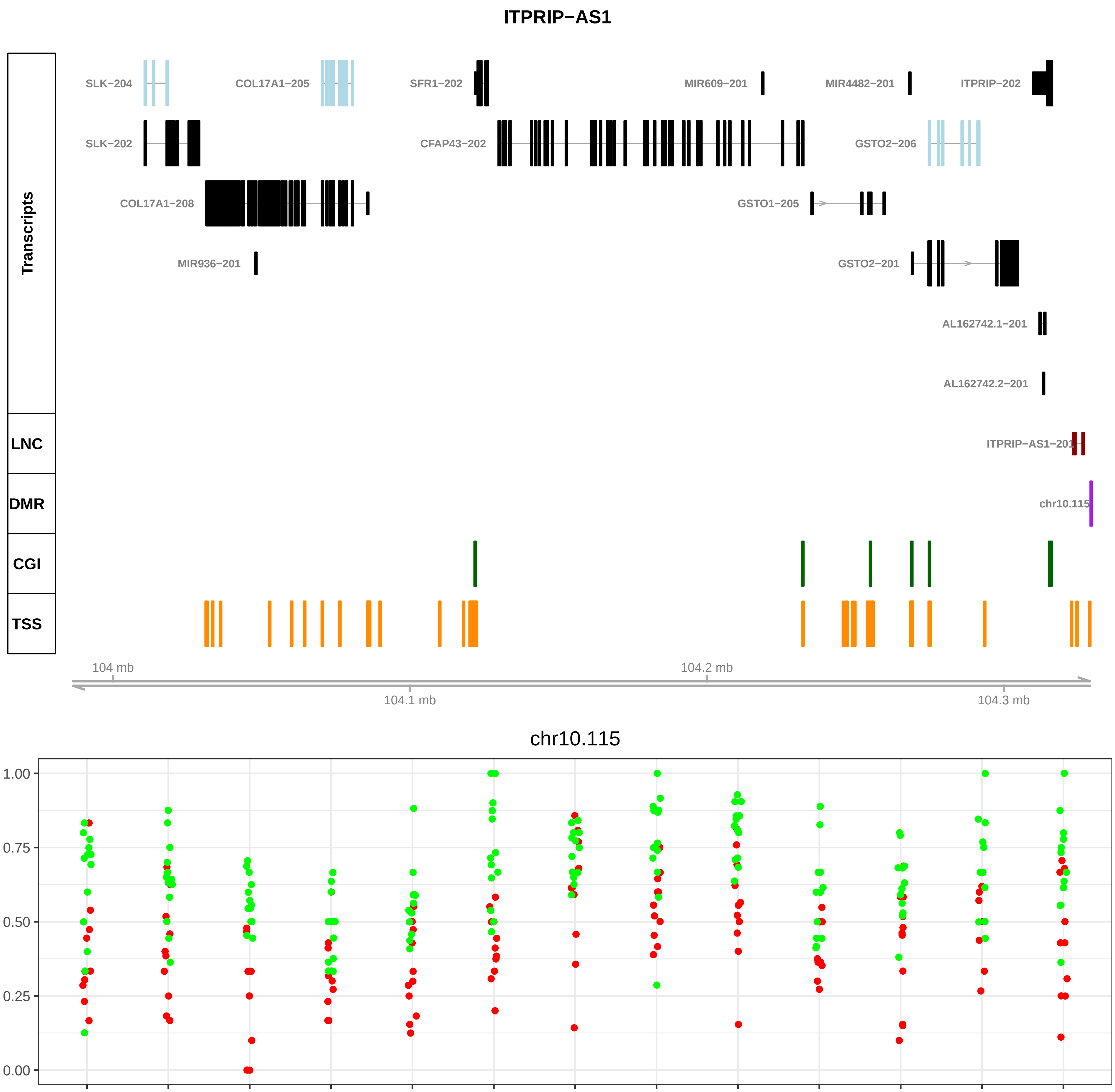
ITPRIP−AS1
Transcripts
SLK−204
COL17A1−205
SFR1−202
MIR609−201
MIR4482−201
ITPRIP−202
SLK−202
CFAP43−202
GSTO2−206
COL17A1−208
GSTO1−205
MIR936−201
GSTO2−201
AL162742.1−201
AL162742.2−201
LNC
ITPRIP−AS1−201
DMR
chr10.115
CGI
TSS
104 mb
104.1 mb
104.2 mb
104.3 mb
chr10.115
1.00
0.75
0.50
0.25
0.00

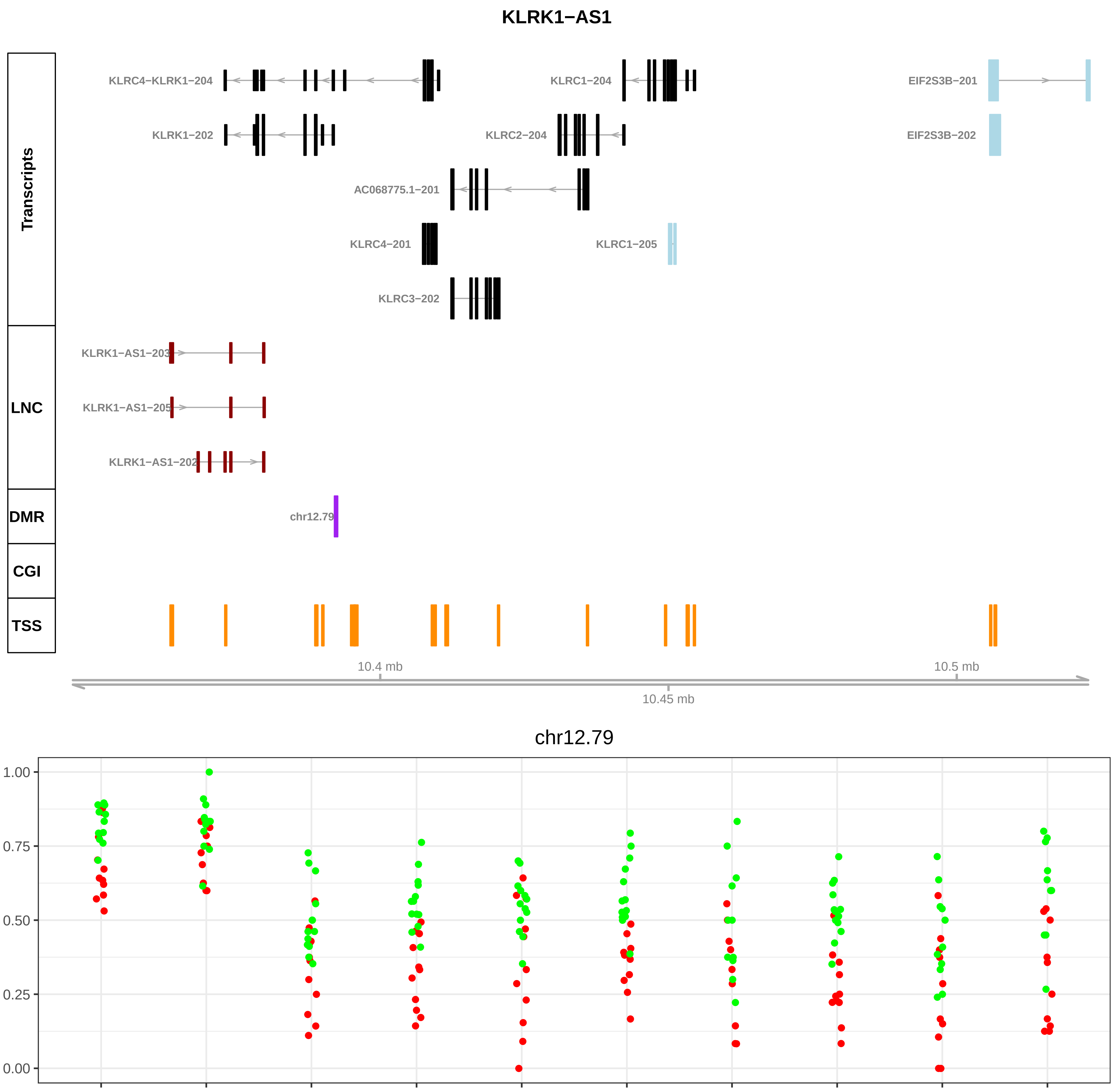
KLRK1−AS1
Transcripts
LNC
DMR
CGI
TSS
KLRC4−KLRK1−204
KLRC1−204
EIF2S3B−201
KLRK1−202
KLRC2−204
EIF2S3B−202
AC068775.1−201
KLRC4−201
KLRC1−205
KLRC3−202
KLRK1−AS1−203
KLRK1−AS1−205
KLRK1−AS1−202
chr12.79
10.4 mb
10.5 mb
10.45 mb
chr12.79
1.00
0.75
0.50
0.25
0.00

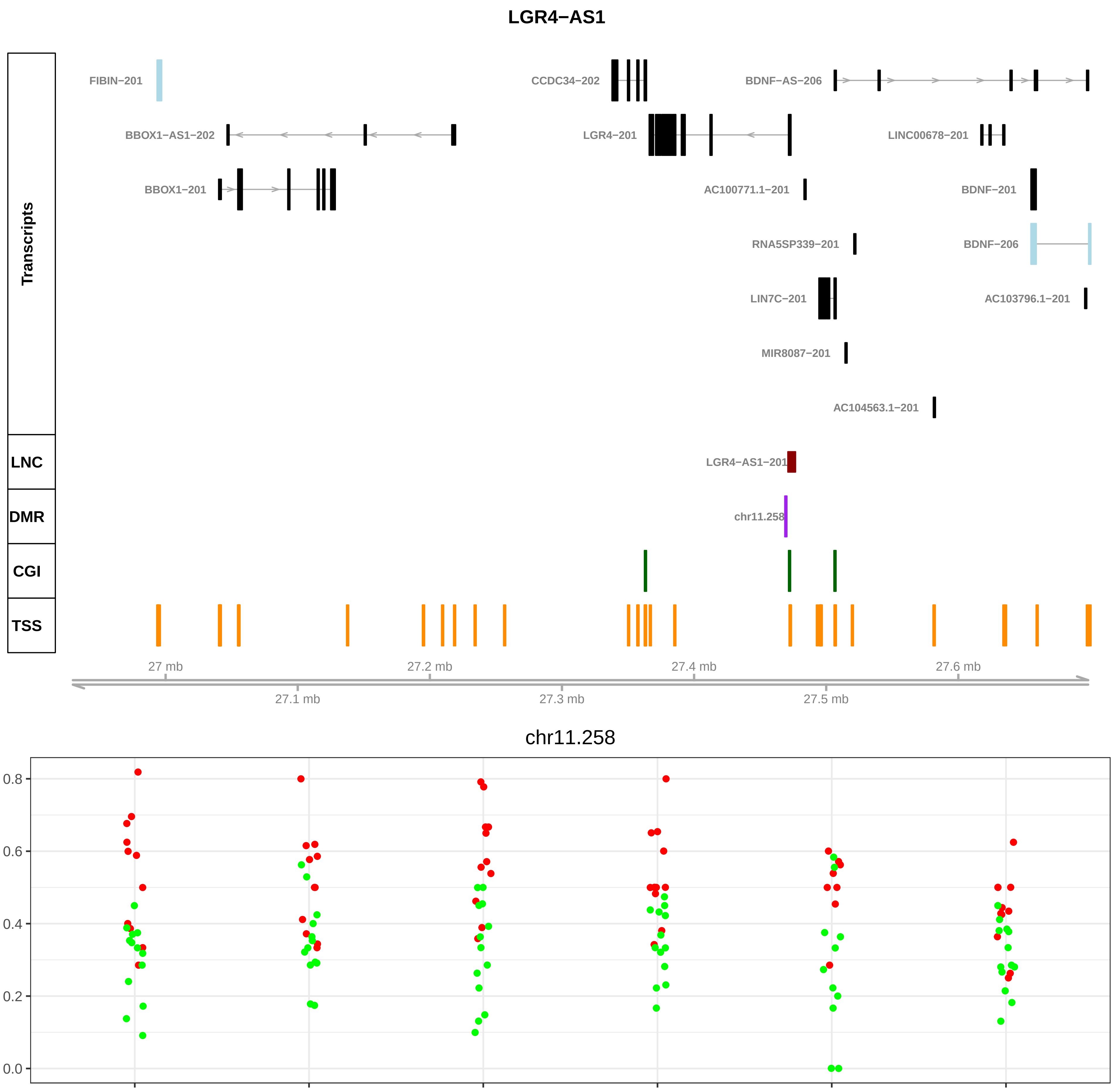
LGR4−AS1
Transcripts
LNC
DMR
CGI
TSS
FIBIN−201
CCDC34−202
BDNF−AS−206
BBOX1−AS1−202
LGR4−201
LINC00678−201
BBOX1−201
AC100771.1−201
BDNF−201
RNA5SP339−201
BDNF−206
LIN7C−201
AC103796.1−201
MIR8087−201
AC104563.1−201
LGR4−AS1−201
chr11.258
27 mb
27.1 mb
27.2 mb
27.3 mb
27.4 mb
27.5 mb
27.6 mb
chr11.258
0.0
0.2
0.4
0.6
0.8

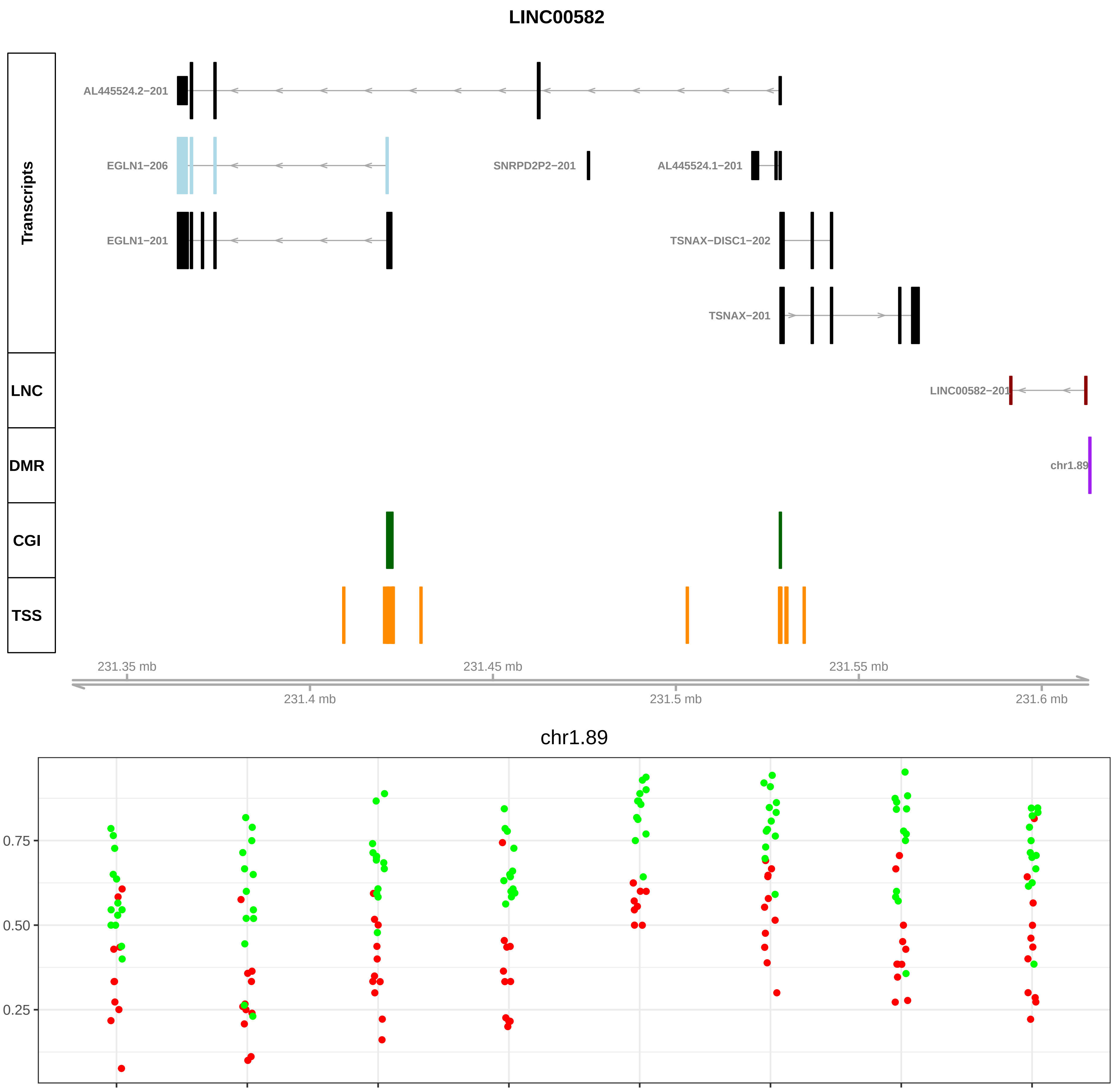
LINC00582
Transcripts
AL445524.2−201
EGLN1−206
SNRPD2P2−201
AL445524.1−201
EGLN1−201
TSNAX−DISC1−202
TSNAX−201
LNC
LINC00582−201
DMR
chr1.89
CGI
TSS
231.35 mb
231.45 mb
231.55 mb
231.4 mb
231.5 mb
231.6 mb
chr1.89
0.75
0.50
0.25

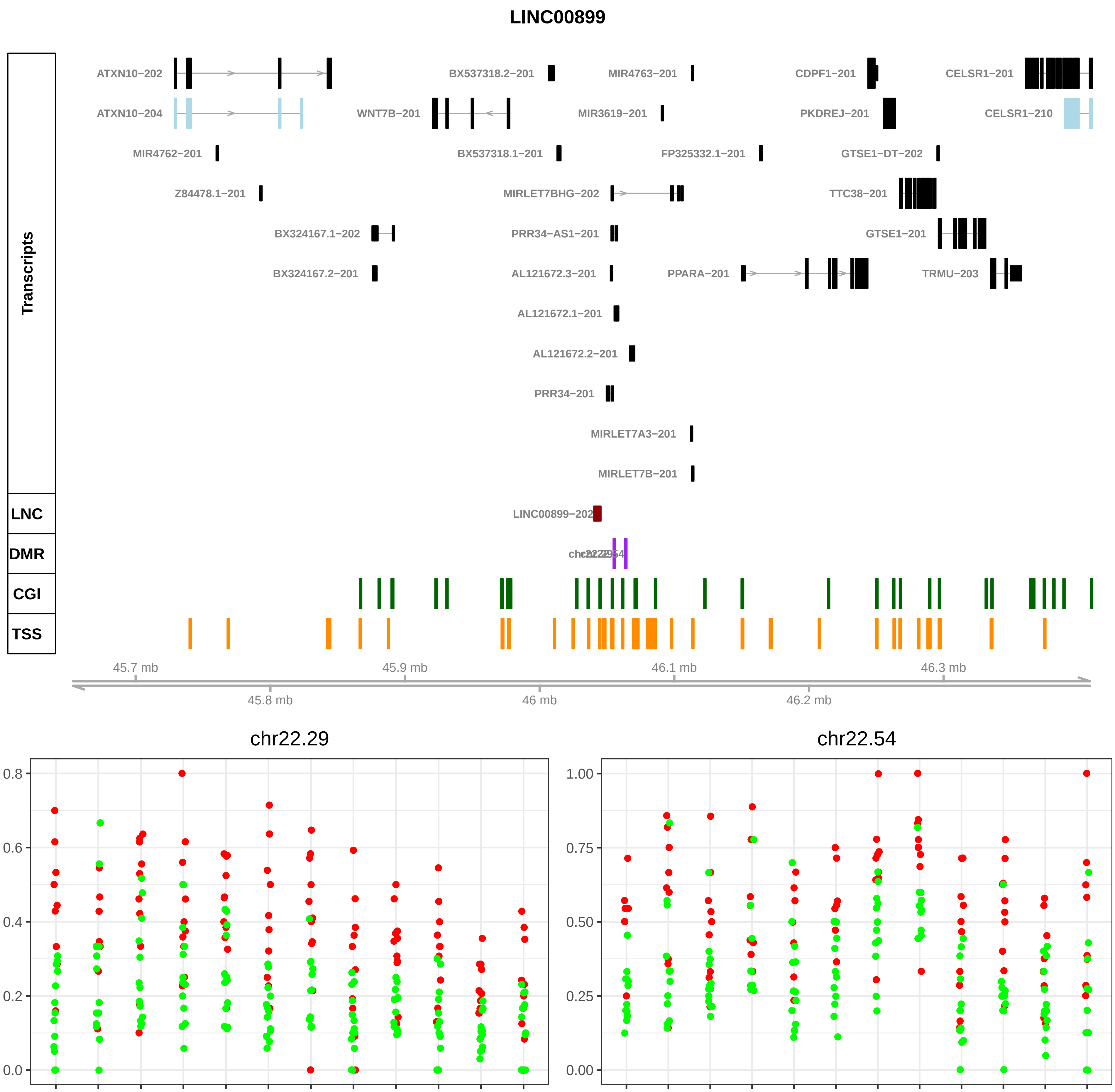
LINC00899
Transcripts
ATXN10−202
ATXN10−204
MIR4762−201
Z84478.1−201
BX324167.1−202
BX324167.2−201
BX537318.2−201
WNT7B−201
BX537318.1−201
MIRLET7BHG−202
PRR34−AS1−201
AL121672.3−201
AL121672.1−201
AL121672.2−201
PRR34−201
MIRLET7A3−201
MIRLET7B−201
MIR4763−201
MIR3619−201
FP325332.1−201
PPARA−201
CDPF1−201
PKDREJ−201
GTSE1−DT−202
TTC38−201
GTSE1−201
TRMU−203
CELSR1−201
CELSR1−210
LNC
LINC00899−202
DMR
CGI
TSS
45.7 mb
45.8 mb
45.9 mb
46 mb
46.1 mb
46.2 mb
46.3 mb
chr22.29
chr22.54
0.0
0.2
0.4
0.6
0.8
0.00
0.25
0.50
0.75
1.00

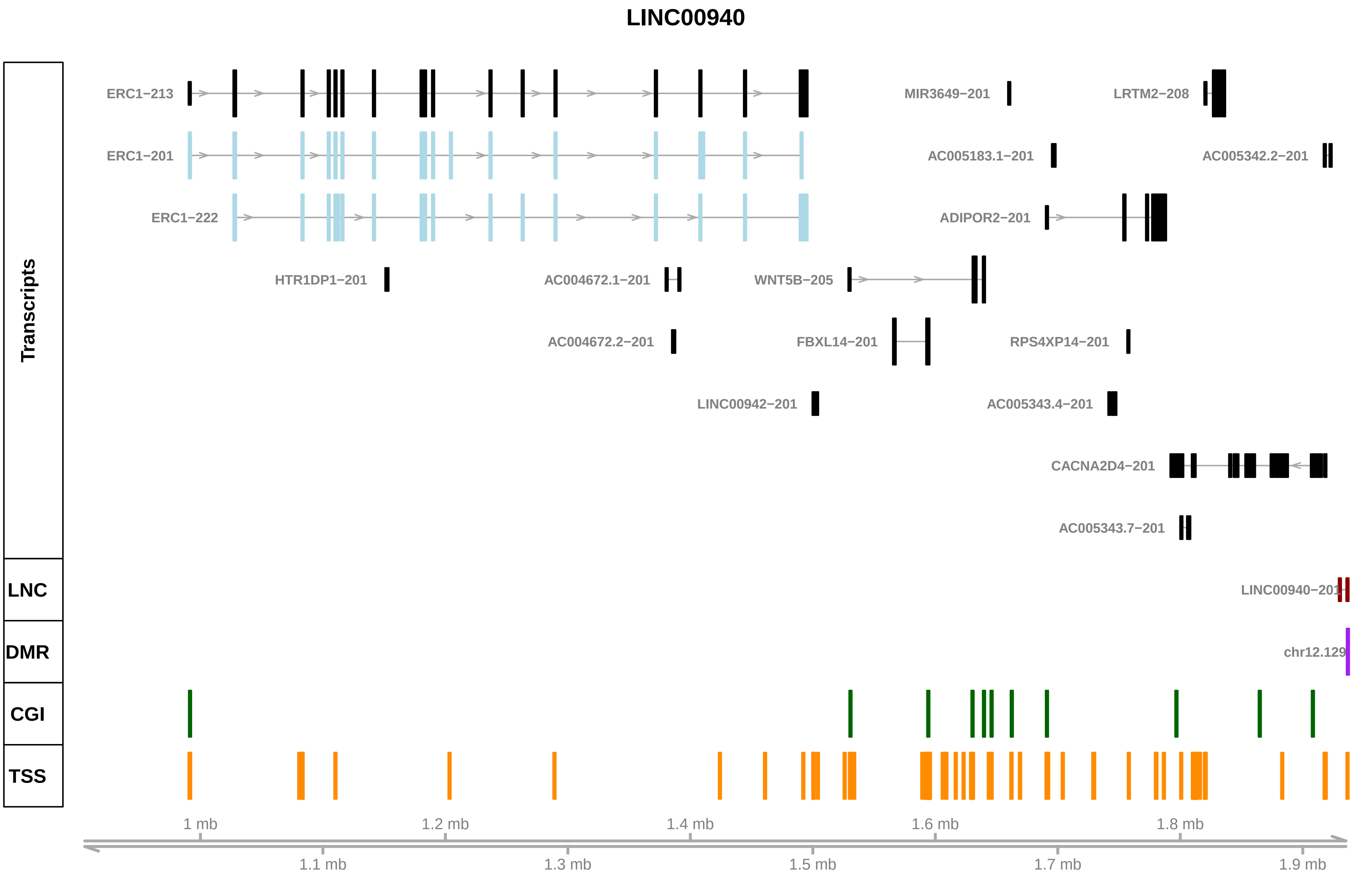
LINC00940
Transcripts
ERC1−213
ERC1−201
ERC1−222
HTR1DP1−201
AC004672.1−201
AC004672.2−201
LINC00942−201
WNT5B−205
FBXL14−201
MIR3649−201
AC005183.1−201
ADIPOR2−201
RPS4XP14−201
AC005343.4−201
CACNA2D4−201
AC005343.7−201
LRTM2−208
AC005342.2−201
LNC
LINC00940−201
DMR
chr12.129
CGI
TSS
1 mb
1.1 mb
1.2 mb
1.3 mb
1.4 mb
1.5 mb
1.6 mb
1.7 mb
1.8 mb
1.9 mb

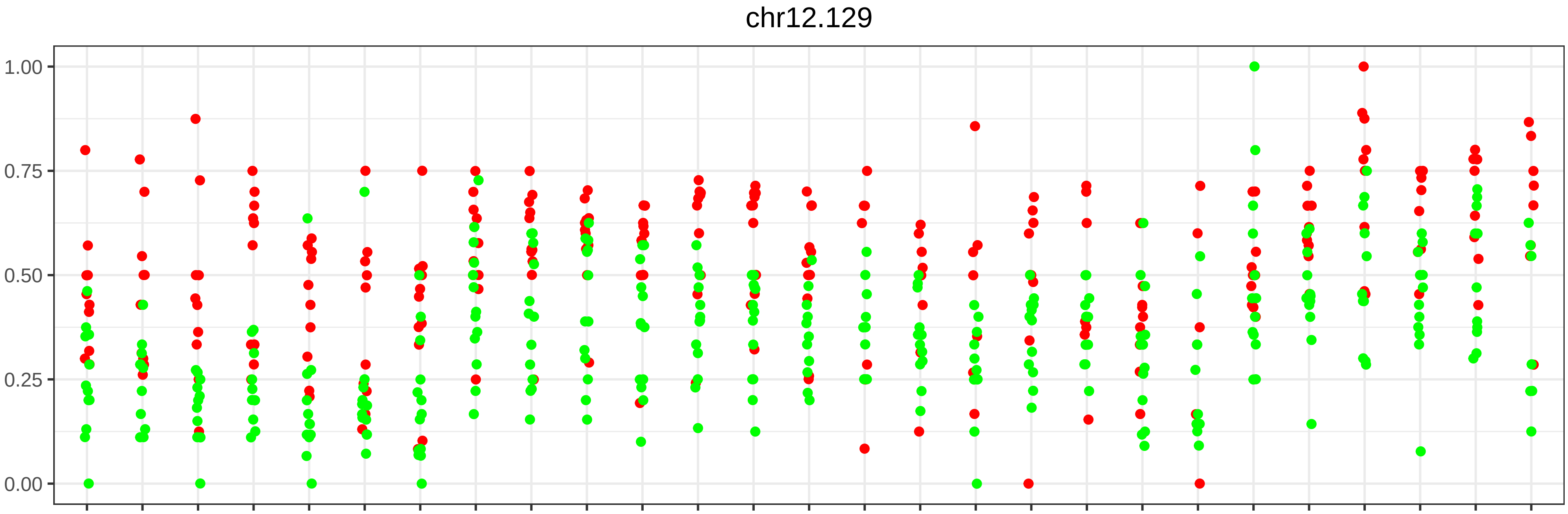
chr12.129
1.00
0.75
0.50
0.25
0.00

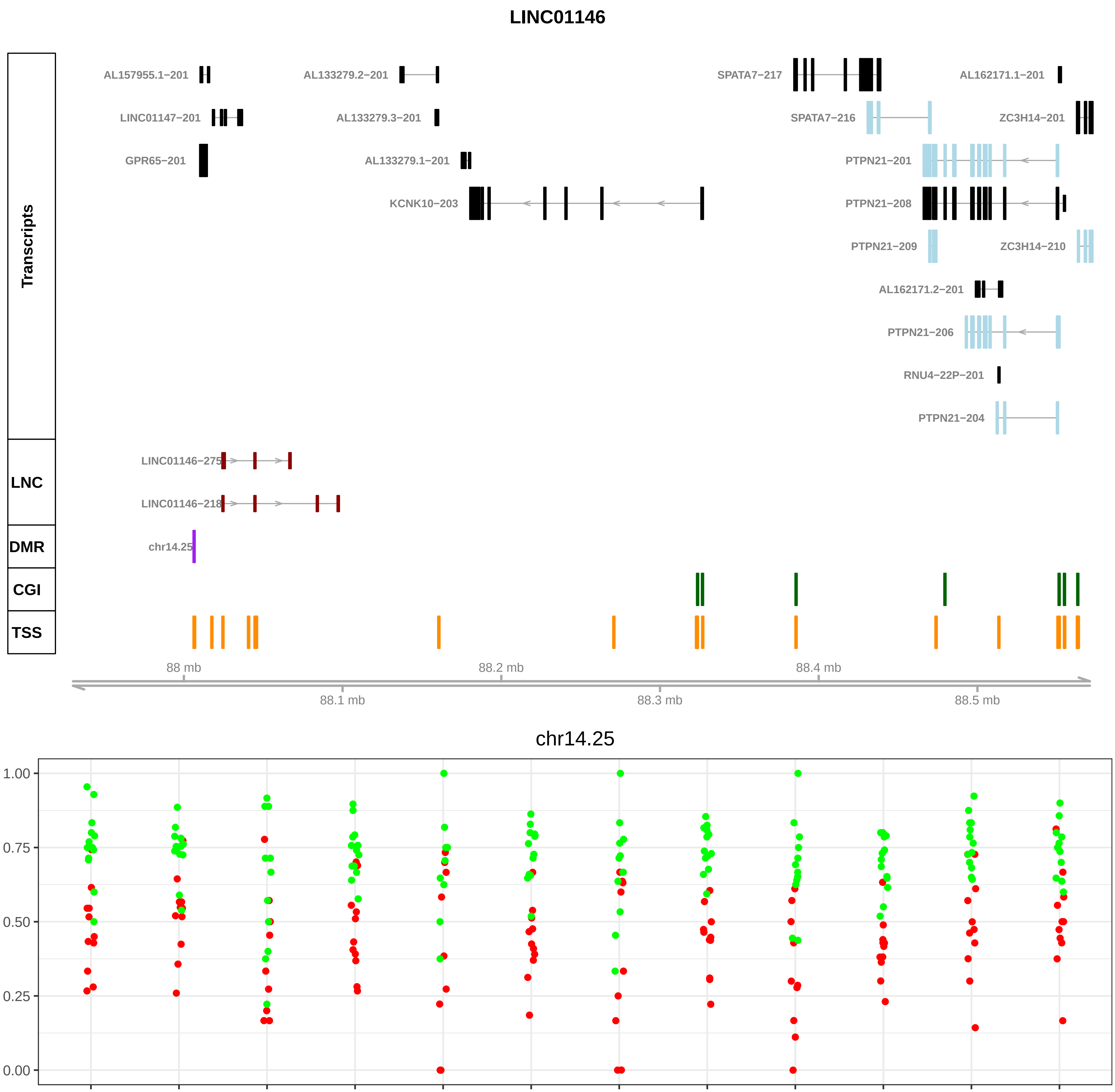

LINC01146
Transcripts
AL157955.1−201
AL133279.2−201
SPATA7−217
AL162171.1−201
LINC01147−201
AL133279.3−201
SPATA7−216
ZC3H14−201
GPR65−201
AL133279.1−201
PTPN21−201
KCNK10−203
PTPN21−208
PTPN21−209
ZC3H14−210
AL162171.2−201
PTPN21−206
RNU4−22P−201
PTPN21−204
LNC
LINC01146−275
LINC01146−218
DMR
chr14.25
CGI
TSS
88 mb
88.1 mb
88.2 mb
88.3 mb
88.4 mb
88.5 mb
chr14.25
1.00
0.75
0.50
0.25
0.00

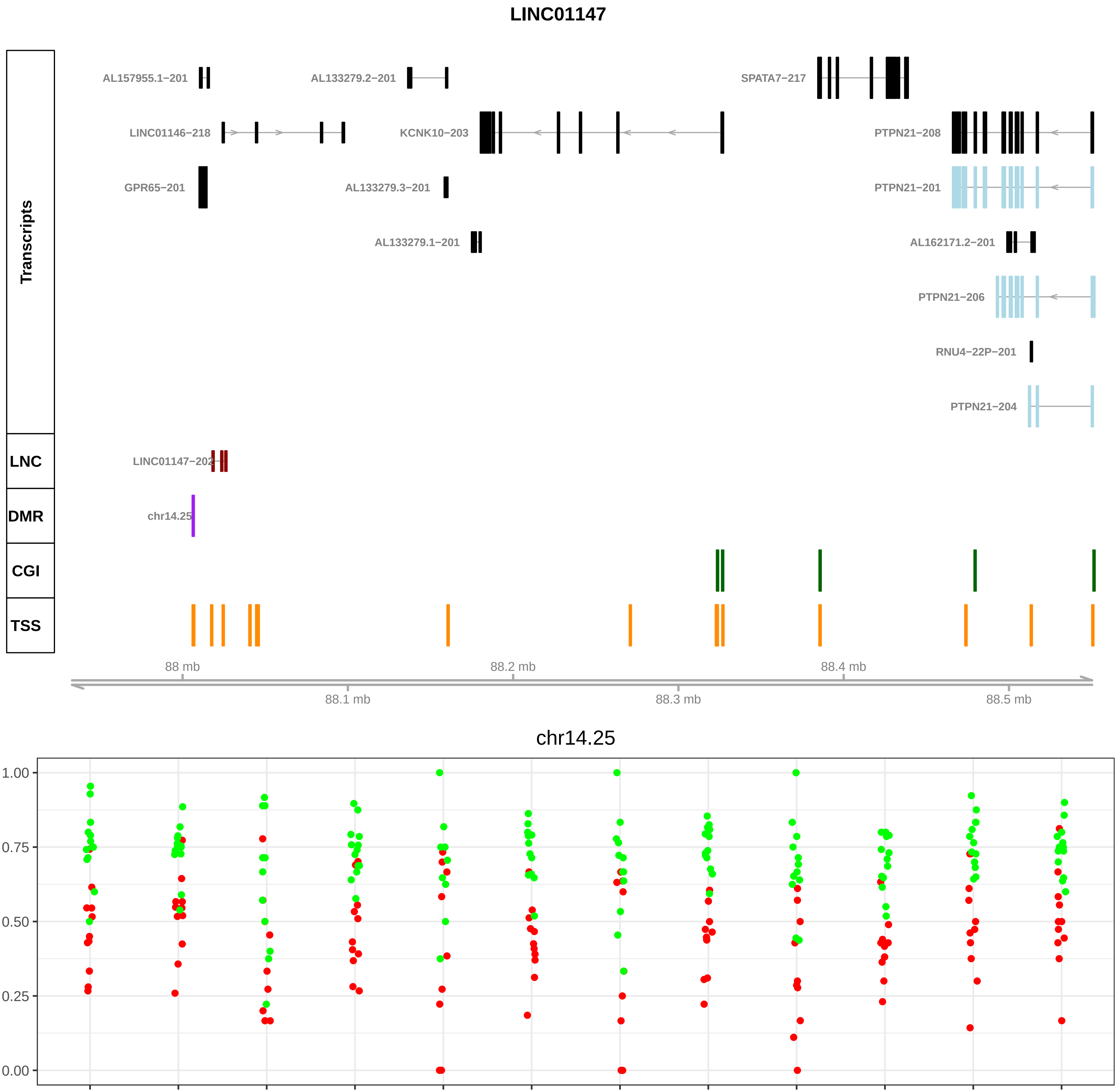

LINC01147
Transcripts
AL157955.1−201
AL133279.2−201
SPATA7−217
LINC01146−218
KCNK10−203
PTPN21−208
GPR65−201
AL133279.3−201
PTPN21−201
AL133279.1−201
AL162171.2−201
PTPN21−206
RNU4−22P−201
PTPN21−204
LNC
LINC01147−202
DMR
chr14.25
CGI
TSS
88 mb
88.1 mb
88.2 mb
88.3 mb
88.4 mb
88.5 mb
chr14.25
1.00
0.75
0.50
0.25
0.00

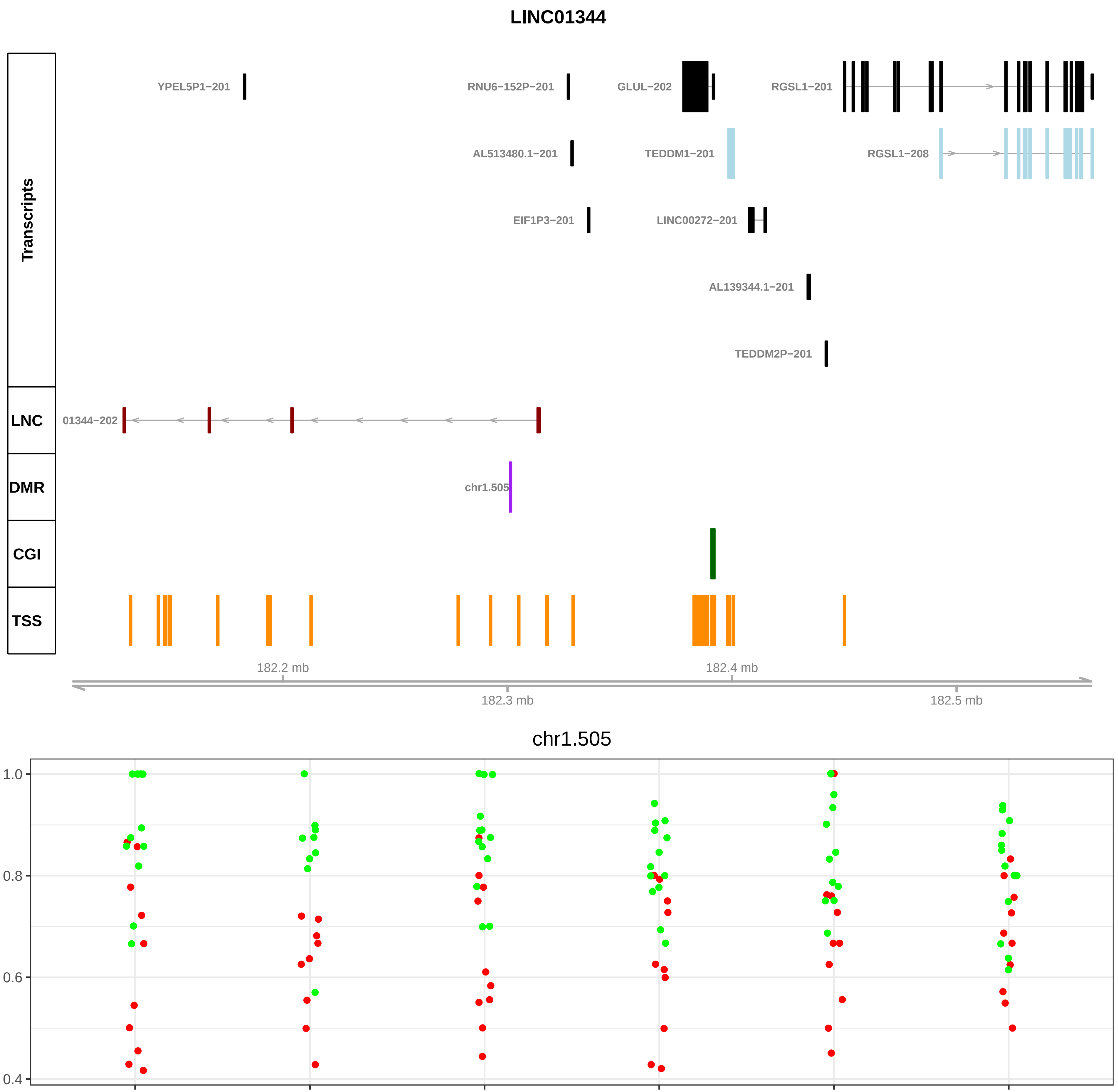

LINC01344
Transcripts
YPEL5P1−201
RNU6−152P−201
GLUL−202
RGSL1−201
AL513480.1−201
TEDDM1−201
RGSL1−208
EIF1P3−201
LINC00272−201
AL139344.1−201
TEDDM2P−201
LNC
:01344−202
DMR
chr1.505
CGI
TSS
182.2 mb
182.4 mb
182.3 mb
182.5 mb
chr1.505
1.0
0.8
0.6
0.4

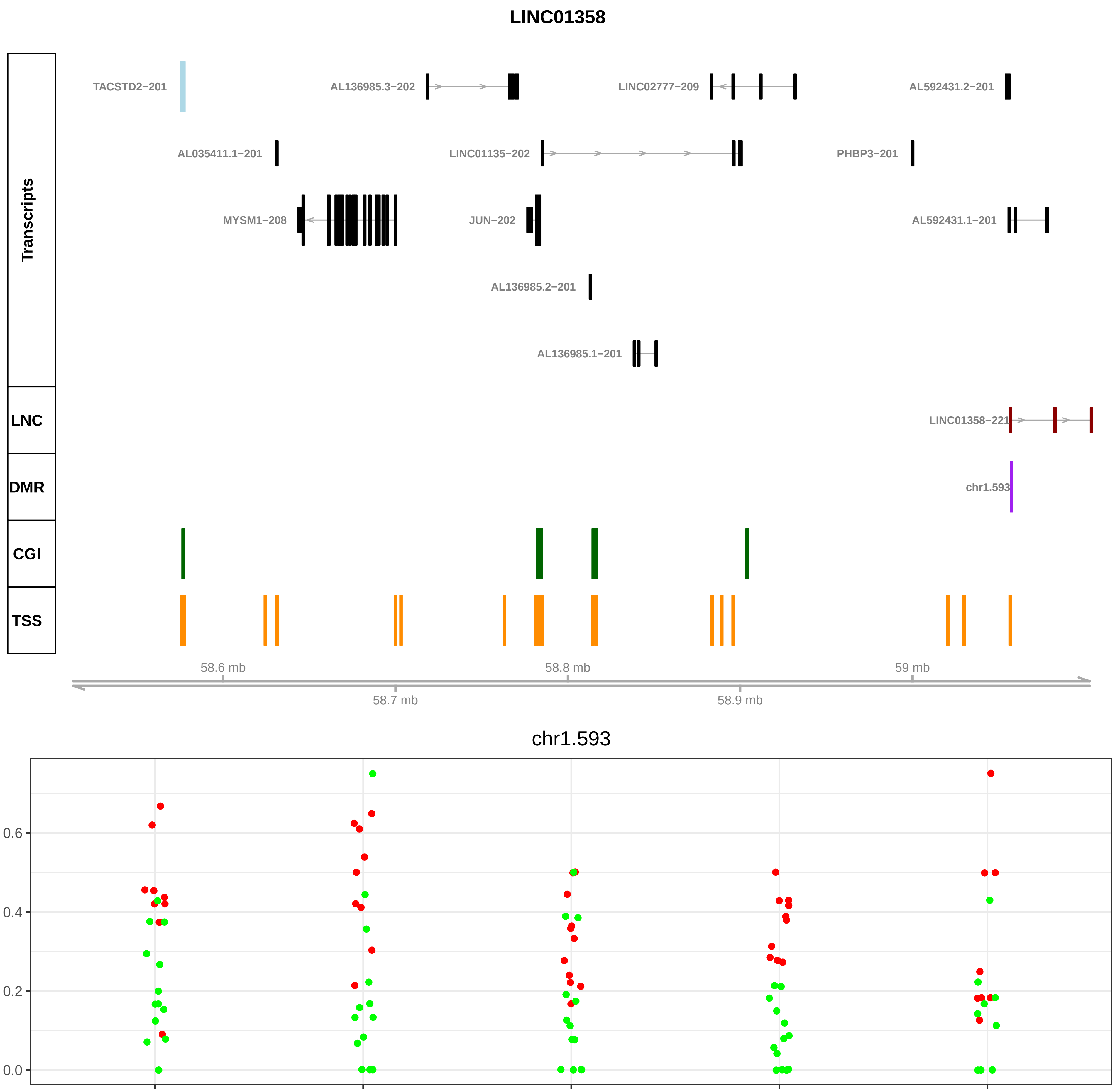
LINC01358
Transcripts
TACSTD2−201
AL136985.3−202
LINC02777−209
AL592431.2−201
AL035411.1−201
LINC01135−202
PHBP3−201
MYSM1−208
JUN−202
AL592431.1−201
AL136985.2−201
AL136985.1−201
LNC
LINC01358−221
DMR
chr1.593
CGI
TSS
58.6 mb
58.8 mb
59 mb
58.7 mb
58.9 mb
chr1.593
0.6
0.4
0.2
0.0

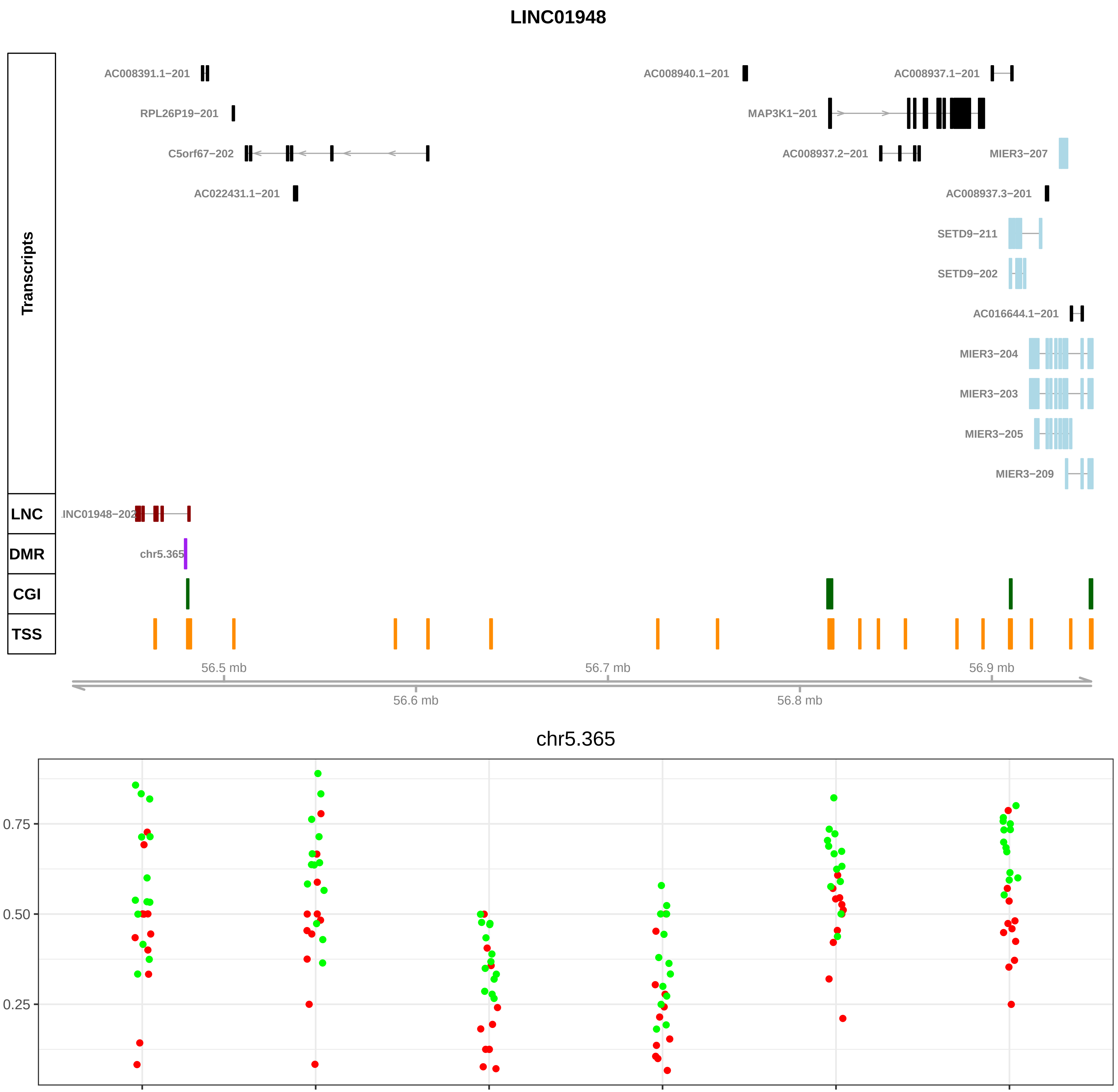

LINC01948
Transcripts
AC008391.1−201
RPL26P19−201
C5orf67−202
AC022431.1−201
AC008940.1−201
AC008937.1−201
MAP3K1−201
AC008937.2−201
MIER3−207
AC008937.3−201
SETD9−211
SETD9−202
AC016644.1−201
MIER3−204
MIER3−203
MIER3−205
MIER3−209
LNC
LINC01948−202
DMR
chr5.365
CGI
TSS
56.5 mb
56.6 mb
56.7 mb
56.8 mb
56.9 mb
chr5.365
0.75
0.50
0.25

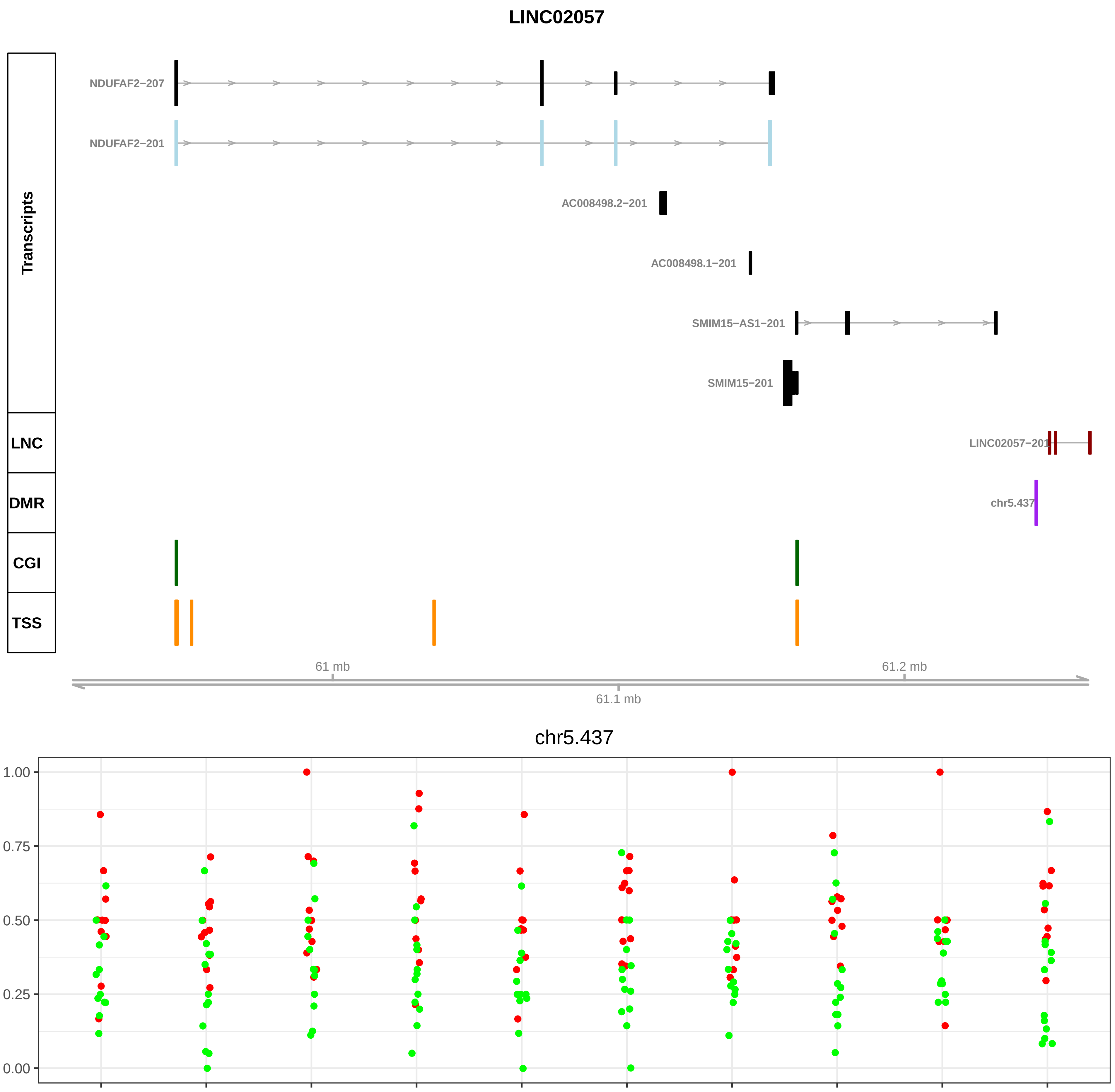

LINC02057
Transcripts
NDUFAF2−207
NDUFAF2−201
AC008498.2−201
AC008498.1−201
SMIM15−AS1−201
SMIM15−201
LNC
LINC02057−201
DMR
chr5.437
CGI
TSS
61 mb
61.1 mb
61.2 mb
chr5.437
1.00
0.75
0.50
0.25
0.00

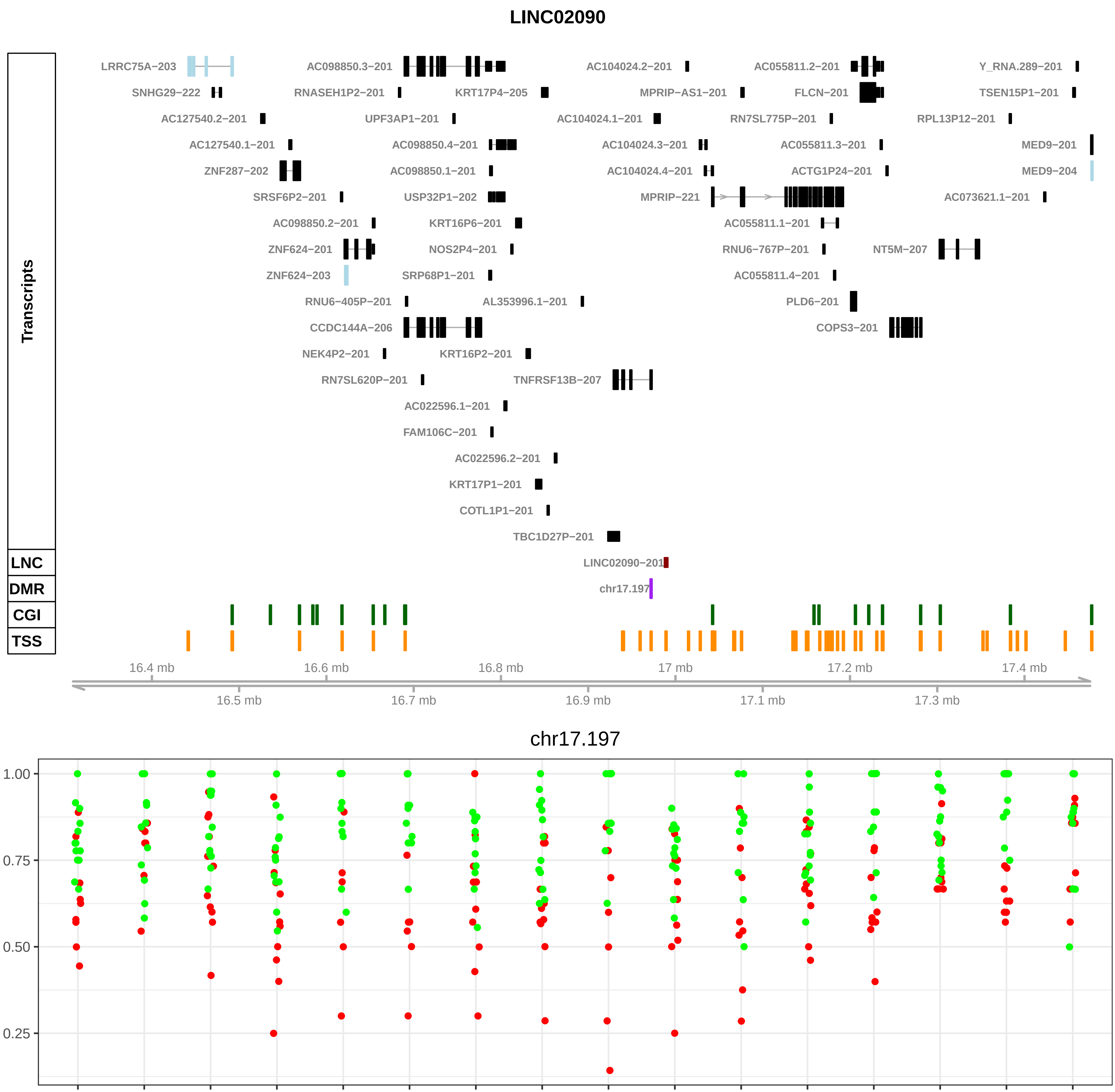

LINC02090
Transcripts
LRRC75A−203
AC098850.3−201
AC104024.2−201
AC055811.2−201
Y_RNA.289−201
SNHG29−222
RNASEH1P2−201
KRT17P4−205
MPRIP−AS1−201
FLCN−201
TSEN15P1−201
AC127540.2−201
UPF3AP1−201
AC104024.1−201
RN7SL775P−201
RPL13P12−201
AC127540.1−201
AC098850.4−201
AC104024.3−201
AC055811.3−201
MED9−201
ZNF287−202
AC098850.1−201
AC104024.4−201
ACTG1P24−201
MED9−204
SRSF6P2−201
USP32P1−202
MPRIP−221
AC073621.1−201
AC098850.2−201
KRT16P6−201
AC055811.1−201
ZNF624−201
NOS2P4−201
RNU6−767P−201
NT5M−207
ZNF624−203
SRP68P1−201
AC055811.4−201
RNU6−405P−201
AL353996.1−201
PLD6−201
CCDC144A−206
COPS3−201
NEK4P2−201
KRT16P2−201
RN7SL620P−201
TNFRSF13B−207
AC022596.1−201
FAM106C−201
AC022596.2−201
KRT17P1−201
COTL1P1−201
TBC1D27P−201
LNC
LINC02090−201
DMR
chr17.197
CGI
TSS
16.4 mb
16.6 mb
16.8 mb
17 mb
17.2 mb
17.4 mb
16.5 mb
16.7 mb
16.9 mb
17.1 mb
17.3 mb
chr17.197
1.00
0.75
0.50
0.25

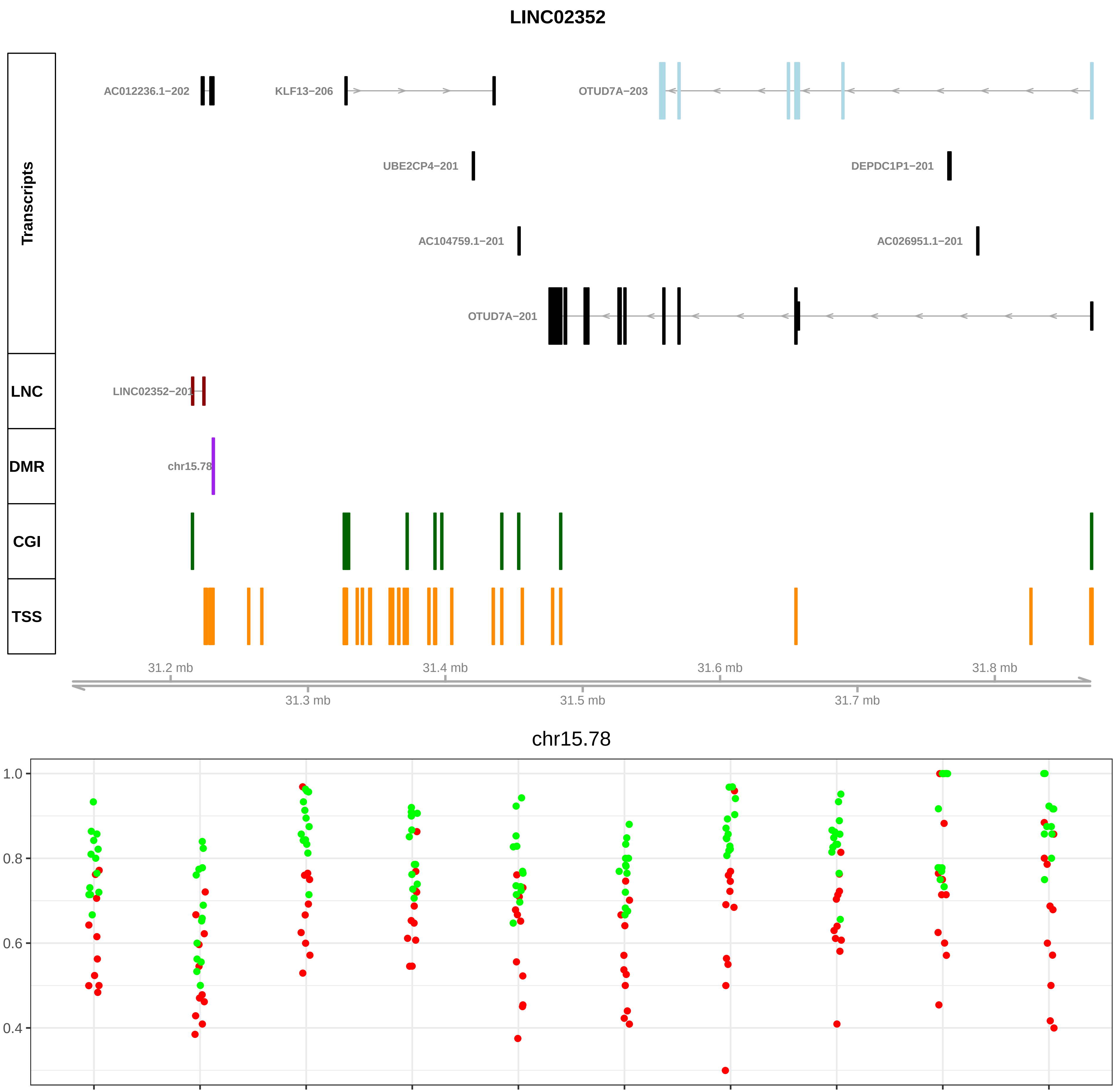

LINC02352
Transcripts
AC012236.1−202
KLF13−206
OTUD7A−203
UBE2CP4−201
DEPDC1P1−201
AC104759.1−201
AC026951.1−201
OTUD7A−201
LNC
LINC02352−201
DMR
chr15.78
CGI
TSS
31.2 mb
31.4 mb
31.6 mb
31.8 mb
31.3 mb
31.5 mb
31.7 mb
chr15.78
1.0
0.8
0.6
0.4

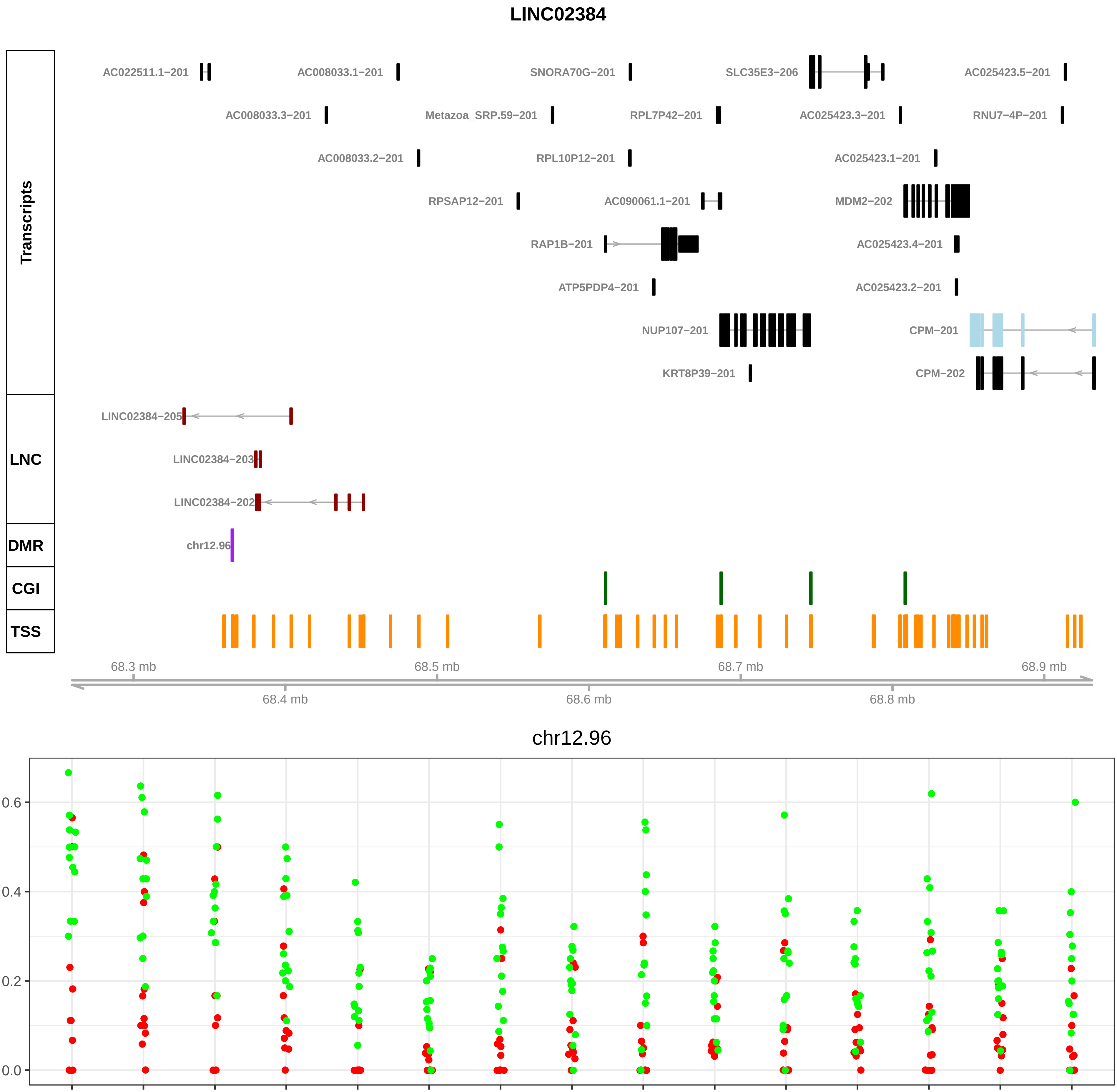
LINC02384
Transcripts
AC022511.1−201
AC008033.1−201
SNORA70G−201
SLC35E3−206
AC025423.5−201
AC008033.3−201
Metazoa_SRP.59−201
RPL7P42−201
AC025423.3−201
RNU7−4P−201
AC008033.2−201
RPL10P12−201
AC025423.1−201
RPSAP12−201
AC090061.1−201
MDM2−202
RAP1B−201
AC025423.4−201
ATP5PDP4−201
AC025423.2−201
NUP107−201
CPM−201
KRT8P39−201
CPM−202
LNC
LINC02384−205
LINC02384−203
LINC02384−202
DMR
chr12.96
CGI
TSS
68.3 mb
68.5 mb
68.7 mb
68.9 mb
68.4 mb
68.6 mb
68.8 mb
chr12.96
0.6
0.4
0.2
0.0

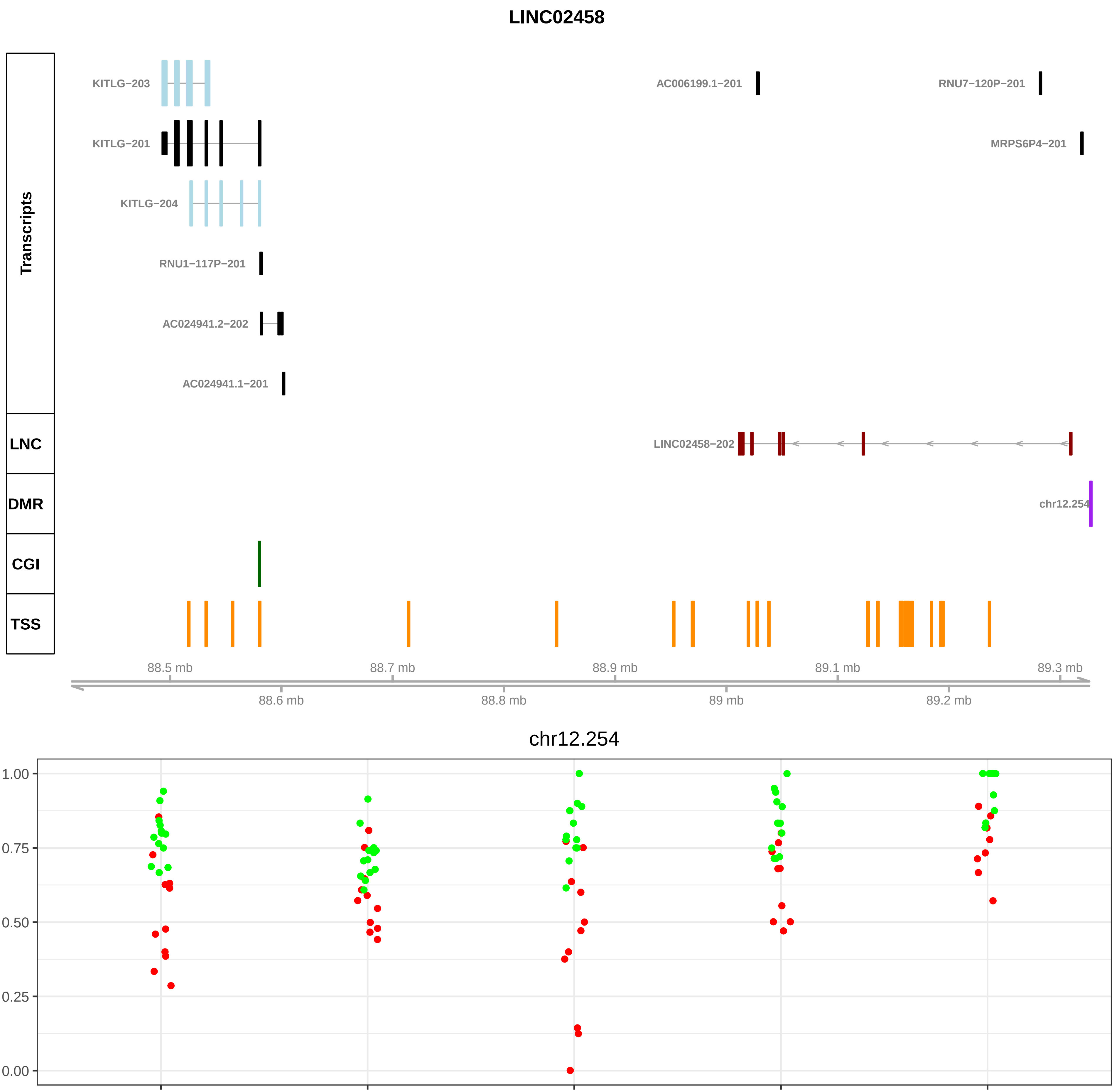

LINC02458
Transcripts
KITLG−203
KITLG−201
KITLG−204
RNU1−117P−201
AC024941.2−202
AC024941.1−201
AC006199.1−201
RNU7−120P−201
MRPS6P4−201
LNC
LINC02458−202
DMR
chr12.254
CGI
TSS
88.5 mb
88.6 mb
88.7 mb
88.8 mb
88.9 mb
89 mb
89.1 mb
89.2 mb
89.3 mb
chr12.254
1.00
0.75
0.50
0.25
0.00

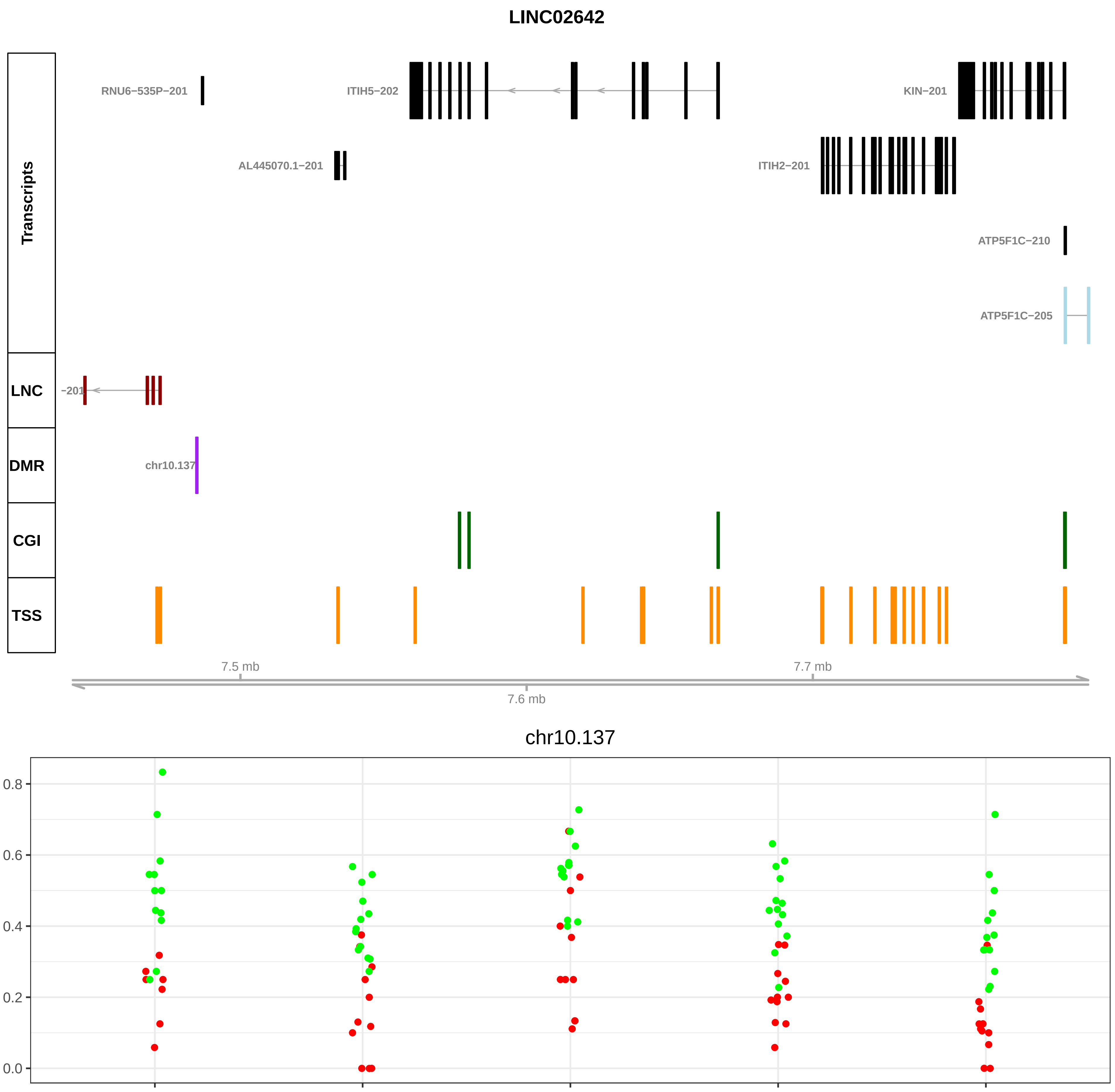

LINC02642
Transcripts
RNU6−535P−201
ITIH5−202
KIN−201
AL445070.1−201
ITIH2−201
ATP5F1C−210
ATP5F1C−205
LNC
−201
DMR
chr10.137
CGI
TSS
7.5 mb
7.6 mb
7.7 mb
chr10.137
0.0
0.2
0.4
0.6
0.8

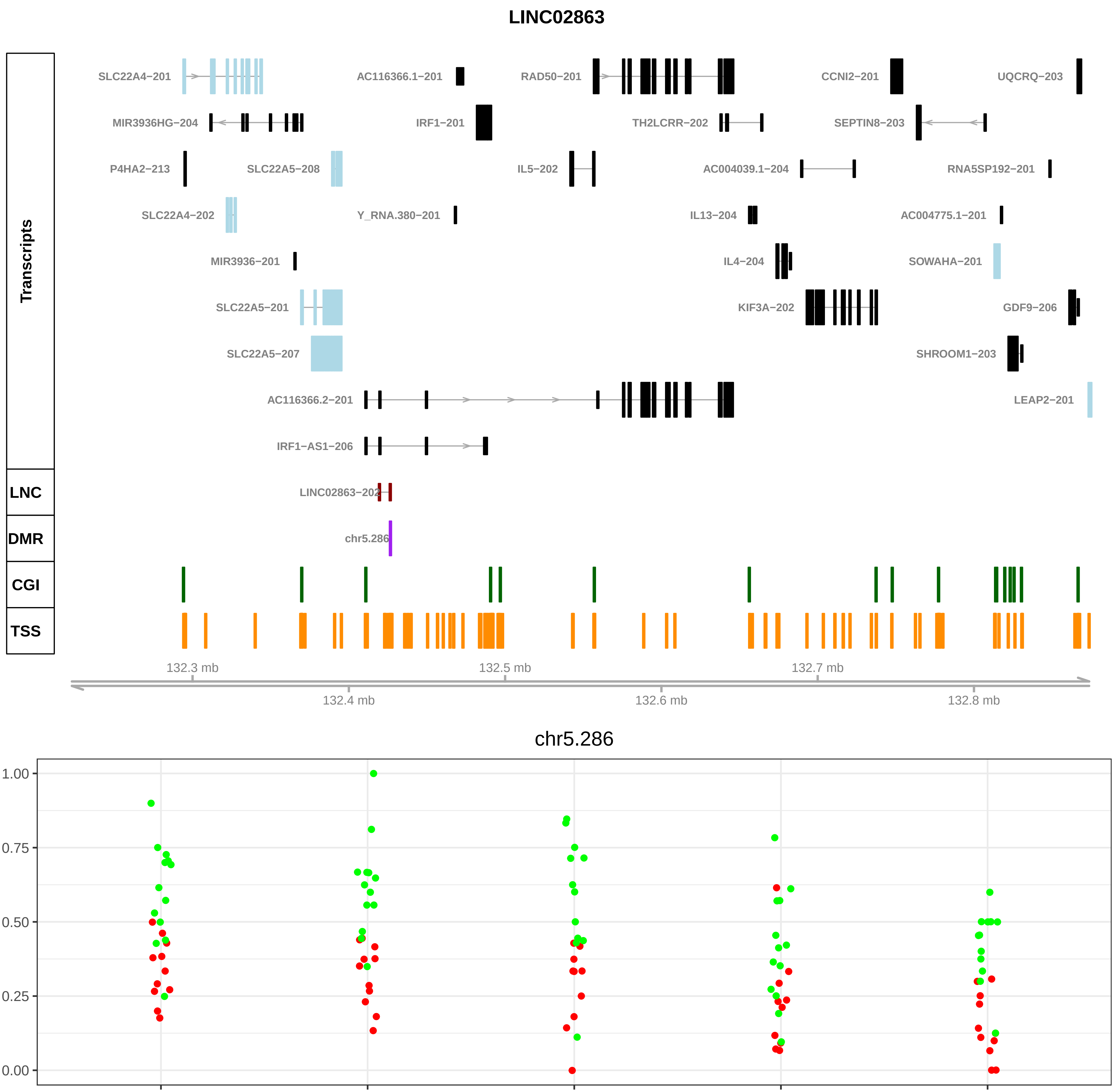
LINC02863
Transcripts
SLC22A4−201
AC116366.1−201
RAD50−201
CCNI2−201
UQCRQ−203
MIR3936HG−204
IRF1−201
TH2LCRR−202
SEPTIN8−203
P4HA2−213
SLC22A5−208
IL5−202
AC004039.1−204
RNA5SP192−201
SLC22A4−202
Y_RNA.380−201
IL13−204
AC004775.1−201
MIR3936−201
IL4−204
SOWAHA−201
SLC22A5−201
KIF3A−202
GDF9−206
SLC22A5−207
SHROOM1−203
AC116366.2−201
LEAP2−201
IRF1−AS1−206
LNC
LINC02863−202
DMR
chr5.286
CGI
TSS
132.3 mb
132.5 mb
132.7 mb
132.4 mb
132.6 mb
132.8 mb
chr5.286
1.00
0.75
0.50
0.25
0.00

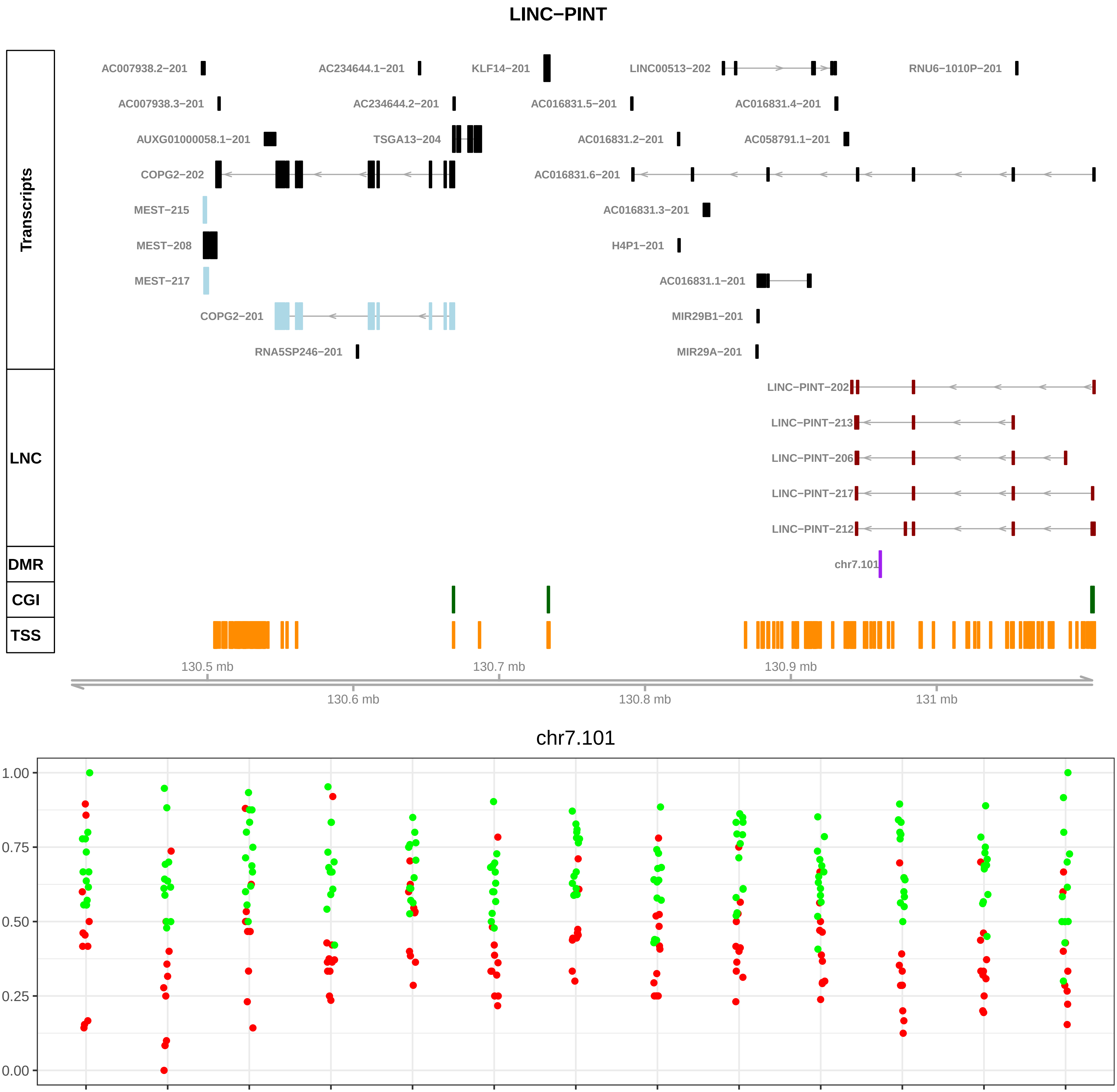
LINC−PINT
Transcripts
LNC
DMR
CGI
TSS
AC007938.2−201
AC234644.1−201
KLF14−201
LINC00513−202
RNU6−1010P−201
AC007938.3−201
AC234644.2−201
AC016831.5−201
AC016831.4−201
AUXG01000058.1−201
TSGA13−204
AC016831.2−201
AC058791.1−201
COPG2−202
AC016831.6−201
MEST−215
AC016831.3−201
MEST−208
H4P1−201
MEST−217
AC016831.1−201
COPG2−201
MIR29B1−201
RNA5SP246−201
MIR29A−201
LINC−PINT−202
LINC−PINT−213
LINC−PINT−206
LINC−PINT−217
LINC−PINT−212
chr7.101
130.5 mb
130.7 mb
130.9 mb
130.6 mb
130.8 mb
131 mb
chr7.101
1.00
0.75
0.50
0.25
0.00

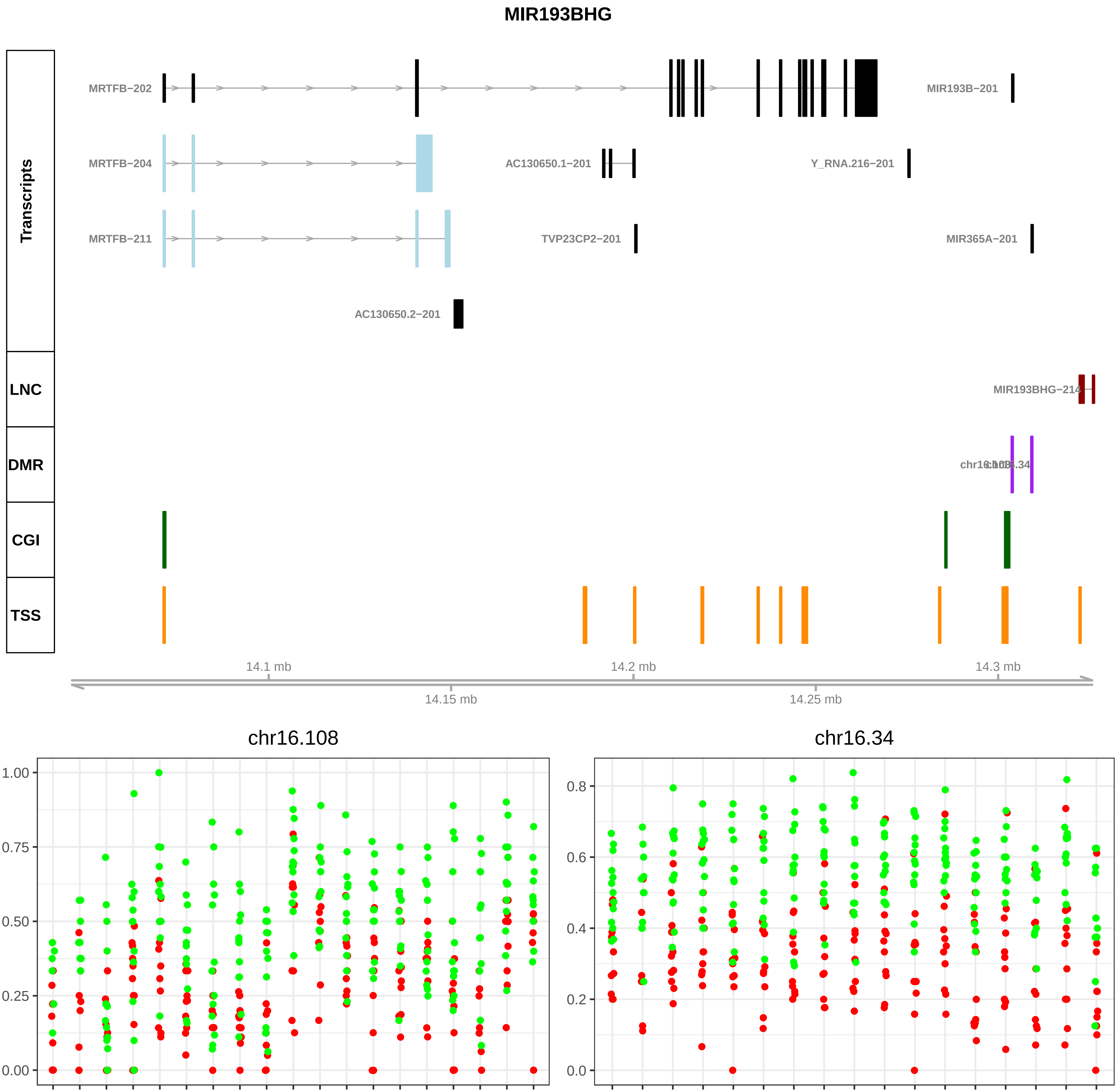
MIR193BHG
Transcripts
LNC
DMR
CGI
TSS
MRTFB−202
MIR193B−201
MRTFB−204
AC130650.1−201
Y_RNA.216−201
MRTFB−211
TVP23CP2−201
MIR365A−201
AC130650.2−201
MIR193BHG−214
chr16.108
chr16.34
14.1 mb
14.2 mb
14.3 mb
14.15 mb
14.25 mb
chr16.108
1.00
0.75
0.50
0.25
0.00
chr16.34
0.8
0.6
0.4
0.2
0.0

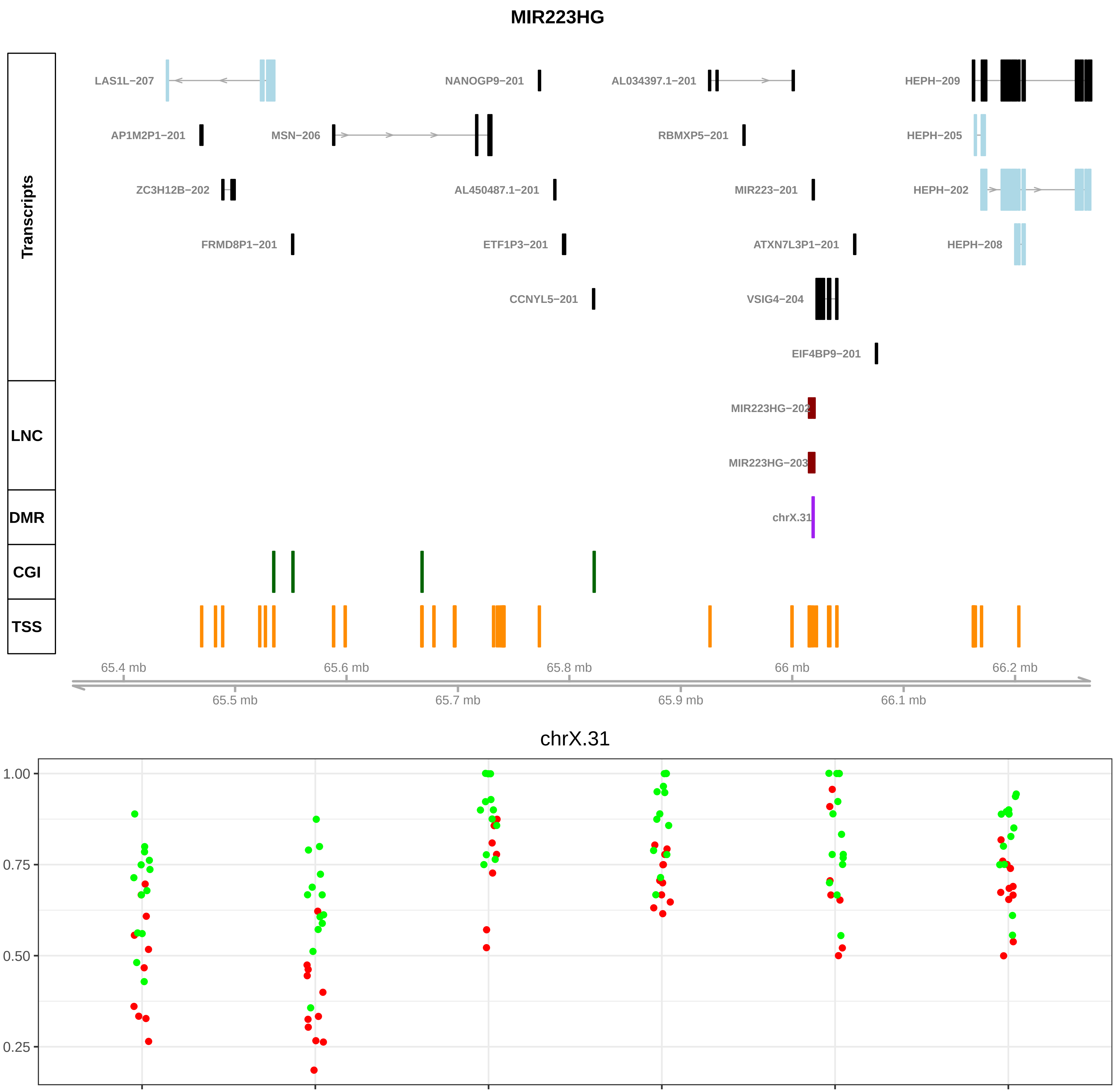

MIR223HG
Transcripts
LAS1L−207
NANOGP9−201
AL034397.1−201
HEPH−209
AP1M2P1−201
MSN−206
RBMXP5−201
HEPH−205
ZC3H12B−202
AL450487.1−201
MIR223−201
HEPH−202
FRMD8P1−201
ETF1P3−201
ATXN7L3P1−201
HEPH−208
CCNYL5−201
VSIG4−204
EIF4BP9−201
LNC
MIR223HG−202
MIR223HG−203
DMR
chrX.31
CGI
TSS
65.4 mb
65.6 mb
65.8 mb
66 mb
66.2 mb
65.5 mb
65.7 mb
65.9 mb
66.1 mb
chrX.31
1.00
0.75
0.50
0.25

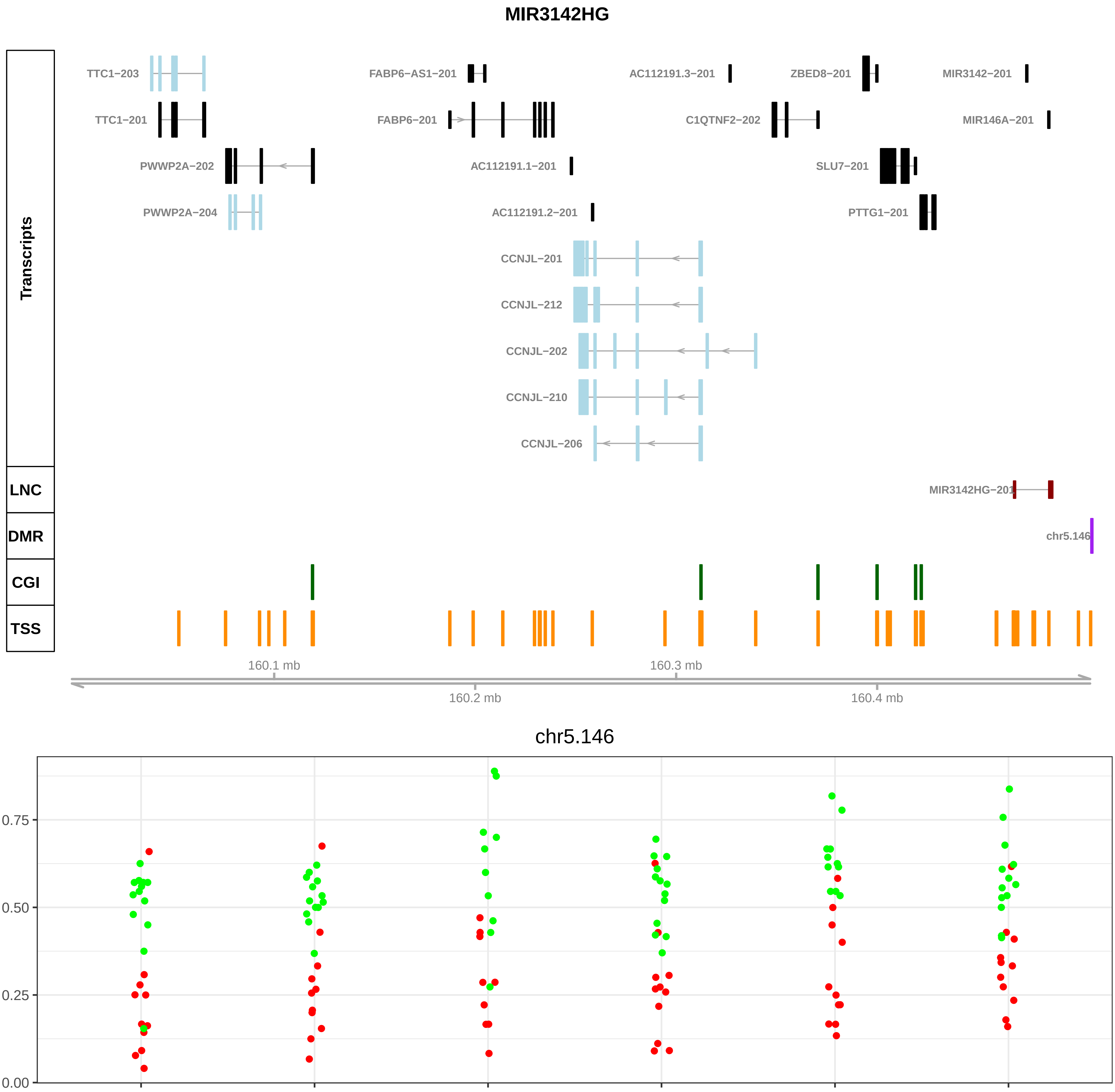

MIR3142HG
Transcripts
TTC1−203
TTC1−201
PWWP2A−202
PWWP2A−204
FABP6−AS1−201
FABP6−201
AC112191.1−201
AC112191.2−201
CCNJL−201
CCNJL−212
CCNJL−202
CCNJL−210
CCNJL−206
AC112191.3−201
C1QTNF2−202
ZBED8−201
SLU7−201
PTTG1−201
MIR3142−201
MIR146A−201
LNC
MIR3142HG−201
DMR
chr5.146
CGI
TSS
160.1 mb
160.2 mb
160.3 mb
160.4 mb
chr5.146
0.00
0.25
0.50
0.75

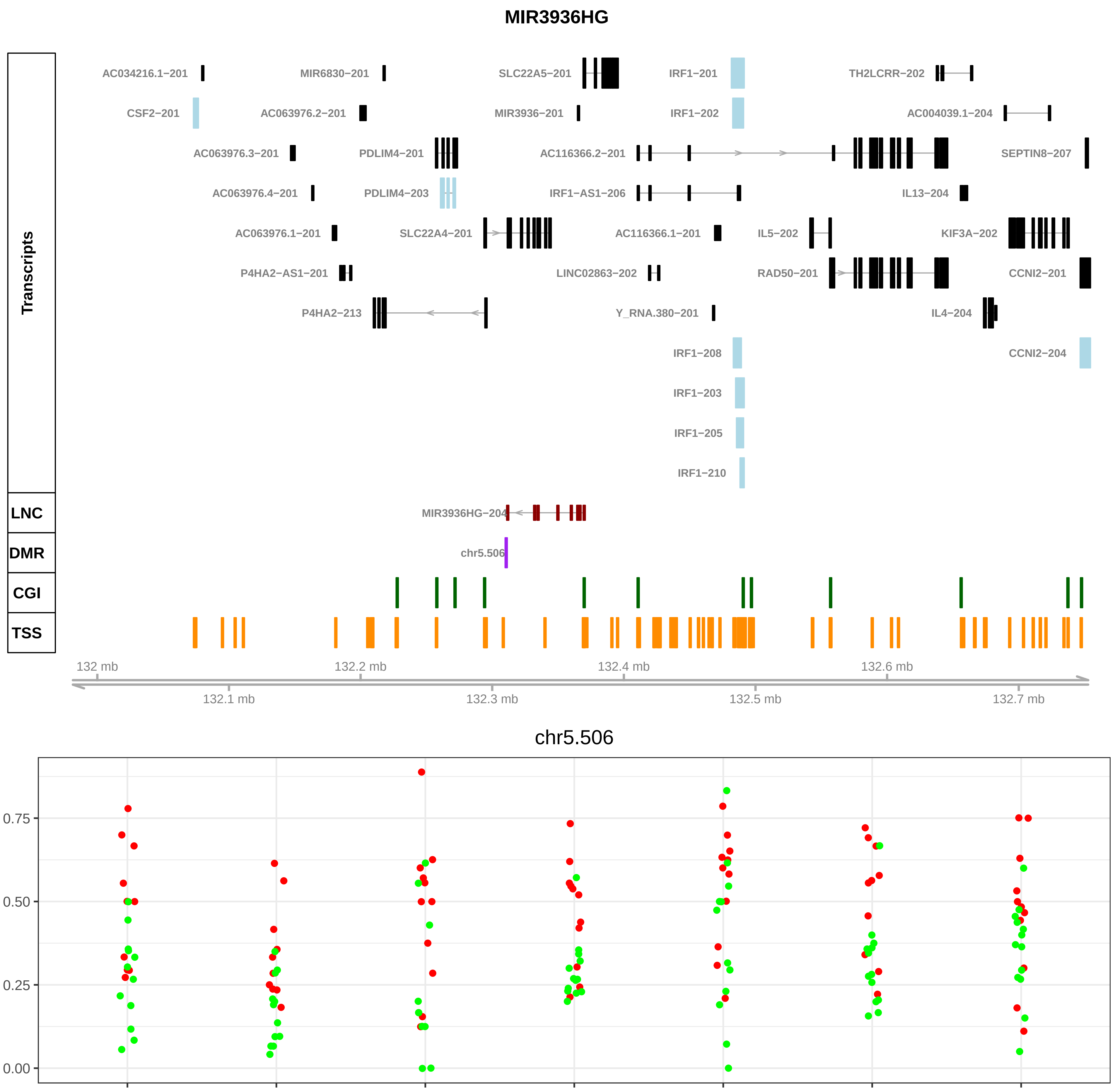

MIR3936HG
Transcripts
AC034216.1−201
MIR6830−201
SLC22A5−201
IRF1−201
TH2LCRR−202
CSF2−201
AC063976.2−201
MIR3936−201
IRF1−202
AC004039.1−204
AC063976.3−201
PDLIM4−201
AC116366.2−201
SEPTIN8−207
AC063976.4−201
PDLIM4−203
IRF1−AS1−206
IL13−204
AC063976.1−201
SLC22A4−201
AC116366.1−201
IL5−202
KIF3A−202
P4HA2−AS1−201
LINC02863−202
RAD50−201
CCNI2−201
P4HA2−213
Y_RNA.380−201
IL4−204
IRF1−208
CCNI2−204
IRF1−203
IRF1−205
IRF1−210
LNC
MIR3936HG−204
DMR
chr5.506
CGI
TSS
132 mb
132.1 mb
132.2 mb
132.3 mb
132.4 mb
132.5 mb
132.6 mb
132.7 mb
chr5.506
0.75
0.50
0.25
0.00

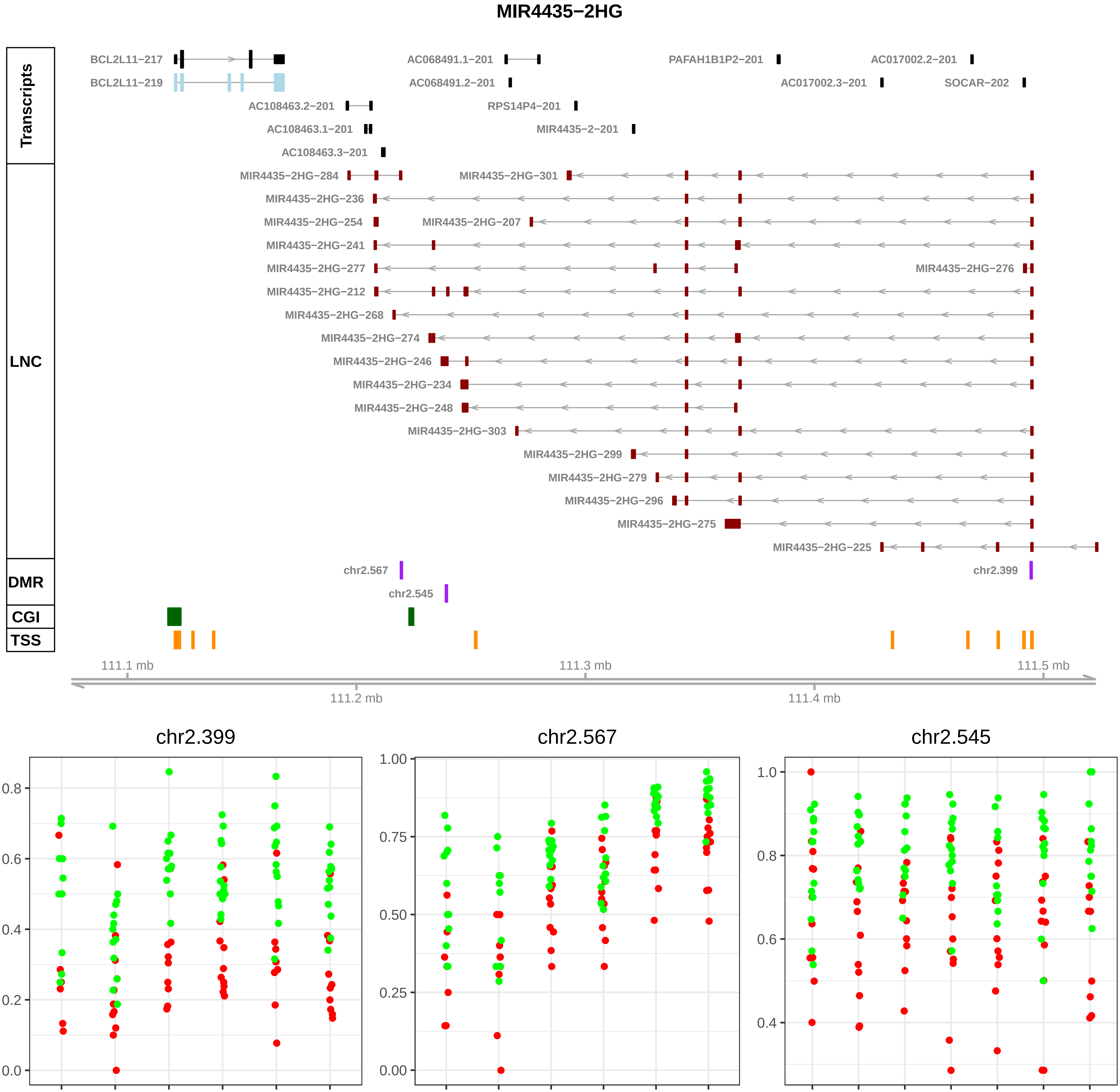
MIR4435−2HG
Transcripts
LNC
DMR
CGI
TSS
BCL2L11−217
BCL2L11−219
AC068491.1−201
AC068491.2−201
PAFAH1B1P2−201
AC017002.2−201
AC017002.3−201
SOCAR−202
AC108463.2−201
RPS14P4−201
AC108463.1−201
MIR4435−2−201
AC108463.3−201
MIR4435−2HG−284
MIR4435−2HG−301
MIR4435−2HG−236
MIR4435−2HG−254
MIR4435−2HG−207
MIR4435−2HG−241
MIR4435−2HG−277
MIR4435−2HG−276
MIR4435−2HG−212
MIR4435−2HG−268
MIR4435−2HG−274
MIR4435−2HG−246
MIR4435−2HG−234
MIR4435−2HG−248
MIR4435−2HG−303
MIR4435−2HG−299
MIR4435−2HG−279
MIR4435−2HG−296
MIR4435−2HG−275
MIR4435−2HG−225
chr2.567
chr2.399
chr2.545
111.1 mb
111.3 mb
111.5 mb
111.2 mb
111.4 mb
chr2.399
chr2.567
chr2.545

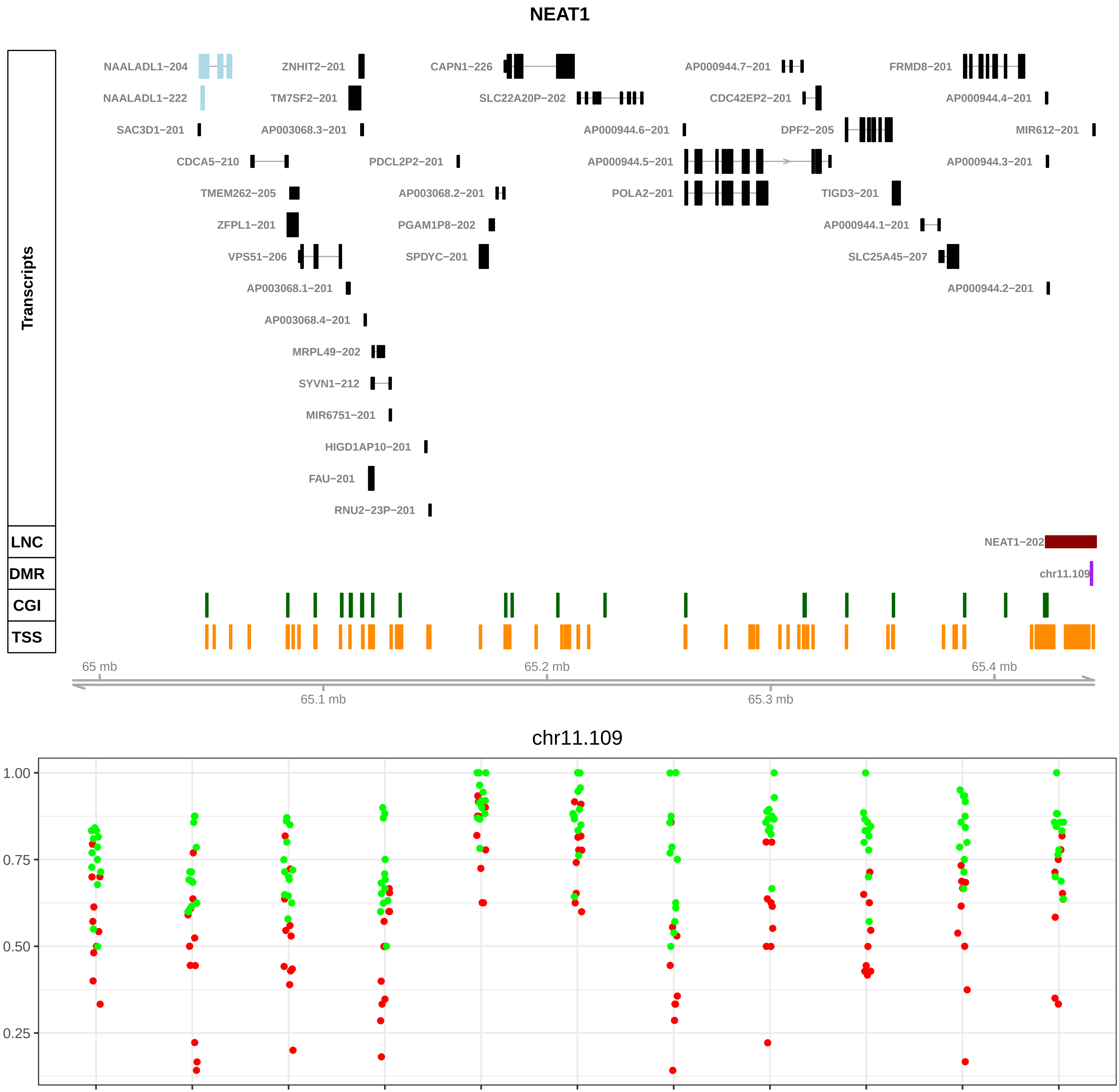
NEAT1
Transcripts
NAALADL1−204
NAALADL1−222
SAC3D1−201
CDCA5−210
TMEM262−205
ZFPL1−201
VPS51−206
AP003068.1−201
AP003068.4−201
MRPL49−202
SYVN1−212
MIR6751−201
HIGD1AP10−201
FAU−201
RNU2−23P−201
ZNHIT2−201
TM7SF2−201
AP003068.3−201
PDCL2P2−201
AP003068.2−201
PGAM1P8−202
SPDYC−201
CAPN1−226
SLC22A20P−202
AP000944.6−201
AP000944.5−201
POLA2−201
AP000944.7−201
CDC42EP2−201
DPF2−205
TIGD3−201
AP000944.1−201
SLC25A45−207
FRMD8−201
AP000944.4−201
MIR612−201
AP000944.3−201
AP000944.2−201
LNC
NEAT1−202
DMR
chr11.109
CGI
TSS
65 mb
65.1 mb
65.2 mb
65.3 mb
65.4 mb
chr11.109
1.00
0.75
0.50
0.25

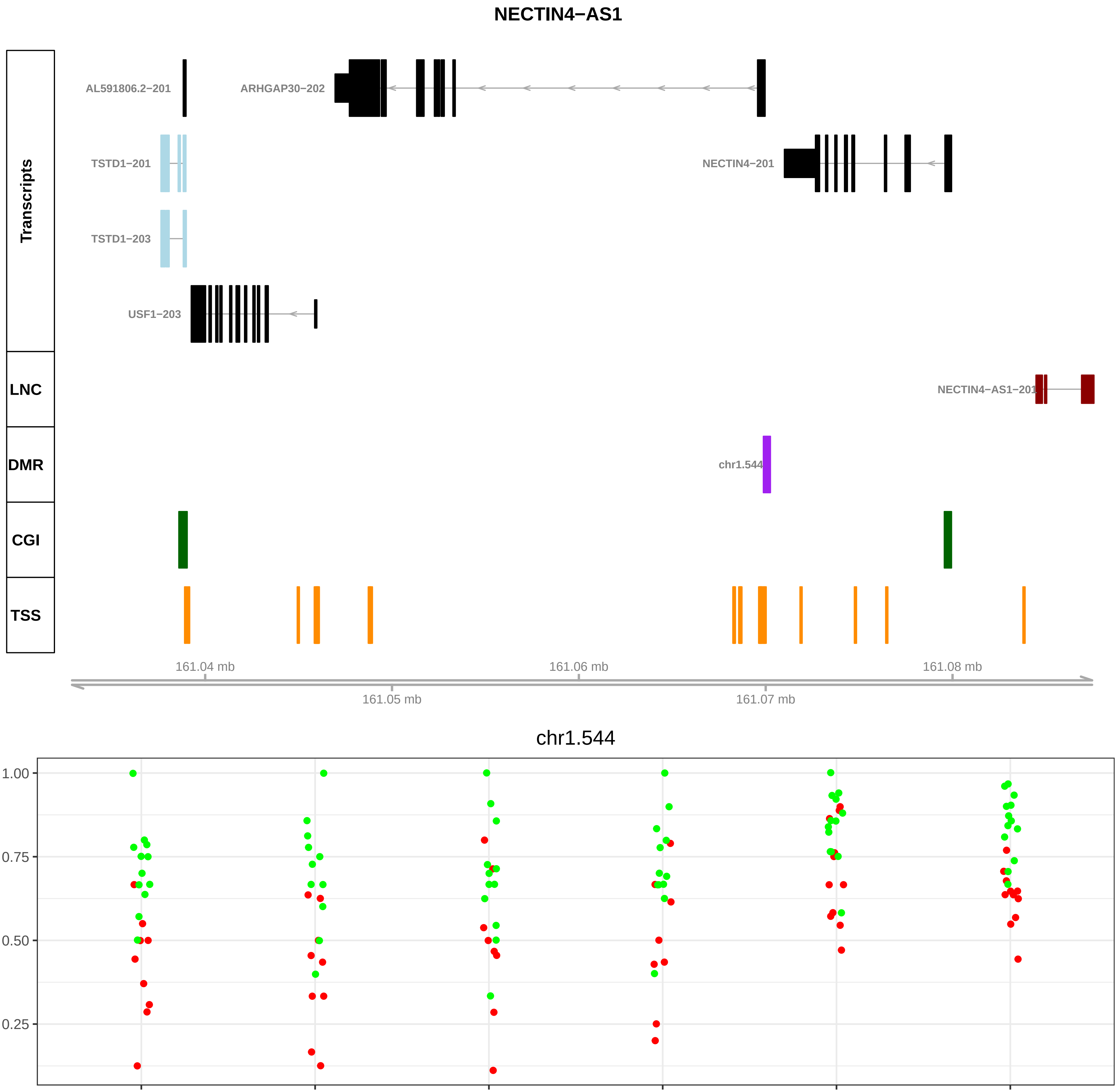

NECTIN4−AS1
Transcripts
AL591806.2−201
ARHGAP30−202
TSTD1−201
NECTIN4−201
TSTD1−203
USF1−203
LNC
NECTIN4−AS1−201
DMR
chr1.544
CGI
TSS
161.04 mb
161.06 mb
161.08 mb
161.05 mb
161.07 mb
chr1.544
1.00
0.75
0.50
0.25

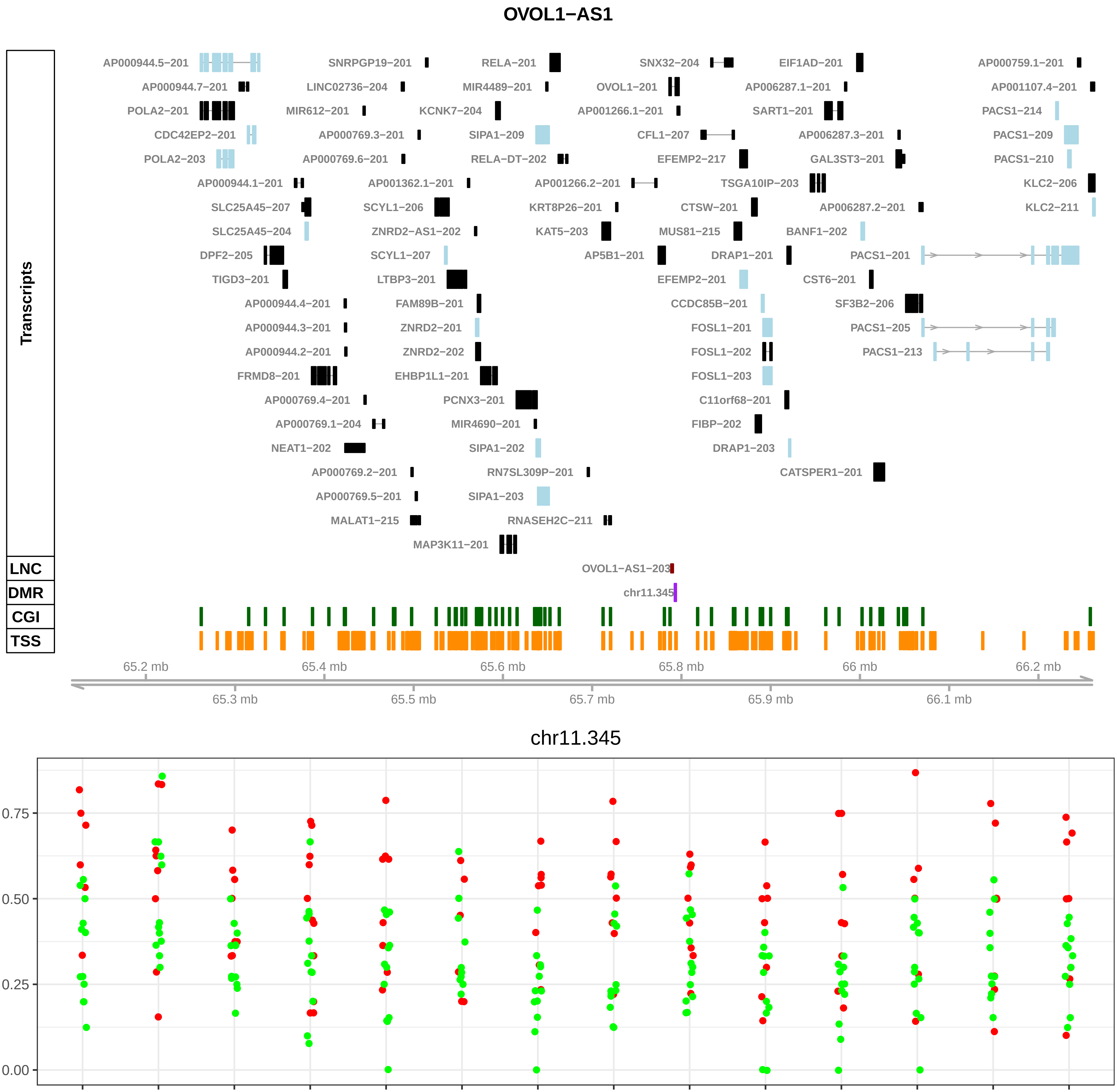
OVOL1−AS1
Transcripts
LNC
DMR
CGI
TSS
AP000944.5−201
SNRPGP19−201
RELA−201
SNX32−204
EIF1AD−201
AP000759.1−201
AP000944.7−201
LINC02736−204
MIR4489−201
OVOL1−201
AP006287.1−201
AP001107.4−201
POLA2−201
MIR612−201
KCNK7−204
AP001266.1−201
SART1−201
PACS1−214
CDC42EP2−201
AP000769.3−201
SIPA1−209
CFL1−207
AP006287.3−201
PACS1−209
POLA2−203
AP000769.6−201
RELA−DT−202
EFEMP2−217
GAL3ST3−201
PACS1−210
AP000944.1−201
AP001362.1−201
AP001266.2−201
TSGA10IP−203
KLC2−206
SLC25A45−207
SCYL1−206
KRT8P26−201
CTSW−201
AP006287.2−201
KLC2−211
SLC25A45−204
ZNRD2−AS1−202
KAT5−203
MUS81−215
BANF1−202
DPF2−205
SCYL1−207
AP5B1−201
DRAP1−201
PACS1−201
TIGD3−201
LTBP3−201
EFEMP2−201
CST6−201
AP000944.4−201
FAM89B−201
CCDC85B−201
SF3B2−206
AP000944.3−201
ZNRD2−201
FOSL1−201
PACS1−205
AP000944.2−201
ZNRD2−202
FOSL1−202
PACS1−213
FRMD8−201
EHBP1L1−201
FOSL1−203
AP000769.4−201
PCNX3−201
C11orf68−201
AP000769.1−204
MIR4690−201
FIBP−202
NEAT1−202
SIPA1−202
DRAP1−203
AP000769.2−201
RN7SL309P−201
CATSPER1−201
AP000769.5−201
SIPA1−203
MALAT1−215
RNASEH2C−211
MAP3K11−201
OVOL1−AS1−203
chr11.345
65.2 mb
65.4 mb
65.6 mb
65.8 mb
66 mb
66.2 mb
65.3 mb
65.5 mb
65.7 mb
65.9 mb
66.1 mb
chr11.345
0.75
0.50
0.25
0.00

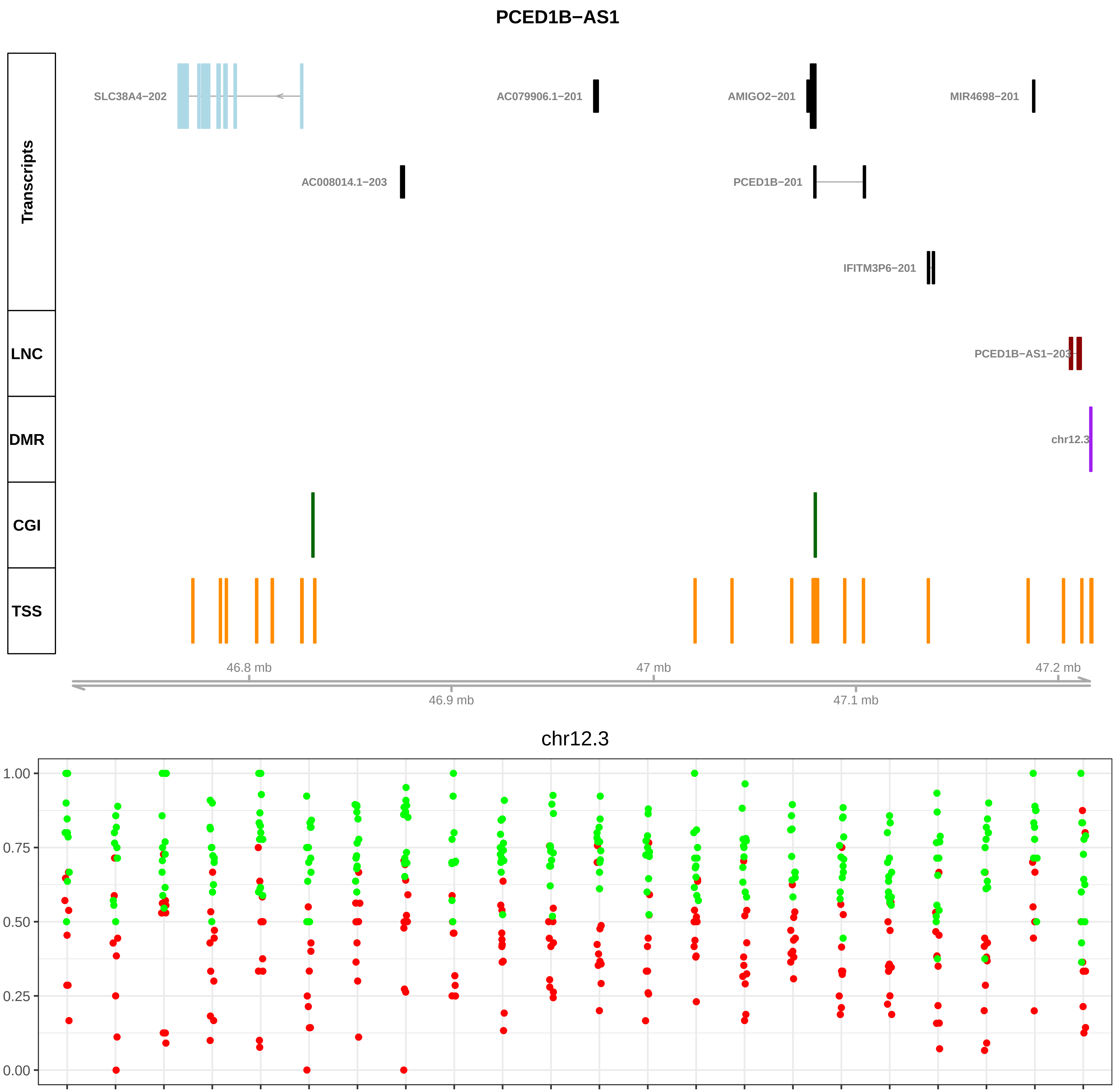

PCED1B−AS1
Transcripts
LNC
DMR
CGI
TSS
SLC38A4−202
AC079906.1−201
AMIGO2−201
MIR4698−201
AC008014.1−203
PCED1B−201
IFITM3P6−201
PCED1B−AS1−203
chr12.3
46.8 mb
47 mb
47.2 mb
46.9 mb
47.1 mb
chr12.3
1.00
0.75
0.50
0.25
0.00

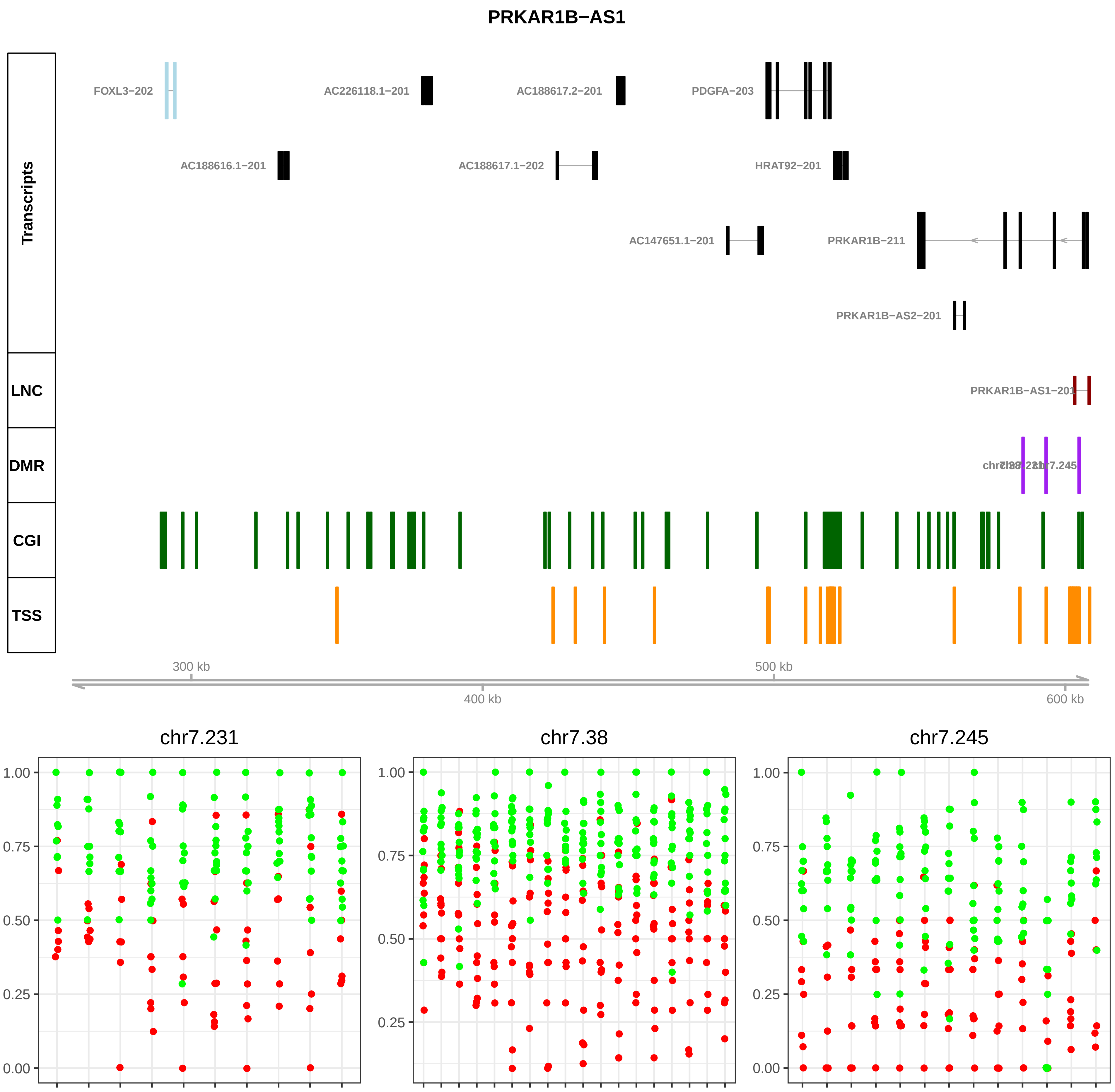

PRKAR1B−AS1
Transcripts
LNC
DMR
CGI
TSS
FOXL3−202
AC226118.1−201
AC188617.2−201
PDGFA−203
AC188616.1−201
AC188617.1−202
HRAT92−201
AC147651.1−201
PRKAR1B−211
PRKAR1B−AS2−201
PRKAR1B−AS1−201
300 kb
400 kb
500 kb
600 kb
chr7.231
chr7.38
chr7.245
0.00
0.25
0.50
0.75
1.00

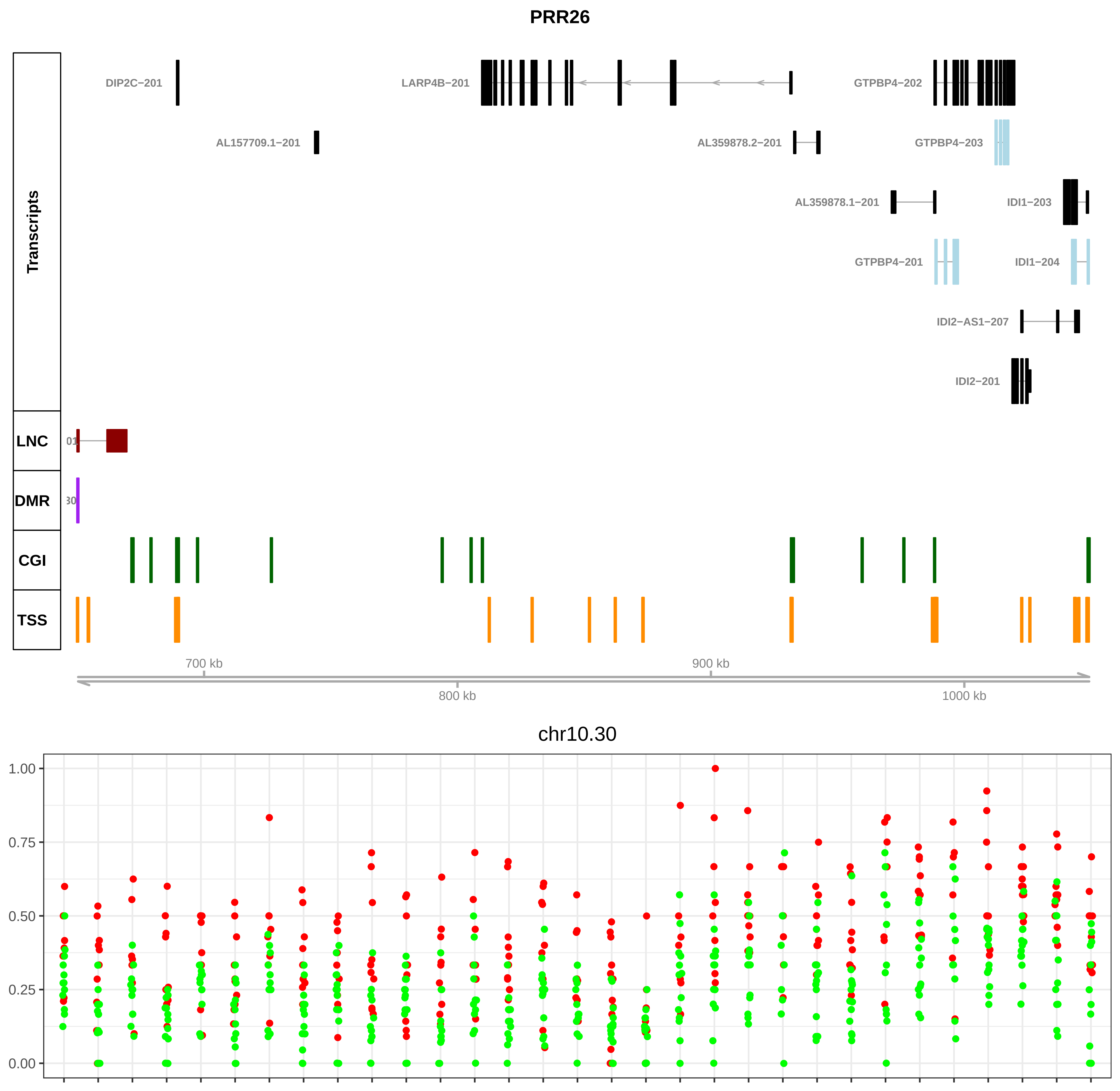

PRR26
Transcripts
DIP2C−201
LARP4B−201
GTPBP4−202
AL157709.1−201
AL359878.2−201
GTPBP4−203
AL359878.1−201
IDI1−203
GTPBP4−201
IDI1−204
IDI2−AS1−207
IDI2−201
LNC
DMR
CGI
TSS
700 kb
900 kb
800 kb
1000 kb
chr10.30
1.00
0.75
0.50
0.25
0.00

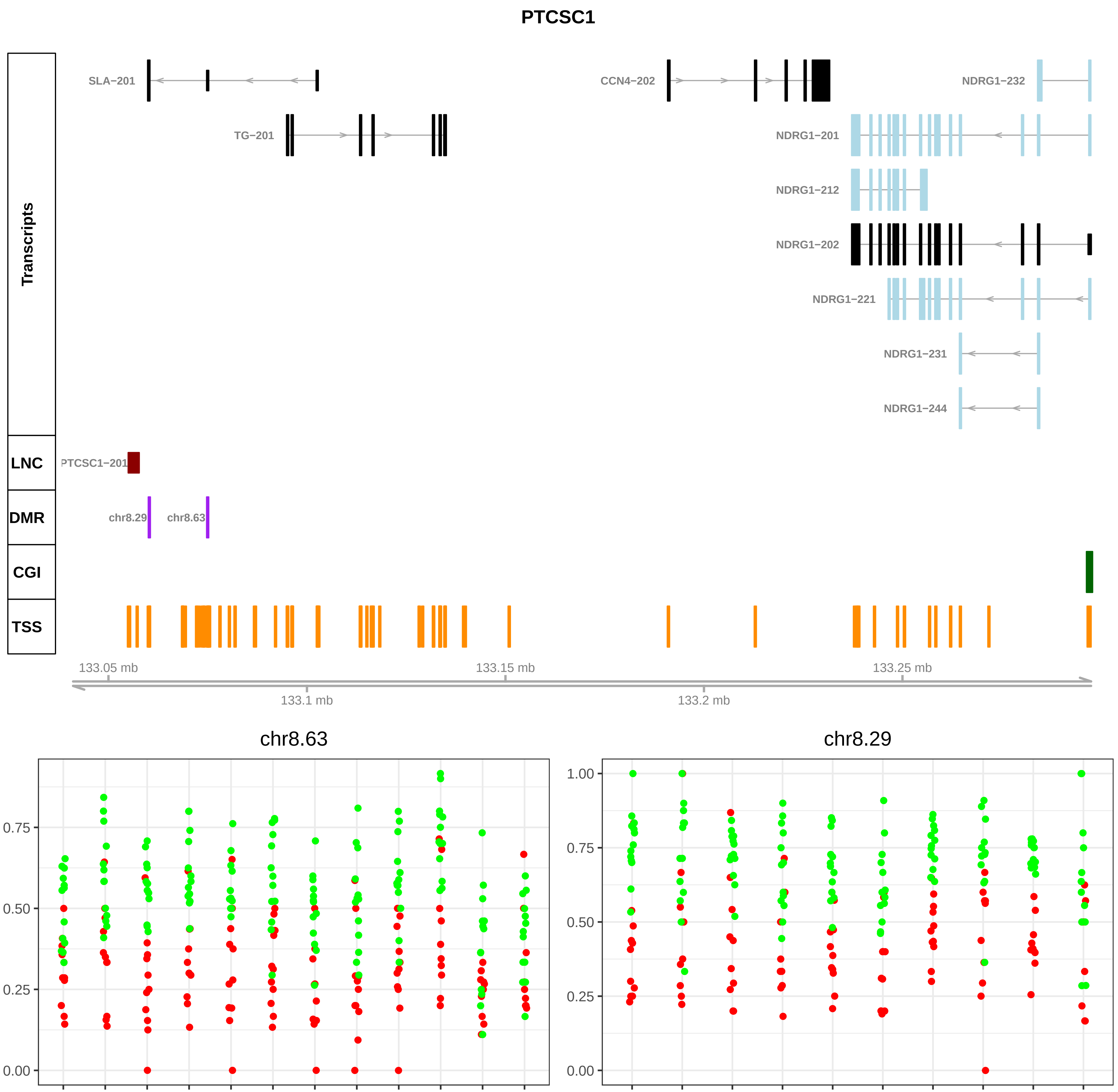
PTCSC1
Transcripts
SLA−201
CCN4−202
NDRG1−232
TG−201
NDRG1−201
NDRG1−212
NDRG1−202
NDRG1−221
NDRG1−231
NDRG1−244
LNC
PTCSC1−201
DMR
chr8.29
chr8.63
CGI
TSS
133.05 mb
133.15 mb
133.25 mb
133.1 mb
133.2 mb
chr8.63
chr8.29
0.00
0.25
0.50
0.75
1.00

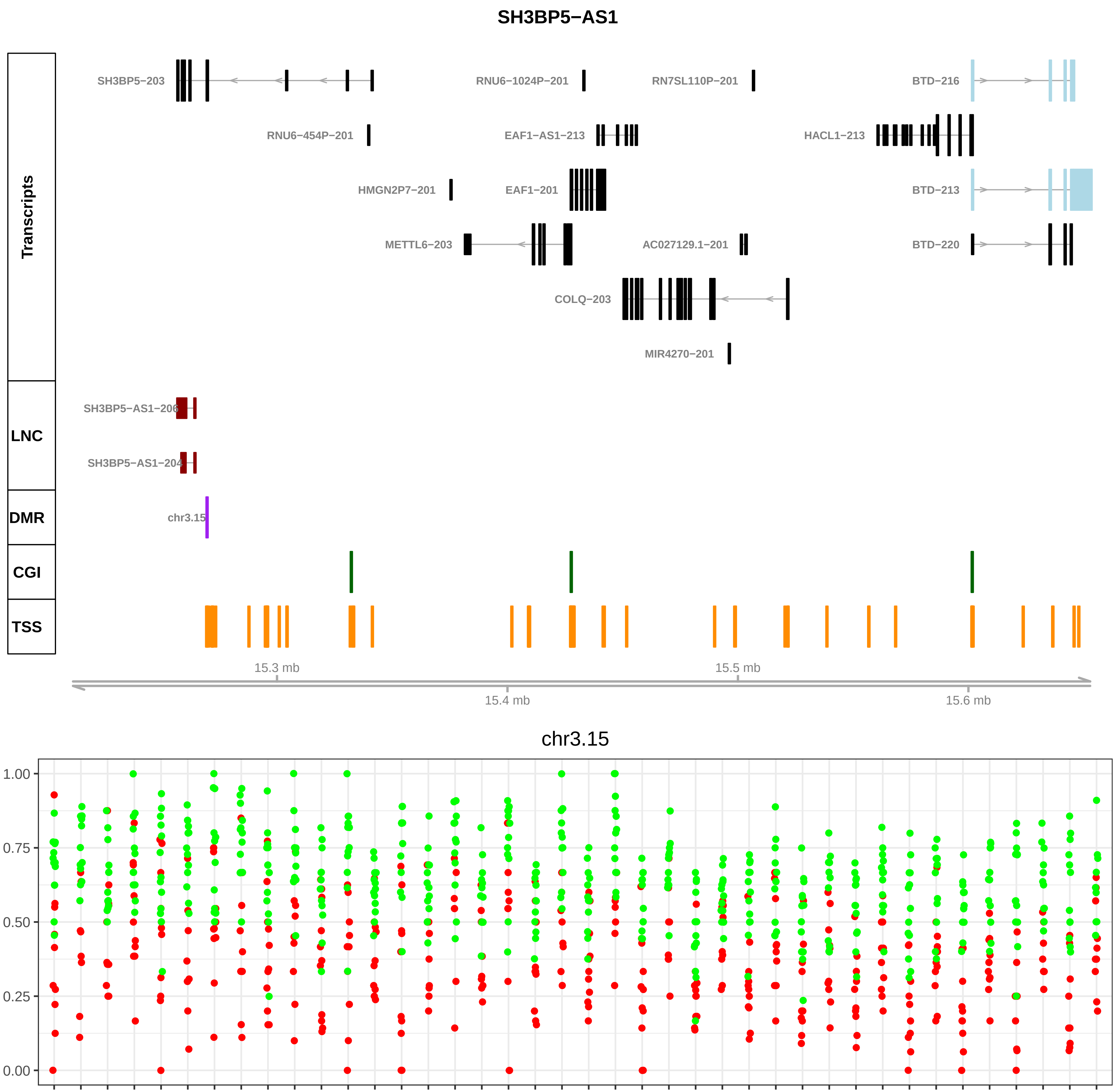

SH3BP5−AS1
Transcripts
SH3BP5−203
RNU6−1024P−201
RN7SL110P−201
BTD−216
RNU6−454P−201
EAF1−AS1−213
HACL1−213
HMGN2P7−201
EAF1−201
BTD−213
METTL6−203
AC027129.1−201
BTD−220
COLQ−203
MIR4270−201
LNC
SH3BP5−AS1−206
SH3BP5−AS1−204
DMR
chr3.15
CGI
TSS
15.3 mb
15.5 mb
15.4 mb
15.6 mb
chr3.15
1.00
0.75
0.50
0.25
0.00

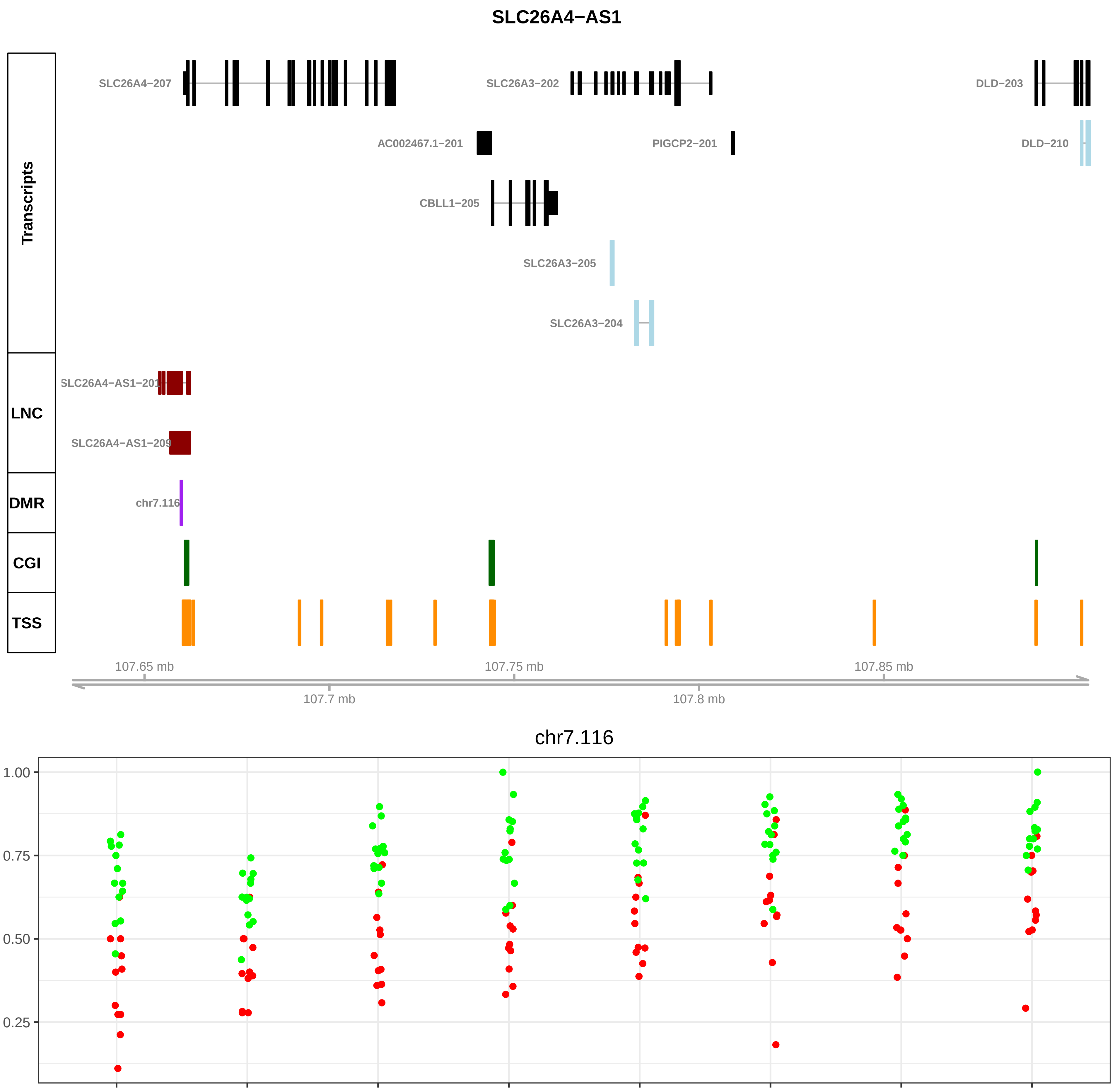

SLC26A4−AS1
Transcripts
SLC26A4−207
SLC26A3−202
DLD−203
AC002467.1−201
PIGCP2−201
DLD−210
CBLL1−205
SLC26A3−205
SLC26A3−204
LNC
SLC26A4−AS1−201
SLC26A4−AS1−209
DMR
chr7.116
CGI
TSS
107.65 mb
107.75 mb
107.85 mb
107.7 mb
107.8 mb
chr7.116
1.00
0.75
0.50
0.25

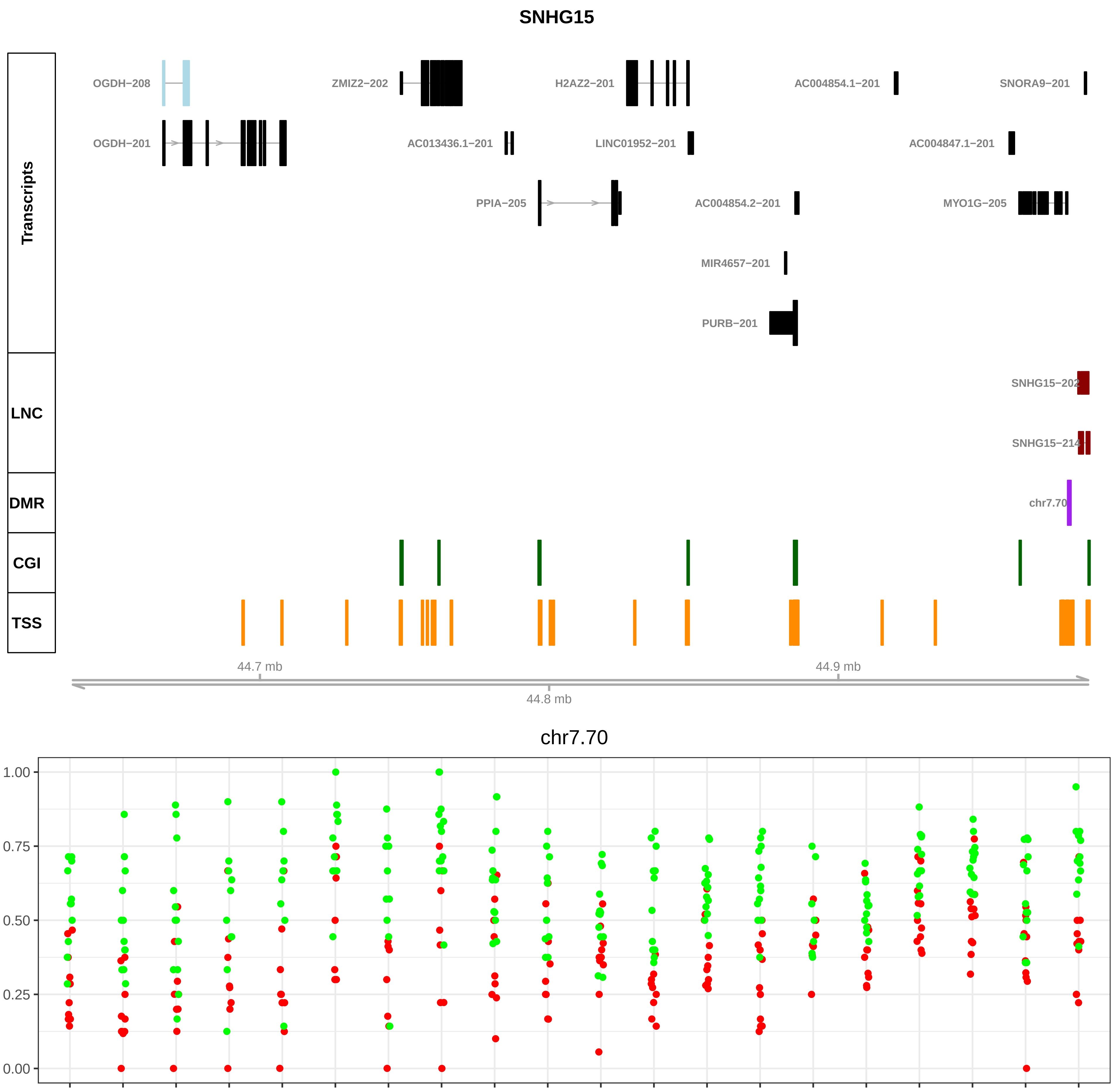
SNHG15
Transcripts
LNC
DMR
CGI
TSS
OGDH−208
ZMIZ2−202
H2AZ2−201
AC004854.1−201
SNORA9−201
OGDH−201
AC013436.1−201
LINC01952−201
AC004847.1−201
PPIA−205
AC004854.2−201
MYO1G−205
MIR4657−201
PURB−201
SNHG15−202
SNHG15−214
chr7.70
44.7 mb
44.8 mb
44.9 mb
chr7.70
1.00
0.75
0.50
0.25
0.00

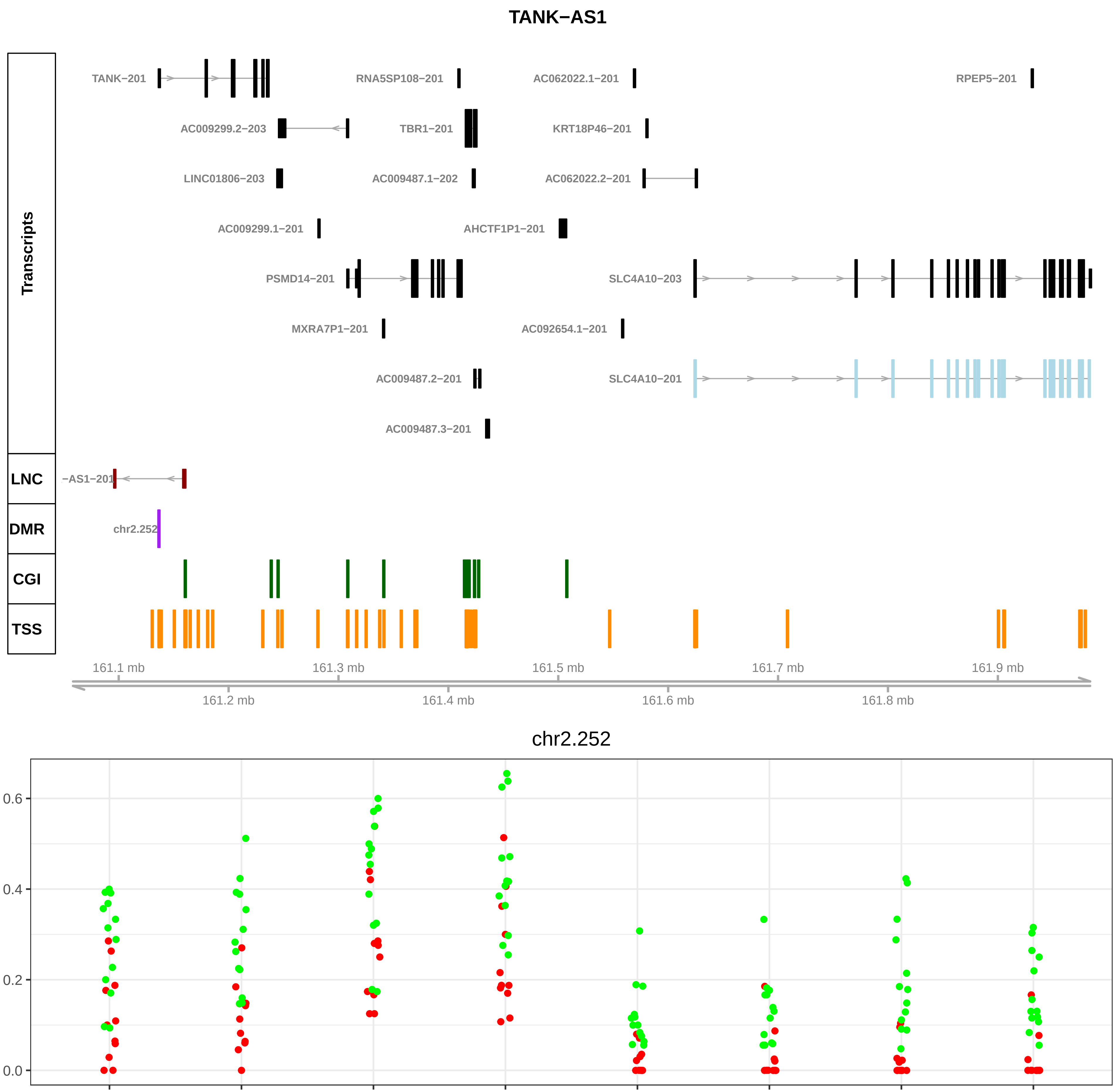

TANK−AS1
Transcripts
TANK−201
RNA5SP108−201
AC062022.1−201
RPEP5−201
AC009299.2−203
TBR1−201
KRT18P46−201
LINC01806−203
AC009487.1−202
AC062022.2−201
AC009299.1−201
AHCTF1P1−201
PSMD14−201
SLC4A10−203
MXRA7P1−201
AC092654.1−201
AC009487.2−201
SLC4A10−201
AC009487.3−201
LNC
−AS1−201
DMR
chr2.252
CGI
TSS
161.1 mb
161.3 mb
161.5 mb
161.7 mb
161.9 mb
161.2 mb
161.4 mb
161.6 mb
161.8 mb
chr2.252
0.6
0.4
0.2
0.0

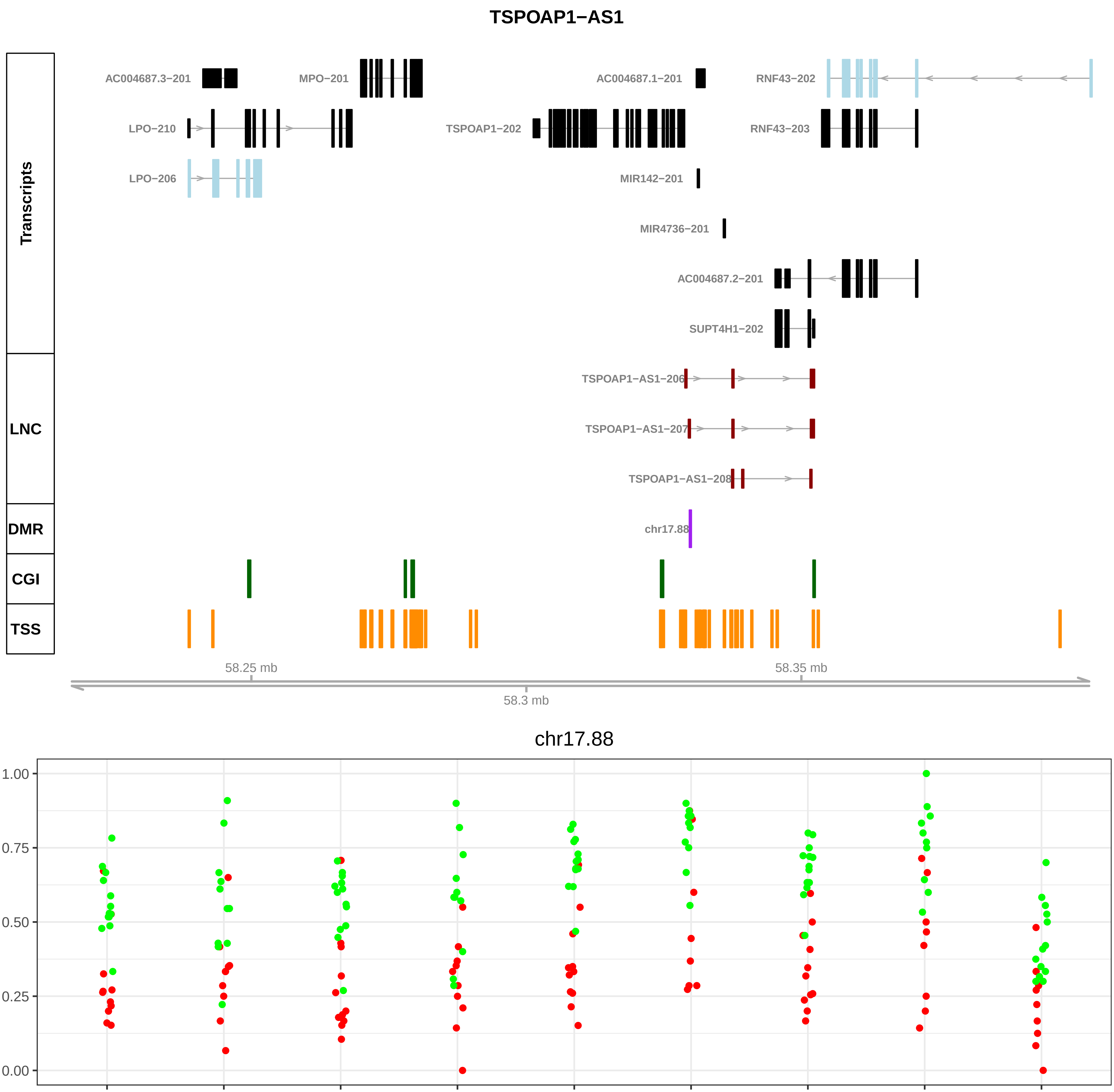

TSPOAP1−AS1
Transcripts
LNC
DMR
CGI
TSS
AC004687.3−201
MPO−201
AC004687.1−201
RNF43−202
LPO−210
TSPOAP1−202
RNF43−203
LPO−206
MIR142−201
MIR4736−201
AC004687.2−201
SUPT4H1−202
TSPOAP1−AS1−206
TSPOAP1−AS1−207
TSPOAP1−AS1−208
chr17.88
58.25 mb
58.35 mb
58.3 mb
chr17.88
1.00
0.75
0.50
0.25
0.00

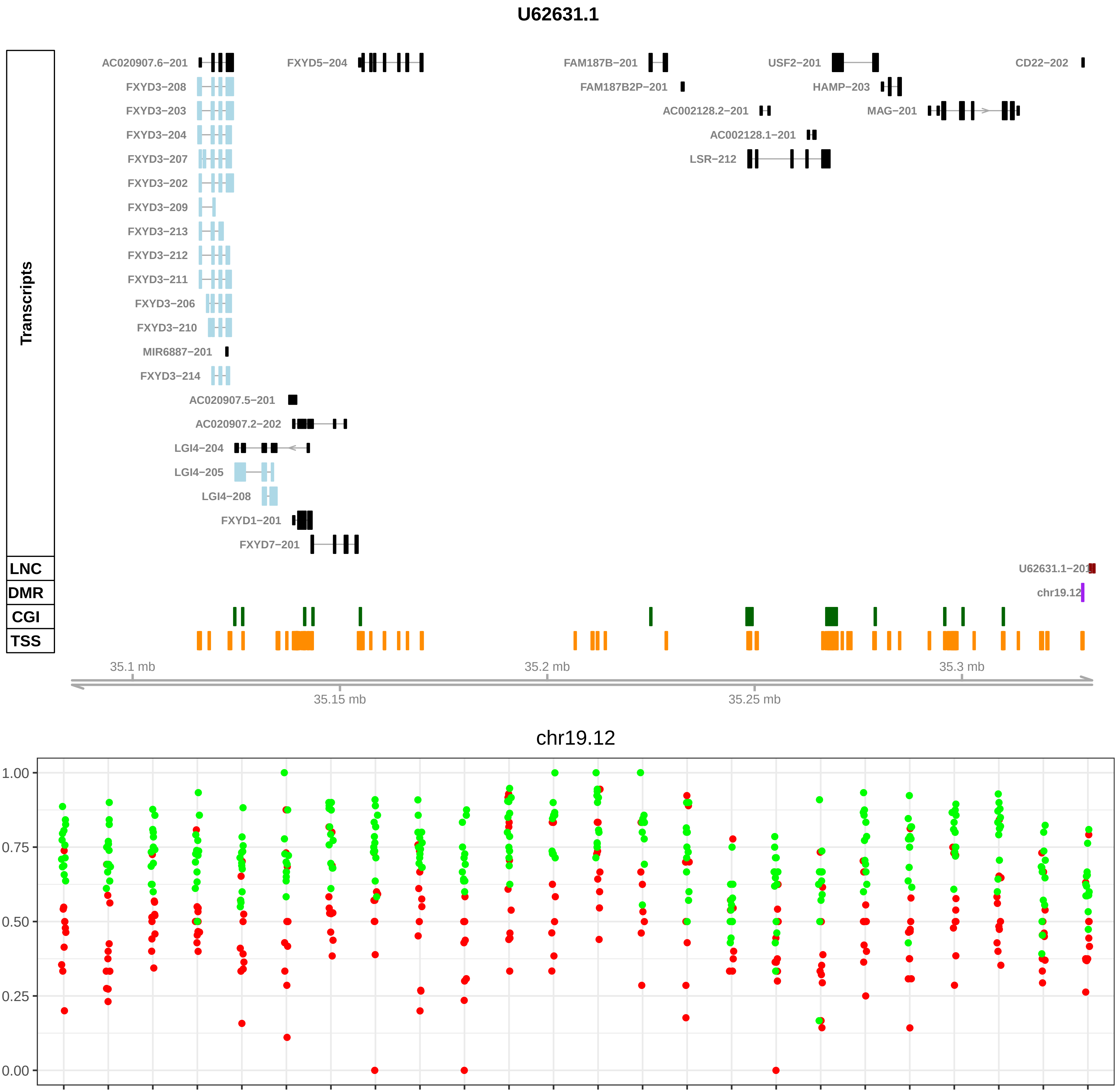

U62631.1
Transcripts
AC020907.6−201
FXYD5−204
FAM187B−201
USF2−201
CD22−202
FXYD3−208
FAM187B2P−201
HAMP−203
FXYD3−203
AC002128.2−201
MAG−201
FXYD3−204
AC002128.1−201
FXYD3−207
LSR−212
FXYD3−202
FXYD3−209
FXYD3−213
FXYD3−212
FXYD3−211
FXYD3−206
FXYD3−210
MIR6887−201
FXYD3−214
AC020907.5−201
AC020907.2−202
LGI4−204
LGI4−205
LGI4−208
FXYD1−201
FXYD7−201
LNC
U62631.1−201
DMR
chr19.12
CGI
TSS
35.1 mb
35.2 mb
35.3 mb
35.15 mb
35.25 mb
chr19.12
1.00
0.75
0.50
0.25
0.00

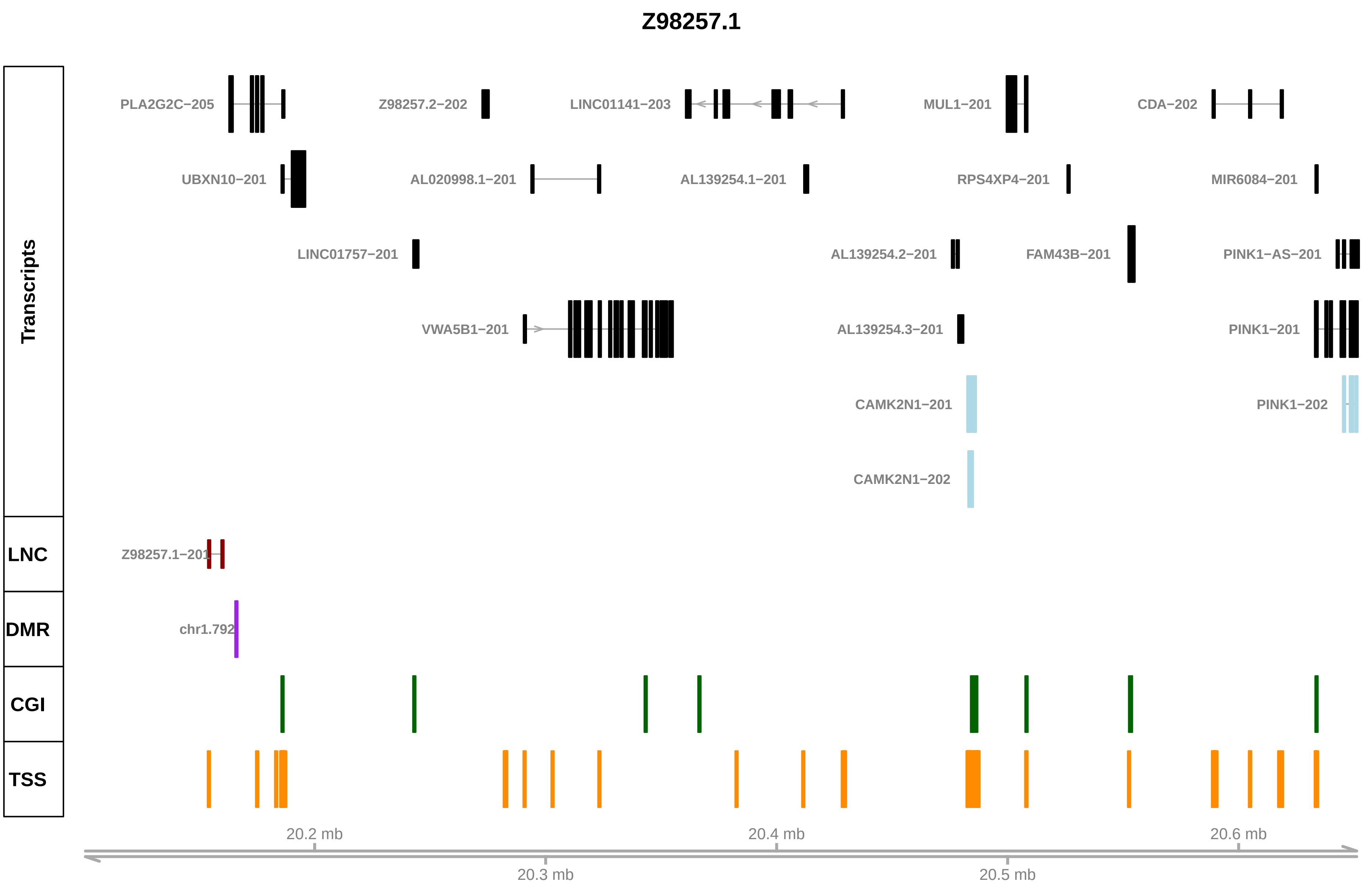
Z98257.1
Transcripts
PLA2G2C−205
Z98257.2−202
LINC01141−203
MUL1−201
CDA−202
UBXN10−201
AL020998.1−201
AL139254.1−201
RPS4XP4−201
MIR6084−201
LINC01757−201
AL139254.2−201
FAM43B−201
PINK1−AS−201
VWA5B1−201
AL139254.3−201
PINK1−201
CAMK2N1−201
PINK1−202
CAMK2N1−202
LNC
Z98257.1−201
DMR
chr1.792
CGI
TSS
20.2 mb
20.4 mb
20.6 mb
20.3 mb
20.5 mb

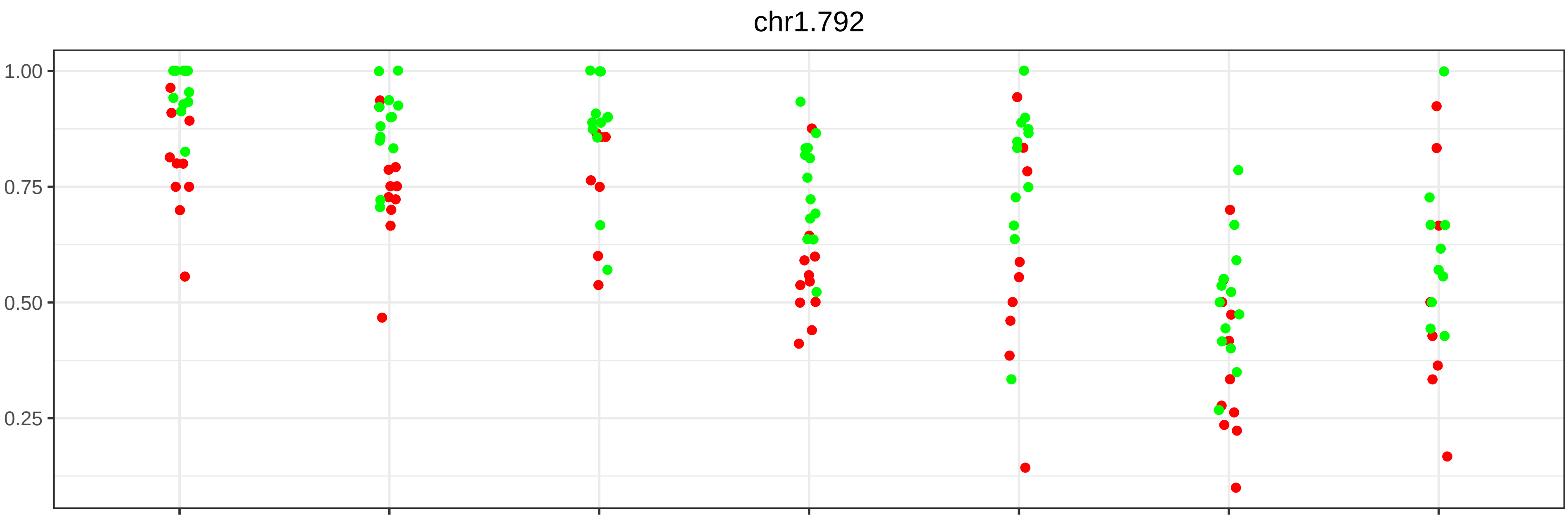
chr1.792
1.00
0.75
0.50
0.25

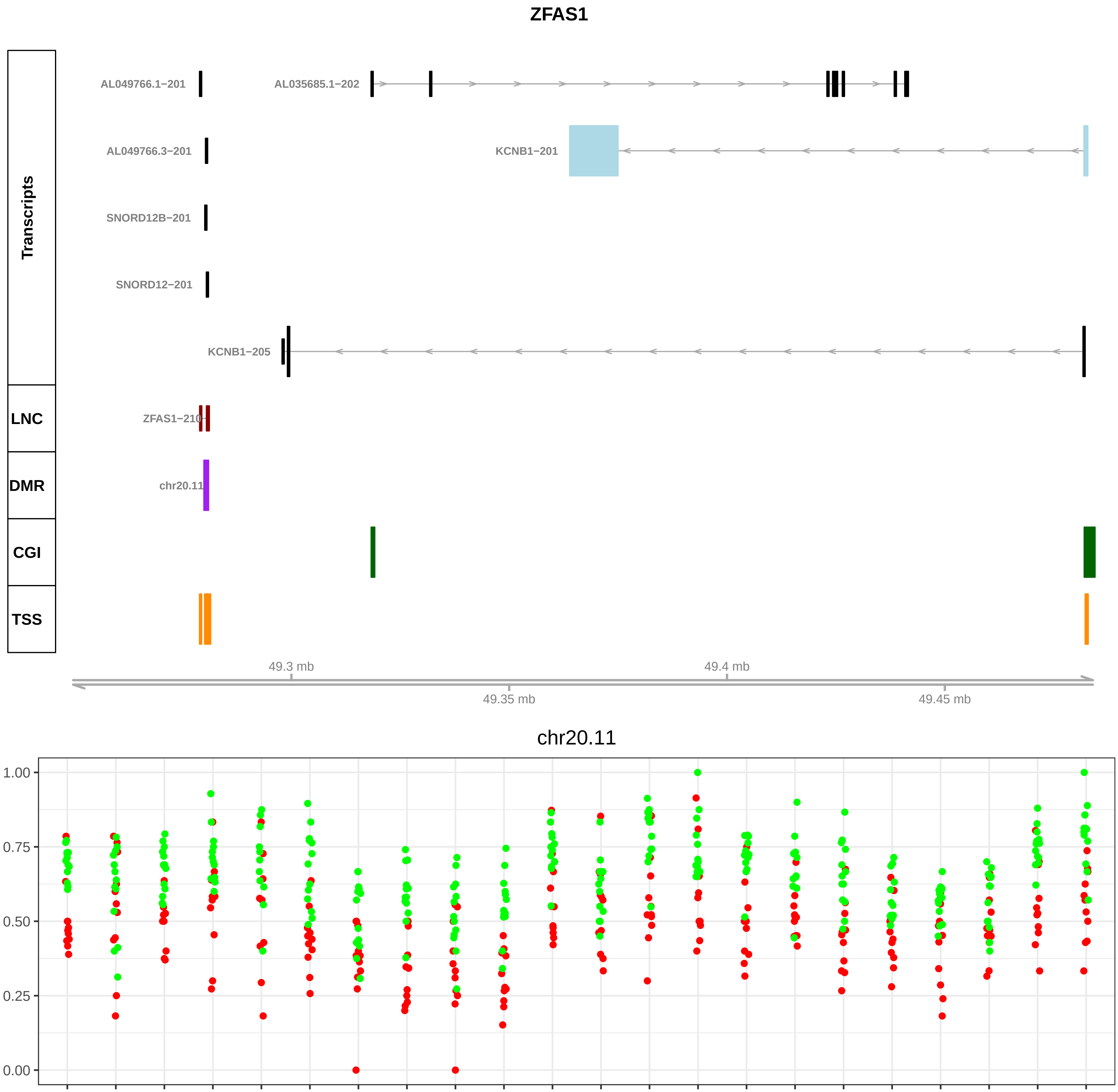

ZFAS1
Transcripts
AL049766.1−201
AL035685.1−202
AL049766.3−201
KCNB1−201
SNORD12B−201
SNORD12−201
KCNB1−205
LNC
ZFAS1−210
DMR
chr20.11
CGI
TSS
49.3 mb
49.4 mb
49.35 mb
49.45 mb
chr20.11
1.00
0.75
0.50
0.25
0.00
